# Revised geochronology, correlation, and dinosaur stratigraphic ranges of the Santonian-Maastrichtian (Late Cretaceous) formations of the Western Interior of North America

**DOI:** 10.1371/journal.pone.0188426

**Published:** 2017-11-22

**Authors:** Denver Warwick Fowler

**Affiliations:** Dickinson Museum Center, Dickinson, North Dakota, United States of America; Indiana University Bloomington, UNITED STATES

## Abstract

Interbasinal stratigraphic correlation provides the foundation for all consequent continental-scale geological and paleontological analyses. Correlation requires synthesis of lithostratigraphic, biostratigraphic and geochronologic data, and must be periodically updated to accord with advances in dating techniques, changing standards for radiometric dates, new stratigraphic concepts, hypotheses, fossil specimens, and field data. Outdated or incorrect correlation exposes geological and paleontological analyses to potential error. The current work presents a high-resolution stratigraphic chart for terrestrial Late Cretaceous units of North America, combining published chronostratigraphic, lithostratigraphic, and biostratigraphic data. ^40^Ar / ^39^Ar radiometric dates are newly recalibrated to both current standard and decay constant pairings. Revisions to the stratigraphic placement of most units are slight, but important changes are made to the proposed correlations of the Aguja and Javelina formations, Texas, and recalibration corrections in particular affect the relative age positions of the Belly River Group, Alberta; Judith River Formation, Montana; Kaiparowits Formation, Utah; and Fruitland and Kirtland formations, New Mexico. The stratigraphic ranges of selected clades of dinosaur species are plotted on the chronostratigraphic framework, with some clades comprising short-duration species that do not overlap stratigraphically with preceding or succeeding forms. This is the expected pattern that is produced by an anagenetic mode of evolution, suggesting that true branching (speciation) events were rare and may have geographic significance. The recent hypothesis of intracontinental latitudinal provinciality of dinosaurs is shown to be affected by previous stratigraphic miscorrelation. Rapid stepwise acquisition of display characters in many dinosaur clades, in particular chasmosaurine ceratopsids, suggests that they may be useful for high resolution biostratigraphy.

## Introduction

In 1952, Cobban and Reeside [1] published a grand correlation of Cretaceous rocks of the Western Interior of central and southern North America, including both marine and terrestrial units, and biostratigraphic ranges for a variety of invertebrates and vertebrates. Such interbasinal correlation diagrams are enormously useful for making stratigraphic comparisons between units and similar style diagrams have become commonplace in the geological literature. Recent, broad-scale correlations akin to that of Cobban and Reeside [1] are less common, but examples include Krystinik and DeJarnett [2], Sullivan and Lucas [3, 4], Miall et al. [5], and Roberts et al. [6]. Construction of these kinds of correlation charts is built upon a great wealth of literature; the product of dedicated work by generations of stratigraphers working in the Western Interior. Individual papers doubtless number in the thousands, and there are far too many to mention directly here, although many are cited in the supporting information (see S1 Table and S1 Text).

Interbasinal correlation charts are not just of use to geologists; more frequently than ever, paleontologists are using high-resolution chronostratigraphic data to formulate and test evolutionary hypotheses. A simple example is that of time-calibrated phylogenies, where the stratigraphic positions of individual taxa are superimposed on phylogenetic trees. These are becoming much more prominent in the dinosaur literature (e.g. [7–10]), and are used to deduce the timing of important phylogenetic branching events, infer ghost ranges, and potentially to calculate rates of evolution. A more nuanced application is assessment of whether two sister taxa are contemporaneous (thereby inferring a genuine speciation event), or whether they form a succession of stratigraphically separated morphologies (potentially supportive of anagenesis; e.g. [9, 11–13]). The value of such analyses is inherently dependent upon the accuracy of the plotted taxa, which in turn depend upon the accuracy of the stratigraphic correlations of the formations from which their fossils were recovered. Herein lies the problem. Precise dating of geological formations is especially critical for testing anagenesis or cladogenesis in dinosaurs [8, 11], but when specimens are very similar in age, imprecision of only a few hundred thousand years is often enough to completely reverse paleobiological interpretation.

The Upper Cretaceous deposits of the North American Western Interior represent a rare opportunity to make a high-resolution chronostratigraphic framework within which to study dinosaur evolution. This is due to the serendipitous combination of large areas of outcrop, interfingering marine units with biostratigraphically informative fossils, and a consistent scattering of radiometric dates due to synorogenic volcanic activity, not to mention a vast literature detailing over a century’s worth of research and an ever increasing collection of fossils. However, despite the large amount of data available, some problems persist that strongly affect paleobiological interpretations:

### 1. It is difficult to find the reasoning behind some correlations

In paleontological papers especially, correlation charts are typically presented as a series of geological columns, and rarely contain detailed justifications for the stratigraphic positions of the depicted horizons. Usually a few citations are given for stratigraphic position, and radiometric dates may be marked (also including citations), but important details may be lacking. This can create many problems, including circular citation of incorrect or unknown stratigraphic data or unknowingly mismatching old outdated stratigraphic data with new interpretations or calibrations (see Discussion for detailed explanation of examples). Admittedly, justifying every boundary or horizon in a stratigraphic column is an arduous task, but without detailed work like this, precise stratigraphic placement of taxa can be either impossible or plotted incorrectly.

### 2. Depositional hiatuses are not depicted

The normal method of illustrating stratigraphic columns often does not include illustration of the depositional hiatuses (lacunae) that exist within formations, and this can affect the perception of unit duration, conformability, and magnetostratigraphic relationships. For example, under conventional lithostratigraphic practice, prominent sandstones are sometimes chosen as uppermost units for formational contacts (e.g. the Capping Sandstone Member, Wahweap Formation, Utah [14]). However, under the conventional sequence stratigraphic model, amalgamated channel sandstones often form the basalmost unit of depositional cycles, resting upon a surface of erosion or depositional hiatus; i.e., the basal bed of a conformable cycle might simultaneously be considered the uppermost unit of a lithostratigraphic formation. For this and other reasons, formation members and the lacunae between them should be plotted on correlation charts where possible.

### 3. Radiometric dates may be incorrect or incomparable

Many currently cited radiometric dates are not properly comparable, because from the early 1980's to the current day radiometric analyses have used a variety of standards, decay constants, or different methods. In order to rectify this, historical dates have been recalibrated by previous workers (e.g. for the Western Interior [6, 13, 15–18]). There is also an emerging issue that analyses performed in different laboratories under slightly different methodologies produce slightly different results, and this is being investigated internally by those labs

This current work presents a comprehensive stratigraphic correlation chart comprising the major terrestrial geological formations of the Santonian through Maastrichtian of the North American Western Interior (S1 Table). The chart is plotted based on extensive reference to the stratigraphic literature on each formation (which is reviewed and cited in detailed notes for each unit), and on the recalibration of ^40^Ar / ^39^Ar radiometric dates. Over 200 recalibrated radiometric dates are presented as a separate excel sheet (S2 Table), and are recalibrated to both currently accepted ^40^Ar / ^39^Ar standards and decay constant pairings (Kuiper et al. [16]combined with the decay constant values of Min et al. [19]and Renne et al. [20]). The resultant stratigraphic framework is used in combination with locality data for individual dinosaur specimens to plot the stratigraphic ranges for dinosaur taxa (currently restricted to Neoceratopsia, Sauropoda, Pachycephalosauridae, and Hadrosauridae). This replotting of dinosaur taxa is discussed with regard to current hypotheses of dinosaur biogeography and evolution.

## Methods

### Display format—Excel sheets

The recalibration sheet and stratigraphic correlation chart are offered as two separate excel files (S1 and S2 Tables). They are kept separate for ease of cross referencing.

The stratigraphic correlation chart is arranged as an Excel spreadsheet (S1 Table), and is intended to be used directly in this format as it offers a number of advantages over a graphic embedded within a PDF or printed page. The grid of cells naturally permit precise plotting of stratigraphic boundaries, with each vertical cell height representing 0.1 m.y. Most usefully, each cell, (or group of cells) can be tagged with a pop-up note that is activated by simply hovering the mouse cursor over any cell with a red triangle in the upper right corner. These pop-up notes comprise the bulk of the results of this study, providing the information that supports each depicted stratigraphic position or boundary of the geologic unit or taxon, along with introductory text. For ideal formatting, the reader is advised to view the chart in native resolution, at 22% zoom level.

Some disadvantages of the Excel format include the limited range of line styles and orientations, such that (for example) it is not possible to represent unconformities by a wavy line, and cell borders necessarily are straight. Due to the need to keep font size small (to increase available space), taxon names are not produced in italics as it makes them much less readable. The reader is advised that under some levels of zoom, a note box might not be fully readable; if so, right click and select edit note, then either read the note in place, or resize the note box such that all the text is visible.

References used in the construction of the chart are available as a separate document (S1 Text).

The recalibration sheet (S2 Table) is also made available in the form of an Excel sheet. This is due to its large size, but also benefits from the pop-up note function, providing additional information on radiometric dates and the original publications. Maintaining the recalibrations as an Excel sheet also permits the retention of the active formulae used to calculate the new dates.

The recalibration sheet is adapted from the EARTHTIME excel recalculation sheet kindly provided by Noah McLean at the earth-time.org website [21]. Unfortunately, the main homepage of the earth-time.org website is currently listed as "under construction"; however, the direct link to the recalibration spreadsheet and instructions download page is still active as of October 28th 2017: http://www.earth-time.org/ar-ar.html. Note that a similar recalibration sheet was provided by Paul Renne (pers. comm. 2012).

The original recalibration formulae were duplicated across into S2 Table such that this is a "live" document that independently recalculates dates based on the input data on each line of the sheet. The source lines for each recalculation have been adapted from the original EARTHTIME recalculation sheet [21] such that in S2 Table all the original input data (standards, decay constant, etc.) are visible for each recalculation. This way the sheet shows all the "working" for all of the ~200 recalculations, and each can be properly independently assessed.

There is an issue with the recalculation of error in the original formulae present in the McLean EARTHTIME sheet [21]. This has the result that for some recalibrations, the excel sheet will only produce a "!VALUE" statement for the recalibrated uncertainty/error (caused by the formula attempting to divide by zero). As a result, the uncertainty/error for many recalibrations cannot be computed (an additional problem is the lack of J-value data in most analyses). To overcome this, for analyses in which the new error cannot be directly computed, the original error has been multiplied by the % change output factor; errors calculated by this method are shown in red (normally calculated error values are shown in black). Comparison to normally calculated error values show that this method produces comparable results such that the new stated error values are not significantly different from what would be calculated if J-values (etc) were known.

There are two tabs of recalibrations. The first, labeled "Kuiper et al 2008", recalibrates all the dates to the Kuiper et al. [16] FCT standard, coupled with the Min et al. [19] decay constant. Dates from this first tab are plotted on the stratigraphic chart (S1 Table). The second tab, labeled "Renne et al 2011", recalibrates all dates to the standard and decay constant pairing of Renne et al. [22]. This second set of recalibrations is provided for comparison. Both tables of recalibrations have the same formatting for ease of comparison.

### Stratigraphic chart (S1 Table)

Construction of the chart is complex and depends upon many different stratigraphic methods. The following text explains the underlying definitions that provide the base framework for the chart, and highlight some of the issues surrounding its construction.

#### Definitions: Stage and substages, magnetostratigraphy, and ammonite biostratigraphy

The chart follows The Geological Time Scale 2012 (GTS2012 [23]) for definitions of stage and substage boundaries [24], magnetostratigraphic boundaries [25], and ammonite biostratigraphy [24]. Although more recent revisions of these definitions are available, GTS2012 integrates all these defined units with chronostratigraphic dates that use the ^40^Ar /^39^Ar standard and decay constant pairing of Kuiper et al. [16] and Min et al. [19], which are also used here. A second magnetostratigraphic column is also offered containing some revised chron boundaries and includes many of the very short duration cryptochrons that have not yet been officially recognised, but are often named in magnetostratigraphic analyses (e.g. [26]). Individual definitions and discussion (where appropriate) can be found in the pop-up notes in the respective parts of the chart.

In some places it is necessary to provide a compromise in stratigraphic placement, generally where a magnetostratigraphic assertion does not match, say, the ammonite zonation (e.g. age of the Dorothy bentonite in the Drumheller Member, Horseshoe Canyon Formation, Alberta [27, 28]. In such cases, the pop-up note text boxes provide explanation of the problem, and references.

#### Positioning of geological units and dinosaur taxa

The stratigraphic ranges of geological units and fossil taxa are plotted as a solid bordered white cell with the lower and upper borders representing the lower and upper contacts of the geological unit, or first and last documented taxon occurrences (respectively).

If stratigraphic position is not sufficiently documented, the possible or likely stratigraphic range is illustrated as a block arrow. For example, if we know a taxon occurs in a given formation, but not the precise stratigraphic position within that formation (or if the age of the formation itself is only roughly known), then the block arrow would show the possible range being equivalent to the full duration of the formation. A combination of a solid cell and block arrow may be used if a taxon or geological unit comprises some specimens or horizons for which stratigraphic position is precisely known (depicted by the solid cell) and some specimens or horizons for which stratigraphic position is unknown (block arrows). Periods of non-material time (lacunae) are represented by blank spaces. In the aforementioned cases, explanation for the plotting of geological units, lacunae, and taxa is given in the corresponding note A graded block arrow is used for units which may continue for a long time below the period of interest (typically used for thick marine shales). The ranges and boundaries of each taxon or geological unit are discussed on a case-by-case basis in S1 Table.

#### Issues with lithostratigraphy

Some features of typical lithostratigraphic units are not possible to depict properly on the stratigraphic chart format. In the Western Interior, many terrestrial packages form clastic wedges thinning basinward. Where possible, it is attempted to represent this in the chart, although for the most part depicted stratigraphic sections are based on single well-sampled sections, cores, or geographic areas, so the wedge-shaped overall geometry might not be visible.

#### Limitation of scope & future versions

There are some limitations of scope for this initial version of the correlation chart.

The chart is currently mostly limited to units of Santonian age (86.3 Ma) up to the K-Pg boundary (66.0 Ma). There are a few exceptions (e.g. Moreno Hill Formation, New Mexico; Straight Cliffs Formation, Utah), which are included because they have yielded important specimens, or provide stratigraphic context for overlying units.

Geological units featured in the correlation chart are currently limited to those for which dinosaurian material has been collected, or which provide contextual information for surrounding units (e.g. intertonguing marine units with biostratigraphically informative fauna, and overlying or underlying units with chronostratigraphic marker beds).

Dinosaurian fossils are limited to Neoceratopsia, Pachycephalosauridae, Sauropoda, and Hadrosauridae. This is partly to limit the amount of data in this first published version of the chart. Thus, the chosen clades represent the most abundant taxa, and also include taxa considered biostratigraphically informative by previous workers (e.g. [1, 3, 4, 29, 30]).

Future versions of the chart are intended to extend the stratigraphic range down to the Jurassic-Cretaceous boundary. However, the plans for the first expansion concern inclusion of more Upper Cretaceous formations from North America, and also similarly aged deposits in Asia. Initial work on expanding faunal coverage has already begun concerning the addition of all remaining dinosaur taxa (including birds), crocodylians, and mammals, with the intent of eventually incorporating all useful taxa if possible.

#### Institutional abbreviations

A list of institutional abbreviations used in the correlation chart are provided in a separate tab of the correlation chart Excel sheet (S1 Table) labeled "repository codes".

#### Taxa display format- phylogenies and lineages

It is not the intention of this project to make significant comment on phylogenies *per se*. However, precise stratigraphic placement of taxa permits testing of speciation hypotheses (see Discussion), and so the arrangement of taxa on the chart should reflect up-to-date phylogenies or other hypotheses of descent. In this current version, this only affects Ceratopsidae and Hadrosauridae. For ceratopsids, the general arrangement follows the chasmosaurine phylogeny from Fowler [31], and for centrosaurines the arrangement follows the phylogeny of Evans and Ryan [32]. For hadrosaurids, the general arrangement of hadrosaurines follows Freedman Fowler and Horner [13], and lambeosaurines follows Evans and Reisz [33].

#### Magnetostratigraphy

The conventional methodology used for delineating magnetostratigraphic chron boundaries can create problems. In magnetostratigraphic analysis, if two stratigraphically adjacent sample points yield opposite polarities (i.e., they are recognisable as different chrons), then it is convention to draw the chron boundary stratigraphically halfway between the two points. However, an issue can arise if these lower and upper sample points are separated by a sandstone from which it is difficult or impossible to extract a magnetostratigraphic signal. In terrestrial floodplain deposition (typical of the units studied in this work), the bases of depositional cycles are characterized by a surface of non-deposition or erosion overlain by a low accommodation systems tract, typically comprising an amalgamated channel sandstone. The combination of the depositional hiatus at the base of the sandstone, and the sandstone itself, means that there may be a considerable time gap (up to millions of years) between the last sampled horizon immediately below the sandstone, and the first sampled horizon immediately above the sandstone. If opposite polarities are recorded for the two sampled horizons on either side of the unsampled sandstone, then the chron boundary would be drawn halfway, within the sandstone, whereas it might be more likely to occur at the base of the sandstone, as this is where the hiatus occurs. This would have the effect of making a unit appear older or younger than it really is. For example, the mudstone immediately beneath the Apex sandstone (basal unit of the upper Hell Creek Formation, Montana [34]) is of normal polarity, assigned to C30n, whereas the mudstone immediately above the Apex Sandstone is of reversed polarity (assigned to C29r [35]). The C30n-C29r boundary is therefore drawn within the Apex Sandstone, whereas it is more likely that it occurs at the hiatus at the base of the sandstone. A more significant case arises with the contact between the Laramie Formation and overlying D1 sequence in central Colorado: here, because of the halfway convention, the uppermost part of the Laramie is drawn as being within the lowermost C31r zone [36], whereas in actuality, all magnetostratigraphic samples recovered by Hicks et al. [36] from the Laramie are normal, and it might therefore be entirely C31n. The effects of this issue are best examined on a case by case basis; the reader is referred to the stratigraphic chart (S1 Table) where more examples are highlighted in pop-up text boxes. It should be noted that this issue is purely an artifact of conventional methodology, not any mistake by a given researcher. So long as the reader is careful and remains cautious of this issue, then mistaken correlation and / or artificial age extension can be avoided.

### Radiometric dating

This analysis recalibrates nearly 200 radiometric dates (S2 Table), most of which are ^40^Ar / ^39^Ar dates that have been recalibrated to the standard and decay constant pairing of Kuiper et al. [16], and Min et al. [19]. It is not the intention here to provide a thorough review of all radiometric dating methods (see [37]); however, given the large number of ^40^Ar / ^39^Ar dates used here, and given discrepancies in past recalibrations, a cursory overview is given to the method. This text is also included (and expanded) in the chart itself (S1 Table).

#### U-Pb and K-Ar

Most radiometric dates reported for Upper Cretaceous units use either U-Pb, K-Ar, or ^40^Ar / ^39^Ar dating methods. U-Pb and K-Ar are primary dating methods, which directly determine the age of a sample and do not require recalibration (unless decay constants change, which is rare); whereas relative or secondary methods (such as ^40^Ar / ^39^Ar dating) require use of a monitor mineral of known or presumed age ("standard"). It is the recent changes to the recognized age of these standards that has been the cause of changing ^40^Ar / ^39^Ar dates.

U-Pb dating actually analyses two decay series (^235^U decay to ^207^Pb, and ^238^U decay to ^206^Pb), such that there are two independent measures of age, the overlap of which is the concordant age of the sample [37]. Recent improvements in analytical techniques (High-Resolution–Secondary Ion Mass Spectrometry: SHRIMP; and Chemical Abrasion Thermal Ionization Mass Spectrometry, CA TIMS) have brought greater precision and accuracy to U-Pb dating, and it remains one of the best methodologies currently available [37]. The decay constant for U-Pb analysis is well established [38], and known to better than 0.07% accuracy [37].

K-Ar dating is an older method of radiometric dating that was commonplace up until the end of the 1980's when it was essentially replaced by the more precise and accurate ^40^Ar / ^39^Ar method [37]. K-Ar had a range of benefits, including a large number of possible datable minerals (due to the common occurrence of potassium in many rock-forming minerals), but among its drawbacks was a relative lack of precision, largely due to the requirement to run two separate analyses per sample for K and ^40^Ar. As such, analytical precision was never better than 0.5%, and with the development of new technologies K-Ar dating was quickly replaced by ^40^Ar / ^39^Ar in the early 1990's [37]. Even so, some K-Ar dates are still the only dates available for a given unit, and so are included in the chart. K-Ar dates typically have error in the region of 1–2 m.y. for Upper Cretaceous units, so are useful indicators as to a general age range for a unit, but not for precise correlation.

#### 40Ar / 39Ar

Detailed reviews of ^40^Ar / ^39^Ar dating have been published elsewhere (e.g. [39, 40]). Notes given here are for the purpose of aiding the reader in understanding the recalculation of radiometric dates reported in this work, how ^40^Ar / ^39^Ar dates are affected by changing standards and decay constants, and comparability of radiometric dates recovered by different methods (e.g., ^40^Ar / ^39^Ar vs U-Pb).

#### 40Ar / 39Ar standards (neutron fluence monitor)

As ^40^Ar / ^39^Ar dating is a secondary dating method, every unknown sample needs to be analysed alongside a sample of known age: a standard. Primary standards are minerals from specific rock samples that have been directly dated by ^40^K / ^40^Ar dating or another method; whereas secondary standards are based on ^40^Ar / ^39^Ar intercalibration with a primary standard [41]. The following list includes (but is not limited to) some of the more popular standards that have been used historically (see McDougall and Harrison [39] for a more complete list):

**MMhb-1** McClure Mountain hornblende, primary standard: ~420 Ma
**GA-1550** Biotite, monazite, NSW, Australia, primary standard: ~98 Ma
**TCR** Taylor Creek Rhyolite (or sanidine, TCs), secondary standard: ~28 Ma
**FCT** Fish Canyon Tuff (or sandine, FCs), secondary standard: ~28 Ma
**ACR** Alder Creek Rhyolite (or sanidine, ACs), secondary or tertiary standard: ~1 Ma

Standards are chosen depending on availability, and should be of an age comparable to the unknown sample [41]. Hence, for Upper Cretaceous deposits, usually the secondary standards TCR or FCT have been used, typically themselves being calibrated against a primary standard (historically, the MMhb-1 is commonly used, although this depends on the preference of the particular laboratory). Many historically popular standards are no longer used, as repeated calibration studies have found the original sample to give inconsistent dates. For example, Baksi et al. [42] found the widely used MMhb-1 primary standard to be inhomogenous, making its use as a standard no longer tenable. Further, intercalibration studies have continually honed and refined the ages of standards (especially the more widely used secondary standards), with the result that radiometric dates published years apart are typically not precisely comparable without recalibration (e.g. [41, 43, 44]).

For ^40^Ar / ^39^Ar analysis, a significant issue concerns the changing age of the Fish Canyon Tuff (FCT: the relative standard used for most ^40^Ar / ^39^Ar analyses of Cretaceous rocks), and to a lesser extent, the associated decay constants (λβ: β- decay of 40K to 40Ca; and λε: electron capture or β+ of 40K to 40Ar; which combined are referred to as λT or λtotal [45].

Cebula et al. [46] first proposed an age of 27.79 Ma for the Fish Canyon Tuff. This was quickly refined to 27.84 Ma by Samson and Alexander [43], which remained the standard used by ^40^Ar / ^39^Ar analyses published up to the mid 1990’s (e.g., [47]). Renne et al. [44] revised the FCT to 27.95 Ma (although this new figure was not commonly used at the time). The next major update was that of Renne et al. [41], whereupon the FCT was revised to 28.02 Ma, which was widely accepted up to 2008 when Kuiper et al. [16] published the current standard of 28.201 Ma. This also brought ^40^Ar / ^39^Ar dates into line with U-Pb dates, unifying these two major chronostratigraphic dating systems [16]. Two further revisions have been offered by Renne et al. in 2010 [40] and 2011 [20], of 28.305 Ma, and 28.294 Ma (respectively). Rivera et al. [22], Meyers et al. [48], Singer et al. [49], and Sageman et al. [18] all found independent support for Kuiper et al.'s [16] 28.201 Ma age for the Fish Canyon Sanidine (and therefore rejected Renne et al.'s [40] further revised 28.305 Ma standard as too old). These analyses also used three methods (^40^Ar / ^39^Ar, U-Pb, cyclostratigraphy) to reach consensus, confirming alignment of U-Pb and ^40^Ar / ^39^Ar dates.

When applied to Upper Cretaceous units, a ~0.2 m.y. difference between the age of two different standards corresponds to ~0.4–0.5 m.y. difference in the ^40^Ar / ^39^Ar age of the analysed sample, and this is exacerbated if the standards used were further apart. For example, using the 27.84 Ma standard of Samson and Alexander [43], Rogers et al. [50] published an ^40^Ar / ^39^Ar date of 74.076 Ma for a bentonite at the top of the Two Medicine Formation, MT. This becomes 75.038 Ma if using the current Kuiper et al. [16] standard, and 75.271 Ma under the less commonly used Renne et al. [20] standard, a difference of 1.28 m.y. from the originally published date.

#### 40Ar / 39Ar decay constants

The ^40^Ar / ^39^Ar method depends upon the β- decay of ^40^K to ^40^Ca (λβ), and electron capture or β+ of ^40^K to ^40^Ar (λε), which combined are referred to as λT or λtotal [45]. The value of the decay constant λT (and its components) has historically been subject to fewer changes than the standards listed above, but has come under increased scrutiny since the late 1990's. The currently accepted standard is 5.463 E-10/y [19], although alternatives are available, and refinement of this figure is the subject of active research (see S1 Table).

The decay constant used for an analysis is not always reported, although it has much less effect on the final calculated age than variations in fluence monitor mineral ages. For example, the difference between using 5.543 E-10/y [38] and 5.463 E-10/y [19] is 0.02%, equating to a difference of 0.013 Ma for a sample from the late Campanian (~75 Ma). It should be noted that different values of λT have been used historically by geochronologists compared to physicists and chemists; this is pointed out by Renne et al., [41] who note that (for example) Endt [51] used a λT value of 5.428 +/- 0.032 E^-10^/y which "is more than 2% different from the values recommended by Steiger and Jaeger (1977) [38]". Thus, there is no guarantee that, unless otherwise stated, a lab that performed an ^40^Ar / ^39^Ar analysis in the 1990's will be using the λT of 5.543 E^-10^/y of Steiger and Jaeger [38], although all dates recalibrated here use either this, Min et al. [19], or Renne et al. [20]. Further details and a history of decay constant values can be found in the corresponding note within S1 Table.

#### 40Ar / 39Ar, recalibration & current standards

In order to compare ^40^Ar / ^39^Ar dates, it is essential to ensure that the same standards and decay constants were used in their calculation, which may require recalibration. If the standards used are different (for example, if an old analysis used the TCR standard, and a more recent one used the FCT), then it will be necessary to find what the equivalent FCT value was to the TCR used in the original analysis. This is usually achieved by referencing either the original publication of the standard, or the relevant published intercalibration analysis (e.g., [44]). It is critical to understand that recalculation cannot simply be performed by entering the original standard used (e.g., TCR = 28.32 Ma) into the equation provided on the recalculation sheet from McLean and EARTHTIME [21] (or the adapted spreadsheet used here); the equivalent FCT value is what must be entered, as the formula only uses FCT.

The decay constant absolute value has only a small effect on the absolute age of a sample, but decay constants contribute a greater amount to the error range of a radiometric date.

There are two current prominently used pairings of standard and decay constant. Kuiper et al. [16] combined an FCT standard age of 28.201 +/-0.023 Ma with the decay constant of Min et al. [19], λT = 5.463 +/- 0.214 E-10/y. Renne et al. [20] use an FCT standard age of 28.294 +/- 0.036 Ma, with a λT of 5.5305 E-10/y. The dates used here in the correlation chart (S1 Table) are calibrated to the Kuiper et al. [16] standard, paired with the Min et al. [19] decay constant. This is not a reflection on the reliability of one method over another; rather it is out of convenience, because the various ammonite biozones and magnetochrons detailed in The Geological Time Scale 2012 ([23]; upon which this chart is based) use the Kuiper et al. [16] FCT standard, and Min et al. [19] decay constant.

#### 40Ar / 39Ar, choice of mineral

Direct comparisons between ^40^Ar / ^39^Ar dates require not only the same standard and decay constant pairing, but also that the subject mineral is the same. Although it is theoretically possible that a date obtained from biotite crystals might be comparable with one from sanidine, in practice the difference in closure temperature (the temperature at which the mineral no longer loses any products of radioactive decay [37]) and other factors such as recoil effects [52] mean that (for example) biotite dates are typically ~0.3% older than sanidine dates (e.g., see [50]). The current "gold standard" mineral for ^40^Ar / ^39^Ar dating is sanidine, and most modern analyses use this mineral exclusively; however, plagioclase and biotite dates are quite common in literature from the 1990's. Here these non-sanidine dates are recalibrated, and they are comparable to each other (i.e., biotite dates can be directly compared with other biotite dates), but caution is advised when comparing non-sanidine dates with those of sanidine (although this is sometimes unavoidable).

#### 40Ar / 39Ar, reporting of uncertainty / error

Reporting of error associated with ^40^Ar / ^39^Ar derived ages is not standardized and varies in the inclusiveness of sources of error, the statistical method used to calculate error, the type of error, and in the amount of analytical information provided.

Sources of error in ^40^Ar / ^39^Ar analyses include analytical error (e.g., J-value), uncertainty in the standard used (e.g., age of the Fish Canyon Tuff, FCT is 28.201 +/- 0.23 Ma at 1σ [16]), uncertainty in the decay constant (e.g., λ_T_ of 5.463 +/- 0.214 E-10/y [19]), and geological processes that may lead to post-crystallization alteration of isotope ratios [37]. Most older publications do not explicitly state what is included in the reported error, but newer studies (e.g., [53]) report both analytical and systematic error.

The statistical method used to report error is not standardized, and is typically given in one of three forms; some authors report 1 or 2 standard deviations (σ); Standard Error is also commonly reported (especially for population means); finally, some authors report the 95% confidence interval for the population mean, which is roughly equivalent to 2σ (= 95.45% confidence interval).

It is common for published radiometric dates to lack associated details of the analysis, by either the date being given as a personal communication, or simply the omission of analytical details. Consequently, it is sometimes unclear as to whether (for example) a stated error of +/- 0.15 Ma refers to 1σ, 2σ, Standard Error, or whether it includes analytical and systematic error.

As such, it is not possible to make the error consistent between each recalibration (although the effects are relatively minor). Where possible, recalibrated error is reported to 1σ analytical error, but generally the original reported error is simply processed through the recalibration spreadsheet, noting wherever possible all details and any issues that may arise. Direct comparison of error between dates (both recalibrated and unrecalibrated) should therefore be approached with caution.

#### Agreement of 40Ar / 39Ar dates with U-Pb dates

^40^Ar / ^39^Ar dates have historically tended to be younger than U-Pb dates by about 1% [54], equating to ~750 k.y. difference in a 75 m.y. old sample (i.e., the approximate age of the units studied here). Possible explanations include longer zircon magma residence times prior to an eruption [37], error in the ^40^K decay constant [55], or interlaboratory bias and geological complexities [16]. Recent revisions of standards and decay constants for ^40^Ar / ^39^Ar dating have closed the gap to within ~0.3% [16, 20]. This led Kuiper et al. [16] to suggest that ^40^Ar / ^39^Ar dating has improved "absolute uncertainty from ~2.5% to 0.25%".

It should be noted [37, 41], that when comparing dates within the same system (i.e., ^40^Ar / ^39^Ar compared to ^40^Ar / ^39^Ar; and U-Pb dates compared to other U-Pb dates) then it is accepted practice to not include internal error (such as data uncertainties in K-Ar, decay constants, and intercalibration factors [41]) as both dates are subject to the same uncertainty, effectively canceling it out. However, when directly comparing dates derived from different systems (i.e., ^40^Ar / ^39^Ar dates with U-Pb dates), then internal error should be included. An example from Renne et al. [41] showed that when reported separately, and therefore without internal error, the age of a biotite-derived ^40^Ar / ^39^Ar date for the Permo-Triassic Siberian Trap basalt was 250.0 +/- 0.1 Ma, whereas a zircon and baddeleyite U-Pb date from the same intrusion was 251.2 +/- 0.2 Ma. When properly compared with the internal error included, the ^40^Ar / ^39^Ar dates became 250.0 +/- 2.3 Ma, whereas the U-Pb date was recalculated as 251.2 +/- 0.3 Ma, such that the error ranges of the dates now overlap. In the case of this current work, only three U-Pb dates are plotted in S1 Table, all of which are from the Javelina and Aguja formations of Texas. The reader should therefore take care when comparing these units directly with other units based on ^40^Ar / ^39^Ar geochronology.

#### Other general comments

The number of decimal places for reported dates and error are left in their original published form where possible.

In previous publications, a number of radiometric dates are reported as personal communication or featured only in abstracts. Such references typically lack any analytical data, so original standards (etc.) must be assumed based on the year in which the analysis was (likely) conducted, and any details of the typical standards used by the scientist and laboratory that carried out the analysis (if known; see individual notes for details of sleuthing).

## Results

The results of this study are presented as separate documents in the Supporting Information; the stratigraphic chart (S1 Table), and the recalibration sheet (S2 Table). These documents contain a large amount of information in the various pop-up notes, most of which is not repeated here as it is best viewed in stratigraphic context.

### Notes on recalibrations by other authors

#### Various analyses published by J. D. Obradovich

Many critical ^40^Ar / ^39^Ar dates have been published by J. D. Obradovich (United States Geological Survey, Colorado), not the least of which his 1993 work, "a Cretaceous time scale" [52] which presented over 30 ^40^Ar / ^39^Ar dates for many key horizons or ammonite biozones, establishing a robust framework for the Late Cretaceous of the U.S. Western Interior. As such, recalibration of Obradovich radiometric dates is of great importance, but requires special caution due to the particular methodology of Obradovich during the 1990's (and possibly early 2000's), which differs slightly from what might be expected. During this time, Obradovich typically used the TCR as the standard for his analyses, but the equivalent age of the FCT (required for recalibration) is not typical. Indication of this is noted by Hicks et al. ([56] p.43) who state:

“The TCR (Duffield & Dalrymple, 1990) [57] has been used exclusively since 1990 by one of us (Obradovich) with an assigned age of 28.32 Ma normalized to an age of 520.4 Ma for MMhb-1 (Samson & Alexander, 1987) [43]. This age differs from that of 27.92 Ma assigned by Sarna-Wojcicki and Pringle (1992) [58]. The choice of 28.32 Ma was entirely pragmatic because this monitor age provided the best comparison with ages delivered by Obradovich and Cobban (1975) [59]. In an intercalibration study […] Renne et al. (1998) [41] obtained ages of 28.34 Ma for TCR and 28.02 Ma for FCT when calibrated against GA1550 biotite as their primary standard with an age of 98.79 Ma. This value of 28.02 agrees quite well with […] 28.03 Ma obtained through calibration based on the astronomical time scale (Renne et al., 1994) [44]. On the basis of unpublished data, one of us (Obradovich) obtained an age of 28.03 Ma for the FCT […] of W. McIntosh (Geoscience Department, New Mexico Institute of Mining and Technology, Socorro, New Mexico), calibrated against an age of 28.32 Ma for TCR.”

However, Obradovich-published analyses from this time do not exclusively use the TCR at 28.32 Ma, as Izzett and Obradovich [60] state that they use FCT sanidine at 27.55 Ma, and TCR sanidine at 27.92 Ma, both relative to MMhb-1 at 513.9 Ma (in conjunction with λT = 5.543 E-10/y). They note that the 513.9 Ma age of MMhb-1 differs from the then standardized age of 520.4 Ma [43] as the former age was calibrated in the lab where their current samples were analysed [61, 62].

This creates a problem when recalibrating ^40^Ar / ^39^Ar ages that used TCR as the fluence monitor (standard). The "official" TCR age of 27.92 Ma has a corresponding FCT age of 27.84 Ma [41, 43]. However, since most analyses by Obradovich use TCR at 28.32 Ma, then the question remains as to what number to use for the equivalent FCT when performing recalibrations. Renne et al. [41] provide an intercalibration factor for FCT:TCR of 1:1.00112 +/- 0.0010, which simply calculated is FCT = 28.32 / 1.100112 = 28.006 Ma. This agrees well with an FCT equivalent of 28.03 Ma (as calculated by Obradovich; see above [56, 63]) and a value of 28.02 Ma of Renne et al. [41]. In *The Geological Time Scale 2012* [23], Schmitz [17] recalibrates a selection of dates from Obradovich [52], and Hicks et al. [64, 65] using a legacy FCT age of 28.00 Ma (not stated, but retrocalculated here). Sageman et al. ([18]; cited as Siewert et al., in press, by Schmitz [17]) recalibrate Obradovich's older dates using a legacy FCT age of 28.02 Ma (thereby agreeing with Renne et al. [41]).

In this analysis, when recalibrating an ^40^Ar / ^39^Ar date that was calculated by Obradovich using TCR = 28.32, I use an FCT value of 28.03, as this is the equivalent FCT explicitly stated by Obradovich [63]. This is a very close value to the FCT value of 28.02 in Renne et al. [41] (where the TCR equivalent is 28.34 +/- 0.16 Ma; 1σ, ignoring decay error) so confusion between the two should be avoided, although the difference between ages calculated using 28.03 or 28.02 Ma standards would correspond to only 0.02 to 0.04 m.y. for ages in the Late Cretaceous (100.5–66 Ma [24]).

#### Roberts et al. (2013)

Roberts et al. [6] present a table of recalibrated radiometric dates from a selection of important dinosaur-bearing formations of the North American Western Interior. Unfortunately, 11 out of 18 dates are incorrectly recalibrated, producing dates that are incorrect by up to a million years.

For recalibrated dates of the Judith River Formation (originally published by Goodwin and Deino in 1989 [66]), the study [6] utilizes an incorrect original (legacy) FCT standard of 28.02 Ma (i.e., from Renne et al., 1998 [41], published after the original 1989 analysis). For the recalibration to be correct, the legacy standard must be the value of FCT that was equivalent to the MMhb-1 at 420.4 Ma, which is FCT = 27.84 Ma ([43]; see Renne et al. [41]). This produces recalibrations for the Judith River Formation that are nearly half a million years different from the corrected recalibrations calculated in the current article. For example, the sample 84MG8-3-4 was originally published as 78.2 Ma [66]; Roberts et al. [6] recalibrate it as 78.71 Ma, whereas the recalibration offered in the current work (see S1 and S2 Tables) is 79.22 Ma.

The same error was made for recalibrations from the Bearpaw, Dinosaur Park, and Oldman formations as the Renne et al. 1998 [41] FCT date of 28.02 was also input as the legacy FCT for dates originally published by Eberth and Deino in 1992 [67] and Eberth and Hamblin in 1993 [68]; i.e. before the 1998 paper was published. The correct legacy standard to be used for these recalibrations is again FCT = 27.84 Ma ([43]; confirmed by Eberth, pers. comm., 2017; in prep.)

When recalibrating ^40^Ar / ^39^Ar dates for the Fruitland and Kirtland formations, New Mexico (originally published by Fassett and Steiner in 1997 [69]), incorrect values are input for the original (legacy) decay constant (λ) and standard [6]. First, the legacy λ used by Roberts et al. [6] is 4.962E-10/y, which was presumably copied from the bottom of the chart on p. 243 of Fassett and Steiner [69], where it is labeled as the value of λβ (ie. the probability of β- decay of 40K to 40Ca), and is printed below the value of λε (0.581 E-10/y; probability of electron capture or β+ of 40Kto 40Ar). In this case, the correct λ value to use for recalibration is 5.543 E-10/y [38], which is the total (λT) of λβ plus λε. Second, Roberts et al. [6] correctly state that the legacy standard used by Fassett and Steiner [69] for fluence monitoring was the TCR at 28.32 Ma; however, this number is then input directly into the recalibration formula with the new FCT standard (28.201; [16]). This is incorrect as recalculation must use the same standard mineral (e.g., FCT) for both legacy and recalibrated dates. For the recalculation to be correct, the legacy standard must therefore be the value of FCT that was equivalent to the TCR at 28.32 at the time of the 1997 analysis, which is either FCT = 27.84 Ma or ~28.03 (see S1 Table; above note on Obradovich), both of which produce recalibrated ages ~1 million years older than the dates presented by Roberts et al. [6]. The resultant misrecalibrated dates are actually younger than the original legacy dates, which should have been more difficult to overlook as the standards for ^40^Ar / ^39^Ar dating have been getting progressively older, so all recalibrations should produce older dates.

The recalibrated dates of Roberts et al. [6] were replicated (therefore confirmed) by rerunning the legacy values through the recalibration spreadsheet provided by the EARTHTIME institute [21].

Seven recalibrations were performed correctly; four from the Kaiparowits Formation, Utah, one from the Wahweap Formation, Utah, and two from the Two Medicine Formation, Montana. All other recalibrated dates are incorrect and should be discarded.

## Discussion

It is beyond the scope of this short work to summarize the implications of everything in the stratigraphic chart. Here, some paleontological effects of recalibration are discussed.

### North-south biogeography and intracontinental faunal endemism

It has been proposed that during the Campanian, the Western Interior of North America was divided into relatively small latitudinally arrayed faunal provinces, each with a unique fauna [8, 70]). This is based primarily on the description of new genera and species of dinosaur collected from the Kaiparowits Formation, Utah (e.g., [8, 71, 72]), and the perception that the Kaiparowits Formation was deposited contemporaneously with other dinosaur-bearing deposits (e.g., the Dinosaur Park Formation, Alberta; Fruitland and Kirtland formations, New Mexico). However, review of the data used in the original publication [8] and recalibrations performed here reduce support for this hypothesis.

In 2005, Roberts et al. [73] presented a thorough stratigraphic and sedimentological description of the Kaiparowits Formation, including three ^40^Ar / ^39^Ar dates (75.96 Ma; 75.02 Ma; and 74.21 Ma) from a series of volcanic ashes throughout the unit. This provided a welcome opportunity to more precisely correlate the Kaiparowits Formation with similarly aged units in the Western Interior, permitting the testing of paleontological hypotheses regarding the biogeography, phylogeny, and mode of evolution of their dinosaur fauna.

These themes were later explored by the hypothesis of 'intracontinental faunal endemism' [8, 70], which proposed that taxonomic differences among the dinosaurs of the Kaiparowits Formation, Dinosaur Park Formation (Alberta), Two Medicine Formation (Montana), and Fruitland and Kirtland formations (New Mexico) were representative of different species being endemic to small geographic ranges. Key evidence for this hypothesis was the presentation and discussion of the stratigraphic ranges of chasmosaurine ceratopsid dinosaurs, of which many taxa were shown to have overlapped [8]. This would mean that these taxa were contemporaneous, but apparently segregated geographically, thereby forming key support for intracontinental faunal endemism [8].

However, the chronostratigraphic data used to plot the stratigraphic ranges of chasmosaurine taxa [8] contained an unexplained inconsistency related to the mixed use of unrecalibrated and recalibrated ^40^Ar / ^39^Ar dates. The stratigraphic ranges of chasmosaurines from the Kaiparowits Formation (*Utahceratops* and *Kosmoceratops*) were plotted as occurring from 76.3 to 75.5 Ma, and regarding the duration of the formation itself, Sampson et al. ([8] p.6) state "Laser-fusion 40Ar/39Ar ages indicate a late Campanian range for the formation, spanning 76.6–74.5 Ma and corresponding to the Judithian land vertebrate age (Fig. 7)", and cite Roberts et al. [73] as the source for these ages. However, as shown above, the dates in Roberts et al. [73] range from 75.96 to 74.21 Ma, i.e. the youngest date given by Roberts et al. [73], 74.21 Ma, is younger than the upper age limit of the entire formation (74.5 Ma) given by Sampson et al. [8], which is clearly impossible. Furthermore, Roberts et al. ([73] p. 312) explicitly state that "utilizing an average rock accumulation rate of 41 cm/ka, the ca. 860-m-thick Kaiparowits Formation accumulated for ca. 2.1 Ma, from ca. 76.1–74.0 Ma". This is therefore inconsistent with the taxon and formational ranges of Sampson et al. ([8]; 76.6–74.5 Ma), and at the time of publication the origin of these dates remained unexplained

More information was provided the following year in a generalized stratigraphic column of the Kaiparowits Formation [74], which presented four ^40^Ar / ^39^Ar dates (76.46 Ma; 75.97 Ma; 75.51 Ma; and again 75.51 Ma), three of which corresponded stratigraphically with the same horizons dated by Roberts et al. in 2005 [73], but with different numerical ages. The 2011 study [74] does not state that these are recalibrated dates, and instead cites Roberts 2007 [75] as the source for three of these dates, but the dates in Roberts [75] are the same as in Roberts et al. [73], and do not correspond with the numbers given in Zanno et al. [74]. It is notable that the dates in Zanno et al. [74] are consistent with the age range given by Sampson et al. [8], i.e. that they probably had the same, still unexplained source.

The source of the new dates was only officially published in 2013, when Roberts et al. [6] published a series of dates from the Kaiparowits Formation that were recalibrated (using the FCT standard and decay constant pairing of Kuiper et al. [16] 28.201 Ma; and Min et al. [19]) from those published by Roberts et al. in 2005 [73]; which used the 28.02 Ma age for the FCT standard; Renne et al. [41]). That the 2010 Sampson et al. [8] Kaiparowits age is indeed based on recalibrated dates is effectively confirmed by a statement in Roberts et al. ([6] p.85) which states, "recalibration of Kaiparowits Formation ash beds demonstrates that the formation is approximately half a million years older than previously suggested, deposited ~76.6–74.5 Ma.", i.e., exactly the same age duration as given by Sampson et al. [8].

This demonstrates unequivocally that the initial formulation of the endemism hypothesis [8] used a mixture of ^40^Ar / ^39^Ar dates calibrated to different standards to plot the stratigraphic occurrence of chasmosaurine taxa, mistakenly resulting in the overlapping of certain taxa. *Utahceratops* and *Kosmoceratops* from the Kaiparowits Formation were the only taxa that were plotted based on radiometric dates recalibrated to the current standard [16]. Other taxa from different units (Dinosaur Park Formation, Alberta; Fruitland and Kirtland formations, New Mexico) were plotted based on unrecalibrated dates which used previous standards, mostly that of Samson and Alexander [43]. This results in taxa from the Kaiparowits Formation being shown ~0.5 m.y. relatively older [6] than they would have been if they had been plotted to the same standard as the taxa from the other units.

When all the available dates are recalibrated to the same standards (as in the current work), the stratigraphic overlap between key taxa is no longer recovered. Only the lower part of the Kaiparowits Formation stratigraphically overlaps with the fossiliferous portion of the Dinosaur Park Formation (see S1 Table). This is important as the lower Kaiparowits Formation does not yield the taxa purportedly endemic to southern Utah, and fragmentary specimens suggest that taxa are shared between the upper part of the Dinosaur Park and lower Kaiparowits formations [76]. Here it is considered more likely that differences between dinosaur species found in the Dinosaur Park Formation and middle Kaiparowits Formation are mostly an artifact of sampling different stratigraphic levels, rather than biogeographic segregation (also see [77]). Similarly, differences between the middle Kaiparowits taxa and those of the Fruitland and Kirtland formations, New Mexico, are also more parsimoniously explained by the slight difference in age of the units, with the Fruitland and Kirtland formations being slightly younger than the middle Kaiparowits Formation [4, 77]. Moreover, the recent identification of purportedly southern *Pentaceratops*-lineage chasmosaurines within the Dinosaur Park Formation, Alberta [31, 78], demonstrates that this lineage was able to move between northern and southern regions in the middle Campanian.

### Biostratigraphy

Cobban and Reeside [1] used the ceratopsid dinosaur *Triceratops* as an index taxon of the latest Maastrichtian. Similarly, dinosaurs were part of the original Land Vertebrate Ages (LVA; Aquilian; Judithian; Edmontonian; Lancian) described by Russell [79] before revision into North American Land Mammal Ages (NALMA; [80–82]). More recently, dinosaurs have been used to stratigraphically correlate Campanian and Maastrichtian units of the United States [29, 83–85], and were utilized by Sullivan and Lucas [3, 4] in their definition of the “Kirtlandian”: an additional LVA roughly equivalent to the early deposition of the Bearpaw Shale and positioned in the gap between the Judithian and Edmontonian identified by Russell [79, 80]. Dinosaurs were also strongly utilised for biostratigraphy in the definition or redefinition of 10 vertebrate biochrons for the Cretaceous of the Western Interior [30].

The demonstration that individual dinosaur species form stratigraphically stacked sequences of non-overlapping taxa could make them useful for biostratigraphy. This might be seen as controversial, since generally dinosaur taxa are known from relatively few specimens and are arguably less abundant than mammals or other groups typically used in terrestrial biostratigraphy. However, at least some clades of dinosaurs would seem ideal for biostratigraphic correlation, especially if current hypotheses of rapid evolution are correct (e.g. [11, 12, 31, 86]). For example, the chasmosaurine dinosaur *Triceratops* has been demonstrated to evolve at least three different metaspecies through the duration of the Hell Creek Formation in Montana [12]. Although the duration of the Hell Creek Formation is not precisely known, two stratigraphically separated metaspecies of *Triceratops* (*T*. *prorsus* and *T*. sp. [12]) are recorded from the uppermost 30 m, which has been recently demonstrated by Ar / Ar dates as representing ~300 k.y. of deposition ([53]; see S1 Table). If we are able to understand the stratigraphic distribution and ontogenetic changes of dinosaurs well enough, then conceivably many more clades may be biostratigraphically informative at resolutions of ~300Ka (or less; see S1 Table).

## Conclusions

Understanding the paleobiology of extinct organisms requires explicit knowledge of their relative positions in time. In turn, this depends upon the accurate correlation of the geological formations from which fossil remains are recovered.

Here, recalibrated radiometric dates are combined with existing stratigraphic data to create a comprehensive stratigraphic correlation chart for terrestrial units of the U.S Western Interior. This revised stratigraphic framework is intended to be a tool for use by other researchers to investigate dinosaur evolution. Recalibration of radiometric dates to the same standard should remove artifacts of miscorrelation, permitting a clearer search for evolutionary patterns. Conflicts between different kinds of stratigraphic data are highlighted, particularly where they may affect paleontological understanding. Future expansions of the chart will increase the geographic scope of formations covered, and include additional taxa.

## Supporting information

S1 Table Stratigraphic chart. Stratigraphic correlation of Upper Cretaceous terrestrial strata of the North American Western Interior from the Santonian through to the K-Pg boundary. Dinosaur taxon ranges plotted on to correlated geological units.
(XLS)

S2 Table Recalibration sheet. This sheet shows recalibration calculations for over 200 published Ar / Ar radiometric dates. These are recalibrated to the two current standards (Kuiper et al., 2008; Renne et al., 2011), shown on separate tabs. References are given within pop up notes for the respective recalibrated date(s).
(XLS)

S1 Text References for stratigraphic chart. This text file lists all the references used in construction of the stratigraphic chart (S1 Table).
(DOC)

S2 Text Comment boxes for stratigraphic chart. This text file provides transcripts of all the pop-up comment boxes featured in the stratigraphic chart (S1 Table). This file should be of use to readers who prefer the text in this larger format.
(DOC)

S1 Fig Stratigraphic chart, graphic version. This is a image file version of the stratigraphic chart (S1 Table). It is an image only, provided for quick reference, and does not have embedded pop-up comments.
(JPG)

S2 Fig Geographic location of stratigraphic sections featured on stratigraphic chart S1 Table. This map shows the geographic location of the different stratigraphic sections shown in stratigraphic chart S1 Table.
(JPG)

## Abbreviations

c.z.: coal zone
FCT: Fish Canyon Tuff
Fm: Formation
Gp: Group
k.y.: thousand years
Ka: thousands of years ago
m.y.: million years
Ma: millions of years ago
Mbr: Member
TCR: Taylor Creek Rhyolite
