# Supplementary material for: Revised geochronology, correlation, and dinosaur stratigraphic ranges of the Santonian-Maastrichtian (Late Cretaceous) formations of the Western Interior of North America
Source: PLoS One. 2017 Nov 22;12(11):e0188426. doi: 10.1371/journal.pone.0188426 (PMC5699823; doi:10.1371/journal.pone.0188426)
Supplement: S1 Text — This text file lists all the references used in construction of the stratigraphic chart (S1 Table). (DOC) [file pone.0188426.s003.doc]

# S1 Text. References for Stratigraphic Chart.

This text file lists all the references used in construction of the stratigraphic chart (S1 Table) for:

Fowler (xxxx) Cretaceous terrestrial stratigraphy and dinosaur biostratigraphy of Santonian-Maastrichtian formations of the Western Interior of North America.

--

1.

Albright LB, Titus AL. Magnetostratigraphy of Upper Cretaceous strata in Grand Staircase-Escalante National Monument, southern Utah: The Santonian–Campanian Stage boundary, reassessment of the C33N/C33R magnetochron boundary, and implications for regional sedimentation patterns within the Sevier Foreland Basin. Cretaceous Research. 2016;63: 77–94.

2.

Alexander EC, Mickelson GM, Lanphere MA. Mmhb-1: a new 40Ar/39Ar dating standard. Short papers of the fourth international conference, geochronology, cosmochronology, and isotope geology: US Geological Survey Open-File Report. 1978. pp. 78–701.

3.

Arbour VM, Burns ME, Sissons RL. A redescription of the ankylosaurid dinosaur *Dyoplosaurus acutosquameus* Parks, 1924 (Ornithischia: Ankylosauria) and a revision of the genus. Journal of Vertebrate Paleontology. 2009;29: 1117–1135. doi:10.1671/039.029.0405

4.

Archibald JD, Butler RF, Lindsay EH, Clemens WA, Dingus L. Upper Cretaceous–Paleocene biostratigraphy and magnetostratigraphy, Hell Creek and Tullock Formations, northeastern Montana. Geology. 1982;10: 153–159.

5.

Atchley SC, Nordt LC, Dworkin SI. Eustatic control on alluvial sequence stratigraphy: a possible example from the Cretaceous-Tertiary transition of the Tornillo Basin, Big Bend National Park, west Texas, U.S.A. Journal of Sedimentary Research. 2004;74: 391–404. doi:10.1306/102203740391

6.

Baadsgaard H, Lerbekmo JF, Wijbrans JR, Swisher III CC, Fanning M. Multimethod radiometric age for a bentonite near the top of the *Baculites reesidei* Zone of southwestern Saskatchewan (Campanian–Maastrichtian stage boundary?). Can J Earth Sci. 1993;30: 769–775. doi:10.1139/e93-063

7.

Baird D. The dome-headed dinosaur *Tylosteus ornatus* Leidy 1872 (Reptilia; Ornithischia; Pachycephalosauridae). Notulae Naturae. 1979;456: 1–11.

8.

Bakker RT, Sullivan RM, Porter V, Larson P, Saulsbury SJ. *Dracorex hogwartsia*, n. gen., n. sp., a spiked, flat-headed pachycephalosaurid dinosaur from the Upper Cretaceous Hell Creek Formation of South Dakota. New Mexico Museum of Natural History and Science Bulletin. 2006;35: 331–345.

9.

Baksi AK, Archibald DA, Farrar E. Intercalibration of 40Ar39Ar dating standards. Chemical Geology. 1996;129: 307–324.

10.

Baszio S. Oldest occurrence of the genus *Stegoceras* (Dinosauria: Pachycephalosauridae) from the Foremost Formation (Campanian) of South Alberta, Canada. Paläontologische Zeitschrift. 1997;71: 129–133.

11.

Bauer CM. Contributions to the geology and paleontology of San Juan County, New Mexico: Part 1. stratigraphy of a part of the Chaco River Valley. US Geological Survey Professional Paper. 1916; 271–278.

12.

Bauer CM, Reeside JB. Contributions to economic geology, 1920, Part II, Mineral fuels - Coal in the middle and eastern parts of San Juan County, New Mexico. US Geological Survey Bulletin. 1921;716–G: 155–237.

13.

Beaumont EC, Hoffman GK. Interrelationships between the upper coal member of the Menefee Formation, the La Ventana Tongue, and the Lewis Shale in the southeastern San Juan Basin, New Mexico. New Mexico: New Mexico Geological Society 43rd Guidebook. 1992; 207–216.

14.

Beckinsale RD, Gale NH. A reappraisal of the decay constants and branching ratio of 40K. Earth and Planetary Science Letters. 1969;6: 289–294.

15.

Befus KS, Hanson RE, Lehman TM, Griffin WR. Cretaceous basaltic phreatomagmatic volcanism in west Texas: maar complex at Pena Mountain, Big Bend National Park. Journal of Volcanology and Geothermal Research. 2008;173: 245–264.

16.

Behringer DH. Geometry, genesis and sequence stratigraphic framework of the Colgate Sandstone Member of the Fox Hills, Garfield County, MT. M.S., Montana State University. 2008.

17.

Bell PR. Cranial osteology and ontogeny of *Saurolophus angustirostris* from the Late Cretaceous of Mongolia with comments on *Saurolophus* osborni from Canada. Acta Palaeontologica Polonica. 2011;56: 703–722. doi:10.4202/app.2010.0061

18.

Benammi M, Ramos CM, Garcia EC. Preliminary Magnetostratigraphic study of the Upper Cretaceous dinosaur site from La Barranca Los Bonites, Tiquicheo (Michoacan State, southern Mexico). Geophysical Research Abstracts. 2006. p. 10656.

19.

Bouligand C, Dyment J, Gallet Y, Hulot G. Geomagnetic field variations between chrons 33r and 19r (83–41 Ma) from sea-surface magnetic anomaly profiles. Earth and Planetary Science Letters. 2006;250: 541–560. doi:10.1016/j.epsl.2006.06.051

20.

Braman DR. Terrestrial palynomorphs of the upper Santonian-? lowest Campanian Milk River Formation, Southern Alberta, Canada. Palynology. 2001;25: 57–107.

21.

Braman DR, Sweet AR, Lerbekmo JF. Upper Cretaceous - lower Tertiary lithostratigraphic relationships of three cores from Alberta, Saskatchewan, and Manitoba, Canada. Can J Earth Sci. 1999;36: 669–683. doi:10.1139/e97-108

22.

Braman DR, Sweet AR. Terrestrial palynomorph biostratigraphy of the Cypress Hills, Wood Mountain, and Turtle Mountain areas (Upper Cretaceous-Paleocene) of western Canada. Canadian Journal of Earth Sciences. 1999;36: 725–741.

23.

Braman DR, Sweet AR. Biostratigraphically useful Late Cretaceous–Paleocene terrestrial palynomorphs from the Canadian Western Interior sedimentary basin. Palynology. 2012;36: 8–35.

24.

Breyer JA, Busbey III AB, Hanson RE, Befus KE, Griffin WR, Hargrove US, et al. Evidence for Late Cretaceous volcanism in Trans‐Pecos Texas. The Journal of Geology. 2007;115: 243–251. doi:10.1086/510640

25.

Brink KS, Zelenitsky DK, Evans DC, Horner JR, Therrien F. Cranial morphology and variation in *Hypacrosaurus stebingeri*. Hadrosaurs. Bloomington, Indiana: Indiana University Press; 2014. pp. 245–266.

26.

Brinkman DB. A review of nonmarine turtles from the Late Cretaceous of Alberta. Canadian Journal of Earth Sciences. 2003;40: 557–571.

27.

Brookins DG, Rigby JK. Geochronologic and geochemical study of volcanic ashes from the Kirtland Shale (Cretaceous), San Juan Basin, New Mexico. Geological Society of America Special Papers. 1987;209: 105–110. doi:10.1130/SPE209-p105

28.

Brown B, Schlaikjer EM. A study of the troodont dinosaurs with the description of a new genus and four new species. Bulletin of the American Museum of Natural History. 1943;82: 121–149.

29.

Brown B, Schlaikjer EM. The skeleton of *Leptoceratops* with the description of a new species. American Museum Novitates. 1942;1169: 1–15.

30.

Brown B. The Hell Creek beds of the Upper Cretaceous of Montana : their relation to contiguous deposits, with faunal and floral lists, and a discussion of their correlation. Bulletin of the American Museum of Natural History. 1907; 823–845.

31.

Brown B. The Cretaceous Ojo Alamo Beds of New Mexico: with description of the new dinosaur genus *Kritosaurus*. Bulletin of the American Museum of Natural History. 1910; 267–274.

32.

Brown B. A new trachodont dinosaur, *Hypacrosaurus*, from the Edmonton Cretaceous of Alberta. Bulletin of the American Museum of Natural History. 1913;32: 395–406.

33.

Brown B. The skeleton of *Saurolophus*: a crested duck-billed dinosaur from the Edmonton Cretaceous. Bulletin of the American Museum of Natural History. 1913;32: 387–393.

34.

Brown B. *Anchiceratops*, a new genus of horned dinosaur from the Edmonton Cretaceous of Alberta. With discussion of the origin of the ceratopsian crest and the brain casts of *Anchiceratops* and *Trachodon*. Bulletin of the American Museum of Natural History. 1914;33: 539–548.

35.

Brown B. *Corythosaurus casuarius*, a new crested dinosaur from the Belly River Cretaceous: with provisional classification of the Family Trachodontidae. Bulletin of the American Museum of Natural History. 1914;33: 539–548.

36.

Brown B. *Leptoceratops*, a new genus of Ceratopsia from the Edmonton Cretaceous of Alberta. Bulletin of the American Museum of Natural History. 1914;33: 567–580.

37.

Brown B. A new crested trachodont dinosaur *Prosaurolophus maximus*. Bulletin of the American Museum of Natural History. 1916;35: 701–708.

38.

Brown CM, Henderson DM. A new horned dinosaur reveals convergent evolution in cranial ornamentation in Ceratopsidae. Current Biology. 2015;25: 1641–1648. doi:10.1016/j.cub.2015.04.041

39.

Brownfield ME, Johnson EA. The Yampa Bed-a regionally extensive tonstein in the Williams Fork Formation, northwestern Piceance Creek and southern Sand Wash Basins, Colorado. USGS Scientific Investigations Report. 2008;5033: 1–32.

40.

Butler RF, Lindsay EH. Mineralogy of magnetic minerals and revised magnetic polarity stratigraphy of continental sediments, San Juan Basin, New Mexico. The Journal of Geology. 1985;93: 535–554.

41.

Calvert WR, Bowen CF, Herald FA, Hance JH, Stebinger E, Beekly AL. Geology of certain lignite fields in eastern Montana. US Geological Survey Bulletin. 1912;471: 187–201.

42.

Campbell JA, Ryan MJ, Holmes RB, Schröder-Adams CJ. A re-evaluation of the chasmosaurine ceratopsid genus *Chasmosaurus* (Dinosauria: Ornithischia) from the Upper Cretaceous (Campanian) Dinosaur Park Formation of western Canada. PLoS ONE. 2016;11: e0145805. doi:10.1371/journal.pone.0145805

43.

Campione NE, Evans DC. Cranial growth and variation in edmontosaurs (Dinosauria: Hadrosauridae): implications for Latest Cretaceous megaherbivore diversity in North America. PLoS ONE. 2011;6: e25186. doi:10.1371/journal.pone.0025186

44.

Cantrell AK, Suazo TL, Lucas SG, Sullivan RM. Preliminary report of a nearly complete juvenile *Pentaceratops* from the Upper Cretaceous Kirtland Formation (Hunter Wash Member), San Juan Basin, New Mexico. New Mexico Geological Society. 2013. p. 17.

45.

Catuneanu O, Sweet AR. Maastrichtian-Paleocene foreland-basin stratigraphies, western Canada: a reciprocal sequence architecture. Canadian Journal of Earth Sciences. 1999;36: 685–703.

46.

Cebula GT, Kunk MJ, Mehnert HH, Naeser CW, Obradovich JD, Sutter JF. The Fish Canyon Tuff, a potential standard for the 40Ar-39Ar and fission-track dating methods. Terra Cognita. 1986;6: 139–140.

47.

Chapman RE, Galton PM, Sepkoski Jr JJ, Wall WP. A morphometric study of the cranium of the pachycephalosaurid dinosaur *Stegoceras*. Journal of Paleontology. 1981; 608–618.

48.

Chen D, Langenberg CW, Beaton AP. Horseshoe Canyon-Bearpaw transition and correlation of associated coal zones across the Alberta plains. Edmonton, Alberta: Alberta Energy and Utilities Board : Alberta Geological Survey; 2005.

49.

Chinnery B. Description of *Prenoceratops pieganensis* gen. et sp. nov. (Dinosauria: Neoceratopsia) from the Two Medicine Formation of Montana. Journal of Vertebrate Paleontology. 2004;24: 572–590. doi:10.1671/0272-4634(2004)024[0572:DOPPGE]2.0.CO;2

50.

Chinnery BJ, Horner JR. A new neoceratopsian dinosaur linking North American and Asian taxa. Journal of Vertebrate Paleontology. 2007;27: 625–641. doi:10.1671/0272-4634(2007)27[625:ANNDLN]2.0.CO;2

51.

Chinnery BJ, Weishampel DB. *Montanoceratops cerorhynchus* (Dinosauria: Ceratopsia) and relationships among basal neoceratopsians. Journal of Vertebrate Paleontology. 1998;18: 569–585. doi:10.1080/02724634.1998.10011085

52.

Christensen AE. Sequence Stratigraphy, Sedimentology and Provenance of the Drip Tank Member, Straight Cliffs Formation, Kaiparowits Plateau, Southwestern Utah. Geological Society of America Abstracts with Programs. 2005. p. 115. Available: https://gsa.confex.com/gsa/2005AM/finalprogram/abstract_91942.htm

53.

Christensen WK. A review of the Upper Campanian and Maastrichtian belemnite biostratigraphy of Europe. Cretaceous Research. 1996;17: 751–766. doi:10.1006/cres.1996.0040

54.

Cifelli RL, Eberle JJ, Lofgren DL, Lillegraven JA, Clemens WA. Mammalian biochronology of the latest Cretaceous. In: Woodburne MO, editor. Late Cretaceous and Cenozoic Mammals of North America. New York: Columbia University Press; 2004. pp. 21–42.

55.

Clemens WA. Continental vertebrates from the Late Cretaceous of the North Slope, Alaska. 1992 Proceedings International Conference on Arctic Margins: Outer Continental Shelf Study, Mineral Management Service. 1994. pp. 94–0040.

56.

Cobban WA. Telegraph Creek Formation of Sweetgrass Arch, north-central Montana. American Association of Petroleum Geologists Bulletin. 1950;34: 1899–1900.

57.

Cobban WA. Cretaceous Rocks of Northwestern Montana. Billings Geological Society: Guidebook: Sixth Annual Field Conference. 1955; 107–119.

58.

Cobban WA. Diversity and distribution of Late Cretaceous ammonites, Western Interior, United States. Evolution of the Western Interior Basin: Geological Association of Canada Special Paper. 1993;39: 435–451.

59.

Cobban WA, Reeside JB. Correlation of the Cretaceous formations of the Western Interior of the United States. Geological Society of America Bulletin. 1952;63: 1011–1044.

60.

Cobban WA, Walaszczyk I, Obradovich JD, McKinney KC. USGS zonal table for the Upper Cretaceous middle Cenomanian-Maastrichtian of the Western Interior of the United States based on ammonites, inoceramids, and radiometric ages. United States Geological Survey. 2006; Available: http://govreports.library.gatech.edu/handle/123456789/9033

61.

Cobban WA. The Late Cretaceous ammonites *Scaphites leei* Reeside and *Scaphites hippocrepis* (DeKay) in the Western Interior of the United States. United States Geological Survey, Professional Paper. 1969;619: 1–29.

62.

Cochran JK, Landman NH, Turekian KK, Michard A, Schrag DP. Paleoceanography of the Late Cretaceous (Maastrichtian) Western Interior Seaway of North America: evidence from Sr and O isotopes. Palaeogeography, Palaeoclimatology, Palaeoecology. 2003;191: 45–64.

63.

Collier AJ, Knechtel MM. Coal resources of McCone County, Montana. US Geological Survey Bulletin. 1939;905: 1–80.

64.

Conrad JE, Mackee EH, Turrin BD. Age of tephra beds at the Ocean Point dinosaur locality, North Slope, Alaska, based on K-Ar and 40Ar/39Ar analyses. US Geological Survey Bulletin. 1992;1990–C: 1–12.

65.

Cross AT, Yi MS. Palynology and a review of vertebrate faunas of the Late Cretaceous-Paleocene North Horn Formation, Price Canyon, Wasatch Plateau and environs, Utah, USA. Dinofest International, Academy of Natural Sciences, Philadelphia. 1997; 417–455.

66.

Cullins HL. Geologic map of the Rangely quadrangle, Rio Blanco County, Colorado [Internet]. Denver, Colorado: United States Geological Survey; 1971 p. 1. Available: http://pubs.er.usgs.gov/publication/gq903

67.

Currie PJ, Langston Jr W, Tanke DH. A new species of *Pachyrhinosaurus* (Dinosauria, Ceratopsidae) from the Upper Cretaceous of Alberta, Canada. In: Currie PJ, Langston Jr W, editors. A New Horned Dinosaur from an Upper Cretaceous Bone Bed in Alberta. Ottawa, Ontario, Canada: NRC Research Press; 2008. pp. 1–108.

68.

Currie PJ, Russell DA. The geographic and stratigraphic distribution of articulated and associated dinosaur remains. In: Currie PJ, Koppelhus EB, editors. Dinosaur Provincial Park. Bloomington, Indiana: Indiana University Press; 2005. pp. 537–569.

69.

Dalrymple GB, Duffield WA. High precision 40Ar/39Ar dating of Oligocene rhyolites from the Mogollon-Datil volcanic field using a continuous laser system. Geophys Res Lett. 1988;15: 463–466. doi:10.1029/GL015i005p00463

70.

Davies JHFL, Wotzlaw J-F, Wolfe AP, Heaman LM. Assessing the age of the Late Cretaceous Danek bonebed with U–Pb geochronology. Can J Earth Sci. 2014;51: 982–986. doi:10.1139/cjes-2014-0136

71.

Davies KL. Hadrosaurian dinosaurs of Big Bend National Park, Brewster County, Texas. M.A. thesis, The University of Texas at Austin. 1983.

72.

Dawson FM, Evans CG, Marsh R, Richardson R. Uppermost Cretaceous and Tertiary strata of the Western Canada sedimentary basin. Geological atlas of the Western Canadian Sedimentary Basin G Mossop and I Shetsen (comps) Canadian Society of Petroleum Geologists and Alberta Research Council. 1994; 387–406.

73.

Dawson FM, Kalkreuth WD, Sweet AR. Stratigraphy and coal resource potential of the Upper Cretaceous to Tertiary strata of northwestern Alberta. Ottawa, Ontario, Canada: Geological Survey of Canada; 1994.

74.

Decker PL. Brookian sequence stratigraphic correlations, Umiat field to Milne Point field, west-central North Slope, Alaska. Fairbanks, Alaska: Alaska Department of Natural Resources, Division of Geological & Geophysical Surveys; 2007.

75.

Deibert JE, Breithaupt BH. Ferdinand v. Hayden’s 1868 “huge bird” tracks in the upper Cretaceous Almond Formation: field evidence for the first dinosaur fossil discovered in Wyoming. Late Cretaceous Vertebrates from the Western Interior: Bulletin 35. 2006;35: 69.

76.

Diem S, Archibald JD. Range extension of southern chasmosaurine ceratopsian dinosaurs into northwestern Colorado. Journal of Paleontology. 2005;79: 251–258. doi:10.1666/0022-3360(2005)0792.0.CO;2

77.

Difley R, Ekdale AA. Biostratigraphic aspects of the Cretaceous-Tertiary (KT) boundary interval at North Horn Mountain, Emery County, Utah. Vertebrate Paleontology in Utah. Salt Lake City: Utah Geological Survey; 1999. pp. 389–398. Available: https://books.google.com/books?hl=en&lr=&id=qeRM16ndBx4C&oi=fnd&pg=PA389&dq=+Difley+Ekdale+1999&ots=W4Hm36wfZC&sig=6X4lvaWFXuJ8Bh753loEjrpunCY

78.

Difley R. Biostratigraphy of the North Horn Formation at North Horn Mountain, Emery County, Utah. Central Utah: Diverse Geology of a Dynamic Landscape. 2007; 439–454.

79.

Difley RL, Ekdale AA. Footprints of Utah’s last dinosaurs: track beds in the Upper Cretaceous (Maastrichtian) North Horn Formation of the Wasatch Plateau, Central Utah. PALAIOS. 2002;17: 327–346. doi:10.1669/0883-1351(2002)017<0327:FOUSLD>2.0.CO;2

80.

Dodson P, Currie PJ. Neoceratopsia. The Dinosauria. Berkeley: University of California Press; 1990. pp. 593–619. Available: http://doc.rero.ch/record/15429

81.

Dodson P. *Avaceratops lammersi*: a new ceratopsid from the Judith River Formation of Montana. Proceedings of the Academy of Natural Sciences of Philadelphia. 1986;138: 305–317.

82.

Doelling HH. Interim geologic map of the Smoky Mountain 30’x 60’ Quadrangle. Utah Geological Survey Open-File Report 359. 1997;

83.

Duffield WA, Dalrymple GB. The Taylor Creek Rhyolite of New Mexico: a rapidly emplaced field of lava domes and flows. Bull Volcanol. 1990;52: 475–487. doi:10.1007/BF00268927

84.

Eaton JG, Cifelli RL. Preliminary report on Late Cretaceous mammals of the Kaiparowits Plateau, southern Utah. Rocky Mountain Geology. 1988;26: 45–55.

85.

Eaton JG, Diem S, Archibald JD, Schierup C, Munk H. Vertebrate paleontology of the Upper Cretaceous rocks of the Markagunt Plateau, southwestern Utah. Vertebrate paleontology in Utah. 1999;99: 323–334.

86.

Eaton JG, Laurin J, Kirkland JI, Tibert NE, Leckie RM, Sageman BB, et al. Cretaceous and Early Tertiary Geology of Cedar and Parowan Canyons, Western Markagunt Plateau, Utah Utah Geological Association Field Trip Road Log September, 2001. The Geologic Transition, High Plateaus to Great Basin - A Symposium and Field Guide. Salt Lake City: Utah Geological Association; 2001. pp. 337–363. Available: http://archives.datapages.com/data/pacific/data/102/102001/337_ps1020337.htm

87.

Eaton JG. Biostratigraphic framework for the Upper Cretaceous rocks of the Kaiparowits Plateau, southern Utah. Geological Society of America Special Papers. 1991;260: 47–64. doi:10.1130/SPE260-p47

88.

Eaton JG. Multituberculate mammals from the Wahweap (Campanian, Aquilan) and Kaiparowits (Campanian, Judithian) formations, within and near Grand Staircase-Escalante National Monument, southern Utah. Salt Lake City: Utah Geological Survey; 2002.

89.

Eaton JG. Santonian (late Cretaceous) mammals from the John Henry Member of the Straight Cliffs Formation, Grand Staircase-Escalante National Monument, Utah. Journal of Vertebrate Paleontology. 2006;26: 446–460. doi:10.1671/0272-4634(2006)26[446:SLCMFT]2.0.CO;2

90.

Eberth DA, Deino A. New 40Ar/39Ar ages from three bentonites in the Bearpaw, Horseshoe Canyon, and Scollard formations (Upper Cretaceous-Paleocene) of southern Alberta, Canada. Dinosaur Park Symposium: Short papers, abstracts, and program Royal Tyrrell Museum of Paleontology, Drumheller, Alberta. 2005. pp. 23–24.

91.

Eberth DA, Deino AL. A geochronology of the non-marine Judith River Formation of southern Alberta. SEPM Theme Meeting, Mesozoic of the Western Interior. 1992. pp. 24–25.

92.

Eberth DA, Thomas RG, Deino A. Preliminary K-Ar dates from bentonites in the Judith River and Bearpaw Formations (Upper Cretaceous) of Dinosaur Provincial park, southern Alberta, Canada. Aspects of nonmarine Cretaceous geology China Ocean Press, Beijing. 1992; 296–304.

93.

Eberth DA, Bell PR. Stratigraphy of the Danek Bonebed (Upper Cretaceous Horseshoe Canyon Formation, central Alberta) and correlations with strata in the Drumheller and Grande Prairie regions. Can J Earth Sci. 2014;51: 975–981. doi:10.1139/cjes-2014-0069

94.

Eberth DA, Braman DR. A revised stratigraphy and depositional history for the Horseshoe Canyon Formation (Upper Cretaceous), southern Alberta plains. Can J Earth Sci. 2012;49: 1053–1086. doi:10.1139/e2012-035

95.

Eberth DA, Brinkman DB, Chen P-J, Yuan F-T, Wu S-Z, Li G, et al. Sequence stratigraphy, paleoclimate patterns, and vertebrate fossil preservation in Jurassic/Cretaceous strata of the Junggar Basin, Xinjiang Autonomous Region, People’s Republic of China. Canadian Journal of Earth Sciences. 2001;38: 1627–1644. doi:10.1139/e01-067

96.

Eberth DA, de Jesús CRD, de la Rosa RAR, Lerbekmo JF, Brinkman DB, Sampson SD. Cerro del Pueblo Fm (Difunta Group, Upper Cretaceous), Parras Basin, southern Coahuila, Mexico: reference sections, age, and correlation. Revista Mexicana de Ciencias Geológicas. 2004;21: 335–352.

97.

Eberth DA, Evans DC, Brinkman DB, Therrien F, Tanke DH, Russell LS. Dinosaur biostratigraphy of the Edmonton Group (Upper Cretaceous), Alberta, Canada: evidence for climate influence. Can J Earth Sci. 2013;50: 701–726. doi:10.1139/cjes-2012-0185

98.

Eberth DA, Hamblin AP. Tectonic, stratigraphic, and sedimentologic significance of a regional discontinuity in the upper Judith River Group (Belly River wedge) of southern Alberta, Saskatchewan, and northern Montana. Canadian Journal of Earth Sciences. 1993;30: 174–200. doi:10.1139/e93-016

99.

Eberth DA. The geology. In: Currie PJ, Koppelhus EB, editors. Dinosaur Provincial Park: A spectacular ancient ecosystem revealed. Bloomington, Indiana: Indiana University Press; 2005. pp. 54–82.

100.

Eberth DA. A revised regional stratigraphy and stratigraphic architecture for the Horseshoe Canyon Formation: outcrop and subsurface. Field Trip 14, May 17–18, 2010. Canadian Society of Petroleum Geology; 2010.

101.

Eberth DA. A Revised chronostratigraphy for the Oldman, Dinosaur Park and Bearpaw formations at Dinosaur Provincial Park, Alberta, Canada [Internet]. Occasional lecture series; 2011; Royal Tyrrell Museum. Available: https://www.youtube.com/watch?v=plGYsW9sOyI

102.

Endt PM, Van der Leun C. Energy levels of A= 21-44 nuclei (V). Nuclear Physics A. 1973;214: 1–625.

103.

Endt PM. Energy levels of A= 21–44 nuclei (VII). Nuclear Physics A. 1990;521: 1–400.

104.

Evans DC, Bavington R, Campione NE. An unusual hadrosaurid braincase from the Dinosaur Park Formation and the biostratigraphy of *Parasaurolophus* (Ornithischia: Lambeosaurinae) from southern Alberta. Can J Earth Sci. 2009;46: 791–800. doi:10.1139/E09-050

105.

Evans DC, Reisz RR, Dupuis K. A juvenile *Parasaurolophus* (Ornithischia: Hadrosauridae) braincase from Dinosaur Provincial Park, Alberta, with comments on crest ontogeny in the genus. Journal of Vertebrate Paleontology. 2007;27: 642–650. doi:10.1671/0272-4634(2007)27[642:AJPOHB]2.0.CO;2

106.

Evans DC, Ryan MJ. Cranial anatomy of *Wendiceratops pinhornensis* gen. et sp. nov., a centrosaurine ceratopsid (Dinosauria: Ornithischia) from the Oldman Formation (Campanian), Alberta, Canada, and the evolution of ceratopsid nasal ornamentation. PLOS ONE. 2015;10: e0130007. doi:10.1371/journal.pone.0130007

107.

Evans DC, Schott RK, Larson DW, Brown CM, Ryan MJ. The oldest North American pachycephalosaurid and the hidden diversity of small-bodied ornithischian dinosaurs. Nature Communications. 2013;4: 1828. doi:10.1038/ncomms2749

108.

Evans DC, Williamson T, Loewen MA, Kirkland JI. Review of pachycephalosaurian dinosaurs from Grand Staircase-Escalante National Monument, southern Utah. In: Titus AL, Loewen MA, editors. At the Top of Grant Staircase: The Late Cretaceous of Southern Utah. Bloomington, Indiana: Indiana University Press; 2013. pp. 482–487.

109.

Fanti F, Catuneanu O. Stratigraphy of the Upper Cretaceous Wapiti Formation, west-central Alberta, Canada. Can J Earth Sci. 2009;46: 263–286. doi:10.1139/E09-020

110.

Fanti F, Catuneanu O. Fluvial sequence stratigraphy: the Wapiti Formation, west-central Alberta, Canada. Journal of Sedimentary Research. 2010;80: 320–338. doi:10.2110/jsr.2010.033

111.

Fanti F, Currie PJ, Burns ME. Taphonomy, age, and paleoecological implication of a new *Pachyrhinosaurus* (Dinosauria: Ceratopsidae) bonebed from the Upper Cretaceous (Campanian) Wapiti Formation of Alberta, Canada. Can J Earth Sci. 2015;52: 250–260. doi:10.1139/cjes-2014-0197

112.

Farke AA, Chok DJ, Herrero A, Scolieri B, Werning S. Ontogeny in the tube-crested dinosaur *Parasaurolophus* (Hadrosauridae) and heterochrony in hadrosaurids. PeerJ. 2013;e182.

113.

Farke AA, Ryan MJ, Barrett PM, Tanke DH, Braman DR, Loewen MA, et al. A new centrosaurine from the Late Cretaceous of Alberta, Canada, and the evolution of parietal ornamentation in horned dinosaurs. Acta Palaeontologica Polonica. 2011;56: 691–702. doi:10.4202/app.2010.0121

114.

Farke AA. Ceratopsid dinosaurs from the Upper Cretaceous Almond Formation of southwestern Wyoming. Rocky Mountain Geology. 2004;39: 1–5. doi:10.2113/gsrocky.39.1.1

115.

Fassett JE, Hinds JS. Geology and fuel resources of the Fruitland Formation and Kirtland shale of the San Juan Basin, New Mexico and Colorado. US Geological Survey Professional Paper. 1971;676. Available: http://www.osti.gov/scitech/biblio/6984417

116.

Fassett JE, Steiner MB. Precise age of C33N-C32R magnetic-polarity reversal, San Juan Basin, New Mexico and Colorado. New Mexico Geological Society Guidebook. 1997;48: 239–247.

117.

Fassett JE. The saga of the Ojo Alamo Sandstone; or the rock-stratigrapher and the paleontologist should be friends. In: Kilgore LW, Fassett JE, editors. Cretaceous and Tertiary Rocks of the Southern Colorado Plateau. Farmington, New Mexico: Four Corners Geological Society; 1973. pp. 123–130.

118.

Fiorillo AR, McCarthy PJ, Flaig PP, Brandlen E, Norton DW, Zippi P, et al. Paleontology and Paleoenvironmental interpretation of the Kikak-Tegoseak quarry (Prince Creek Formation: Late Cretaceous), northern Alaska: a multi-disciplinary study of a high-latitude ceratopsian dinosaur bonebed. In: Ryan MJ, Chinnery-Allgeier BJ, Eberth DA, editors. New Perspectives on Horned Dinosaurs The Royal Tyrrell Museum Ceratopsian Symposium. Bloomington, Indiana: Indiana University Press; 2010. pp. 456–477.

119.

Fiorillo AR, Tykoski RS. A new Maastrichtian species of the centrosaurine ceratopsid *Pachyrhinosaurus* from the North Slope of Alaska. Acta Palaeontologica Polonica. 2012;57: 561–573. doi:10.4202/app.2011.0033

120.

Fiorillo AR, Tykoski RS. A diminutive new tyrannosaur from the top of the world. PLOS ONE. 2014;9: e91287. doi:10.1371/journal.pone.0091287

121.

Fiorillo AR. Taphonomy and depositional setting of Careless Creek Quarry (Judith River Formation), Wheatland County, Montana, U.S.A. Palaeogeography, Palaeoclimatology, Palaeoecology. 1991;81: 281–311. doi:10.1016/0031-0182(91)90151-G

122.

Flight JN. Sequence stratigraphic analysis of the Fox Hills and Hell Creek Fms (Maastrichtian), eastern Montana and its relationship to dinosaur paleontology. M.S. thesis, Montana State University. 2004.

123.

Flores RM, Myers MD, Houseknecht DW, Stricker GD, Brizzolara DW, Ryherd TJ, et al. Stratigraphy and facies of Cretaceous Schrader Bluff and Prince Creek formations in Colville River Bluffs, North Slope, Alaska. US Geological Survey Professional Paper. 2007;1748: 45.

124.

Foreman BZ, Rogers RR, Deino AL, Wirth KR, Thole JT. Geochemical characterization of bentonite beds in the Two Medicine Formation (Campanian, Montana), including a new 40Ar/39Ar age. Cretaceous Research. 2008;29: 373–385.

125.

Forster CA, Sereno PC, Evans TW, Rowe T. A complete skull of *Chasmosaurus mariscalensis* (Dinosauria: Ceratopsidae) from the Aguja Formation (late Campanian) of West Texas. Journal of Vertebrate Paleontology. 1993;13: 161–170. doi:10.1080/02724634.1993.10011498

126.

Fouch TD, Lawton TF, Nichols DJ, Cashion WB, Cobban WA. Patterns and timing of synorogenic sedimentation in Upper Cretaceous rocks of central and northeast Utah. Mesozoic Paleogeography of the West-Central United States: Rocky Mountain Symposium 2. 1983; 305–336.

127.

Fowler D. Terrestrial Late Cretaceous stratigraphy of North America and the utility of ceratopsids in biostratigraphy. Society of Vertebrate Paleontology Annual Meeting. 2006;26: 63A.

128.

Fowler D. Anagenesis and long-term morphologic trends in Chasmosaurinae (Dinosauria: Ceratopsidae) revealed by a new high-resolution chronostratigraphic framework, ontogenetic analysis, and description of two new taxa. Journal of Vertebrate Paleontology. 2010;30: 91A.

129.

Fowler DW. Dinosaurs and Time: Chronostratigraphic frameworks and their utility in analysis of dinosaur paleobiology. Ph.D dissertation, Montana State University. 2016.

130.

Franczyk KJ, Pitman JK, Nichols DJ. Sedimentology, mineralogy, palynology, and depositional history of some uppermost Cretaceous and lowermost Tertiary rocks along the Utah Book and Roan cliffs east of the Green River. US Geological Survey Bulletin. 1990;1787–N: 1–27.

131.

Frederiksen NO, McIntyre DJ, Sheehan TP. Palynological dating of some Upper Cretaceous to Eocene outcrop and well samples from the region extending from the easternmost part of NPRA in Alaska to the western part of ANWR. North Slope of Alaska: US Geological Survey, Open-File Report. 2002; 02–405.

132.

Frederiksen NO. Pollen zonation and correlation of Maastrichtian marine beds and associated strata, Ocean Point dinosaur locality, North Slope of Alaska. US Geological Survey Bulletin. 1991;B 1990-E: 1–22.

133.

Freedman Fowler EA, Horner JR. A new brachylophosaurin hadrosaur (Dinosauria: Ornithischia) with an intermediate nasal crest from the Campanian Judith River Formation of northcentral Montana. PLOS ONE. 2015;10: e0141304. doi:10.1371/journal.pone.0141304

134.

Gabriel DL, Berghaus CB. Three new specimens of *Stygimoloch spinifer* (Ornithischia: Pachycephalosauridae) and behavior inferences based on cranial morphology. International Symposium on Vertebrate Behavior as Derived from the Fossil Record Museum of the Rockies, Montana State University, Bozeman, Montana. 1988.

135.

Gaffney ES, Tong H, Meylan PA. Evolution of the side-necked turtles: the families Bothremydidae, Euraxemydidae, and Araripemydidae. Bulletin of the American Museum of Natural History. 2006; 1–698.

136.

Galiano H, Mehling C. A Sue site surprise! (A new pachycephalosaurid). Dino Press. 2001;4: 2–3.

137.

Galton PM, Sues H-D. New data on pachycephalosaurid dinosaurs (Reptilia: Ornithischia) from North America. Canadian Journal of Earth Sciences. 1983;20: 462–472.

138.

Galton PM. A primitive dome-headed dinosaur (Ornithischia: Pachycephalosauridae) from the Lower Cretaceous of England and the function of the dome of pachycephalosaurids. Journal of Paleontology. 1971; 40–47.

139.

Gangloff RA, Fiorillo AR, Norton DW. The first pachycephalosaurine (Dinosauria) from the paleo-arctic of Alaska and its paleogeographic implications. Journal of Paleontology. 2005;79: 997–1001.

140.

Gangloff RA, Fiorillo AR. Taphonomy and paleoecology of a bonebed from the Prince Creek Formation, North Slope, Alaska. PALAIOS. 2010;25: 299–317. doi:10.2110/palo.2009.p09-103r

141.

Gates TA, Sampson SD, Zanno LE, Roberts EM, Eaton JG, Nydam RL, et al. Biogeography of terrestrial and freshwater vertebrates from the late Cretaceous (Campanian) Western Interior of North America. Palaeogeography, Palaeoclimatology, Palaeoecology. 2010;291: 371–387. doi:10.1016/j.palaeo.2010.03.008

142.

Gates TA, Horner JR, Hanna RR, Nelson CR. New unadorned hadrosaurine hadrosaurid (Dinosauria, Ornithopoda) from the Campanian of North America. Journal of Vertebrate Paleontology. 2011;31: 798–811. doi:10.1080/02724634.2011.577854

143.

Gates TA, Lund EK, Boyd CA, DeBlieux DD, Titus AL, Evans DC, et al. Ornithopod dinosaurs from the Grand Staircase-Escalante National Monument region, Utah and their role in paleobiogeographic and macroevolutionary studies. Advances in Late Cretaceous Western Interior Basin Paleontology and Geology. In: Titus AL, Loewen MA, editors. At the top of the Grand Staircase: The Late Cretaceous of southern Utah. Bloomington, Indiana: Indiana University Press; 2013. pp. 463–481.

144.

Gates TA, Sampson SD, Jesús CRDD, Zanno LE, Eberth D, Hernandez-Rivera R, et al. *Velafrons coahuilensis*, a new lambeosaurine hadrosaurid (Dinosauria: Ornithopoda) from the late Campanian Cerro del Pueblo Formation, Coahuila, Mexico. Journal of Vertebrate Paleontology. 2007;27: 917–930. doi:10.1671/0272-4634(2007)27[917:VCANLH]2.0.CO;2

145.

Gates TA, Sampson SD. A new species of *Gryposaurus* (Dinosauria: Hadrosauridae) from the late Campanian Kaiparowits Formation, southern Utah, USA. Zoological Journal of the Linnean Society. 2007;151: 351–376. doi:10.1111/j.1096-3642.2007.00349.x

146.

Gates TA. Taxonomy, biogeography, and paleoecology of North American hadrosaurid (Ornithopoda) dinosaurs. Ph.D dissertation, University of Utah. 2007.

147.

Gee JS, Kent DV. Source of oceanic magnetic anomalies and the geomagnetic polarity timescale. In: Schubert G, editor. Treatise on Geophysics. Elsevier; 2007. pp. 455–507.

148.

Giffin EB, Gabriel DL, Johnson RE. A new pachycephalosaurid skull (Ornithischia) from the Cretaceous Hell Creek Formation of Montana. Journal of Vertebrate Paleontology. 1987;7: 398–407.

149.

Giffin EB. Notes on pachycephalosaurs (Ornithischia). Journal of Paleontology. 1989;63: 525–529.

150.

Gill JR, Cobban WA. Stratigraphy and geologic history of the Montana Group and equivalent rocks, Montana, Wyoming, and North and South Dakota. US Geological Survey Professional Paper. 1973;776: 37.

151.

Gill JR, Hail Jr. WJ. Stratigraphic sections across Upper Cretaceous Mancos Shale-Mesaverde Group boundary, eastern Utah and western Colorado [Internet]. 1975. Report No.: 68.

152.

Gill JR, Cobban WA, Schultz LG. Correlation, ammonite zonation, and a reference section for the Montana Group, central Montana. Montana Geological Society, Twenty-First Annual Geological Conference: Crazy Mountains Basin. 1972;21: 91–97.

153.

Gilmore CW. On the skull and skeleton of *Hypacrosaurus*, a helmet-crested dinosaur from the Edmonton Cretaceous of Alberta. Canada Geological Survey Bulletin. 1924;38: 49–89.

154.

Gilmore CW. A new carnivorous dinosaur from the Lance Formation of Montana. Smithsonian Miscellaneous Collections. 1946;106: 1–19.

155.

Gilmore CW. Reptilian fauna of the North Horn Formation of central Utah. US Geological Survey Professional Paper 210-C. 1946; 1–52.

156.

Gilmore CW. A new sauropod dinosaur from the Ojo Alamo Formation of New Mexico. Smithsonian Miscellaneous Collections. 1922;72: 1–9.

157.

Gilmore CW. On dinosaurian reptiles from the Two Medicine Formation of Montana. Proceedings of the United States National Museum. 1930;77: 1–39.

158.

Gilmore CW. Ceratopsian dinosaurs from the Two Medicine Formation, Upper Cretaceous of Montana. Proceedings of the United States National Museum. 1939;87: 1–18.

159.

Gilmore CW. A new species of troödont dinosaur from the Lance Formation of Wyoming. Proceedings of the United States National Museum. 1931;79: 1–11.

160.

Godfrey SJ, Holmes R. Cranial morphology and systematics of *Chasmosaurus* (Dinosauria: Ceratopsidae) from the Upper Cretaceous of western Canada. Journal of Vertebrate Paleontology. 1995;15: 726–742. doi:10.1080/02724634.1995.10011258

161.

Goodwin MB, Buchholtz EA, Johnson RE. Cranial anatomy and diagnosis of *Stygimoloch spinifer* (Ornithischia: Pachycephalosauria) with comments on cranial display structures in agonistic behavior. Journal of Vertebrate Paleontology. 1998;18: 363–375.

162.

Goodwin MB, Deino AL. The first radiometric ages from the Judith River Formation (Upper Cretaceous), Hill County, Montana. Can J Earth Sci. 1989;26: 1384–1391. doi:10.1139/e89-118

163.

Gradstein F, Ogg J. Geologic Time Scale 2004 – why, how, and where next! Lethaia. 2004;37: 175–181. doi:10.1080/00241160410006483

164.

Gradstein FM, Agterberg FP, Ogg JG, Hardenbol J, van Veen P, Thierry J, et al. A Mesozoic time scale. J Geophys Res. 1994;99: 24051–24074. doi:10.1029/94JB01889

165.

Gradstein FM, Ogg JG, Smith AG. A Geologic Time Scale 2004. Cambridge, UK: Cambridge University Press; 2004.

166.

Gradstein FM, Ogg JG, Schmitz M, Ogg G. The Geologic Time Scale 2012. Oxford, UK: Elsevier; 2012.

167.

Hamblin AP, Abrahamson BW. Stratigraphic architecture of “basal Belly River” cycles, Foremost Formation, Belly River Group, subsurface of southern Alberta and southwestern Saskatchewan. Bulletin of Canadian Petroleum Geology. 1996;44: 654–673.

168.

Hamblin AP. Edmonton Group/St. Mary River Formation: summary of literature and concepts. Geological Survey of Canada, Open File. 1998;3578: 1–36.

169.

Hamblin AP. Scollard/Willow Creek/Coalspur formations: summary of literature and concepts. 2010;

170.

Haq BU, Hardenbol J, Vail PR. Chronology of fluctuating sea levels since the Triassic. Science. 1987;235: 1156–1167.

171.

Haq BU, Hardenbol J, Vail PR. Mesozoic and Cenozoic chronostratigraphy and cycles of sea-level change. In: Wilgus CK, Hastings BS, Kendall CGSC, Ross CA, Van Wagoner JC, editors. Sea-Level Changes: An Integrated Approach,  SEPM Special Publication 42. Tulsa, Oklahoma: Society for Sedimentary Geology; 1988. pp. 7–108.

172.

Harrell TLJ, Martin JE. A mosasaur from the Maastrichtian Fox Hills Formation of the northern Western Interior Seaway of the United States and the synonymy of *Mosasaurus maximus* with *Mosasaurus hoffmanni* (Reptilia: Mosasauridae). Netherlands Journal of Geosciences. 2015;94: 23–37. doi:10.1017/njg.2014.27

173.

Hartman JH, Butler RD, Weiler MW, Schumaker KK. Context, naming, and formal designation of the Cretaceous Hell Creek Formation lectostratotype, Garfield County, Montana. Geological Society of America Special Papers. 2014;503: 89–121.

174.

Hartman JH, Kirkland JI. Brackish and marine mollusks of the Hell Creek Formation of North Dakota: evidence for a persisting Cretaceous seaway. Geological Society of America Special Papers. 2002;361: 271–296. doi:10.1130/0-8137-2361-2.271

175.

Hatcher JB, Osborn HF, Marsh OC. The Ceratopsia. Monographs of the United States Geological Survey. 1907;49: 1–300.

176.

Heckert AB, Lewis C, Lucas S, Williamson T. New mixed marine-nonmarine microvertebrate fauna from the Upper Cretaceous (late Santonian-early Campanian) Menefee Formation, northwestern New Mexico. Geological Society of America Abstracts with Programs. 2007;39: 73.

177.

Hettinger RD, Kirschbaum MA. Stratigraphy of the Upper Cretaceous Mancos Shale (upper part) and Mesaverde Group in the southern part of the Uinta and Piceance basins, Utah and Colorado. US Geological Survey Geologic Investigations Series. 2002;I-2764: 1–21.

178.

Hicks JF, Fastovsky D, Nichols DJ, Watabe M. Magnetostratigraphic correlation of Late Cretaceous dinosaur-bearing localities in the Nemegt and Ulan Nuur basins, Gobi Desert, Mongolia. Abstracts with Programs - Geological Society of America. 2001;33: 323.

179.

Hicks JF, Johnson KR, Obradovich JD, Miggins DP, Tauxe L. Magnetostratigraphy of Upper Cretaceous (Maastrichtian) to lower Eocene strata of the Denver Basin, Colorado. Rocky Mountain Geology. 2003;38: 1–27.

180.

Hicks JF, Johnson KR, Obradovich JD, Tauxe L, Clark D. Magnetostratigraphy and geochronology of the Hell Creek and basal Fort Union Formations of southwestern North Dakota and a recalibration of the age of the Cretaceous-Tertiary boundary. Geological Society of America Special Papers. 2002;361: 35–55.

181.

Hicks JF, Obradovich JD, Tauxe L. A new calibration point for the Late Cretaceous time scale: The (40Ar/39Ar isotopic age of the C33r/C33n geomagnetic reversal from the Judith River Formation (Upper Creataceous), Elk Basin, Wyoming, USA. Journal of Geology. 1995;103: 243–256.

182.

Hicks JF, Watabe M, Fastovsky DE, Johnson KR, Nichols DJ. Magnetostratigraphic correlation of Late Cretaceous dinosaur-bearing localities, Nemegt Basin, Gobi Desert, Mongolia. Abstracts with Programs - Geological Society of America. 1999;31: 234.

183.

Hofmann MH, Wroblewski A, Boyd R. Mechanisms controlling the clustering of fluvial channels and the compensational stacking of cluster belts. Journal of Sedimentary Research. 2011;81: 670–685. doi:10.2110/jsr.2011.54

184.

Hoganson JW, Murphy EC. Marine Breien Member (Maastrichtian) of the Hell Creek Formation in North Dakota: Stratigraphy, vertebrate fossil record, and age. Geological Society of America Special Papers. 2002;361: 247–269. doi:10.1130/0-8137-2361-2.247

185.

Holmes RB, Forster CA, Ryan M, Shepherd KM. A new species of *Chasmosaurus* (Dinosauria: Ceratopsia) from the Dinosaur Park Formation of southern Alberta. Canadian Journal of Earth Sciences. 2001;38: 1423–1438.

186.

Holroyd PA, Wilson GP, Hutchison JH. Temporal changes within the latest Cretaceous and early Paleogene turtle faunas of northeastern Montana. Geological Society of America Special Papers. 2014;503: 299–312. doi:10.1130/2014.2503(11)

187.

Horner JR, Currie PJ. Embryonic and neonatal morphology and ontogeny of a new species of *Hypacrosaurus* (Ornithischia, Lambeosauridae) from Montana and Alberta. Dinosaur Eggs and Babies. Cambridge, UK: Cambridge University Press; 1994. pp. 312–336.

188.

Horner JR, Goodwin MB. Extreme cranial ontogeny in the Upper Cretaceous dinosaur *Pachycephalosaurus*. PLoS ONE. 2009;4: e7626. doi:10.1371/journal.pone.0007626

189.

Horner JR, Makela R. Nest of juveniles provides evidence of family structure among dinosaurs. Nature. 1979;282: 296–298. doi:10.1038/282296a0

190.

Horner JR, Schmitt JG, Jackson F, Hanna R. Bones and rocks of the Upper Cretaceous Two Medicine-Judith River clastic wedge complex, Montana. Field trip guidebook, Society of Vertebrate Paleontology 61st Annual Meeting: Mesozoic and Cenozoic Paleontology in the Western Plains and Rocky Mountains Museum of the Rockies Occasional Paper. 2001. pp. 3–14.

191.

Horner JR, Varricchio DJ, Goodwin MB. Marine transgressions and the evolution of Cretaceous dinosaurs. Nature. 1992;358: 59–61. doi:10.1038/358059a0

192.

Horner JR. Upper Cretaceous dinosaurs from the Bearpaw Shale (marine) of south-central Montana with a checklist of Upper Cretaceous dinosaur remains from marine sediments in North America. Journal of Paleontology. 1979;53: 566–577.

193.

Horner JR. Three ecologically distinct vertebrate faunal communities from the Late Cretaceous Two Medicine Formation of Montana, with discussion of evolutionary pressures induced by seaway fluctuations. Montana Geological Society Guidebook. 1984;1984: 299–303.

194.

Horner JR. Cranial morphology of *Prosaurolophus* (Ornithischia: Hadrosauridae): with descriptions of two new hadrosaurid species and an evaluation of hadrosaurid phylogenetic relationships. Museum of the Rockies Occasional Paper. 1992; 1–119.

195.

Hunt AP, Lucas SG. Stratigraphy, paleontology and age of the Fruitland and Kirtland formations (Upper Cretaceous), San Juan Basin, New Mexico. New Mexico Geological Society Guidebook. 1992;43: 217–239.

196.

Hunt AP, Lucas SG. Cretaceous Vertebrates of New Mexico. New Mexico Museum of Natural History and Science Bulletin. 1993;2: 77–91.

197.

Hunt AP, Lucas SG. Origin and stratigraphy of historic dinosaur quarries in the Upper Cretaceous Fruitland Formation of the Fossil Forest Research Natural Area, northwestern New Mexico. New Mexico Geological Society Guidebook. 2003;54: 383–388.

198.

Hunt AP. “Bisti member” of the Kirtland Shale, San Juan Basin, New Mexico: implications for basin development. Geological Society of America, Abstracts with Programs. 1986;24: 26–27.

199.

Hunt RK, Lehman TM. Attributes of the ceratopsian dinosaur *Torosaurus*, and new material from the Javelina Formation (Maastrichtian) of Texas. Journal of Paleontology. 2008;82: 1127–1138. doi:10.1017/S0022336000055335

200.

Hunter JP, Heinrich RE, Weishampel DB. Mammals from the St. Mary River Formation (Upper Cretaceous), Montana. Journal of Vertebrate Paleontology. 2010;30: 885–898. doi:10.1080/02724631003763490

201.

Ifrim C, Stinnesbeck W, Garza RR, Ventura JF. Hemipelagic cephalopods from the Maastrichtian (Late Cretaceous) Parras Basin at La Parra, Coahuila, Mexico, and their implications for the correlation of the lower Difunta Group. Journal of South American Earth Sciences. 2010;29: 597–618.

202.

Ifrim C, Stinnesbeck W, Ventura JF. An endemic cephalopod assemblage from the lower Campanian (Late Cretaceous) Parras Shale, western Coahuila, Mexico. Journal of Paleontology. 2013;87: 881–901. doi:10.1666/12-123

203.

Imhof M, Albright LB. Preliminary magnetostratigraphic analysis of the Upper Cretaceous Kaiparowits Formation, southern Utah. Journal of Vertebrate Paleontology. 2003;23: 65A.

204.

Izett GA, Cobban WA, Obradovich JD, Dalrymple GB. 40Ar/39Ar age of the Manson impact structure, Iowa, and correlative impact ejecta in the Crow Creek Member of the Pierre Shale (Upper Cretaceous), South Dakota and Nebraska. Geological Society of America Bulletin. 1998;110: 361–376.

205.

Izett GA, Obradovich JD. 40Ar/39Ar age constraints for the Jaramillo normal subchron. Journal of Geophysical research. 1994;99: 2925–2934.

206.

Jasinski SE, Sullivan RM. The validity of the Late Cretaceous pachycephalosaurid *Stegoceras novomexicanum* (Dinosauria: Pachycephalosauridae). New Mexico Museum of Natural History and Science Bulletin. 2016;74: 107–115.

207.

Jasinski SE, Sullivan RM. Re-evaluation of pachycephalosaurids from the Fruitland-Kirtland transition (Kirtlandian, Late Campanian), San Juan Basin, New Mexico, with a description of a new species of *Stegoceras* and a reassessment of *Texacephale langstoni*. Fossil Record 3: Bulletin 53. 2011;53: 202–215.

208.

Jeletzky JA, Clemens WA. Comments on Cretaceous Eutheria, Lance *Scaphites*, and *Inoceramus*? ex gr. *tegulatus*. Journal of Paleontology. 1965;39: 952–959.

209.

Jerzykiewicz T, Norris DK. Stratigraphy, structure and syntectonic sedimentation of the Campanian “Belly River” clastic wedge in the southern Canadian Cordillera. Cretaceous Research. 1994;15: 367–399. doi:10.1006/cres.1994.1022

210.

Jinnah ZA, Roberts EM, Deino AL, Larsen JS, Link PK, Fanning CM. New 40Ar-39Ar and detrital zircon U-Pb ages for the Upper Cretaceous Wahweap and Kaiparowits formations on the Kaiparowits Plateau, Utah: implications for regional correlation, provenance, and biostratigraphy. Cretaceous Research. 2009;30: 287–299. doi:10.1016/j.cretres.2008.07.012

211.

Jinnah ZA, Roberts EM. Facies Associations, Paleoenvironment, and Base-Level Changes in the Upper Cretaceous Wahweap Formation, Utah, U.S.A. Journal of Sedimentary Research. 2011;81: 266–283. doi:10.2110/jsr.2011.22

212.

Jinnah ZA. Tectonic and Sedimentary Controls, Age, and Correlation of the Upper Cretaceous Wahweap Formation, Southern Utah. In: Titus AL, Loewen MA, editors. At the Top of the Grand Staircase: The Late Cretaceous of Southern Utah. Bloomington, Indiana: Indiana University Press; 2013. pp. 57–73.

213.

Jinnah ZA. Tectonic and sedimentary controls, age and correlation of the Upper Cretaceous Wahweap Formation, southern Utah, USA [Internet]. Ph.D dissertation, University of the Witwatersrand. 2011.

214.

Jourdan F, Verati C, Féraud G. Intercalibration of the Hb3gr 40 Ar/39 Ar dating standard. Chemical Geology. 2006;231: 177–189.

215.

Kauffman EG, Sageman BB, Kirkland JI, Elder WP, Harries PJ, Villamil T. Molluscan biostratigraphy of the Cretaceous Western Interior Basin, North America. Evolution of the Western Interior Basin: Geological Association of Canada Special Paper. 1993;39: 397–434.

216.

Kennedy WJ, Landman NH, Christensen WK, Cobban WA, Hancock JM. Marine connections in North America during the late Maastrichtian: palaeogeographic and palaeobiogeographic significance of *Jeletzkytes nebrascensis* Zone cephalopod fauna from the Elk Butte Member of the Pierre Shale, SE South Dakota and NE Nebraska. Cretaceous Research. 1998;19: 745–775. doi:10.1006/cres.1998.0129

217.

Kirkland J, DeBlieux D. Dinosaur remains from the lower to middle Campanian Wahweap Formation at Grand Staircase-Escalante National Monument, southern Utah. Journal of Vertebrate Paleontology. 2005;25: 78A.

218.

Kirkland JI, DeBlieux DD. New horned dinosaurs from the Wahweap Formation. Utah Geological Survey Survey Notes. 2007;39: 4–5.

219.

Kirkland JI, DeBlieux DD. New basal centrosaurine ceratopsian skulls from the Wahweap Formation (Middle Campanian), Grand Staircase-Escalante National Monument, southern Utah. In: Ryan MJ, Chinnery-Allgeier BJ, Eberth DA, editors. New Perspectives on Horned Dinosaurs: The Royal Tyrrell Museum Ceratopsian Symposium. Bloomington, Indiana: Indiana University Press; 2010. pp. 117–140.

220.

Kirkland JI, Hernández-Rivera R, Gates T, Paul GS, Nesbitt SJ, Serrano-Brañas CI, et al. Large hadrosaurine dinosaurs from the latest Campanian of Coahuila, Mexico. New Mexico Museum of Natural History and Science Bulletin. 2006;35: 299–315.

221.

Kirkland JI, Hernández-Rivera R, Aguillón Martínez MC, Delgado de Jesús CR, Gómez-Nuñez R, Vallejo I. The Late Cretaceous Difunta Group of the Parras Basin, Coahuila, Mexico, and its vertebrate fauna. Society of Vertebrate Paleontology Annual Meeting, Field Trip Guidebook. Pachuca, Mexico: Universidad autonoma del Estado de Hidalgo, Ciudad Universitaria; 2000. pp. 133–172.

222.

Kirkland JI. The quest for new dinosaurs at Grand Staircase–Escalante National Monument: Utah Geological Survey Notes, v. 33. Utah Geological Survey Survey Notes. 2001;33: 4.

223.

Kirschbaum MA, Hettinger RD. Facies analysis and sequence stratigraphic framework of upper Campanian strata (Neslen and Mount Garfield formations, Bluecastle Tongue of the Castlegate sandstone, and Mancos shale), eastern Book Cliffs, Colorado and Utah. US Geological Survey, Digital Data Series. 2004;69–G: 1–40.

224.

Krystinik LF, DeJarnett BB. Lateral variability of sequence stratigraphic framework in the Campanian and Lower Maastrichtian of the Western Interior Seaway. American Association of Petroleum Geologists, Memoir. 1995;64: 11–25.

225.

Kuiper KF, Deino A, Hilgen FJ, Krijgsman W, Renne PR, Wijbrans JR. Synchronizing rock clocks of earth history. Science. 2008;320: 500–504. doi:10.1126/science.1154339

226.

Kwon J, Min K, Bickel PJ, Renne PR. Statistical methods for jointly estimating the decay constant of 40K and the age of a dating standard. Mathematical Geology. 2002;34: 457–474. doi:10.1023/A:1015035228810

227.

Lambe LM. New genera and species from the Belly River series (Mid-Cretaceous). Contributions to Canadian Palaeontology. 1902;3: 25–81.

228.

Lambe LM. On the squamoso-parietal crest of the horned dinosaurs *Centrosaurus apertus* and *Monoclonius canadensis* from the Cretaceous of Alberta. The Ottawa Naturalist. 1904;18: 81–84.

229.

Lambe LM. A new genus and species of ceratopsia from Belly River Formation of Alberta. The Ottawa Naturalist. 1913;27: 109–116.

230.

Lambe LM. On *Gryposaurus notabilis*, a new genus and species of trachodont dinosaur from the Belly River Formation of Alberta, with a description of the skull of *Chasmosaurus belli*. The Ottawa Naturalist. 1914;27: 145–155.

231.

Lambe LM. A new genus and species of crestless hadrosaur from the Edmonton Formation of Alberta. The Ottawa Naturalist. 1917;31: 65–73.

232.

Lambe LM. The Cretaceous genus *Stegoceras*, typifying a new family referred provisionally to the Stegosauria. Transactions of the Royal Society of Canada. 1918;12: 23–36.

233.

Landman NH, Johnson RO, Edwards LE. Cephalopods from the Cretaceous/Tertiary boundary interval on the Atlantic Coastal Plain, with a description of the highest ammonite zones in North America. Part 2. Northeastern Monmouth County, New Jersey. Bulletin of the American Museum of Natural History. 2004; 1–107.

234.

Landman NH, Waagé KM (Karl M. Scaphitid ammonites of the Upper Cretaceous (Maastrichtian) Fox Hills Formation in South Dakota and Wyoming. Bulletin of the American Museum of Natural History. 1993;215: 1–257.

235.

Langston W. *Anchiceratops* from the Oldman Formation of Alberta. Natural History Papers. 1959;3: 11.

236.

Langston W. The ceratopsian dinosaurs and associated lower vertebrates from the St. Mary River Formation (Maestrichtian) at Scabby Butte, southern Alberta. Can J Earth Sci. 1975;12: 1576–1608. doi:10.1139/e75-142

237.

Langston W. A Late Cretaceous vertebrate fauna from the St. Mary River Formation in western Canada. Athalon, Royal Ontario Museum, Life Sciences Miscellaneous Publications. 1976;9: 114–133.

238.

Lanphere MA, Dalrymple GB, Fleck RJ, Pringle MS. Intercalibration of mineral standards for K-Ar and 40Ar/39Ar age measurements. EOS, Transactions of the American Geophysical Union. 1990;71: 1–658.

239.

LaRock JW. Sedimentology and taphonomy of a dinosaur bonebed from the Upper Cretaceous (Campanian) Judith River Formation of north central Montana [Internet]. Montana State University. 2000.

240.

Larson DW. Diversity and variation of theropod dinosaur teeth from the uppermost Santonian Milk River Formation (Upper Cretaceous), Alberta: a quantitative method supporting identification of the oldest dinosaur tooth assemblage in Canada. Can J Earth Sci. 2008;45: 1455–1468. doi:10.1139/E08-070

241.

Lawson DA. *Tyrannosaurus* and *Torosaurus*, Maastrichtian dinosaurs from Trans-Pecos, Texas. Journal of Paleontology. 1976;50: 158–164.

242.

Lawton TF, Christensen AE. Sequence boundaries in terrestrial foreland-basin strata: do they lie above or below the amalgamated fluvial facies tract? AAPG Search and Discovery. Calgary, Alberta: AAPG; 2005. p. #90039.

243.

Lawton TF, Pollock SL, Robinson R a. J. Integrating sandstone petrology and nonmarine sequence stratigraphy: application to the Late Cretaceous fluvial systems of southwestern Utah, U.S.A. Journal of Sedimentary Research. 2003;73: 389–406. doi:10.1306/100702730389

244.

Lawton TF. Chapter 12 Laramide Sedimentary Basins. In: Miall AD, editor. Sedimentary Basins of the World. Oxford, UK: Elsevier; 2008. pp. 429–450.

245.

Leahy GD, Lerbekmo JF. Macrofossil magnetobiostratigraphy for the upper Santonian-lower Campanian interval in the Western Interior of North America: comparisons with European stage boundaries and planktonic foraminiferal zonal boundaries. Canadian Journal of Earth Sciences. 1995;32: 247–260.

246.

LeCain R, Clyde WC, Wilson GP, Riedel J. Magnetostratigraphy of the Hell Creek and lower Fort Union formations in northeastern Montana. Geological Society of America Special Papers. 2014;503: 137–147. doi:10.1130/2014.2503(04)

247.

Lehman TM, Coulson AB. A juvenile specimen of the sauropod dinosaur *Alamosaurus sanjuanensis* from the Upper Cretaceous of Big Bend National Park, Texas. Journal of Paleontology. 2002;76: 156–172. doi:10.1017/S0022336000017431

248.

Lehman TM, Mcdowell FW, Connelly JN. First isotopic (U-Pb) age for the Late Cretaceous *Alamosaurus* vertebrate fauna of west Texas, and its significance as a link between two faunal provinces. Journal of Vertebrate Paleontology. 2006;26: 922–928. doi:10.1671/0272-4634(2006)26[922:FIUAFT]2.0.CO;2

249.

Lehman TM, Tomlinson SL. *Terlinguachelys fischbecki*, a new genus and species of sea turtle (Chelonioidea: Protostegidae) from the Upper Cretaceous of Texas. Journal of Paleontology. 2004;78: 1163–1178. doi:10.1017/S0022336000043973

250.

Lehman TM. Late Maastrichtian paleoenvironments and dinosaur biogeography in the Western Interior of North America. Palaeogeography, Palaeoclimatology, Palaeoecology. 1987;60: 189–217. doi:10.1016/0031-0182(87)90032-0

251.

Lehman TM. *Chasmosaurus mariscalensis*, sp. nov., a new ceratopsian dinosaur from Texas. Journal of Vertebrate Paleontology. 1989;9: 137–162. doi:10.1080/02724634.1989.10011749

252.

Lehman TM. The ceratopsian subfamily Chasmosaurinae: sexual dimorphism and systematics. In: Carpenter K, Currie PJ, editors. Dinosaur Systematics: Approaches and Perspectives. Cambridge, UK: Cambridge University Press; 1990. pp. 211–229.

253.

Lehman TM. Paleosols and the Cretaceous/Tertiary transition in the Big Bend region of Texas. Geology. 1990;18: 362–364. doi:10.1130/0091-7613(1990)018<0362:PATCTT>2.3.CO;2

254.

Lehman TM. New data on the ceratopsian dinosaur *Pentaceratops sternbergii* Osborn from New Mexico. Journal of Paleontology. 1993;67: 279–288.

255.

Lehman TM. Late Cretaceous dinosaur provinciality. In: Tanke DH, Carpenter K, editors. Mesozoic Vertebrate Life. Bloomington, Indiana: Indiana University Press; 2001. pp. 310–328.

256.

Lehman TM. Stratigraphy, sedimentology, and paleontology of upper Cretaceous (Campanian-Maastrichtian) sedimentary rocks in Trans-Pecos Texas. Ph.D, University of Texas, Austin. 1985.

257.

Lehman TM. The Alamo Wash local fauna: a new look at the old Ojo Alamo fauna. Advances in San Juan Basin Paleontology New Mexico University Press, Albuquerque, New Mexico. 1981; 189–221.

258.

Leidy J. Remarks on some extinct vertebrates. Proceedings of the Academy of Natural Sciences of Philadelphia. 1872;24: 38–40.

259.

Lerbekmo JF, Braman DR, Catuneanu O, Humphrey NC. Magnetostratigraphic and palynostratigraphic correlation of Late Campanian strata of the Bearpaw and Horseshoe Canyon Formations of the RCA Castor Corehole to the Red Deer Valley, Alberta. Bulletin of Canadian Petroleum Geology. 2003;51: 70–77.

260.

Lerbekmo JF, Braman DR. Magnetostratigraphic and biostratigraphic correlation of late Campanian and Maastrichtian marine and continental strata from the Red Deer Valley to the Cypress Hills, Alberta, Canada. Can J Earth Sci. 2002;39: 539–557. doi:10.1139/e01-085

261.

Lerbekmo JF, Braman DR. Magnetostratigraphic and palynostratigraphic correlation of Late Campanian to Early Maastrichtian strata of the Bearpaw and Horseshoe Canyon formations between the CPOG Strathmore Corehole and the Red Deer Valley Section, Alberta, Canada. Bulletin of Canadian Petroleum Geology. 2005;53: 154–164.

262.

Lerbekmo JF, Evans ME. Cryptochrons and tiny wiggles: new magnetostratigraphic evidence from chrons 32 and 33 in western Canada. Physics of the Earth and Planetary Interiors. 2012;202: 8–13.

263.

Lerbekmo JF, Lehtola N. Magnetostratigraphy of the Bearpaw and Blood Reserve formations on the St. Mary River: Evidence for the effect of the Sweetgrass Arch. Bulletin of Canadian Petroleum Geology. 2011;59: 1–6.

264.

Lerbekmo JF, Sweet AR, Duke MJ. A normal polarity subchron that embraces the K/T boundary: A measure of sedimentary continuity across the boundary and synchroneity of boundary events. Geological Society of America Special Papers. 1996;307: 465–476.

265.

Lerbekmo JF. Magnetostratigraphic and biostratigraphic correlations of Maastrichtian to Early Paleocene strata between south-central Alberta and Southwestern Saskatchewan. Bulletin of Canadian Petroleum Geology. 1985;33: 213–226.

266.

Lerbekmo JF. The stratigraphic position of the 33-33r (Campanian) polarity chron boundary in southeastern Alberta. Bulletin of Canadian Petroleum Geology. 1989;37: 43–47.

267.

Lerbekmo JF. Magnetostratigraphy of the Canadian Continental Drilling Program Cretaceous-Tertiary (KT) Boundary Project core holes, western Canada. Canadian Journal of Earth Sciences. 1999;36: 705–715.

268.

Lerbekmo JF. The Dorothy bentonite: an extraordinary case of secondary thickening in a late Campanian volcanic ash fall in central Alberta. Can J Earth Sci. 2002;39: 1745–1754. doi:10.1139/e02-079

269.

Lerbekmo JF. Glacioeustatic sea level fall marking the base of supercycle TA-1 at 66.5 Ma recorded by the kaolinization of the Whitemud Formation and the Colgate Member of the Fox Hills Formation. Marine and Petroleum Geology. 2009;26: 1299–1303. doi:10.1016/j.marpetgeo.2008.08.001

270.

Lerbekmo JF. The Chicxulub-Shiva extraterrestrial one-two killer punches to Earth 65 million years ago. Marine and Petroleum Geology. 2014;49: 203–207. doi:10.1016/j.marpetgeo.2013.05.014

271.

Lerbekmo JF, Coulter KC. Late Cretaceous to early Tertiary magnetostratigraphy of a continental sequence: Red Deer Valley, Alberta, Canada. Canadian Journal of Earth Sciences. 1985;22: 567–583.

272.

Lerbekmo JF. Paleomagnetostratigraphy. In: Currie PJ, Koppelhus EB, editors. Dinosaur Provincial Park. Bloomington, Indiana: Indiana University Press; 2005. pp. 83–87.

273.

Lewis C, Heckert A, Lucas S, Williamson T. A diverse new microvertebrate fauna from the Upper Cretaceous (late Santonian-early Campanian) Menefee Formation of New Mexico. Journal of Vertebrate Paleontology. 2007;27: 105A.

274.

Lillegraven JA, McKenna MC. Fossil mammals from the “Mesaverde” Formation (Late Cretaceous, Judithian) of the Bighorn and Wind River basins, Wyoming : with definitions of Late Cretaceous North American land-mammal “ages.” Mesaverde mammals. 1986;2840: 1–68.

275.

Lindsay EH, Butler RF, Johnson NM. Magnetic polarity zonation and biostratigraphy of Late Cretaceous and Paleocene continental deposits, San Juan Basin, New Mexico. Am J Sci. 1981;281: 390–435. doi:10.2475/ajs.281.4.390

276.

Little WW. Tectonic and eustatic controls on cyclical fluvial patterns, Upper Cretaceous strata of the Kaiparowits Basin Utah. In: Hill LM, editor. Learning from the Land: Grand Staircase–Escalante National Monument Science Symposium Proceedings. Salt Lake City: U.S. Department of the Interior, Bureau of Land Management; 1997. pp. 489–504.

277.

Loewen MA, Farke AA, Sampson SD, Getty MA, Lund EK, O’Connor PM. Ceratopsid dinosaurs from the Grand Staircase of southern Utah. In: Titus AL, Loewen MA, editors. At the top of the Grand Staircase: the Late Cretaceous of Southern Utah. Bloomington, Indiana: Indiana University Press; 2013. pp. 488–503.

278.

Loewen MA, Irmis RB, Sertich JJW, Currie PJ, Sampson SD. Tyrant dinosaur evolution tracks the rise and fall of Late Cretaceous oceans. PLOS ONE. 2013;8: e79420. doi:10.1371/journal.pone.0079420

279.

Loewen MA, Sampson SD, Lund EK, Farke AA, Aguillón-Martínez MC, de Leon CA, et al. Horned dinosaurs (Ornithischia: Ceratopsidae) from the Upper Cretaceous (Campanian) Cerro del Pueblo Formation, Coahuila, Mexico. In: Ryan MJ, Chinnery-Allgeier BJ, Eberth DA, editors. New Perspectives on Horned Dinosaurs: The Royal Tyrrell Museum Ceratopsian Symposium. Bloomington, Indiana: Indiana University Press; 2010. pp. 99–116.

280.

Longrich N. Systematics of *Chasmosaurus* - new information from the Peabody Museum skull, and the use of phylogenetic analysis for dinosaur alpha taxonomy. F1000Research. 2015;4. doi:10.12688/f1000research.7573.1

281.

Longrich NR, Currie PJ. < i> Albertonykus borealis</i>, a new alvarezsaur (Dinosauria: Theropoda) from the Early Maastrichtian of Alberta, Canada: implications for the systematics and ecology of the Alvarezsauridae. Cretaceous Research. 2009;30: 239–252.

282.

Longrich NR, Sankey J, Tanke D. *Texacephale langstoni*, a new genus of pachycephalosaurid (Dinosauria: Ornithischia) from the upper Campanian Aguja Formation, southern Texas, USA. Cretaceous Research. 2010;31: 274–284.

283.

Longrich NR. *Mojoceratops perifania*, a new chasmosaurine ceratopsid from the Late Campanian of western Canada. Journal of Paleontology. 2010;84: 681–694.

284.

Longrich NR. The horned dinosaurs *Pentaceratops* and *Kosmoceratops* from the upper Campanian of Alberta and implications for dinosaur biogeography. Cretaceous Research. 2014;51: 292–308. doi:10.1016/j.cretres.2014.06.011

285.

Lorenz JC, Gavin W. Geology of the Two Medicine Formation and the sedimentology of a dinosaur nesting ground. Montana Geological Society 1984 Field Conference Guidebook. Montana Geological Society Helena; 1984. pp. 175–186.

286.

Lucas SG, Sullivan RM, Spielmann JA. Cretaceous vertebrate biochronology, North American Western Interior. Journal of Stratigraphy. 2012;36: 436–461.

287.

Lucas SG, Sullivan RM. The sauropod dinosaur *Alamosaurus* from the Upper Cretaceous of the San Juan Basin, New Mexico. New Mexico Museum of Natural History and Science Bulletin. 2000;17: 147–156.

288.

Lucas SG, Hunt AP, Sullivan RM. Stratigraphy and age of the Upper Cretaceous Fruitland Formation, west-central San Juan Basin, New Mexico. New Mexico Museum of Natural History and Science Bulletin. 2006;35: 1–6.

289.

Lucas SG, Spielmann JA, Kirkland JI, Foster JR, Sullivan RM. A juvenile hadrosaurine from the middle Campanian (Late Cretaceous) interval of the Mancos Shale, western Colorado. Late Cretaceous Vertebrates from the Western Interior: New Mexico Museum of Natural History and Science Bulletin. 2006;35: 281–292.

290.

Lucas SG, Sullivan RM, Cather SM, Jasinski SE, Fowler DW, Heckert AB, et al. No definitive evidence of Paleocene dinosaurs in the San Juan Basin. Paleontologia Electronica. 2009;12: 1–10.

291.

Lucas SG, Sullivan RM, Hunt AP. Re-evaluation of *Pentaceratops* and *Chasmosaurus* (Ornithischia: Ceratopsidae) in the Upper Cretaceous of the Western Interior. New Mexico Museum of Natural History and Science Bulletin. 2006;35: 367–370.

292.

Lull RS, Wright NE. Hadrosaurian dinosaurs of North America. Geological Society of America Special Papers. 1942;40: 1–272. doi:10.1130/SPE40-p1

293.

Lull RS. A revision of the Ceratopsia or horned dinosaurs. Memoirs of the Peabody Museum of Natural History. 1933;3: 175.

294.

Lund EK, Sampson SD, Loewen MA. *Nasutoceratops titusi* (Ornithischia, Ceratopsidae), a basal centrosaurine ceratopsid from the Kaiparowits Formation, southern Utah. Journal of Vertebrate Paleontology. 2016;36: e1054936.

295.

Machalski A, Jagt JWM, Landman NH, Motchurova-Dekova N. The highest records of North American scaphitid ammonites in the European Maastrichtian (Upper Cretaceous) and their stratigraphic implications. Acta Geologica Polonica. 2007;57: 169–185.

296.

Maidment SCR, Barrett PM. A new specimen of *Chasmosaurus belli* (Ornithischia: Ceratopsidae), a revision of the genus, and the utility of postcrania in the taxonomy and systematics of ceratopsid dinosaurs. Zootaxa. 2011;2963: 1–47. doi:10.11646/zootaxa.2963.1.1

297.

Malik AME. Sedimentology of the Judith River Formation in the Milk River Valley and the Little Rocky Mountains, Montana [Internet]. MS. 1990. Available: http://ecommons.usask.ca/handle/10388/etd-07112012-152552

298.

Mallon JC, Evans DC, Ryan MJ, Anderson JS. Megaherbivorous dinosaur turnover in the Dinosaur Park Formation (upper Campanian) of Alberta, Canada. Palaeogeography, Palaeoclimatology, Palaeoecology. 2012;350–352: 124–138. doi:10.1016/j.palaeo.2012.06.024

299.

Mallon JC, Holmes R, Anderson JS, Farke AA, Evans DC, Sues H-D. New information on the rare horned dinosaur *Arrhinoceratops brachyops* (Ornithischia: Ceratopsidae) from the Upper Cretaceous of Alberta, Canada. Canadian Journal of Earth Sciences. 2014;51: 618–634.

300.

Mallon JC, Holmes R, Eberth DA, Ryan MJ, Anderson JS. Variation in the skull of *Anchiceratops* (Dinosauria, Ceratopsidae) from the Horseshoe Canyon Formation (Upper Cretaceous) of Alberta. Journal of Vertebrate Paleontology. 2011;31: 1047–1071. doi:10.1080/02724634.2011.601484

301.

Mallon JC, Ott CJ, Larson PL, Iuliano EM, Evans DC. *Spiclypeus shipporum* gen. et sp. nov., a boldly audacious new chasmosaurine ceratopsid (Dinosauria: Ornithischia) from the Judith River Formation (Upper Cretaceous: Campanian) of Montana, USA. PLoS ONE. 2016;11: e0154218.

302.

Marsh OC. Notice of new reptiles from the Laramie Formation. American Journal of Science. 1892;s3-43: 449–453. doi:10.2475/ajs.s3-43.257.449

303.

Mason IP, Heizler MT, Williamson TE. 40 Ar/39 Ar sanidine chronostratigraphy of K-Pg boundary sediments of the San Juan Basin, NM. New Mexico Geological Society 2013 Spring Meeting. 2013. p. 42.

304.

McDonald AT, Horner JR, Ryan MJ, Chinnery-Allgeier BJ, Eberth DA. New material of “*Styracosaurus*” *ovatus* from the Two Medicine Formation of Montana. New Perspectives on Horned Dinosaurs. Bloomington, Indiana: Indiana University Press; 2010. pp. 156–168.

305.

McDonald AT. A subadult specimen of *Rubeosaurus ovatus* (Dinosauria: Ceratopsidae), with observations on other ceratopsids from the Two Medicine Formation. PLoS ONE. 2011;6: e22710. doi:10.1371/journal.pone.0022710

306.

McDougall I, Harrison TM. Geochronology and thermochronology by the 40Ar/39Ar method [Internet]. 2nd ed. Oxford, UK: Oxford University Press; 1999.

307.

McKenna MC, Love JD. Local stratigraphic and tectonic significance of *Leptoceratops*, a Cretaceous dinosaur in the Pinyon Conglomerate, northwestern Wyoming. United States Geological Survey, Professional Paper. 1970;700: 55–61.

308.

Meek FB, Hayden FV. Descriptions of New Lower Silurian, (Primordial), Jurassic, Cretaceous and Tertiary fossils: collected in Nebraska, by the Exploring Expeditions under the command of Capt. Wm. F. Raynolds. Proceedings of the Academy of Natural Sciences. 1861;13: 415–447.

309.

Meyers SR, Siewert SE, Singer BS, Sageman BB, Condon DJ, Obradovich JD, et al. Intercalibration of radioisotopic and astrochronologic time scales for the Cenomanian-Turonian boundary interval, Western Interior Basin, USA. Geology. 2012;40: 7–10. doi:10.1130/G32261.1

310.

Miall AD, Catuneanu O, Vakarelov BK, Post R. The Western Interior Basin. In: Andrew D. Miall, editor. Sedimentary Basins of the World. Oxford, UK: Elsevier; 2008. pp. 329–362.

311.

Min K, Mundil R, Renne PR, Ludwig KR. A test for systematic errors in 40 Ar/39 Ar geochronology through comparison with U/Pb analysis of a 1.1-Ga rhyolite. Geochimica et Cosmochimica Acta. 2000;64: 73–98.

312.

Molenaar CM, Cobban WA, Merewether EA, Pillmore CL, Wolfe DG, Holbrook JM. Regional stratigraphic cross sections of Cretaceous rocks from east-central Arizona to the Oklahoma panhandle. US Geological Survey Miscellaneous Field Studies Map. 2002;MF-2382: 1–6.

313.

Montgomery P, Hailwood EA, Gale AS, Burnett JA. The magnetostratigraphy of Coniacian-late Campanian chalk sequences in southern England. Earth and Planetary Science Letters. 1998;156: 209–224. doi:10.1016/S0012-821X(98)00008-9

314.

Mori H, Druckenmiller PS, Erickson GM. A new arctic hadrosaurid from the Prince Creek Formation (lower Maastrichtian) of northern Alaska. Acta Palaeontologica Polonica. 2016;61: 15–32.

315.

Mull CG, Houseknecht DW, Bird KJ. Revised Cretaceous and Tertiary stratigraphic nomenclature in the Colville Basin, northern Alaska. US Geological Survey Professional Paper. 2003;1673: 1–51.

316.

Murphy EC, Hoganson JW, Johnson KR. Lithostratigraphy of the Hell Creek Formation in North Dakota. Geological Society of America Special Papers. 2002;361: 9–34. doi:10.1130/0-8137-2361-2.9

317.

Murray GE, Weidie Jr AE, Boyd DR, Forde RH, Lewis Jr PD. Formational divisions of Difunta Group, Parras Basin, Coahuila and Nuevo León, Mexico. AAPG Bulletin. 1962;46: 374–383.

318.

Nambudiri EMV, Binda PL. Paleobotany, palynology and depositional environment of the Maastrichtian Whitemud Formation in Alberta and Saskatchewan, Canada. Cretaceous research. 1991;12: 579–596.

319.

Newman KR. Biostratigraphic correlation of Cretaceous-Tertiary boundary rocks, Colorado to San Juan Basin, New Mexico. Geological Society of America Special Papers. 1987;209: 151–164.

320.

Nichols DJ. Palynostratigraphy in relation to sequence stratigraphy, Straight Cliffs Formation (Upper Cretaceous), Kaiparowits Plateau, Utah. US Geological Survey Bulletin. 1995;2115–B: 1–21.

321.

Nichols DJ. Palynology and ages of some Upper Cretaceous formations in the Markagunt and northwestern Kaiparowits Plateaus, southwestern Utah. US Geological Survey Bulletin. 1997;2153: 1–26.

322.

Nordt L, Atchley S, Dworkin S. Terrestrial evidence for two greenhouse events in the latest Cretaceous. GSA today. 2003;13: 4–9.

323.

Obradovich JD. A Cretaceous time scale. Geological Association of Canada Special Paper. 1993;39: 379–396.

324.

Obradovich JD, Cobban WA. A time-scale for the Late Cretaceous of the Western Interior of North America. Geological Association of Canada Special Paper. 1975;13: 31–54.

325.

Obradovich JD. Geochronology of Laramide synorogenic strata in the Denver Basin, Colorado. Rocky Mountain Geology. 2002;37: 165–171. doi:10.2113/gsrocky.37.2.165

326.

O’Connor PM, Roberts EM, Tapanila L, Deino AL, Hieronymous T, Jinnah ZA. Reconnaissance paleontology in the Straight Cliffs and Wahweap formations (Turonian– Campanian), Grand Staircase–Escalante National Monument, Utah. Advances in Western Interior Late Cretaceous Paleontology and Geology, St George, Utah, Abstracts with Program. 2009. p. 40.

327.

Ogg JG, Agterberg FP, Gradstein FM. The Cretaceous Period. In: Gradstein FM, Ogg JG, Smith AG, editors. A Geologic Time Scale. Cambridge, UK: Cambridge University Press; 2004. pp. 344–383.

328.

Ogg JG, Hinnov LA. Cretaceous. In: Gradstein FM, Ogg JG, Schmitz MD, Ogg G, editors. The Geologic Time Scale. Oxford, UK: Elsevier; 2012. pp. 793–853.

329.

Ogg JG, Smith AG. The geomagnetic polarity time scale. In: Gradstein FM, Ogg JG, Smith AG, editors. A Geologic Time Scale. Cambridge, UK: Cambridge University Press; 2004. pp. 63–86.

330.

Ogg JG. Geomagnetic polarity time scale. In: Gradstein FM, Ogg JG, Schmitz MD, Ogg G, editors. The Geologic Time Scale. Oxford, UK: Elsevier; 2012. pp. 85–113.

331.

Olsen T, Steel R, Hogseth K, Skar T, Roe SL. Sequential architecture in a fluvial succession; sequence stratigraphy in the Upper Cretaceous Mesaverde Group, Prince Canyon, Utah. Journal of Sedimentary Research. 1995;65: 265–280.

332.

Osborn HF. A new genus and species of Ceratopsia from New Mexico, *Pentaceratops sternbergii*. American Museum Novitates. 1923;93: 3.

333.

Ostrom JH. A new species of hadrosaurian dinosaur from the Cretaceous of New Mexico. Journal of Paleontology. 1961;35: 575–577.

334.

Ott CJ. Cranial anatomy and biogeography of the first *Leptoceratops gracilis* (Dinosauria: Ornithischia) specimens from the Hell Creek Formation, southeast Montana. In: Carpenter K, editor. Horns and Beaks Ceratopsian and Ornithopod Dinosaurs. Bloomington, Indiana: Indiana University Press; 2006. pp. 213–233.

335.

Parks WA. Preliminary description of a new species of trachodont dinosaur of the genus *Kritosaurus, Kritosaurus incurvimanus*. Transactions of the Royal Society of Canada, series. 1919;3: 51–59.

336.

Parks WA. The osteology of the trachodont dinosaur *Kritosaurus incurvimanus*. University of Toronto Studies. 1920;11: 5–74.

337.

Parks WA. *Parasaurolophus walkeri*: a new genus and species of crested trachodont dinosaur. University of Toronto Studies. 1922;13: 5–32.

338.

Parks WA. *Arrhinoceratops brachyops*, a new genus and species of Ceratopsia from the Edmonton Formation of Alberta. University of Toronto Studies. 1925;19: 5–15.

339.

Payenberg TH, Braman DR, Davis DW, Miall AD. Litho-and chronostratigraphic relationships of the Santonian-Campanian Milk River Formation in southern Alberta and Eagle Formation in Montana utilising stratigraphy, U-Pb geochronology, and palynology. Canadian Journal of Earth Sciences. 2002;39: 1553–1577.

340.

Penkalski P, Dodson P. The morphology and systematics of *Avaceratops*, a primitive horned dinosaur from the Judith River Formation (Late Campanian) of Montana, with the description of a second skull. Journal of Vertebrate Paleontology. 1999;19: 692–711. doi:10.1080/02724634.1999.10011182

341.

Peterson F. Four new members of the Upper Cretaceous Straight Cliffs Formation in the southeastern Kaiparowits region, Kane County, Utah. US Geological Survey Bulletin. 1969;1274–J: 1–28.

342.

Powell JS. Paleontology and sedimentation models of the Kimbeto Member of the Ojo Alamo Sandstone. Four Corners Geological Society Memoir. 1973;1973: 111–122.

343.

Prieto-Marquez A. Phylogeny and historical biogeography of hadrosaurid dinosaurs. Ph.D dissertation, Florida State University. 2008.

344.

Prieto-Marquez A. The braincase and skull roof of *Gryposaurus notabilis*(Dinosauria, Hadrosauridae), with a taxonomic revision of the genus. Journal of Vertebrate Paleontology. 2010;30: 838–854. doi:10.1080/02724631003762971

345.

Prieto-Márquez A. The skull and appendicular skeleton of *Gryposaurus latidens*, a saurolophine hadrosaurid (Dinosauria: Ornithopoda) from the early Campanian (Cretaceous) of Montana, USA. Can J Earth Sci. 2012;49: 510–532. doi:10.1139/e11-069

346.

Prieto-Márquez A. Skeletal morphology of *Kritosaurus navajovius* (Dinosauria: Hadrosauridae) from the Late Cretaceous of the North American south-west, with an evaluation of the phylogenetic systematics and biogeography of Kritosaurini. Journal of Systematic Palaeontology. 2013;12: 133–175. doi:10.1080/14772019.2013.770417

347.

Ramírez-Velasco AA, Benammi M, Prieto-Márquez A, Ortega JA, Hernández-Rivera R. *Huehuecanauhtlus tiquichensis*, a new hadrosauroid dinosaur (Ornithischia: Ornithopoda) from the Santonian (Late Cretaceous) of Michoacán, Mexico. Can J Earth Sci. 2012;49: 379–395. doi:10.1139/e11-062

348.

Raynolds RG, Johnson KR. Synopsis of the stratigraphy and paleontology of the uppermost Cretaceous and lower Tertiary strata in the Denver Basin, Colorado. Rocky Mountain Geology. 2003;38: 171–181. doi:10.2113/gsrocky.38.1.171

349.

Raynolds RG. Upper Cretaceous and Tertiary stratigraphy of the Denver Basin, Colorado. Rocky Mountain Geology. 2002;37: 111–134. doi:10.2113/gsrocky.37.2.111

350.

Reeside JB. Upper Cretaceous and Tertiary formations of the western part of San Juan Basin, Colorado, and New Mexico. US Geological Survey Professional Paper. 1924;134: 1–70.

351.

Reeside JB. The cephalopods of the Eagle Sandstone and related formations in the Western Interior of the United States. US Geological Survey Professional Paper. 1927;151: 1–87.

352.

Renne PR, Balco G, Ludwig KR, Mundil R, Min K. Response to the comment by WH Schwarz et al. on “Joint determination of 40 K decay constants and 40 Ar∗/40 K for the Fish Canyon sanidine standard, and improved accuracy for 40 Ar/39 Ar geochronology” by PR Renne et al.(2010). Geochimica et Cosmochimica Acta. 2011;75: 5097–5100.

353.

Renne PR, Deino AL, Walter RC, Turrin BD, Swisher CC, Becker TA, et al. Intercalibration of astronomical and radioisotopic time. Geology. 1994;22: 783–786. doi:10.1130/0091-7613(1994)022<0783:IOAART>2.3.CO;2

354.

Renne PR, Mundil R, Balco G, Min K, Ludwig KR. Joint determination of 40K decay constants and 40Ar*/40K for the Fish Canyon sanidine standard, and improved accuracy for 40Ar/39Ar geochronology. Geochimica et Cosmochimica Acta. 2010;74: 5349–5367. doi:10.1016/j.gca.2010.06.017

355.

Renne PR, Swisher CC, Deino AL, Karner DB, Owens TL, DePaolo DJ. Intercalibration of standards, absolute ages and uncertainties in 40Ar/39Ar dating. Chemical Geology. 1998;145: 117–152. doi:10.1016/S0009-2541(97)00159-9

356.

Rivera TA, Storey M, Zeeden C, Hilgen FJ, Kuiper K. A refined astronomically calibrated 40 Ar/39 Ar age for Fish Canyon sanidine. Earth and Planetary Science Letters. 2011;311: 420–426.

357.

Roberts EM, Deino AL, Chan MA. 40Ar/39Ar age of the Kaiparowits Formation, southern Utah, and correlation of contemporaneous Campanian strata and vertebrate faunas along the margin of the Western Interior Basin. Cretaceous Research. 2005;26: 307–318. doi:10.1016/j.cretres.2005.01.002

358.

Roberts EM, Sampson SD, Deino AL, Bowring S, Buchwaldt R. The Kaiparowits Formation: a remarkable record of Late Cretaceous terrestrial environments, ecosystems and evolution in western North America. In: Titus AL, Loewen MA, editors. At the Top of the Grand Staircase: The Late Cretaceous of Southern Utah. Bloomington, Indiana: Indiana University Press; 2013. pp. 85–106.

359.

Roberts EM. Facies architecture and depositional environments of the Upper Cretaceous Kaiparowits Formation, southern Utah. Sedimentary Geology. 2007;197: 207–233. doi:10.1016/j.sedgeo.2006.10.001

360.

Roehler HW. Stratigraphy of the Mesaverde Group in the Central and Eastern Greater Green River Basin, Wyoming, Colorado, and Utah. United States Geological Survey, Professional Paper. 1990;1508: 1–52.

361.

Rogers RR, Brady ME. Origins of microfossil bonebeds: insights from the Upper Cretaceous Judith River Formation of north-central Montana. Paleobiology. 2010;36: 80–112.

362.

Rogers RR, Kidwell SM, Deino AL, Mitchell JP, Nelson K, Thole JT. Age, correlation, and lithostratigraphic revision of the Upper Cretaceous (Campanian) Judith River Formation in its type area (north-central Montana), with a comparison of low-and high-accommodation alluvial records. The Journal of Geology. 2016;124: 99–135.

363.

Rogers RR, Swisher III CC, Horner JR. 40Ar/39Ar age and correlation of the nonmarine Two Medicine Formation (Upper Cretaceous), northwestern Montana, USA. Canadian Journal of Earth Sciences. 1993;30: 1066–1075.

364.

Rogers RR. Marine facies of the Judith River Formation (Campanian) in the type area, north-central Montana. Montana Geological Society: 1993 Field Conference Guidebook: Old Timers’ Rendezvous Edition: Energy and Mineral Resources of Central Montana. 1993; 61–69.

365.

Rogers RR. Nature and origin of through-going discontinuities in nonmarine foreland basin strata, Upper Cretaceous, Montana: implications for sequence analysis. Geology. 1994;22: 1119–1122. doi:10.1130/0091-7613(1994)022<1119:NAOOTG>2.3.CO;2

366.

Rogers RR. Sequence analysis of the Upper Cretaceous Two Medicine and Judith River formations, Montana; nonmarine response to the Claggett and Bearpaw marine cycles. Journal of Sedimentary Research. 1998;68: 615–631. doi:10.2110/jsr.68.604

367.

Rogers RR, Swisher CC. The Claggett and Bearpaw transgressions revisited; new 40Ar/39Ar data and a review of possible drivers. Geological Society of America. 1996;28: 62.

368.

Rowe T, Cifelli RL, Lehman TM, Weil A. The Campanian Terlingua local fauna, with a summary of other vertebrates from the Aguja Formation, Trans-Pecos Texas. Journal of Vertebrate Paleontology. 1992;12: 472–493. doi:10.1080/02724634.1992.10011475

369.

Rowe T, Colbert EH, Nations JD. The occurrence of *Pentaceratops* with a description of its frill. In: Lucas SG, Rigby JK, Kues BS, editors. Advances in San Juan Basin Paleontology. Albuquerque: University of New Mexico Press; 1981. pp. 29–48.

370.

Russell LS. Mammalian faunal succession in the Cretaceous System of western North America. Geological Association of Canada Special Paper. 1975;13: 137–161.

371.

Russell LS. Evidence for an unconformity at the Scollard–Battle contact, Upper Cretaceous strata, Alberta. Can J Earth Sci. 1983;20: 1219–1231. doi:10.1139/e83-109

372.

Russell LS, Landes RW. Geology of the southern Alberta plains. Geological Survey of Canada, Memoir. 1940;221.

373.

Russell LS. Cretaceous non-marine faunas of northwestern North America. Life Sciences Contributions, Royal Ontario Museum. 1964;61: 1–21.

374.

Ryan M, Tanke D, Brinkman D, Eberth D, Currie P. A new *Pachyrhinosaurus*-like ceratopsian from the upper Dinosaur Park Formation (late Campanian) of southern Alberta, Canada. Journal of Vertebrate Paleontology. 2006;26: 117A.

375.

Ryan MJ, Eberth DA, Brinkman DB, Currie PJ, Tanke DH. A new *Pachyrhinosaurus*-like ceratopsid from the upper Dinosaur Park Formation (late Campanian) of southern Alberta, Canada. New Perspectives on Horned Dinosaurs The Royal Tyrrell Museum Ceratopsian Symposium. Bloomington, Indiana: Indiana University Press; 2010. pp. 141–155.

376.

Ryan MJ, Evans DC, Currie PJ, Brown CM, Brinkman D. New leptoceratopsids from the Upper Cretaceous of Alberta, Canada. Cretaceous Research. 2012;35: 69–80. doi:10.1016/j.cretres.2011.11.018

377.

Ryan MJ, Evans DC, Shepherd KM. A new ceratopsid from the Foremost Formation (middle Campanian) of Alberta. Can J Earth Sci. 2012;49: 1251–1262. doi:10.1139/e2012-056

378.

Ryan MJ, Evans DC. Ornithischian dinosaurs. In: Currie PJ, Koppelhus EB, editors. Dinosaur Provincial Park: A spectacular ancient ecosystem revealed. Bloomington, Indiana: Indiana University Press; 2005. pp. 312–348.

379.

Ryan MJ, Holmes R, Russell AP. A revision of the late campanian centrosaurine ceratopsid genus *Styracosaurus* from the Western Interior of North America. Journal of Vertebrate Paleontology. 2007;27: 944–962. doi:10.1671/0272-4634(2007)27[944:AROTLC]2.0.CO;2

380.

Ryan MJ, Russell AP, Hartman S. A new chasmosaurine ceratopsid from the Judith River Formation, Montana. In: Ryan MJ, Chinnery-Allgeier BJ, Eberth DA, editors. New Perspectives on Horned Dinosaurs The Royal Tyrrell Museum Ceratopsian Symposium. Bloomington, Indiana: Indiana University Press; 2010. pp. 181–188.

381.

Ryan MJ, Russell AP. A new centrosaurine ceratopsid from the Oldman Formation of Alberta and its implications for centrosaurine taxonomy and systematics. Canadian Journal of Earth Sciences. 2005;42: 1369–1387.

382.

Ryan MJ. A new basal centrosaurine ceratopsid from the Oldman Formation, southeastern Alberta. Journal of Paleontology. 2007;81: 376–396. doi:10.1666/0022-3360(2005)079[0790:ANESJA]2.0.CO;2

383.

Ryan MJ. Taxonomy, systematics and evolution of centrosaurine ceratopsids of the Campanian Western Interior Basin of North America [Internet]. Ph.D dissertation, University of Calgary. 2003.

384.

Sageman BB, Singer BS, Meyers SR, Siewert SE, Walaszczyk I, Condon DJ, et al. Integrating 40Ar/39Ar, U-Pb, and astronomical clocks in the Cretaceous Niobrara Formation, Western Interior Basin, USA. Geological Society of America Bulletin. 2014;126: 956–973. doi:10.1130/B30929.1

385.

Sahni A. The vertebrate fauna of the Judith River Formation, Montana. Bulletin of the American Museum of Natural History. 1972;147: 325–412.

386.

Sampson SD, Loewen M. Unraveling a radiation: a review of the diversity, stratigraphic distribution, biogeography, and evolution of horned dinosaurs. (Ornithischia : Ceratopsidae). In: Ryan MJ, Chinnery BJ, Eberth DA, editors. New Perspectives on Horned Dinosaurs The Royal Tyrrell Museum Ceratopsian Symposium. Bloomington, Indiana: Indiana University Press; 2010. pp. 405–427.

387.

Sampson SD, Loewen MA, Farke AA, Roberts EM, Forster CA, Smith JA, et al. New horned dinosaurs from Utah provide evidence for intracontinental dinosaur endemism. PLoS ONE. 2010;5: e12292. doi:10.1371/journal.pone.0012292

388.

Sampson SD, Loewen MA. *Tyrannosaurus rex* from the Upper Cretaceous (Maastrichtian) North Horn Formation of Utah: biogeographic and paleoecologic implications. Journal of Vertebrate Paleontology. 2005;25: 469–472. doi:10.1671/0272-4634(2005)025[0469:TRFTUC]2.0.CO;2

389.

Sampson SD, Lund EK, Loewen MA, Farke AA, Clayton KE. A remarkable short-snouted horned dinosaur from the Late Cretaceous (late Campanian) of southern Laramidia. Proceedings of the Royal Society of London B: Biological Sciences. 2013;280: 20131186. doi:10.1098/rspb.2013.1186

390.

Sampson SD. Two new horned dinosaurs from the upper Cretaceous Two Medicine Formation of Montana; with a phylogenetic analysis of the Centrosaurinae (Ornithischia: Ceratopsidae). Journal of Vertebrate Paleontology. 1995;15: 743–760. doi:10.1080/02724634.1995.10011259

391.

Samson SD, Alexander Jr EC. Calibration of the interlaboratory 40Ar/39Ar dating standard, MMhb-1. Chemical Geology: Isotope Geoscience section. 1987;66: 27–34. doi:10.1016/0168-9622(87)90025-X

392.

Sankey JT, Gose WA. Late Cretaceous mammals and magnetostratigraphy, Big Bend, Texas. Occasional Papers of the Museum of Natural Science, Louisiana State University. 2001;77: 1–16.

393.

Sankey JT. Late Campanian southern dinosaurs, Aguja Formation, Big Bend, Texas. Journal of Paleontology. 2001;75: 208–215. doi:10.1017/S0022336000031991

394.

Sankey JT. Turtles of the upper Aguja Formation (late Campanian), Big Bend National Park, Texas. New Mexico Museum of Natural History and Science Bulletin. 2006;35: 235–243.

395.

Sankey JT. Faunal composition and significance of high-diversity, mixed bonebeds containing *Agujaceratops mariscalensis* and other dinosaurs, Aguja Formation (Upper Cretaceous), Big Bend, Texas. New Perspectives on Horned Dinosaurs The Royal Tyrrell Museum Ceratopsian Symposium. 2010; 520–37.

396.

Sarna-Wojcicki AM, Pringle Jr MS. Laser-fusion 40 Ar/39 Ar ages of the Tuff of Taylor Canyon and Bishop Tuff, E. California-W. Nevada. Eos Trans AGU. 1992;73: 43.

397.

Scannella JB, Fowler DW. Anagenesis in *Triceratops*: evidence from a newly resolved stratigraphic framework for the Hell Creek Formation. Cincinnati Museum Center Scientific Contributions. 2009;3: 148–149.

398.

Scannella JB, Fowler DW, Goodwin MB, Horner JR. Evolutionary trends in *Triceratops* from the Hell Creek Formation, Montana. Proceedings of the National Academy of Sciences. 2014;111: 10245–10250.

399.

Scannella JB, Horner JR. *Torosaurus* Marsh, 1891, is *Triceratops* Marsh, 1889 (Ceratopsidae: Chasmosaurinae): synonymy through ontogeny. Journal of Vertebrate Paleontology. 2010;30: 1157–1168. doi:10.1080/02724634.2010.483632

400.

Schiebout JA, Rigsby CA, Rapp SD, Hartnell JA, Standhardt BR. Stratigraphy of the Cretaceous-Tertiary and Paleocene-Eocene transition rocks of Big Bend National Park, Texas. The Journal of Geology. 1987;95: 359–375.

401.

Schmitz MD, Bowring SA. U-Pb zircon and titanite systematics of the Fish Canyon Tuff: an assessment of high-precision U-Pb geochronology and its application to young volcanic rocks. Geochimica et Cosmochimica Acta. 2001;65: 2571–2587. doi:10.1016/S0016-7037(01)00616-0

402.

Schmitz MD. Radiogenic isotope geochronology. In: Gradstein FM, Ogg JG, Schmitz MD, Ogg G, editors. The Geological Time Scale. Oxford, UK: Elsevier; 2012. pp. 115–125.

403.

Schmitz MD. Radiometric Ages used in GTS2012. In: Gradstein FM, Ogg JG, Schmitz MD, Ogg G, editors. The Geological Time Scale. Oxford, UK: Elsevier; 2012. pp. 1045–1082.

404.

Schoene B, Crowley JL, Condon DJ, Schmitz MD, Bowring SA. Reassessing the uranium decay constants for geochronology using ID-TIMS U–Pb data. Geochimica et Cosmochimica Acta. 2006;70: 426–445. doi:10.1016/j.gca.2005.09.007

405.

Schoene B, Samperton KM, Eddy MP, Keller G, Adatte T, Bowring SA, et al. U-Pb geochronology of the Deccan Traps and relation to the end-Cretaceous mass extinction. Science. 2015;347: 182–184. doi:10.1126/science.aaa0118

406.

Schott RK, Evans DC, Williamson TE, Carr TD, Goodwin MB. The anatomy and systematics of *Colepiocephale lambei* (Dinosauria: Pachycephalosauridae). Journal of Vertebrate Paleontology. 2009;29: 771–786.

407.

Schwarz WH, Kossert K, Trieloff M, Hopp J. Comment on the “Joint determination of 40K decay constants and 40Ar∗/40K for the Fish Canyon sanidine standard, and improved accuracy for 40Ar/39Ar geochronology” by Paul R. Renne et al. (2010). Geochimica et Cosmochimica Acta. 2011;75: 5094–5096. doi:10.1016/j.gca.2011.06.022

408.

Shelton JA. Application of sequence stratigraphy to the nonmarine Upper Cretaceous Two Medicine Formation, Willow Creek anticline, northwestern Montana. M.S. thesis, Montana State University. 2007.

409.

Singer BS, Jicha BR, Coe RS, Mochizuki N. An Earthtime chronology for the Matuyama-Brunhes geomagnetic field reversal. AGU Fall Meeting Abstracts. 2012;21.

410.

Soegaard K, Ye H, Halik N, Daniels AT, Arney J, Garrick S. Stratigraphic evolution of latest Cretaceous to early Tertiary Difunta foreland basin in northeast Mexico: Influence of salt withdrawal on tectonically induced subsidence by the Sierra Madre Oriental fold and thrust belt. AAPG Memoir. 2003;79: 364–394.

411.

Spear B, Kirschbaum MA. Re-evaluation of depositional environments of the Neslen Formation from subsurface and surface data, Book Cliffs, Utah. 2012;2012 AAPG Rocky Mountain Section Meeting.

412.

Speiker EM. Late Mesozoic and early Cenozoic history of central Utah. US Geological Survey Professional Paper. 1946;205: 117–161.

413.

Sprain CJ, Renne PR, Wilson GP, Clemens WA. High-resolution chronostratigraphy of the terrestrial Cretaceous-Paleogene transition and recovery interval in the Hell Creek region, Montana. Geological Society of America Bulletin. 2015;127: 393–409. doi:10.1130/B31076.1

414.

Steiger R, Jäger E. Subcommission on geochronology: convention on the use of decay constants in geo- and cosmochronology. Earth and Planetary Science Letters. 1977;36: 359–362.

415.

Sternberg CM. A new hadrosaur from the Oldman Formation of Alberta: discussion of nomenclature. Bulletin of the National Museum of Canada. 1953;28: 1–12.

416.

Sternberg CM. Pachycephalosauridae proposed for dome-headed dinosaurs, *Stegoceras lambei*, n. sp., described. Journal of Paleontology. 1945;19: 534–538.

417.

Sternberg CM. Complete skeleton of *Leptoceratops gracilis* Brown from the upper Edmonton member on Red Deer River, Alberta. National Museum of Canada Bulletin. 1951;123: 225–255.

418.

Sternberg CM. A new species of horned dinosaur from the Upper Cretaceous of Alberta. Bulletin of the National Museum of Canada. 1929;54: 34–37.

419.

Sternberg CM. Hooded hadrosaurs of the Belly River Series of the Upper Cretaceous. Bulletin of the Geological Survey of Canadaa. 1935;52: 1–37.

420.

Sternberg CM. Ceratopsidae from Alberta. Journal of Paleontology. 1940;14: 468–480.

421.

Sternberg CM. *Pachyrhinosaurus canadensis*, representing a new family of the Ceratopsia, from southern Alberta. National Museum of Canada Bulletin. 1950;118: 109–120.

422.

Sues H-D, Galton PM. Anatomy and classification of the North American Pachycephalosauria (Dinosauria: Ornithischia). Palaeontographica A. 1987;198: 1–40.

423.

Sues H-D, Larson DW. Diversity and variation of theropod dinosaur teeth from the uppermost Santonian Milk River Formation (Upper Cretaceous), Alberta: a quantitative method supporting identification of the oldest dinosaur tooth assemblage in Canada. Canadian Journal of Earth Sciences. 2008;45: 1455–1468.

424.

Sullivan RM, Jasinkski SE, Guenther M, Lucas SG. The first lambeosaurin (Dinosauria, Hadrosauridae, Lambeosaurinae) from the Upper Cretaceous Ojo Alamo Formation (Naashoibito Member), San Juan Basin, New Mexico. New Mexico Museum of Natural History and Science Bulletin. 2011;53: 405–417.

425.

Sullivan RM, Williamson TE. A new skull of *Parasaurolophus* (Dinosauria: Hadrosauridae) and a revision of the genus. New Mexico Museum of Natural History and Science. New Mexico Museum of Natural History and Science Bulletin. 1999;15: 52.

426.

Sullivan RM, Bennett GE. A juvenile *Parasaurolophus* (Ornithischia: Hadrosauridae) from the Upper Cretaceous Fruitland Formation of New Mexico. New Mexico Museum of Natural History and Science Bulletin. 2000;17: 215–220.

427.

Sullivan RM, Boere AC, Lucas SG. Redescription of the ceratopsid dinosaur *Torosaurus utahensis* (Gilmore, 1946) and a revision of the genus. Journal of Paleontology. 2005;79: 564–582. doi:10.1666/0022-3360(2005)0792.0.CO;2

428.

Sullivan RM, Lucas SG, Braman DR. Dinosaurs, pollen, and the Cretaceous-Tertiary boundary in the San Juan Basin, New Mexico. New Mexico Geological Society Guidebook. 2005;56: 395–407.

429.

Sullivan RM, Lucas SG. The Kirtlandian, a new land-vertebrate “age” for the Late Cretaceous of western North America. New Mexico Geological Society Guidebook. 2003;54: 369–377.

430.

Sullivan RM, Lucas SG. The Kirtlandian land-vertebrate “age”–faunal composition, temporal position and biostratigraphic correlation in the nonmarine Upper Cretaceous of western North America. New Mexico Museum of Natural History and Science Bulletin. 2006;35: 7–29.

431.

Sullivan RM, Lucas SG. A new Chasmosaurine (Ceratopsidae, Dinosauria) from the Upper Cretaceous Ojo Alamo Formation (Naashoibito Member), San Juan Basin, New Mexico. In: Ryan MJ, Chinnery BJ, Eberth DA, editors. New Perspectives on Horned Dinosaurs. Bloomington, Indiana: Indiana University Press; 2010. pp. 169–180.

432.

Sullivan RM. Revision of the dinosaur *Stegoceras Lambe* (Ornithischia, Pachycephalosauridae). Journal of Vertebrate Paleontology. 2003;23: 181–207.

433.

Sullivan RM. A taxonomic review of the Pachycephalosauridae (Dinosauria: Ornithischia). New Mexico Museum of Natural History and Science Bulletin. 2006;35: 347–365.

434.

Swisher CC, Wang Y, Wang X, Xu X, Wang Y. Cretaceous age for the feathered dinosaurs of Liaoning, China. Nature. 1999;400: 58–61. doi:10.1038/21872

435.

Swisher CC, Wang X, Zhou Z, Wang Y, Jin F, Zhang J, et al. Further support for a Cretaceous age for the feathered-dinosaur beds of Liaoning,China:New 40Ar/39Ar dating of the Yixian and Tuchengzi Formations. ChinSciBull. 2002;47: 136–139. doi:10.1360/02tb9031

436.

Talling PJ, Burbank DW, Lawton TF, Hobbs RS, Lund SP. Magnetostratigraphic chronology of Cretaceous-to-Eocene thrust belt evolution, central Utah, USA. The Journal of Geology. 1994;102: 181–196.

437.

Tanke DH. The 2003 Royal Tyrrell Museum of Palaeontology expedition to the Grande Prairie region (northwestern Alberta, Canada). Alberta Palaeontological Society Bulletin. 200419: 3–31.

438.

Tanke DH. Ceratopsian discoveries and work in Alberta, Canada: a historical review and census. In: Ryan MJ, Chinnery-Allgeier BJ, editors. New Perspectives on Horned Dinosaurs: The Royal Tyrrell Museum Ceratopsian Symposium. Bloomington, Indiana: Indiana University Press; 2010. p. Supp CD.

439.

Tanke DH. Sixty years of pachyrhinosaur (Dinosauria: Ceratopsidae) discoveries in North America. Alberta Paleontological Society, 10th Annual Symposium, Abstract Volume Alberta Paleontological Society, Calgary, Alta. 2006. pp. 33–35.

440.

Thom WT, Dobbin CE. Stratigraphy of Cretaceous-Eocene transition beds in eastern Montana and the Dakotas. Geological Society of America Bulletin. 1924;35: 481–506.

441.

Thomas RG, Eberth DA, Deino AL, Robinson D. Composition, radioisotopic ages, and potential significance of an altered volcanic ash (bentonite) from the Upper Cretaceous Judith River Formation, Dinosaur Provincial Park, southern Alberta, Canada. Cretaceous Research. 1990;11: 125–162. doi:10.1016/S0195-6671(05)80030-8

442.

Thomson TJ, Irmis RB, Loewen MA. First occurrence of a tyrannosaurid dinosaur from the Mesaverde Group (Neslen Formation) of Utah: implications for upper Campanian Laramidian biogeography. Cretaceous Research. 2013;43: 70–79. doi:10.1016/j.cretres.2013.02.006

443.

Titus AL, Roberts EM, Albright LB. Geologic overview. In: Titus AL, Loewen MA, editors. At the top of the Grand Staircase: The Late Cretaceous of Southern Utah. Bloomington, Indiana: Indiana University Press; 2013. pp. 13–41.

444.

Trexler D. Two Medicine Formation, Montana: geology and fauna. In: Tanke DH, Carpenter K, editors. Mesozoic Vertebrate Life. Bloomington, Indiana: Indiana University Press; 2001. pp. 298–309.

445.

Tsujita CJ. Origin of concretion-hosted shell clusters in the Late Cretaceous Bearpaw Formation, southern Alberta, Canada. PALAIOS. 1995;10: 408–423. doi:10.2307/3515044

446.

Tyson H. The structure and relationships of the horned dinosaur *Arrhinoceratops* Parks (Ornithischia: Ceratopsidae). Can J Earth Sci. 1981;18: 1241–1247. doi:10.1139/e81-115

447.

Upchurch P, Barrett PM, Dodson P. Sauropoda. In: Weishampel DB, Osmólska H, Dodson P, editors. The Dinosauria II. University of California Press; 2004. pp. 259–322.

448.

Varricchio DJ, Koeberl C, Raven RF, Wolbach WS, Elsik WC, Miggins DP. Tracing the Manson impact event across the Western Interior Cretaceous Seaway. Geological Society of America Special Papers. 2010;465: 269–299. doi:10.1130/2010.2465(17)

449.

Villeneuve M. Radiogenic Isotope Geochronology. In: Gradstein FM, Ogg G, editors. A Geologic Time Scale 2004. Cambridge, UK: Cambridge University Press; 2004. pp. 87–95.

450.

Waagé KM. The type Fox Hills Formation, Cretaceous (Maestrichtian), South Dakota [Internet]. Peabody Museum of Natural History, Yale University Bulletin; 1968. Available: http://www.getcited.org/pub/101491193

451.

Wagner JR, Lehman TM. An enigmatic new lambeosaurine hadrosaur (Reptilia: Dinosauria) from the Upper Shale member of the Campanian Aguja Formation of Trans-Pecos Texas. Journal of Vertebrate Paleontology. 2009;29: 605–611. doi:10.1671/039.029.0208

452.

Wagner JR. The hadrosaurian dinosaurs (Ornithischia: Hadrosauria) of Big Bend National Park, Brewster County, Texas, with implications for late Cretaceous paleozoogeography [Internet]. M.S. thesis, Texas Tech University. 2001.

453.

Wagoner JCV, Mitchum RM, Campion KM, Rahmanian VD. Siliciclastic sequence stratigraphy in well logs, cores, and outcrops: concepts for high-resolution correlation of time and facies. American Association of Petroleum Geologists, Methods in Exploration. 1990;174: 55.

454.

Wall WP, Galton PM. Notes on pachycephalosaurid dinosaurs (Reptilia: Ornithischia) from North America, with comments on their status as ornithopods. Can J Earth Sci. 1979;16: 1176–1186. doi:10.1139/e79-104

455.

Weimer RJ. Upper Cretaceous stratigraphy, Rocky Mountain area. AAPG Bulletin. 1960;44: 1–20.

456.

Weishampel DB, Jensen JA. <i>Parasaurolophus>/i> (Reptilia: Hadrosauridae) from Utah. Journal of Paleontology. 1979;53: 1422–1427.

457.

Wick SL, Lehman TM. A new ceratopsian dinosaur from the Javelina Formation (Maastrichtian) of west Texas and implications for chasmosaurine phylogeny. Naturwissenschaften. 2013;100: 667–682. doi:10.1007/s00114-013-1063-0

458.

Williamson TE, Carr TD. A new genus of derived pachycephalosaurian from western North America. Journal of Vertebrate Paleontology. 2002;22: 779–801.

459.

Williamson TE, Weil A. Stratigraphic distribution of sauropods in the Upper Cretaceous of the San Juan Basin, New Mexico, with comments on North America’s Cretaceous “sauropod hiatus.” Journal of Vertebrate Paleontology. 2008;28: 1218–1223.

460.

Williamson TE. ?*Brachychampsa sealeyi*, sp nov., (Crocodylia, Alligatoroidea) from the Upper Cretaceous (lower Campanian) Menefee Formation, northwestern New Mexico. Journal of Vertebrate Paleontology. 1996;16: 421–431. doi:10.1080/02724634.1996.10011331

461.

Williamson TE. A new Late Cretaceous (early Campanian) vertebrate fauna from the Allison Member, Menefee Formation, San Juan Basin, New Mexico. New Mexico Museum of Natural History and Science Bulletin. 1997;11: 51–59.

462.

Williamson TE. Review of Hadrosauridae (Dinosauria, Ornithischia) from the San Juan Basin, New Mexico. New Mexico Museum of Natural History and Science Bulletin. 2000;17: 191–213.

463.

Wiman C. *Parasaurolophus tubicen* n. sp. aus der Kreide in New Mexico. Nova Acta Regiae Scientiarum Upsaliensis. 1931;7: 3–11.

464.

Wolfe DG, Kirkland JI. *Zuniceratops christopheri* n. gen. & n. sp., a ceratopsian dinosaur from the Moreno Hill Formation (Cretaceous, Turonian) of west-central New Mexico. Lower and Middle Cretaceous Terrestrial Ecosystems New Mexico Museum of Natural History and Science Bulletin. 1998;14: 303–317.

465.

Wu X, Brinkman DB, Eberth DA, Braman DR. A new ceratopsid dinosaur (Ornithischia) from the uppermost Horseshoe Canyon Formation (upper Maastrichtian), Alberta, Canada. Canadian Journal of Earth Sciences. 2007;44: 1243–1265.

466.

Xing H, Zhao X, Wang K, Li D, Chen S, Mallon JC, et al. Comparative osteology and phylogenetic relationship of *Edmontosaurus* and *Shantungosaurus* (Dinosauria: Hadrosauridae) from the Upper Cretaceous of North America and east Asia. Acta Geologica Sinica - English Edition. 2014;88: 1623–1652. doi:10.1111/1755-6724.12334

467.

Yi M-S, Cross AT. Palynostratigraphy of Upper Cretaceous-Lower Tertiary strata, Price Canyon, Utah. Review of Palaeobotany and Palynology. 1997;97: 53–66. doi:10.1016/S0034-6667(96)00063-2

468.

Zanno LE, Varricchio DJ, O’Connor PM, Titus AL, Knell MJ. A new troodontid theropod, *Talos sampsoni* gen. et sp. nov., from the Upper Cretaceous Western Interior Basin of North America. PloS one. 2011;6: e24487.
