# Supplementary material for: Revised geochronology, correlation, and dinosaur stratigraphic ranges of the Santonian-Maastrichtian (Late Cretaceous) formations of the Western Interior of North America
Source: PLoS One. 2017 Nov 22;12(11):e0188426. doi: 10.1371/journal.pone.0188426 (PMC5699823; doi:10.1371/journal.pone.0188426)
Supplement: S2 Text — This text file provides transcripts of all the pop-up comment boxes featured in the stratigraphic chart (S1 Table). This file should be of use to readers who prefer the text in this larger format. (DOC) [file pone.0188426.s004.doc]

**S2 Text**

**Comment boxes pertaining to stratigraphic chart Fowler v. 1.0.00**

# Instructions for use of this document

This document contains transcriptions of all the pop-up comment boxes that are featured in the stratigraphic chart (S1 Table). This is provided for the convenience of readers who do not wish to use the pop-up comment feature within excel. It should be particularly useful if the chart is printed as a poster.

The comment box transcriptions are arranged in order of the column in which they appear in the chart itself (S1 Table). Each column title (e.g. "ALBERTA") is presented as a level 1 or 2 heading, and subdivided for ease of use into level 3 headings which contain the individual comments.

The reader is advised to use this document with the feature "document map" activated (typically an option within "view" of the file menu system). This displays the arrangement of the comment box transcriptions as different level headings which can be expanded or collapsed to make navigation of the document easier.

**References**

References for this document are the same as for the chart, and can therefore be found in Supporting Information S1 Text.

# Stratigraphy

## STAGE

-----

### STAGE

**Stage boundaries**:

From Ogg & Hinnov (2012)

-----

### Maastrichtian

**Maastrichtian**

Maastrichtian-Palaeogene (K-Pg) boundary at 66.0 +/- 0.2 Ma (2-sigma)

Campanian-Maastrichtian boundary at 72.1 +/- 0.2 Ma (2-sigma)

(Ogg & Hinnov, 2012)

**Previous definition**

Maastrichtian-Palaeogene (K-Pg) boundary at 65.5 +/- 0.3 Ma

Campanian-Maastrichtian boundary at 70.60 +/- 0.6 Ma

(Ogg et al., 2004)

**The Campanian-Maastrichtian boundary -occasional use of previous figure**

In some recent publications (e.g. Sankey, 2006; Longrich & Currie, 2009), 71.3 Ma has been used as the boundary. The 71.3 Ma date is based on previous work (e.g. Gradstein et al., 1994). This probably has little effect on any current interpretation.

-----

### upper

**upper Maastrichtian**

Top: 66.0 Ma

Base: 69.91 Ma

(Ogg & Hinnov, 2012)

The Lower-Upper Maastrichtian boundary is only informally defined (Ogg et al., 2004; Ogg & Hinnov, 2012), but the definition shown here is the appearance of *H. birkelundi* (Landman & Waage, 1993; Cobban, 1993) at 69.91 Ma (Ogg & Hinnov, 2012).

Alternatives suggested for the defintion of the boundary include the base of C31n, exinction of rudist reefs, or inoceramid extinctions (Ogg et al., 2004; Ogg & Hinnov, 2012).

The base of the Upper Maastrichtian (Europe) was correlated with the base of the *H. birkelundi* zone by Machalski et al. (2007).

Previous definition

Top: 65.5 +/- 0.3 Ma

Base: 69.23 Ma

(Ogg et al., 2004)

-----

### lower

**lower Maastrichtian**

Top: 69.23 Ma

Base: 70.6 +/- 0.6 Ma

(Ogg et al., 2004)

Lower-Upper Maastrichtian boundary is only informally defined (Ogg et al., 2004; Ogg & Hinnov, 2012), but the definition shown here is the appearance of *H. birkelundi* (Landman & Waage, 1993; Cobban, 1993). Alternatives include the base of C31n, exinction of rudist reefs, or inoceramid extinctions (Ogg et al., 2004; Ogg & Hinnov, 2012).

-----

### Campanian

**Campanian:**

Campanian-Maastrichtian boundary at 72.1 +/- 0.2 Ma

Santonian-Campanian boundary at 83.6 +/- 0.3 Ma

(Ogg & Hinnov, 2012)

Ogg & Hinnov (2012) place the base of the Campanian at the base of the *Scaphites leei* III ammonite zone.

Ogg et al. (2004) define the base of the Campanian on the extinction of crinoid *Marsupites testudinarius* (provisional boundary marker), which is assumed to be equivalent with the base of *Scaphites leei* III ammonite zone.

The Campanian is informally subdivided into Lower, Middle, and Upper substages in the North American Western Interior (Cobban, 1993; Cobban et al., 2006; see substage text boxes), and into Lower and Upper in northwest Europe (Ogg & Hinnov, 2012). The European Upper/Lower boundary is typically defined as the base of the *Belemnitella mucronata* zone, which projects slightly below the Middle/Lower Campanian boundary in the Western Interior (Ogg & Hinnov, 2012).

Sageman et al. (2014) redefine the Santonian-Campanian boundary as 84.19 ± 0.38 Ma. However, this chart follows the 83.6 Ma boundary date of Ogg & Hinnov (2012), mainly to maintain consistency with other stratigraphic systems defined in GTS 2012.

It is anticipated that a future update to the chart should incorporate the new dates of Sageman et al. (2014).

**Previous definition**

Campanian-Maastrichtian boundary at 70.60 +/- 0.6 Ma

Santonian-Campanian boundary at 85.53 +/- 0.7 Ma

(Ogg et al., 2004)

----

### upper

**upper Campanian**

Ogg & Hinnov (2012) note that the upper Campanian is only informally defined by Cobban (1993; and Cobban et al., 2006) as the time between the appearance of *Didymoceras nebrascense* (76.27 Ma; Ogg & Hinnov, 2012) and the base of the Maastrichtian (see entry; Ogg & Hinnov, 2012, in Gradstein et al., 2012).

Previous definition

Top: 70.6 +/- 0.6 Ma (Ogg & Smith, 2004)

Bottom: 76.38 Ma (Ogg et al., 2004)

----

### middle

middle Campanian

Ogg & Hinnov (2012) note that the middle Campanian is only informally defined by Cobban (1993; and Cobban et al., 2006) as the time between the appearance of Baculites obtusus (lower boundary) and the appearance of *Didymoceras nebrascense* (upper boundary).

Previous range

80.64- 76.38 Ma (Ogg et al., 2004)

----

### lower

**lower Campanian**

Ogg & Hinnov (2012) note that the lower Campanian is only informally defined by Cobban (1993; and Cobban et al., 2006) as the time between the base of the Campanian, and the appearance of *Baculites obtusus*.

Ogg & Hinnov (2012) place the base of the Campanian at the base of the *Scaphites leei* III ammonite zone

Sageman et al. (2014) redefine the Santonian-Campanian boundary as 84.19 ± 0.38 Ma. However, this chart follows the 83.6 Ma boundary date of Ogg & Hinnov (2012), mainly to maintain consistency with other stratigraphic systems defined in GTS 2012.

Previous definition

83.53-80.64 Ma (Ogg et al., 2004)

Upper boundary informally defined by Cobban (1993) as the appearance of *Baculites obtusus*.

Base of the Campanian based on extinction of crinoid *Marsupites testudinarius* (provisional boundary marker). Assumed equivalence with base of *S. leei III* ammonite (Ogg et al., 2004).

----

### Sant.

**Santonian**

Plotted here:

Campanian-Santonian boundary at 83.6 +/- 0.3 Ma (2-sigma)

Santonian-Coniacian boundary at 86.3 +/- 0.5 Ma (2-sigma)

(Ogg & Hinnov, 2012)

Subdivisions of the Santonian are still not formalised (Ogg & Hinnov, 2012). Consequently I am following the ammonite zone based definitions given by Ogg et al. (2004), but using the new range dates of Ogg & Hinnov (2012).

Sageman et al. (2014) redefine the Santonian-Campanian boundary as 84.19 ± 0.38 Ma. However, this chart follows the 83.6 Ma boundary date of Ogg & Hinnov (2012), mainly to maintain consistency with other stratigraphic systems defined in GTS 2012.

Santonian-Campanian boundary based on extinction of crinoid *Marsupites testudinarius* (provisional boundary marker), assumed equivalence with base of *Scaphites leei III* ammonite (Ogg et al., 2004).

Sageman et al. (2014) redefine the Coniacian-Santonian boundary as 86.49 ± 0.44 Ma. However, this chart follows the 86.3 Ma boundary date of Ogg & Hinnov (2012), mainly to maintain consistency with other stratigraphic systems defined in GTS 2012.

It is anticipated that a future update to the chart should incorporate the new dates of Sageman et al. (2014).

Base-Santonian is the lowest occurrence of the widespread inoceramid bivalve *Cladoceramus undulatoplicatus*, equated to the base of the *C. saxitonanus* ammonite zone (Ogg et al., 2004).

Also note alternative/older definitions:

Campanian-Santonian boundary at 84.19 +/- 0.38 Ma (2-sigma)

Santonian-Coniacian boundary at 86.49 +/- 0.44 Ma (2-sigma)

(Sageman et al., 2014)

Campanian-Santonian boundary at 83.53 Ma

Santonian-Coniacian boundary at 85.85 Ma

(Ogg et al., 2004)

----

### ur.

**upper Santonian**

Top: 83.64 Ma base of *Scaphites leei III* ammonite zone.

Base: 84.52 Ma base of *Desmoscaphites erdmanni* zone.

Santonian-Campanian boundary based on extinction of crinoid *Marsupites testudinarius* (provisional boundary marker). Assumed equivalence with base *of Scaphites leei III* ammonite zone (Ogg et al., 2004; Ogg & Hinnov, 2012).

Sageman et al. (2014) redefine the Santonian-Campanian boundary as 84.19 ± 0.38 Ma. However, this chart follows the 83.6 Ma boundary date of Ogg & Hinnov (2012), mainly to maintain consistency with other stratigraphic systems defined in GTS 2012.

The lower boundary is not formalised but is shown by Ogg et al. (2004) and Kauffman et al. (1993) as coincident with the base of the *Desmoscaphites erdmanni* zone (shown here). An alternative definition is given by Cobban (1993) who correlates the lower boundary with the base of the *Clioscaphites choteauensis* zone, which is directly beneath *D. erdmanni*.

----

### mid.

**middle Santonian**

Top: 84.52 Ma base of *Desmoscaphites erdmanni* zone.

Base: 84.94 Ma base of *Clioscaphites vermiformis* zone (see below).

The boundaries are not formalised but the upper boundary is shown by Ogg et al. (2004) and Kauffman et al. (1993) as coincident with the base of the *Desmoscaphites erdmanni* zone (shown here). Cobban (1993) correlates the upper boundary with the base of the *Clioscaphites* *choteauensis* zone, which is directly beneath *D. erdmanni*.

The lower boundary is shown by Ogg et al. (2004), Kauffman et al (1993), and Cobban (1993) as coincident with the base of the *Clioscaphites vermiformis* zone.

----

### lr.

**lower Santonian**

Top: 85.56 Ma base of *Clioscaphites vermiformis* zone.

Base: 86.26 Ma base of *C. saxitonianus* zone.

(Ogg & Hinnov, 2012)

The upper boundary is not formalised but is shown by Ogg et al. (2004), Kauffman et al. (1993), and Cobban (1993) as coincident with the base of the *Clioscaphites vermiformis* zone.

Base-Santonian is the lowest occurrence of the widespread inoceramid bivalve *Cladoceramus undulatoplicatus*, equated to the base of the *C. saxitonanus* ammonite zone (Ogg et al., 2004

Sageman et al. (2014) redefine the Coniacian-Santonian boundary as 86.49 ± 0.44 Ma. However, this chart follows the 86.3 Ma boundary date of Ogg & Hinnov (2012), mainly to maintain consistency with other stratigraphic systems defined in GTS 2012.

----

### Coniacian

**Coniacian**

Coniacian-Santonian boundary at 86.3 +/- 0.5 Ma (2-sigma)

Turonian-Coniacian boundary at 89.8 +/- 0.4 Ma (2-sigma)

(Ogg & Hinnov, 2012)

Sageman et al. (2014) redefine the Coniacian-Santonian boundary as 86.49 ± 0.44 Ma. However, this chart follows the 86.3 Ma boundary date of Ogg & Hinnov (2012), mainly to maintain consistency with other stratigraphic systems defined in GTS 2012.

Bases of the upper, middle, and lower Coniacian are shown by Ogg & Hinnov (2012) as occurring at the bases of the ammonite zones (respectively) *Scaphites depressus*, *S. ventricosus*, and *S. preventricosus*. Ogg & Hinnov (2012) note that the bases of these substages are however based on inoceramid taxa not shown here.

Sageman et al. (2014) redefine the Turonian-Coniacian boundary as 89.75 ± 0.38 Ma. However, this chart follows the 89.8 Ma boundary date of Ogg & Hinnov (2012), mainly to maintain consistency with other stratigraphic systems defined in GTS 2012.

It is anticipated that a future update to the chart should incorporate the new dates of Sageman et al. (2014).

Also see:

Coniacian-Santonian boundary at 86.49 +/- 0.44 Ma (2-sigma)

Turonian-Coniacian boundary at 89.75 +/- 0.38 Ma (2-sigma)

(Sageman et al., 2014)

Coniacian-Santonian boundary at 85.85 Ma

Turonian-Coniacian boundary at 89.27 Ma

(Ogg et al., 2004)

----

### upper

**upper Coniacian**

Top: 86.26 Ma base of *C. saxitonianus* zone.

Base: 87.86 Ma base of *Scaphites depressus* zone

(Ogg & Hinnov, 2012)

Sageman et al. (2014) redefine the Coniacian-Santonian boundary as 86.49 ± 0.44 Ma. However, this chart follows the 86.3 Ma boundary date of Ogg & Hinnov (2012), mainly to maintain consistency with other stratigraphic systems defined in GTS 2012.

----

### m.

**middle Coniacian**

Top: 87.86 Ma base of *Scaphites depressus* zone

Base: 88.77 Ma base of *Scaphites ventricosus* zone

(Ogg & Hinnov, 2012)

----

### lr.

**lower Coniacian**

Top: 88.77 Ma base of *Scaphites ventricosus* ammonite zone

Base: 89.77 Ma base of *Scaphites preventricosus* ammonite zone

(Ogg & Hinnov, 2012)

Sageman et al. (2014) redefine the Turonian-Coniacian boundary as 89.75 ± 0.38 Ma. However, this chart follows the 89.8 Ma boundary date of Ogg & Hinnov (2012), mainly to maintain consistency with other stratigraphic systems defined in GTS 2012.

----

### Turonian

**Turonian**

Turonian-Coniacian boundary at 89.8 +/- 0.4 Ma (2-sigma)

Cenomanian-Turonian boundary at 93.9 +/- 0.2 Ma (2-sigma)

(Ogg & Hinnov, 2012)

Sageman et al. (2014) redefine the Turonian-Coniacian boundary as 89.75 ± 0.38 Ma. However, this chart follows the 89.8 Ma boundary date of Ogg & Hinnov (2012), mainly to maintain consistency with other stratigraphic systems defined in GTS 2012. It is anticipated that a future update to the chart should incorporate the new dates of Sageman et al. (2014).

Note:

Turonian-Coniacian boundary at 89.75 +/- 0.38 Ma (2-sigma)

(Sageman et al., 2014)

Previous definition

Turonian-Coniacian boundary at 89.27 Ma

Cenomanian-Turonian boundary at 93.55 Ma

(Ogg et al., 2004)

----

### ur.

**upper Turonian**

Top: 89.77 Ma base of *Scaphites preventricosus* ammonite zone

Base: 90.65 Ma base of the *Scaphites whitfeldi* ammonite zone

(Ogg & Hinnov, 2012)

Sageman et al. (2014) redefine the Turonian-Coniacian boundary as 89.75 ± 0.38 Ma. However, this chart follows the 89.8 Ma boundary date of Ogg & Hinnov (2012), mainly to maintain consistency with other stratigraphic systems defined in GTS 2012. It is anticipated that a future update to the chart should incorporate the new dates of Sageman et al. (2014).

----

### middle

**middle Turonian**

Top: 90.65 Ma base of the *Scaphites whitfeldi* ammonite zone

Base: 92.9 Ma base of the *Collignoniceras woollgari* ammonite zone

(Ogg & Hinnov, 2012)

The upper boundary of the middle Turonian is not formalized, but Ogg & Hinnov (2012) suggest using the base of *S. whitfeldi* in the Western Interior.

----

### lr.

**lower Turonian**

Top: 92.90 Ma base of the *Collignoniceras woollgari* ammonite zone

Base: 93.90 Ma base of the *Watinoceras devonense* ammonite zone

(Ogg & Hinnov, 2012)

----

## WIS AMMONITE BIOZONES

### WIS AMMONITE BIOZONES

**Ammonite biozones**

Ammonite biozones are based on data provided in Ogg & Hinnov (2012). These might not agree fully with more recent revisions such as Siewert (2011), Meyers et al., (2012), or Sageman et al. (2014), however I have chosen to use Ogg & Hinnov (2012) as it is the standard which is integrated with other stratigraphic methods in GTS 2012.

Given ages represent basal age of the respective ammonite zone (see Ogg & Hinnov, 2012)

----

### *Jeletzkytes nebrascensis*

***Jeletzkytes nebrascensis***

**(=*Discoscaphites nebrascensis*)**

Base = 68.69 Ma

(Ogg & Hinnov, 2012)

Previous dates

Base = 68.33 Ma

(Ogg et al., 2004)

This taxon is not well known in the WIS exposures, but some specimens have been collected from the Fox Hills Fm and other higher units (see later, Landman et al., 2004a). *J.* *nebrascensis* is also known from the *Discoscaphites* *conradi* assemblage zone of Eastern USA (equivalent to the WIS *Hoploscaphites* *nicolleti* and *J.* *nebrascensis* zones combined, and possibly higher: see later) which has yielded dinoflagellates dated reliably as 68.2-67.4 Ma (Landman et al., 2004a, although it should be noted that Landman et al. (2004a) place the K-Pg boundary at 65 Ma, which may influence these dates to be a little younger relatively than the K-Pg of 66 Ma that I use here).

The upper boundary of the *J.* *nebrascensis* zone is poorly constrained, since the late Maastrichtian WIS regression occurs at this time, preserving little marine strata. Fragmentary remains attributed to the taxon are known from the Fox Hills, Hell Creek, and Lance Fms of WY, ND, & SD. This may mean that *J. nebrascensis* extends much later in the Cretaceous than portrayed. However, in Eastern USA sections, while *J*. *nebrascensis* is present alongside other ammonites in the D. conradi zone (68.2-67.4 Ma) it is not present in the overlying *D. minardi* zone (66.4-66 Ma, although see above note on authors placement of K-Pg) or *D. iris* zone (65.6-65 Ma, Landman et al., 2004a). At least some environmental consistency is maintained through these successions since some contemporaries of *J. nebrascensis* survive through the *D. conradi* and *D. minardi* zones. Hence, it is possible that *J. nebrascensis* does not extend upwards beyond its range represented by currently known fossils, and certainly not beyond the *D. conradi* zone (to 67.4 Ma). This has implications for the ages of the Fox Hills, Hell Creek, and Lance Fms (see entries).

**From Landman et al. (2004b, p38-39)**

"In the Western Interior, the D. iris Zone correlates with the dinosaur-bearing strata of the Lance and Hell Creek formations and their equivalents. The confirmed highest occurrence of *Jeletzkytes nebrascensis*, and hence the top of the *J. nebrascensis* Zone, is in the lowermost part of the Hell Creek Formation, South Dakota (Hartman and Kirkland, 2002; Cochran et al., 2003). All of the ammonites above the basal Hell Creek Formation are fragmentary specimens and lie well below the Cretaceous/Tertiary boundary. Hoganson and Murphy (2002) reported a fragment of *Discoscaphites* cf*. D. conradi* or *Jeletzkytes* cf*. J. nebrascensis* from the Breien Member of the Hell Creek Formation in south-central North Dakota, the top of which is 46–61 m below the Hell Creek/Fort Union formational contact. Hartman and Kirkland (2002: 292) reported a fragment of *Hoploscaphites*? from the Fort Rice unit in the middle of the Hell Creek Formation above the Breien Member, and speculated that this specimen was probably the youngest ammonite in the Western Interior. Jeletzky and Clemens (1965) reported a fragment of the early whorls of a scaphite from the Lance Formation approximately 330 m above the top of the Fox Hills Formation in eastern Wyoming. These occurrences of fragmentary scaphites could represent an extension of the *J. nebrascensis* Zone or evidence of a higher, as yet poorly documented zone (Kennedy et al., 1998). However, even these occurrences are probably below the *D. iris* Zone on the Gulf and Atlantic Coastal Plains."

Given the conflicting identification of the Breien Mbr ammonite fragment as either *J. nebrascensis* or *D. conradi*, it would be tentative at best to extend the range of *J.* *nebrascensis* upwards to whichever age the Breien represents.

----

### *Hoploscaphites nicolleti*

***Hoploscaphites nicolleti***

Base = 69.30 Ma

(Ogg & Hinnov, 2012)

Previous dates

Base = 68.78 Ma (Ogg et al, 2004)

In the Fox Hills Fm, SD, the H. nicolleti zone includes *Discoscaphites conradi* (Landman et al., 2004a). In the Eastern US, *D. conradi* forms an assemblage zone equivalent to *H. nicolleti* & *J. nebrascensis* combined.

----

### *H. birkelundi*

***Hoploscaphites birkelundi***

(also spelled "birkelundae", e.g. Landman et al., 2004a)

=aff. *Hoploscaphites nicolleti* (Ogg & Hinnov, 2012)

69.91 Ma (Ogg & Hinnov, 2012)

69.23 Ma (Ogg et al., 2004)

----

### *Baculites clinolobatus*

***Baculites clinolobatus***

70.44 Ma (Ogg & Hinnov, 2012)

69.68 Ma (Ogg et al., 2004).

Ogg et al. (2004) note that their figure is within the 69.42 +/- 0.37 Ma published by Obradovich (1993).

----

### *B. grandis*

***Baculites grandis***

71.13 Ma (Ogg & Hinnov, 2012)

70.11 Ma (Ogg et al., 2004)

----

### *B. baculus*

***Baculites baculus***

Ammonite strat & the Campanian-Maastrichtian boundary

The Campanian-Maastrichtian boundary is marked by the base of the *Scaphites* (*Hoploscaphites*) *constrictus* - *Inoceramus* *fibrosus* zone in Western Canada. This

is equivalent to the base of the *B. baculus* zone in the USA, and the *Belemnella* *lanceolata* zone in Northern Europe (from Lerbekmo & Braman, 2002).

72.05 Ma (Ogg & Hinnov, 2012)

70.56 Ma (Ogg et al., 2004)

----

### *B. eliasi*

***Baculites eliasi***

72.74 Ma (Ogg & Hinnov, 2012)

71.04 Ma (Ogg et al., 2004)

----

### *B. jenseni*

***Baculites jenseni***

73.27 Ma (Ogg & Hinnov, 2012)

71.56 Ma (Ogg et al., 2004)

----

### *B. reesidei*

***Baculites reesidei***

73.63 Ma (Ogg & Hinnov, 2012)

72.14 Ma (Ogg et al., 2004)

The upper part of the *B. reesidei* zone was dated by Baadsgaard et al. (1993) who recovered a date of 72.47 +/- 0.23 Ma, which I have recalibrated to 73.41 +/- 0.23 Ma (the same recalibrated date was reported by Schmitz, 2012b).

However, another radiometric date for the *B. reesidei* zone does not fit with that of Baadsgard et al. (1993). Hicks et al. (1999) published an Ar-Ar date of 72.02 Ma, which I recalibrated to 72.47 Ma. No error is given, but the date is based on 2 sanidine crystals (72.15 +/- 0.33 Ma; 71.92 +/- 0.35 Ma). This 72.47 Ma date falls outside of the range for *B. reesidei* given by Ogg & Hinnov (2012). It is noted that if this 72.47 Ma recalibrated date was correct, then this would cause overlap with the definition of the overlying *B. jenseni* and *B. eliasi* zones (as defined by Ogg & Hinnov, 2012).

----

### *B. cuneatus*

***Baculites cuneatus***

73.91 Ma (Ogg & Hinnov, 2012)

72.78 Ma (Ogg et al., 2004)

----

### *B. compressus*

***Baculites compressus***

74.21 Ma (Ogg & Hinnov, 2012)

One problem with the placement of Ogg & Hinnov (2012) is that it suggests that the entire *B. compressus* zone should be of reversed magnetic polarity, residing within C32r.2r. However, *B. compressus* occurs in a mostly normal polarity zone designated as C33n.1n to C33n.2n, with only a short reversed zone near the top (C33n.1r; Lerbekmo & Braman, 2002; Lerbekmo & Lehtola, 2011). Lerbekmo & Braman (2002) do suggest, however, that this short reversal that they identify as C33n.1r might be the same as a reversed interval within *B. compressus* identified by Fassett & Steiner (1997) in New Mexico, but named as C32r. Hence the C32r.2r subchron defined by Ogg (2012) may be the same reversal identified by Lerbekmo & Braman as C33n.1r. Given that *B. compressus* should be within a mostly normal polarity subchron, the magnetostratigraphic arrangement of Lerbekmo & Braman (2012) seems most consistent.

Old date

73.50 Ma (Ogg et al., 2004)

Ogg et al. (2004) state that their date falls within 73.35 +/- 0.39 Ma (Obradovich, 1993).

----

### *Didymoceras cheyennense*

***Didymoceras cheyennense***

74.60 Ma (Ogg & Hinnov, 2012)

74.28 Ma (Ogg et al., 2004)

----

### *Exiteloceras jenneyi*

***Exiteloceras jenneyi***

75.08 Ma (Ogg & Hinnov, 2012)

75.05 Ma (Ogg et al., 2004). Ogg et al. (2004) state that their number falls within 74.76 +/- 0.45 Ma (Obradovich, 1993).

They also note that this zone is correlative with the C32r/C33n boundary, although this does not seem to agree with Lerbekmo & Braman (2002) who show this boundary occurring between the *B. cuneatus* and *B. compressus* zones.

----

### *D. stevensoni*

***Didymoceras stevensoni***

75.64 Ma (Ogg & Hinnov, 2012)

75.74 Ma (Ogg et al., 2004)

----

### *D. nebrascense*

***Didymoceras nebrascense***

76.27 Ma (Ogg & Hinnov, 2012)

76.38 Ma (Ogg et al., 2004). Ogg et al. (2004) state that their date falls within 75.89 +/- 0.72 Ma (Obradovich, 1993)

----

### *B. scotti*

***Baculites scotti***

76.94 Ma (Ogg & Hinnov, 2012)

77.00 Ma (Ogg et al., 2004)

----

### *B. reduncus*

***Baculites reduncus***

77.63 Ma (Ogg & Hinnov, 2012)

not mentioned in (Ogg et al., 2004)

----

### *B. gregoryensis*

***Baculites gregoryensis***

78.34 Ma (Ogg & Hinnov, 2012)

77.59 Ma (Ogg et al., 2004)

----

### *(B. gilberti)*

***Baculites gilberti***

Not mentioned in Ogg & Hinnov (2012)

78.68 Ma (Ogg et al., 2004)

----

### *B. perplexus*

***Baculites perplexus***

79.01 Ma (Ogg & Hinnov, 2012)

See below (Ogg et al., 2004)

In Ogg et al., (2004) the *B. perplexus* zone comprises 2 morphs of *B. perplexus*, which are split by *B. gilberti*:

*B. perplexus* (late): base = 78.15 Ma

*B. gilberti*: base = 78.68 Ma

*B. perplexus* (early): base = 79.16 Ma

(Ogg et al., 2004)

----

### *B.* sp. (smooth)

***Baculites* sp. (smooth)**

*B*. sp (smooth) 79.64 Ma (Ogg & Hinnov, 2012)

*B*. sp (smooth) 79.61 Ma (Ogg et al., 2004)

----

### *B. asperiformes*

***Baculites asperiformes***

80.21 Ma (Ogg & Hinnov, 2012)

80.00 Ma (Ogg et al., 2004)

----

### *B. maclearni*

***Baculites maclearni***

80.67 Ma (Ogg & Hinnov, 2012)

80.35 Ma (Ogg et al., 2004)

----

### *B. obtusus*

***Baculites obtusus***

80.97 Ma (Ogg & Hinnov, 2012)

80.64 Ma (Ogg et al., 2004).

Ogg et al. (2004) state that their figure falls within 80.54 +/- 0.55 Ma (Obradovich, 1993).

----

### *B.* sp. (weak flanking ribs)

***Baculites* sp. (weak flanking ribs)**

*B.* sp (weak flank ribs) 81.13 Ma (Ogg & Hinnov, 2012)

*B.* sp (weak flank ribs) 80.91 Ma (Ogg et al., 2004)

----

### *B*. sp. (smooth)

***Baculites* sp. (smooth)**

*B.* sp (smooth) 81.28 Ma (Ogg & Hinnov, 2012)

*B*. sp (smooth) 81.22 Ma (Ogg et al., 2004)

----

### *Scaphites hippocrepis III*

***Scaphites hippocrepis III***

81.53 Ma (Ogg & Hinnov, 2012)

81.63 Ma (Ogg et al., 2004)

----

### *S. hippocrepis II*

***Scaphites hippocrepis II***

82.00 Ma (Ogg & Hinnov, 2012)

82.29 Ma (Ogg et al., 2004).

Ogg et al. (2004) note that their date falls within 81.71 +/- 0.34 Ma (Obradovich, 1993)

83.4 Ma date for *Scaphites hippocrepis II*: Rogers et al. (1993), citing Gill et al., 1972; and Gill & Cobban (1973).

----

### *S. hippocrepis I*

***Scaphites hippocrepis I***

82.70 Ma (Ogg & Hinnov, 2012)

82.89 Ma (Ogg et al., 2004)

----

### *S. leei III*

***Scaphites leei III***

83.64 Ma given in Ogg & Hinnov (2012), who cite Siewert (2011) as having given 84.64 +/- 0.23 Ma, and the alternative 83.75 +/- 0.11 Ma from Siewert et al. (in press).

83.53 Ma (Ogg et al., 2004)

----

### *Desmoscaphites bassleri*

***Desmoscaphites bassleri***

84.08 Ma (Ogg & Hinnov, 2012)

83.99 Ma (Ogg et al., 2004)

Ogg et al. (2004) state that their date falls within 83.91 +/- 0.43 Ma and 84.09 +/- 0.40 Ma (Obradovich, 1993).

----

### *Clioscaphites choteauensis*

***Clioscaphites choteauensis***

85.23 Ma (Ogg & Hinnov, 2012)

84.62 Ma (Ogg et al., 2004)

----

### *C. vermiformis*

***Clioscaphites vermiformis***

85.56 Ma (Ogg & Hinnov, 2012)

84.94 Ma (Ogg et al., 2004)

----

### *C. saxitonianus*

***Clioscaphites saxitonianus***

86.26 Ma cited by Ogg & Hinnov (2012) who give the source as 86.26 +/- 0.45 Ma by Siewert (2011), or alternatively 86.35 +/- 0.11 Ma by Siewert et al. (in press).

85.85 Ma (Ogg et al., 2004)

----

### *S. depressus*

***Scaphites depressus-Protexanites bourgeoisianus***

87.86 Ma (Ogg & Hinnov, 2012)

86.96 Ma (Ogg et al., 2004)

Ogg et al. (2004) state that their date falls within 86.92 +/- 0.39 Ma "within the lower third."

----

### *S. ventricosus*

***Scaphites ventricosus***

88.77 Ma (Ogg & Hinnov, 2012)

87.88 Ma (Ogg et al., 2004)

----

### *S. preventricosus*

***Scaphites preventricosus***

89.77 Ma (Ogg & Hinnov, 2012)

88.58 Ma (Ogg et al., 2004)

Ogg et al. (2004) state that their date falls within 88.34 +/- 0.60 Ma.

Formerly "*Forresteria allaudi - S. preventricosus*" zone (Ogg & Hinnov, 2012).

----

### *S. mariasensis*

***Scaphites mariasensis***

89.87 Ma (Ogg & Hinnov, 2012)

89.07 Ma (Ogg et al., 2004)

Formerly *Forresteria peruuana* (Ogg & Hinnov, 2012).

----

### *Prionocyclus germari*

***Prionocyclus germari***

89.98 Ma (Ogg & Hinnov, 2012)

89.40 Ma (Ogg et al., 2004)

*Prionocyclus quadratus* occupies the uppermost Turonian in Cobban (1993).

----

### *S. nigricollensis*

***Scaphites nigricollensis***

90.24 Ma (Ogg & Hinnov, 2012)

89.63 Ma (Ogg et al., 2004)

----

### *S. whitfieldi*

***Scaphites whitfieldi***

90.65 Ma (Ogg & Hinnov, 2012)

89.79 Ma (Ogg et al., 2004)

----

### *S. ferronensis* & *S. warreni*

***Scaphites ferronensis* & *Scaphites warreni***

*S. ferronensis*: 91.08 Ma

*S. warreni*: 91.34 Ma

(Ogg & Hinnov, 2012)

*S. ferronensis*: 89.96 Ma

*S. warreni*: 90.17 Ma

(Ogg et al., 2004)

----

### *P. macombi*

***Prionocyclus macombi***

91.41 Ma (Ogg & Hinnov, 2012)

90.48 Ma (Ogg et al., 2004)

Ogg et al. (2004) state 90.21 +/- 0.72 Ma is within this zone.

*Prionocyclus wyomingensis* shown as above *P. macombi* (Cobban, 1993).

----

### *P. hyatti*

***Prionocyclus hyatti***

91.60 Ma (Ogg & Hinnov, 2012)

90.94 Ma (Ogg et al., 2004)

Ogg et al. (2004) state: 90.51 +/- 0.45 Ma is within this zone; assigned to middle.

----

### *Collignoniceras praecox*

***Collignoniceras praecox***

92.08 Ma (Ogg & Hinnov, 2012)

91.51 Ma (Ogg et al., 2004)

Formerly *Prionocyclus percarinatus* (Ogg & Hinnov, 2012).

----

### *C. woollgari*

***Collignoniceras woollgari***

92.90 Ma (Ogg & Hinnov, 2012)

92.13 Ma (Ogg et al., 2004)

----

### *Mammites nodosoides*

***Mammites nodosoides***

93.35 Ma (Ogg & Hinnov, 2012)

92.70 Ma (Ogg et al., 2004)

----

### *Vascoceras birchbyi*

***Vascoceras birchbyi***

93.45 Ma (Ogg & Hinnov, 2012)

93.15 Ma (Ogg et al., 2004)

Ogg et al. (2004) state: 93.40 +/- 0.63 Ma is within this zone; 92.98 Ma assigned to base.

----

### *Pseudaspidoceras fexuosum*

***Pseudaspidoceras fexuosum***

93.55 Ma (Ogg & Hinnov, 2012)

93.41 Ma (Ogg et al., 2004)

Ogg et al. (2004) state: 93.25 +/- 0.55 Ma is within this zone; 93.33 Ma assigned to base.

----

### *Watinoceras devonense*

***Watinoceras devonense***

93.90 Ma (Ogg & Hinnov, 2012)

93.55 Ma (Ogg et al., 2004)

----

## radiometric & ammonite inconsistency

**Inconsistency between ammonite biozones and radiometric dates**

The revised dates for ammonite biozones provided by Ogg & Hinnov (2012) fail to resolve irreconcilable differences with radiometric dates and magnetostratigraphy from Southern Alberta (Lerbekmo & Braman, 2002; 2005; Eberth, 2005). This appears to be due to radiometric dates chosen for the spline-fit used to calculate the new biozone boundary ages in the Geological Time Scale 2012 (Gradstein et al., 2012), but in some cases was already a problem that existed GTS 2004.

A radiometric date of 75.46 +/- 0.24 Ma (recalibrated from 74.8 Ma; Eberth, 2005; see individual entry) was acquired from a horizon 8m above the base of the Bearpaw Shale in southern Alberta, reportedly within the *B. compressus* zone (Tsujita, 1995; Lerbekmo & Braman, 2002; Eberth, 2005). However, the *B. compressus* zone is defined as 74.21 - 73.91 Ma (Ogg & Hinnov, 2012), i.e. much younger than the radiometric date. This problem existed in the GTS 2004 as the previous age given for the base of the *B. compressus* zone is 73.50 Ma (Ogg, 2004), i.e. younger than the previous 74.8 Ma Bearpaw date.

These issues may have occurred due to a change in the choice of radiometric dates used in the cubic spline-fit methodology of GTS 2012 (Gradstein et al., 2012), compared to its previous incarnation (Gradstein et al., 2004; see second tab in this file), which was dominated by ages published by Obradovich (1993). Perhaps most significant is the inclusion of a date (73.41 Ma; Baadsgaard et al., 1993) for the uppermost part of the *B. reesidei* zone, whereas previously no radiometric date was used, and the *B. reesidei* / *B. jenseni* boundary was positioned relatively higher. This has the effect of condensing the *B. reesidei*, *B. cuneatus*, and *B.compressus* zones into a ~1 million year timespan. The inclusion in GTS 2012 of dates from Hicks et al. (1999) also squeezes this zone from beneath as the Hicks et al. (1999) recalibrated date for the *E. jenneyi* zone (74.85 +/- 0.43 Ma; Schmidt, 2012) is roughly the same as the unrecalibrated date 74.81 +/- 0.45 Ma, given by Obradovich (1993) and used in GTS 2004, effectively.

It is not clear how to reconcile these issues. Resampling and reanalysis of all historical radiometric dates (rather than just recalibration, as performed here) is desirable, and this is being undertaken (D. Eberth pers. comm. to DF, 2014).

## Radiometric dating

### Notes on Ar/Ar dating

**40Ar / 39Ar dating**

Detailed reviews of Ar / Ar dating have been published elsewhere (e.g. McDougall & Harrison, 1999). Notes given here are for the purpose of aiding the reader in understanding the calculation of radiometric dates reported in this chart, how Ar-Ar dates are affected by changing standards and decay constants, and comparability of radiometric dates recovered by different methods (e.g. Ar-Ar vs U-Pb).

**Standards (neutron fluence monitor)**

As 40Ar / 39Ar dating is a relative dating method, every unknown sample needs to be analysed alongside a sample of known age: a standard. Primary standards are minerals from specific rock samples that have been directly dated by K-Ar dating or another method; whereas secondary standards are based on 40Ar / 39Ar intercalibration with a primary standard (Renne et al., 1998). The following list includes (but is not limited to) some of the more popular standards that have been used historically (see McDougall & Harrison, 1999, for a more complete list):

MMhb-1 McClure Mountain hornblende, primary standard: ~520 Ma

GA-1550 Biotite, monazite, NSW, Australia, primary standard: ~98 Ma

TCR Taylor Creek Rhyolite (or sanidine, TCs), secondary standard: ~28 Ma

FCT Fish Canyon Tuff (or sandine, FCs), secondary standard: ~28 Ma

ACR Alder Creek Rhyolite (or sanidine, ACs), secondary or tertiary standard: ~1 Ma

Standards are chosen depending on availability, and should be of comparable age to the unknown sample (Renne et al., 1998). Hence, for Late Cretaceous deposits, usually the secondary standards TCR or FCT are used, typically themselves being calibrated against a primary standard (historically, the MMhb-1 is commonly used, although this depends on the preference of the particular laboratory). Many historically popular standards are no longer used as repeated calibration studies have found the original sample to give inconsistent dates; for example, Baksi et al. (1996) found the widely used MMhb-1 primary standard to be inhomogenous, making its use as a standard no longer tenable. Further, intercalibration studies have continually honed and refined the ages of standards (especially the more widely used secondary standards), with the result that radiometric dates published years apart are typically not precisely comparable without recalibration.

**Decay constants**

The Ar / Ar method depends upon the β- decay of 40K to 40Ca (λβ), and electron capture or β+ of 40K to 40Ar (λε), which combined are referred to as λT or λtotal (Beckinsale & Gale, 1969). The value of the decay constant λT (and its components) have historically been subject to fewer changes than the standards listed above, but have come under increased scrutiny since the late 1990's. It is also notable that different values of λT have been used historically by geochronologists compared to physicists and chemists (see decay constant note).

**Reporting of error**

When reporting error, it is important to note the number of standard deviations (σ, typically 1 or 2). Care must be taken to note when error is given in standard error (SE) rather than standard deviations (σ); this is rare (e.g. Rogers et al., 1993), but can lead to errors being compared that are not strictly comparable (e.g. Roberts et al., 2013; table 6.1). It can also be useful (where possible) to specify whether the reported error is only the "internal error" (which is typically reported), or whether it also includes error in the decay constant.

**Recalibration & current standards**

In order to compare Ar / Ar dates, it is essential to ensure that the same standards and decay constants were used in their calculation, which may require recalibration. If the standards used are different, for example, if an old analysis used the TCR standard, and a more recent one used the FCT, then it will be necessary to find what the equivalent FCT value was to the TCR used in the original analysis. Equivalent values are discussed in the relevant note on this chart. The decay constant absolute value has only a small effect on the absolute age of a sample, but decay constants contribute a greater amount to the error of a radiometric date.

There are two currently prominently used pairings of standard and decay constant:

Kuiper et al. (2008) combined an FCT standard age of 28.201 +/-0.023 Ma, with the decay constant of Min et al. (2000), λT = 5.463 +/- 0.214 E-10/y

Renne et al. (2011) use an FCT standard age of 28.294 +/- 0.036 Ma, with a λT of 5.5305 E-10/y.

This chart is calibrated to the Kuiper et al. (2008) standard, paired with the Min et al. (2000) decay constant. This is not a judgment on the reliability of one method over another; rather it is out of convenience, since the various ammonite biozones and magnetochrons detailed in The Geological Time Scale 2012 (Gradstein et al., 2012; upon which this chart is based) use the Kuiper et al. (2008) FCT standard, and Min et al. (2000) decay constant.

**Agreement with U-Pb dates**

Ar / Ar dates have historically tended to be younger than U-Pb dates by about 1% (Schoene et al., 2006), equating to ~750 ky difference in a 75 m.y. old sample. Possible explanations include longer zircon magma residence times prior to an eruption (Villeneuve, 2004; GTS 2004, p89), error in the potassium-40 decay constant (Schmitz & Bowring, 2001), interlaboratory bias and geological complexities (Kuiper et al., 2008). Recent revisions of standards and decay constants for Ar / Ar dating have closed the gap to within ~0.3% (Kuiper et al., 2008; Renne et al., 2011). Kuiper et al. (2008) consequently state that Ar / Ar dating has improved "absolute uncertainty from ~2.5% to 0.25%".

----

### McClure Mountain hornblende (MMhb-1) standard

**McClure Mountain hornblende (MMhb-1) standard**

A historically important and widely used primary standard, MMhb-1 was found to be too heterogeneous to be reliably used as a primary standard.

**Alexander et al. (1978)** introduce MMhb-1 as 519.5 +/- 2.5 Ma (1σ)

**Samson & Alexander (1987)** revise MMhb-1 to 520.4 +/- 1.7 Ma (1σ).

**Baksi et al. (1996)** reviewed the MMhb-1 standard and concluded that it was too heterogeneous to be used as a primary standard. Since this time, use of and reference to MMhb-1 has declined, but it remains an important historical standard.

**Renne et al. (1998)** performed an intercalibration study which recovered the MMhb-1 at 523.1 +/- 2.6 Ma (1σ; ignoring decay constant error).

----

### Fish Canyon Tuff (FCT) standard

**Fish Canyon Tuff (FCT) standard**

Sometimes also referred to as the Fish Canyon sanidine (FCs).

**Cebula et al. (1986)** first proposed Fish Canyon Tuff (FCT) as a standard, with a value of 27.79 Ma (relative to 518.9 Ma for the McClure Mountain hornblende (MMhb-1; Alexander et al., 1978).

**Samson & Alexander (1987)** performed an intercalibration analysis which changed MMhb-1 to 520.4 +/- 1.7 Ma, which altered the FCT to 27.84 Ma (Renne et al., 1998; although note that in the print article Samson & Alexander, 1987, give the age as 27.9 +/- 0.6 Ma).

**Renne et al. (1994)** perform an intercalibration analysis and recover a FCT of 27.95 +/- 0.18 Ma, equivalent to Mmhb-1 of 522.5 Ma.

**Renne et al. (1998)**

FCT = 28.02 +/- 0.28 Ma (including decay constant error), +/- ; TCR = 28.34 +/- 0.16 Ma; MMhb-1 = 523.1 +/- 2.6 Ma.

**Kuiper et al. (2008)** used orbital tuning to calculate the FCT at 28.201 +/-0.046 Ma (2 sigma).

**Renne et al. (2010)**

FCT = 28.305 +/- 0.031 Ma (see note).

**Renne et al. (2011)**

FCT = 28.294 +/- 0.036 Ma (see note).

**Current usage**

Rivera et al. (2011), Meyers et al. (2012), Singer et al. (2012), and Sageman et al. (2014) all found independent support for Kuiper et al. (2008)'s 28.201 Ma age for the Fish Canyon Sanidine (and therefore rejected Renne et al.'s (2010) further revised 28.3 Ma standard as too old). These three analyses also used three methods (Ar / Ar, U-Pb, cyclostratigraphy) to reach consensus, confirming alignment of U-Pb and Ar / Ar dates.

This chart is calibrated to the Kuiper et al. (2008) standard of 28.201 Ma, which is convenient as this therefore allows use of the GTS 2012 system which also used this figure.

----

### Taylor Creek Rhyolite (TCR) standard

**Taylor Creek Rhyolite (TCR) standard**

The Taylor Creek Rhyolite of New Mexico was initially used as an intralaboratory standard at the USGS in Menlo Park, CA, and was later adopted by numerous labs internationally (Renne et al., 1998).

**Duffield & Dalrymple (1990)** propose TCR as a standard at 27.92 +/- 0.04 Ma, based on analysis of TCR sanidine alongside primary standard SB-3 at 162.9 +/- 0.8 Ma. Samples of MMhb-1 and FCT were run simultaneously with retrieved ages of 519.10 Ma and 27.73 Ma, respectively.

**Obradovich (1990 until at least 2002).**

Recalibration of radiometric dates from analyses by Obradovich conducted in the 1990's (and possibly early 2000's) requires special caution due to the particular methodology of Obradovich during this time. Hicks et al. (2002, p.43) state:

"The TCR (Duffield & Dalrymple, 1990) has been used exclusively since 1990 by one of us (Obradovich) with an assigned age of 28.32 Ma normalized to an age of 520.4 Ma for MMhb-1 (Samson & Alexander, 1987). This age differs from that of 27.92 Ma assigned by Sarna-Wojcicki and Pringle (1992). The choice of 28.32 Ma was entirely pragmatic because this monitor age provided the best comparison with ages delivered by Obradovich and Cobban (1975). In an intercalibration study [...] Renne et al. (1998) obtained ages of 28.34 Ma for TCR and 28.02 Ma for FCT when calibrated against GA1550 biotite as their primary standard with an age of 98.79 Ma. This value of 28.02 agrees quite well with [..] 28.03 Ma obtained through calibration based on the astronomical time scale (Renne et al., 1994). On the basis of unpublished data, one of us (Obradovich) obtained an age of 28.03 Ma for the FCT [...] of W, McIntosh (Geoscience Dept. NM Institute of Mining and Technology, Socorro), calibrated against an age of 28.32 Ma for TCR."

However, note that Obradovich-published analyses from this time do not exclusively use the TCR at 28.32 Ma, as Izzett and Obradovich (1994) state that they use FCT sanidine at 27.55 Ma, and TCR sanidine at 27.92 Ma, both relative to MMhb-1 at 513.9 Ma (in conjunction with λT = 5.543 E-10/y). They note that the 513.9 Ma age of MMhb-1 differs from the then standardized age of 520.4 Ma (Samson & Alexander, 1987) as the former age was calibrated in the lab where their current samples were analysed (Lanphere et al., 1990; Dalrymple et al., 1993).

This creates a problem when recalibrating Ar-Ar ages that used TCR as the fluence monitor (standard). The "official" TCR age of 27.92 Ma has a corresponding FCT age of 27.84 Ma (Samson & Alexander, 1987; Renne et al., 1998). However, since most analyses by Obradovich use TCR at 28.32 Ma, then the question remains as to what number to use for the equivalent FCT when performing recalibrations. Renne et al. (1998) provide an intercalibration factor for FCT : TCR of 1 : 1.00112 +/- 0.0010, which simply calculated is FCT = 28.32 / 1.100112 = 28.006 Ma. This agrees well with the calculated FCT equivalent of 28.03 Ma (Hicks et al., 2002; above; Obradovich, 2002) and a value of 28.02 Ma of Renne et al. (1998). In the Geological Time Scale 2012 (Gradstein et al., 2012), Schmitz (2012) recalibrates dates from Obradovich (1993), and Hicks et al. (1995, 1999) using a legacy FCT age of 28.00 Ma (not stated, but retrocalculated by DF). Sageman et al. (2014; cited as Siewert et al., in press, by Schmitz, 2012b) recalibrate Obradovich's older dates using a legacy FCT age of 28.02 Ma (thereby agreeing with Renne et al., 1998).

In this analysis, when recalibrating an Ar-Ar date that was calculated by Obradovich using a TCR = 28.32, I will use an FCT value of 28.03, as this is the equivalent FCT explicitly stated by Obradovich (2002). This is a very close value to 28.02 (Renne et al., 1998; where the TCR equivalent is 28.34 +/- 0.16 Ma; 1σ, ignoring decay error) so confusion between the two should be avoided, although the difference between ages calculated using 28.03 or 28.02 Ma standards would correspond to only 0.02 to 0.04 m.y. for ages in the Late Cretaceous (100.5 - 66 Ma; Ogg & Hinnov, 2012)

**Renne et al. (1998) p**erformed an intercalibration analysis and recovered the TCR (sanidine) as 28.34 Ma with an error (1σ) of +/- 0.16 Ma (ignoring decay constant error), or +/- 0.28 Ma (including decay constant error).

----

### Decay constant (λT)

**Potassium (40K) decay constant**

The total decay constant (λT) for 40K is given by the product of λβ + λε, where λβ is the probability of β- decay of 40K to 40Ca, and λε is the probability of electron capture or β+ of 40Kto 40Ar (Beckinsale & Gale, 1969).

The currently (2014) accepted standard is 5.463 E-10/y (Min et al., 2000), although alternatives are available, and refinement of this figure is the subject of active research (see below).

The decay constant used for an analysis is not always reported, although it has much less effect on the final calculated age than variations in fluence monitor mineral ages. For example, the difference between using 5.543 E-10/y (Steiger & Jaeger, 1977) and 5.463 E-10/y (Min et al., 2000) is 0.02%, equating to a difference of 0.013 Ma for a sample from the Late Campanian (~75 Ma). However, the reported error for a given date is more strongly affected by the error of the decay constant used.

**HISTORY**

**Beckinsale & Gale (1969)** proposed a 40K decay constant (λT) of 5.480 E-10/y.

**Endt & Van der Leun (1973)** recalculated a λT of 5.428 E-10/y. This is not widely used among geochronologists, although is more commonly used by nuclear physicists, even as late as 2002 (Renne et al., 1998; Kwon et al., 2002).

**Steiger & Jaeger (1977)** revised the Beckinsale & Gale (1969) data to calculate a λT of 5.543 +/- 0.010 E-10/y. This value was standard for geochronologists up until the 2009 vote by geochronologists attending the Earthtime IV meeting, whereupon it was agreed to adopt the 5.463 +/- 0.214 E-10/y of Min et al. (2000).

**Renne et al. (1998)** state [my edits]: "It is noteworthy that values of the decay constants recommended by Steiger and Jaeger (1977) [λ = 5.543 +/- 0.010 E-10/y] are at odds with values used since at least 1990 by the nuclear physics and chemistry communities."; Renne et al. then state that Endt (1990) uses a λ of 5.428 +/- 0.032 x10^-10^/y, which "is more than 2% different from the values recommended by Steiger and Jaeger (1977)". Thus, there is no absolute guarantee that a lab that performed an Ar / Ar analysis in the 1990's will be using the λT of 5.543 E-10/y of Steiger and Jaeger (1977). It is notable that the λT of Endt (1990) is actually lower than the currently (2014) used λT of 5.463 +/- 0.214 Ma E-10/y (Min et al., 2000).

**Min et al. (2000)** revisited the decay constant and calculated a λT of 5.463 +/- 0.214 E-10/y. This was adopted as the current standard after a vote of geochronologists at the 2009 Earthtime IV meeting.

**Kwon et al. (2002)** used statistical methods to jointly estimate a decay constant of 5.4755 +/- 0.0170 E-10/y and a Fish Canyon Tuff sanidine as 28.269 +/- 0.0661 Ma (compared to the current FCT standard of 28.201 Ma; Kuiper et al., 2008).

**Kuiper et al. (2008)** used the decay constant of Min et al. (2000) when recalibrating the FCT to the current standard of 28.201 Ma (noted by Renne et al., 2010). This pairing of the FCT and λT values is the current standard used in (for example) GTS 2012.

**Renne et al. (2010)** determined the 40K decay constant as λβ = 4.9737 +/- 0.0093 E-10/y and λε = 0.5755 +/- 0.0016 E-10/y, giving a λT of 5.5492 E-10/y. This was jointly determined along with a new FCT age of 28.305 +/- 0.036 Ma.

**Renne et al. (2011)** responded to a comment on Renne et al. (2010) by Schwarz et al. (2011) by revising λβ to 4.9548 +/- 0.0134 E-10y, and λε to 0.5757 +/- 0.0016 E-10/y, giving a λT of 5.5305 E-10/y. This alters the new FCT age to 28.294 +/- 0.036 Ma.

----

## Acknowledgements:

Special thanks to my supervisor John Horner, David Eberth, Liz Freedman Fowler, Jack Wilson, Paul Renne, and Robert Sullivan. Thanks to David Bowen, Dennis Braman, Ray Butler, Peter Dodson, Federico Fanti, Jim Fassett, Joe Hartman, Rebecca Hunt-Foster, Neil Landman, Spencer Lucas, Jay Nair, Jason Noble, Ray Rogers, Julia Sankey, John Scannella, Courtney Sprain, David Varricchio, Anton Wroblewski, and everyone at the library project for discussion and sending me many essential papers. Version 0.0.01 of this chart was improved by comments from David Evans, Jim Kirkland, Andrew McDonald, and reviews from Spencer Lucas, Robert Sullivan, and two anonymous referees. Thanks to various people at SVP2006 for their helpful comments and suggestions.

# ALASKA

### PRINCE CREEK Fm

**Prince Creek Fm, AK**

The Prince Creek Fm is exposed over ~72km along the Colville River in Alaska (Mull et al., 2003; Fiorillo et al., 2010). It comprises nonmarine sandstones interbedded with carbonaceous mudstone, coal, and bentonite (Mull et al., 2003), and was divided into a lower Tuluvak, and upper Kogosukruk tongues. However, in the revised stratigraphy of Mull et al. (2003), the Tuluvak was raised to formational status, leaving the Prince Creek Fm as comprising only what was previously considered as the Kogosukruk Tongue.

The Prince Creek Fm is underlain by and intertongues with the marine Schrader Bluff Fm, and is overlain by the Sagavanirktok Fm (Mull et al., 2003).

Total thickness of the Prince Creek Fm is unknown due to lack of exposure of a complete section, but a section of ~550 m (1800 ft) is recorded by Mull et al. (2003), such that the full thickness will be greater than this.

**Age**

Radiometric dates and palynological analysis indicate a Campanian through Paleocene age for the Prince Creek Fm.

The erosive base of the Prince Creek Fm is considered as occurring within the Middle Campanian (Decker, 2007; Flores et al., 2007). Greater precision is not yet available, although the underlying unit (Schrader Bluff Fm) is marine so it might be possible to constrain this further if stratigraphically informative marine fossils are recovered immediately beneath the Prince Creek Fm. Palynostratigraphy indicates a Santonian to Early Campanian age for the of the underlying Schrader Bluff Fm (Frederiksen et al. 2002).

The contact with the overlying Sagavanirktok Fm occurs after the K-Pg boundary, at ~60 Ma (Mull et al., 2003).

A series of K-Ar and Ar / Ar radiometric dates were retrieved from rhyolitic tephras spread over ~100m thickness of section, interspersed with dinosaur bonebeds (Conrad et al., 1992). Ar / Ar dates were between 71.1 and 64.1 Ma (Conrad et al., 1992), recalibrated here as 72.0 to 64.9 Ma (see individual entry). It should be noted that many of these samples are believed to have suffered from argon loss, and have relatively high error. Previous accounts (based on these unrecalibrated dates) have suggested "a best age estimate of 69.1 +/- 0.3 Ma" (Fiorillo et al. 2010, p. 458); when readjusted for new standards this becomes 70.0 +/- 0.3 Ma. An Ar / Ar reanalysis of one of the lower tephras was performed by Obradovich (it is not specified which specific horizon this was), cited as a pers. comm. in 1993 by Clemens (1994); the reanalysis yielded a date of 72.9 Ma, recalibrated here to 73.4 Ma.

Frederiksen (1991) sampled the Ocean Point area of the Colville River for palynomorphs (the same area which yields dinosaur remains). He concluded that the recovered palynomorphs were from within the "middle" Maastrichtian *Wodehouseia spinata* Assemblage Zone.

----

### 70 ± 0.3

**Conrad et al. (1992); Fiorillo et al., (2010); recalibration, Fowler (this article)**

~69.1 +/- 0.3 Ma (Ar / Ar, glass, average of multiple samples from Conrad et al. 1992; Fiorillo et al., 2010)

**~70.0 +/- 0.3 Ma** (Ar / Ar, glass, average of multiple samples; recalibration, this article; see below)

A series of K-Ar and Ar / Ar radiometric dates were retrieved from rhyolitic tephras spread over ~100m thickness of section (Conrad et al., 1992); Ar / Ar dates were between 71.1 and 64.1 Ma, recalibrated here as 72.0 to 64.9 Ma. It should be noted that many of these samples are believed to have suffered from argon loss, and have relatively high error.

Previous accounts (based on unrecalibrated dates) have suggested "a best age estimate of 69.1 +/- 0.3 Ma" (Fiorillo et al., 2010, p. 458); when readjusted to the Kuiper et al. (2008) standard, this becomes 70.0 +/- 0.3 Ma.

Note that one of the lower tephras was reanalysed by Orbadovich (1993 pers. comm. to Gangloff et al., 2005; see individual entry, below).

**Standard**

Conrad et al., (1992) use the unusual standard of SB-3 at 162.9 Ma. Few intercalibration analyses include SB-3, but through combination of intercalibrations can be shown to be equivalent to FCT at 27.84 Ma (Cebula et al., 1986; Renne et al., 1998; Jourdan et al., 2006; Schwarz and Trieloff, 2007). Decay constant (λT) follows Steiger & Jaeger (1977), at 5.543 +/- 0.010 E-10/y.

**Recalibration (Fowler, this article)**

I have chosen not to list the individual radiometric dates from individual samples (these can be found in the accompanying excel recalibration sheet). Instead I have simply posted a recalibration of the average age as given by Fiorillo et al., (2010).

Legacy date; FCT at 27.84 Ma (see above); legacy λT at 5.543 +/- 0.010 E-10/y (Steiger & Jaeger, 1977).

~69.1 +/- 0.3 Ma (Ar / Ar, glass, average of multiple samples from Conrad et al. 1992; Fiorillo et al., 2010)

1st recalibration; FCT at 28.201 +/- 0.023 Ma (1σ; Kuiper et al., 2008); λT at 5.463 E-10/y +/- 1.07 E-11/y; 1σ (Min et al., 2000)

~70.0 +/- 0.3 Ma (Ar / Ar, glass, average of multiple samples; recalibration, this article)

2nd recalibration (for reference); FCT at 28.294 +/- 0.294 Ma (1σ), and λT at 5.531 E-10/y +/- 1.35 E-12/y (1σ; both Renne et al., 2011).

~70.2 +/- 0.3 Ma (Ar / Ar, glass, average of multiple samples; recalibration, this article)

----

### 73.4

**Clemens (1994); recalibration, Fowler (this article)**

72.9 Ma (Ar / Ar, sanidine, Obradovich, pers. comm. 1993 in Clemens, 1994)

**73.4** Ma (Ar / Ar, sanidine; recalibration, this article; see below)

A series of K-Ar and Ar / Ar radiometric dates were retrieved from rhyolitic tephras spread over ~100m thickness of section (Conrad et al., 1992); Ar / Ar dates were between 71.1 and 64.1 Ma, recalibrated here (above) as 72.0 to 64.9 Ma. It should be noted that many of these samples are believed to have suffered from argon loss, and have relatively high error. Previous accounts (based on unrecalibrated dates) have suggested "a best age estimate of 69.1 +/- 0.3 Ma" (Fiorillo et al., 2010, p. 458); when readjusted to the Kuiper et al. (2008) standard, this becomes 70.0 +/- 0.3 Ma (see individual entry above).

Gangloff et al. (2005) state that an Ar / Ar reanalysis of some samples (it is not specified which specific horizon this was) was performed by Obradovich, citing a pers. comm. in 1993, however, they give no precise date and merely state (p. 998) that "[t]he best results of a reanalysis using 40Ar/39Ar single sanidine crystals (Obradovich, personal commun., 1993) would place the lowermost bone bed between 71 and 72 My". More information is offered by Gangloff & Fiorillo (2010), who state that Obradovich determined an age of 72.9 Ma for one of the lower tuffs sampled by Conrad et al. (1992). Based on the typical standard used by Obradovich at this time (an FCT of 28.03; atypical for the time), this can be recalibrated to give an age of 73.4 Ma.

**Standard**

The standard used in the reanalysis of Obradovich is not explicitly known, but can be inferred based on other analyses performed by Obradovich during this time. Hicks et al. (1995; of which Obradovich is a couthor) used the Taylor Creek Rhyolite (Dalrymple & Duffield, 1988) normalized against a 520.4 Ma age for the MMhb-1 (Samson & Alexander, 1987). A precise age for the TCR is not given by Hicks et al. (1995), however, in other analyses with Hicks and Obradovich as authors (e.g. Hicks et al., 2002; see Taylor Creek Rhyolite Note) the TCR is 28.32 when calibrated against an MMhb-1 of 520.4, so I will assume that this is the value used here.

Decay constant (λT) should be 5.543 +/- 0.010 E-10/y (Steiger and Jaeger, 1977), confirmed by reference to Hicks et al. (2002).

**Recalibration (Fowler, this article)**

A legacy FCT value of 28.03 was used, as this was given by Hicks et al. (2002) as equivalent of the TCR at 28.32 (see note on TCR standard, and above note on standards). The unusual standard is due to the particular methods of Obradovich, who ran the analysis.

Legacy date; FCT at 28.03 (see above); legacy λT at 5.543 +/- 0.010 E-10/y (Steiger and Jaeger, 1977).

72.9 Ma (Ar / Ar, sanidine, Obradovich, pers. comm. 1993 in Clemens, 1994)

1st recalibration; FCT at 28.201 +/- 0.023 Ma (1σ; Kuiper et al., 2008); λT at 5.463 E-10/y +/- 1.07 E-11/y; 1σ (Min et al., 2000)

73.4 Ma (Ar / Ar, sanidine; recalibration, this article)

2nd recalibration (for reference); FCT at 28.294 +/- 0.294 Ma (1σ), and λT at 5.531 E-10/y +/- 1.35 E-12/y (1σ; both Renne et al., 2011).

73.6 Ma (Ar / Ar, sanidine; recalibration, this article)

----

# ALBERTA

## NW Plains (columns O-R)

### WAPITI Fm

**Wapiti Fm, Alberta**

The Wapiti Fm comprises nonmarine interbedded fluvial sandstones, siltstones, and mudstones, with occasional coals and lacustrine units (Fanti & Catuneanu, 2009).

The most recent revision of Wapiti Fm stratigraphy divides it into 5 numbered units (Fanti & Catuneanu, 2009).

**Correlation**

The Wapiti Fm is the more landward equivalent of the Oldman, Dinosaur Park, Bearpaw, and Horseshoe Canyon Fms. Sequence stratigraphic analysis (Fanti & Catuneanu, 2010) has correlated surfaces from the Wapiti Fm to the more basinward Belly River and Horseshoe Canyon Fms. The specific details of these surfaces are given in the notes for individual units.

----

### Unit 5

**Unit 5**

Unit 5 has a gradational contact with Unit 4 and is characterized in its lower part by channel and floodplain deposits in roughly equal proportions, and an upper part comprising thick coal units, referred to as the Cutbank Coal Zone (Fanti & Catuneanu, 2009).

**Age**

The Red Willow Coal Zone at the top of Unit 4 is considered to be the lateral equivalent of the Drumheller Marine Tongue transgressive event (Fanti & Catuneanu, 2009). This had previously been considered to be equivalent to the Campanian-Maastrichtian boundary (e.g. Fanti & Catuneanu, 2009), however, alteration to both the definition of the Campanian-Maastrichtian boundary (Ogg & Hinnov, 2012) and recalibration of a radiometric date from the Drumheller Marine Tongue (see individual entry) suggest that this horizon is lower Maastrichtian instead.

The Cutbank Coal Zone occurs at the top of Unit 5; palynological analysis supports Unit 5 being correlated with the Carbon & Thompson Coal Zones at the top of the Horseshoe Canyon Fm (Fanti & Catuneanu, 2010). The contact with the overlying Entrance Mbr of the Scollard Fm is abrupt (Fanti & Catuneanu, 2010), with a hiatus in deposition likely, although it is not known how long this hiatus may be.

----

### Unit 4

**Unit 4**

Unit 4 comprises up to ~350 m of terrestrial channel sediments and extensive overbank facies, deposited during high-accommodation conditions, and roughly correlating with the lower part of the Horseshoe Canyon Fm of southern & central Alberta (Fanti & Catuneanu, 2009). Unit 4 is capped by the Red Willow Coal Zone that is age-equivalent to the Drumheller Marine Tongue (Fanti & Catuneanu, 2009).

**Age**

An Ar / Ar ash date of 73.73 Ma (recalibrated; see individual entry) has been recovered from near the base of Unit 4 (Fanti & Catuneanu, 2009). Age of the basal contact is further constrained by chronostratigraphic indicators at the top of the underlying Unit 3, including a radiometric date and correlation of the Maximum Flooding Surface of the Bearpaw Transgression (Fanti & Catuneanu, 2009).

An Ar / Ar date of 71.89 Ma (see individual entry) occurs in the middle of Unit 4 (Fanti et al., 2015).

The Red Willow Coal Zone at the top of Unit 4 is considered to be the lateral equivalent of the Drumheller Marine Tongue transgressive event (Fanti & Catuneanu, 2009). This had previously been considered to be equivalent to the Campanian-Maastrichtian boundary (e.g. Fanti & Catuneanu, 2009), however, alteration to both the definition of the Campanian-Maastrichtian boundary (Ogg & Hinnov, 2012) and recalibration of a radiometric date from the Drumheller Marine Tongue (see individual entry) suggest that this horizon is lower Maastrichtian instead.

----

### 71.89 ± 0.14

**Fanti et al. (2015)**

**71.89 +/- 0.14** Ma (Ar / Ar, mineral not stated, Fanti et al., 2015)

A 25 cm thick, altered volcanic ash located approximately 180 cm below the "Wapiti River Bonebed" which occurs in Unit 4 of the Wapiti Fm (Fanti et al., 2015). This bonebed yields material from the ceratopsid dinosaur Pachyrhinosaurus lakustai.

**Standards**

Few details regarding the analysis are available. The standards used to acquire the data are not explicitly stated. Fanti et al. (2015) state that the Ar / Ar analysis was conducted at the Berkeley Geochronology Center under the direction of A.L. Deino. Since this analysis was perfromed after 2009 (it is not mentioned in Fanti & Catuneanu, 2009), then it is likely that it uses up to date standards. Berkeley Geochronology Center uses both the Kuiper et al. (2008) standard, and those of Renne et al. (2011), so I do not know which was used to acquire this date. However, in past analyses Deino has used the standard of Kuiper et al. (2008). As such, I am leaving the radiometric date unchanged.

----

### 73.73 ± 0.25

**Fanti & Catuneanu (2009); recalibration, Fowler (this article)**

73.25 +/- 0.25 Ma (Ar / Ar, unknown mineral, Fanti & Catuneanu, 2009)

**73.73 +/- 0.25** Ma (Ar / Ar, unknown mineral, recalibration, this article)

The ash is 27m above the Pipestone Creek Pachyrhinosaurus lakustai bonebed, which is very near the base of Unit 4 (Tanke, 2004).

Fanti & Catuneanu (2009, p.278) cite the source of this date as "Eberth in Currie et al., 2008" (Pachyrhinosaurus book, Indiana Univ. Press). Currie et al. (2008, p. 7) refer to this date as 73.27 +/- 0.25 Ma, which differs slightly from that given by Fanti & Catuneanu (2009).

Similarly, Tanke (2004) gives the date as 73.27 +/- 0.25 Ma, citing Eberth pers. comm.. However, Tanke (2004) states that the analysis is K-Ar, rather than Ar / Ar (as stated by Fanti & Catuneanu, 2009). It seems likely that this is in error as K-Ar analyses are much less frequently conducted during this time period (2000's), and generally have much higher error.

**Standard**

No indication is given for the standards used for this analysis. However, the FCT equivalent during the time of analysis should be 28.02 Ma (Renne et al., 1998), the next change in FCT was not until Kuiper (2008).

**Recalibration (this article)**

Legacy date; FCT at 28.02 (see above); legacy λT at 5.543 +/- 0.010 E-10/y (Steiger & Jaeger, 1977)).

73.25 +/- 0.25 Ma (Ar / Ar, unknown mineral, Fanti & Catuneanu, 2009)

1st recalibration; FCT at 28.201 +/- 0.023 Ma (1σ; Kuiper et al., 2008); λT at 5.463 E-10/y +/- 1.07 E-11/y; 1σ (Min et al., 2000)

73.73 +/- 0.25 Ma (Ar / Ar, unknown mineral, recalibration, this article)

2nd recalibration (for reference); FCT at 28.294 +/- 0.294 Ma (1σ), and λT at 5.531 E-10/y +/- 1.35 E-12/y (1σ; both Renne et al., 2011).

73.95 +/- 0.25 Ma (Ar / Ar, unknown mineral, recalibration, this article)

----

### Unit 3

**Unit 3**

Unit 3 comprises ~140 m of terrestrial mudstones, and fluvial sandstones which form an overall fining upwards succession (Fanti & Catuneanu, 2009). General dominance of coarse grained facies in the lower part of the succession suggests an overall low accommodation setting (Fanti & Catuneanu, 2009). The upper part of Unit 3 is comprised more dominantly of fine grained deposits, including thin coals and IHS, suggesting overall increase in accommodation.

**Age**

Fanti & Catuneanu (2010) tentatively correlate the amalgamated channel units at the base of Unit 3 with the Claggett cyclothem maximum regressive surface which occurs at the base of the Dinosaur Park Fm in southeastern Alberta. This is dated here as ~77 Ma, within the *Baculites scotti* zone.

A radiometric date of 73.77 Ma +/- 1.46 Ma (not recalibrated; see individual entry) is reported from the uppermost part of Unit 3 by Fanti & Catuneanu (2009). The second order Maximum Flooding Surface of the Bearpaw Shale (occurring within the B. compressus zone; 74.21-73.91 Ma; Ogg & Hinnov, 2012) lies within fine grained fluvial deposits in the upper part of Unit 3 (including coaly beds). This is consistent with the radiometric date. An Ar / Ar ash date of 73.73 Ma (recalibrated; see individual entry) has been recovered from near the base of the overlying Unit 4 (Fanti & Catuneanu, 2009).

Hence here I show Unit 3 ranging from 77.0 - 73.9 Ma. Note that this causes a slight issue with the single radiometric date, although is well within the large stated error (+/- 1.46 Ma).

----

### 73.77 ± 1.46

**Fanti & Catuneanu (2009)**

73.77 +/- 1.46 Ma (Fanti & Catuneanu, 2009)

Fanti & Catuneanu (2009) cite "Eberth in Fanti, 2007", but no explicit reference is given for the date in Fanti (2007; an extended abstract), and details of the analysis are not stated. It is not known if the analysis is Ar / Ar or K-Ar. The error is quite high (1.46 Ma) so K-Ar might be more likely.

----

### Unit 2

**Unit 2**

Unit 2 (~100m thick) records a transition from the thick tabular coals and mainly fine grained sediment of Unit 1, through to thinner discontinuous coals and more coarse grained channel sandstones (Fanti & Catuneanu, 2009).

**Age**

Unit 2 is correlated with the Oldman Fm of south eastern Alberta (Fanti & Catuneanu, 2010).

The lower contact is illustrated by Fanti & Catuneanu (2010) as coincident with the base of the Oldman Fm, but it is not made clear whether this is the Herronton Sandstone or the overlying mudstone ("Unit 1" of the Oldman Fm). As the Wapiti Fm Unit 1/2 boundary juxtaposes fine coal-bearing stat of Unit 1 with coarse alluvial sandstones at the base of Unit 2, then I am placing the boundary as equivalent to the base of the Herronton Sandstone.

Fanti & Catuneanu (2010) tentatively correlate the amalgamated channel units at the base of Unit 3 with the Claggett cyclothem maximum regressive surface which occurs at the base of the Dinosaur Park Fm in southeastern Alberta. This is dated here as ~77 Ma, within the *Baculites scotti* zone.

----

### Unit 1

**Unit 1**

Unit 1 (~120m thick) marks the transition from the underlying marine facies of the Puskwaskau Fm to fluvial facies of the Wapiti Fm (Fanti & Catuneanu, 2009). The lower boundary is defined by the first laterally persistent coal, and thick coals are present throughout unit 1 (Fanti & Catuneanu, 2010).

Age

Basal coals are correlated with the McKay Coal Zone of the Foremost Fm by Fanti & Catuneanu (2010), supported by palynological analysis (Dawson et al., 1994a,b). Other than this, there are few chronostratigraphic controls on the age of the basal contact, so here I follow Fanti & Catuneanu in drawing it as correlated with the base of the Foremost Fm.

The upper contact is illustrated by Fanti & Catuneanu (2010) as coincident with the base of the Oldman Fm, but it is not made clear whether this is the Herronton Sandstone or the overlying mudstone ("Unit 1" of the Oldman Fm). As the boundary between Unit 1 and Unit 2 of the Wapiti Fm juxtaposes fine coal-bearing strata of unit 1 with coarse alluvial sandstones at the base of Unit 2, then I am placing the boundary as equivalent to the base of the Herronton Sandstone.

----

## S. Plains

### WILLOW CREEK Fm

**Willow Creek**

The Willow Creek Fm is a more landward equivalent of the Scollard Fm (and equivalents), and comprises up to 1300m of terrestrial sandstones and mudstones, but notably lacks coal units seen in lateral equivalents (Hamblin, 2010).

The Willow Creek Fm conformably overlies the St Mary River Fm to the west, but disconformably to the east (Hamblin, 1998).

**Age**

The lower contact of the Willow Creek Fm with the underlying St Mary River Fm occurs at the Kneehills Tuff which has been dated elsewhere as 66.97 Ma (Hicks et al., 2003; recalibrated here). However, it is likely that there is a hiatus of unknown length which occurs at the base of the Willow Creek Fm (Hamblin, 2010; as similarly seen in the Scollard Fm to the East). I have therefore chosen to represent this contact by a short hiatus, although it is unknown as to its duration, and is simply shown here to be the same as the Scollard Fm.

The upper contact of the Willow Creek Fm occurs well after the K-Pg boundary and so is not depicted here.

----

### St MARY RIVER Fm

**St Mary River Fm**

Up to ~750 m thick, the St. Mary River Fm was deposited in an entirely terrestrial environmental setting, a more landward equivalent of the Horseshoe Canyon Fm (Hamblin, 1998). The lower 60m of the St Mary River Fm is considered approximately equivalent to the upper 60m of the Bearpaw Shale in the Cypress Hills area, whereas the uppermost unit is a white sandstone with mauve shale and tuffs that is a direct equivalent of the Whitemud and Battle Fms (Hamblin, 1998).

**Age**:

Lerbekmo & Lehtola (2011) place the base of the St. Mary River Fm as C32n.1r (71.939 - 71.689 Ma; Ogg, 2012).

The upper formational contact is defined by the Kneehills Tuff (66.97 Ma; recalibrated from Hicks et al., 2003; see individual entry) which separates the St. Mary River Fm from the overlying Willow Creek Fm (Dawson et al., 1994).

----

### BLOOD RESERVE Fm

**Blood Reserve Fm**

The Blood Reserve Fm is a shallow marine sandstone facies deposited during the regression of the Bearpaw Seaway. Although lithostratigraphically equivalent to the Fox Hills Fm, the Blood Reserve Fm (and equivalent Horsethief Fm in Montana) was deposited earlier, representing the initial phase of the Fox Hills regression (Gill & Cobban, 1973).

**Age**

The base of the Blood Reserve Fm is shown as occurring near the middle of C32n.3n (~71.9 Ma) by Lerbekmo & Lehtola (2011). The base of the overlying St. Mary River Fm was placed as C32n.1r by Lerbekmo & Lehtola (2011), which is ~71.5 Ma. As a regressive sandstone deposited ahead of a prograding delta, the Blood Reserve Fm is expected to be time transgressive at both its base and top, becoming younger to the east. As such a hiatus is expected between the top of the Blood Reserve Fm and the overlying St, Mary River Fm.

Ammonite biostratigraphy of the correlative Horsethief Fm in Montana suggests that the initial regression of the Bearpaw seaway began during the *B. compressus* zone, and continued through the *B. grandis* zone (74.21 - 70.44 Ma; Ogg & Hinnov, 2012).

Thus, here I have plotted the Blood Reserve Fm from C32.3n (~71.9 Ma) to C32n.1r (~71.5 Ma), although it is likely to be time transgressive at its base, and at the contact with the overlying St. Mary River Fm.

----

### PAKOWKI

**Pakowiki Fm, CAN**

The Pakowki Fm is a marine shale resting upon a ravinement surface which forms the boundary with the underlying Milk River Fm. It is overlain by sediments of the Foremost Fm, Belly River Group (not shown here specifically).

A hiatus of ~2.5 m.y. occurs between the Pakowki Fm and the underlying Milk River Fm.

**Age**

**Lower contact**

Obradovich and Cobban (1975) show the lower contact of the Pakowki within the *Baculites obtusus* zone (80.97 - 80.67 Ma; Ogg & Hinnov, 2012). Leahy and Lerbekmo show the lower contact occurring within the lower part of magnetozone C33r. This is corroborated by Payenberg et al. (2002) who recovered a U-Pb date of 80.7 +/- 0.2 Ma for a bentonite recovered from the lower part of the Pakowki Fm.

**Upper contact**

Obradovich and Cobban (1975) show the upper Pakowki ranging from the *B. mclearni* zone (80.67 - 80.21 Ma) through to the top of the *B. asperiformis* zone (80.21 - 79.64 Ma; all zone ranges Ogg & Hinnov, 2012). Although Leahy & Lerbekmo (1995) note that the ranges of *B. obtusus*, *B. mclearni*, and *B. asperifomis* overlap in the Pakowki Formation (which does not happen in US equivalent sections), with the likelihood that this is caused by the earlier than usual appearance of *B. asperiformis*.

Lerbekmo (1989; and Leahy & Lerbekmo, 1995) show the basal contact of the Foremost Fm with the underlying Pakowki Fm occurring in the uppermost C33r, with C33n occurring <10m above. This is problematic as the radiometric date recovered ~30 m above the base of the Foremost is 80.17 +/- 0.15 Ma (Eberth, 2005; recalibrated; see individual entry); this should be within C33n according to Lerbekmo (1989 and Leahy & Lerbekmo, 1995), but the base of C33n is defined as 79.900 by Ogg (2012), i.e. above the radiometric date. Lerbekmo (1989) shows that the first sample analysed from the base of the Foremost Fm is of normal polarity, and assigned to C33n, hence the illustration of the C33r-33n boundary in the basal Foremost Fm (Leahy & Lerbekmo, 1995) is an artifact of the convention of drawing the boundary between two chrons halfway between the adjacent samples.

The radiometric date may also cause issue with proposed ammonite biostratigraphy. Eberth (2005) suggests that the base of the Foremost Fm is correlative with the Baculites asperiformis zone (although no reference is given for an ammonite occurrence in Alberta that corroborates this, this relationship is shown in Leahy & Lerbekmo, 1995). Since Ogg & Hinnov (2012) define the base of the *B. asperiformis* zone as 80.21 Ma, and that the radiometric date from ~30 m above the base of the Foremost is 80.17 Ma (Eberth, 2005; see above), then this suggests that the lowermost 30 m of the Foremost Fm were deposited in a mere 40,000 years. This discrepancy may be explained if (as suspected; Leahy & Lerbekmo, 1995) *B. asperiformis* occurs slightly earlier in Canadian sections than is typical.

The Pakowki Fm is therefore shown here to extend only up to the upper boundary of the C33r magnetozone. However, this is almost certainly incorrect, but will not be resolved until solutions are found to the issues with the radiometric date from the base of the Foremost Fm.

----

### Lower

**Lower Pakowki Fm:**

Leahy & Lerbekmo (1995) state that the Lower Pakowki occurs in *Baculites obtusus* ammonite zone (80.97-80.67 Ma; Ogg & Hinnov, 2012).

----

### 80.7 ± 0.2

**Payenberg et al. (2002)**

Ardmore bentonite (Payenberg et al., 2002)

"10-34-17-3w4" core at 328.8m depth (Payenberg et al., 2002)

80.7 +/- 0.2 Ma (1σ); sample no. 1034-2 (U / Pb, zircon, 2 crystals; Payenberg et al., 2002)

Payenberg et al. (2002) record this U / Pb date from one of nine bentonites present in the lower part of the Pakowki Fm (a more precise stratigraphic position is not given explicitly). These bentonites are noted in the text as being referred to as the Ardmore bentonites.

**Other ages for the Ardmore bentonite**

The Ardmore bentonite is also dated by other workers who give slightly different ages, all based on Ar / Ar dates. Obradovich (1993; Claggett Shale, WY) reports an age of 80.54 +/- 0.55 Ma (81.041 +/- 0.55 Ma, recalibrated), Hicks et al. (1999; Pierre Shale, WY) gives 80.04 +/- 0.4 Ma (80.54 +/- 0.4 Ma; recalibrated), and Hicks et al. (1995, Claggett Shale, Elk Basin, WY) give 80.71 +/- 0.55 Ma (81.21 +/- 0.17 Ma; recalibrated; all recalibrations by Fowler, this article, using Kuiper et al., 2008, standards; see recalibration sheet).

----

MILK RIV.

**Milk River Fm**

The Milk River Fm is a progradational clastic wedge subdivided into three members (from lower to upper): the Telegraph Creek (regressive marine sandstone), Virgelle (shallow marine, estuarine & terrestrial channelling), and Deadhorse Coulee (terrestrial; Braman, 2001).

Larson (2008) notes that the Deadhorse Coulee Mbr is the only source of vertebrate fossils in the Milk River Fm, which is of importance as it means that the range of the Aqulian NALMA cannot technically be extended down to the base of the Milk River Fm.

**Upper contact (Deadhorse Coulee Mbr)**

The upper contact of the Deadhorse Coulee Mbr (and therefore the Milk River Fm) occurs in the lowermost part of C33n (Leahy & Lerbekmo, 1995), at the base of the *Scaphites leei III* ammonite zone (Payenberg et al., 2002).

The upper contact of the Deadhorse Coulee Mbr is marked by an extensive black-chert-pebble horizon, interpreted to represent a ravinement surface separating the regressive Milk River / Eagle Fms from the overlying transgressive Pakowki / Clagget Fms (Payenberg et al., 2002). The depositional hiatus represented by this surface is illustrated by Payenberg et al. (2002) as extending from the *Scaphites leei III* zone through to the *B. obtusus* zone present at the base of the Pakowki Fm.

The lower contact of the Deadhorse Coulee Mbr occurs within the uppermost part of C34n (Leahy & Lerbekmo, 1995).

**Lower contact (Telegraph Creek Mbr)**

The lower contact of the Telegraph Creek Mbr is defined by correlation with the Telegraph Creek Formation of Montana, which has yielded specimens of *Desmoscaphites bassleri*; as such I have plotted the base of the Milk River Fm as occurring at the base of the *D. bassleri* zone.

**Note**:

Jinnah et al. (2009, p.297) suggest that radiometric dates are published from the Milk River: "radiometric dating of the Milk River Formation place it firmly within the Santonian and earliest Campanian (84.5–83.5 Ma; Leahy and Lerbekmo, 1995; Payenberg et al., 2002)". However, the dates to which Jinnah et al. refer were extracted from equivalent units in Montana, or the overlying Pakowki Fm, no actual dates have been recovered from the Milk River Fm itself.

----

## South Central

### SCOLLARD Fm

**Scollard Fm**

The Scollard Fm is a Canadian equivalent of the Hell Creek and Lance Fms which occur in the Northern US (although the Scollard is more time-inclusive). The Scollard Fm bears comparable lithologies, comprising channel sandstones, overbank fines, and coals. The Scollard Fm is subdivided into Lower and Upper members, with the division occurring at the K-Pg boundary, above which coaly units are prevalent and well developed (similarly seen in the US where typically the coaly units are considered a separate formation).

**Age**

An Ar / Ar date of 66.97 Ma (recalibrated; see individual entry) is recorded from the underlying Battle Fm, thereby constraining the maximum possible age of the basal part of the Scollard Fm.

The Scollard Fm basal contact with the underlying Battle Fm is disconformable, with the duration of the depositional hiatus unknown (Russell, 1983; Dawson et al, 1994b). As with the Hell Creek Fm of Montana, the basalmost beds of the Scollard belong to C30n (Lerbekmo & Coulter, 1985; Lerbekmo et al., 1995), and most workers seem to place the base of the Scollard approximately halfway through the C30n magnetozone (Eberth pers. comm. 2006; Lerbekmo & Braman, 2002).

The base of the Scollard Fm is known to be older than the base of the Frenchman Fm. The Frenchman Fm (which occurs further east in Saskatchewan) has an erosive contact with the underlying Battle Fm (Dawson et al., 1994b), and through magnetostratigraphic analysis has been shown to be younger (C29r) at its measurable point than the Scollard Fm (C30n; Lerbekmo, 1999; Lerbekmo & Braman, 2002).

The upper contact of the Scollard Fm occurs above the K-Pg boundary, and so is not considered here.

Lerbekmo (1999) shows a small normal subchron occurring at the K-T boundary, Wood Mountain core, Saskatchewan, CAN, at the top of the Frenchman Fm. A similarly placed normal subchron was detected 15-20m below the K-T boundary in the Scollard Fm, Red Deer River, Alberta (Lerbekmo & Coulter, 1985). Whether this is merely inconsistent placement, two separate normal polarity horizons, or demonstrates that a small hiatus exists at the top of the Frenchman Fm is not clear. On this chart, a single normal horizon is shown at the K-T boundary since this is the position illustrated in the most recent paper (Lerbekmo, 2008).

The Nevis & Arbour coals (both Upper Scollard) both have Ar / Ar dates (see Braman & Sweet, 2012), however, only the recalibrated Nevis coal date is shown here as the Arbour coal is too young to fit within the boundaries of the chart. Lerbekmo & Braman (2002) show the Nevis Coal (their coal 13) occurring in the uppermost 29r, whereas the Arbour Coal (their coal 14) occurs in the middle of 29n.

**Accommodation variation vs Hell Creek - Fort Union Fm**

The radiometric date of 65.32 Ma (recalibrated) for the Nevis coal ~36 cm above the K-Pg boundary suggests a relatively low rate of accommodation space creation / net sedimentation immediately after the K-Pg boundary. This contrasts with high rates of sediment accumulation in the earliest Paleogene suggested by radiometric dating conducted in the Hell Creek - Fort Union Fms of Montana (Renne et al., 2013; Sprain et al., 2014).

----

### 65.32

**Eberth & Deino (2005); recalibration, Fowler (this article)**

Bentonite 36 cm above the K-Pg boundary (at the top of the Nevis coal, no. 13; Eberth & Braman, 2012; Braman & Sweet, 2012)

64.90 Ma (error not known; Eberth & Deino, 2005)

**65.32** Ma (recalibration; Fowler, this article, see below)

**Standard**

Not currently known (I do not have the initial reference), but can be calculated by recalibration performed on another date given by Eberth & Deino (2005). The age of an ash 8 m above the base of the Bearpaw Shale was given as 74.98 Ma by Eberth & Braman (2012), with a recalibrated date of 75.5 Ma given by Eberth (Tyrrell talk in 2011).When recalibrated to the Kuiper et al. (2008) standard and Min et al. (2000) decay constant, and assuming an original FCT standard of 28.02 and Min et al. (2000) decay constant, this yields a date of 75.458 Ma, i.e. the same as the date given in the Eberth 2011 lecture. It is likely that the analyses in Eberth & Deino (2005) were conducted using the same standards, which is what I have used for the recalibration, below).

**Recalibration**

Legacy dates; FCT at 28.02 *Renne et al., 1998); legacy λT at 5.463 E-10/y +/- 1.07 E-11/y (1σ; Min et al., 2000).

64.90 Ma (Eberth & Deino, 2005)

1st recalibration; FCT at 28.201 +/- 0.023 Ma (1σ; Kuiper et al., 2008), and λT at 5.463 E-10/y +/- 1.07 E-11/y (1σ; Min et al., 2000)

65.32 Ma (Fowler, this article)

2nd recalibration (for reference); FCT at 28.294 +/- 0.294 Ma (1σ), and λT at 5.531 E-10/y +/- 1.35 E-12/y (1σ; both Renne et al., 2011).

65.52 Ma (Fowler, this article)

----

### BATTLE Fm

**Battle Fm**

The Battle Fm comprises typically 9m (but up to 14 m) of mauve grey nonmarine mudstones (Braman et al., 1999). This includes the Kneehills Tuff (0.3m thick, 3.6m from top of Battle Fm, Russell, 1983).

**Age**

The Kneehills Tuff occurs within C30N (Lerbekmo & Braman, 2002) and has been Ar / Ar dated at 66.97 +/- 0.10 Ma (recalibrated here; Hicks et al., 2003).

A disconformity between the the Battle Fm and underlying Whitemud is noted by Lerbekmo (1999) based on palynostratigraphy in Saskatchewan, although both units occur within C30n. Similarly, the upper contact with the Scollard Fm is also disconformable, with the duration of the depositional hiatus unknown (Russell, 1983; Dawson et al., 1994).

----

### 66.97 ± 0.10

**Hicks et al. (2003)**

Kneehills Tuff

~1.8m above Kneehills Tuff, Drumheller, Alberta (Obradovich, 1993; Hicks et al., 2003) = 4.5m above base of Battle Fm

66.8 +/- 1.1 Ma (95% confidence interval for the error of the mean, ~2σ; sample AK-476; Obradovich, 1993; no further anlaysis details given).

66.56 +/- 0.10 Ma (1σ); (Ar / Ar, sanidine, 8 samples: 1 x 6 crystals; 3 x 7 crystals; 4 x 8 crystals; sample AK-19; Hicks et al., 2003)

66.97 +/- 0.10 Ma (1σ); (recalibration of Hicks et al., 2003; this article; see below)

.

**Standard**

Hicks et al. (2003) state that the methods used are presented in detail in Obradovich (2002), who states that the monitor mineral used was a sanidine from the Taylor Creek Rhyolite (TCR) standard, assigned an age of 28.32 Ma (relative to MMhb-1 of 520.4). The decay constant used is not stated, but is likely to be λT = 5.543 +/- 0.010 E-10/y (Steiger & Jaeger, 1977).

Obradovich (1993) similarly used TCR relative to an MMhb-1 of 520.4 Ma, and the decay constant is again assumed to be λT = 5.543 +/- 0.010 E-10/y (Steiger & Jaeger, 1977).

**Recalibration**

A legacy FCT value of 28.03 was used, as this was given by Hicks et al. (2002) as equivalent of the TCR at 28.32. The unusual standard is due to the particular methods of Obradovich, who ran the analysis. Legacy decay constant was assumed to have been λT = 5.543 +/- 0.010 E-10/y (Steiger & Jaeger, 1977). For recalibration, it is assumed that dates of Obradovich (1993) were calculated using the same TCR date and λT.

For a discussion of the issues surrounding recalibration and comparison of the TCR and FCT standards in Obradovich analyses from the 1990's through to ~2002, see the Ar-Ar notes elsewhere on this chart.

Legacy dates; legacy FCT at 28.03; legacy λT at 5.543 +/- 0.010 E-10/y.

66.56 +/- 0.1 Ma (1σ); (Ar / Ar, sanidine, 8 samples: 1 x 6 crystals; 3 x 7 crystals; 4 x 8 crystals; AK-19; Hicks et al., 2003)

66.8 +/- 1.1 Ma (95% confidence interval for the error of the mean, ~2σ; sample AK-476; Obradovich, 1993; no further analysis details given).

1st recalibration; FCT at 28.201 +/- 0.023 Ma (1σ; Kuiper et al., 2008), and λT at 5.463 E-10/y +/- 1.07 E-11/y (1σ; Min et al., 2000)

66.97 +/- 0.10 Ma (1σ); (recalibration of Hicks et al., 2002; Fowler, this article)

67.21 +/- 1.1 Ma (95% confidence interval for the error of the mean, ~2σ); (recalibration of Obradovich, 1993; Fowler, this article)

2nd recalibration (for reference); FCT at 28.294 +/- 0.294 Ma (1σ), and λT at 5.531 E-10/y +/- 1.35 E-12/y (1σ; both Renne et al., 2011).

67.18 +/- 0.10 Ma (1σ); (recalibration of Hicks et al., 2002; Fowler, this article)

67.21 +/- 1.1 Ma (95% confidence interval for the error of the mean, ~2σ); (recalibration of Obradovich, 1993; Fowler, this article)

**Notes**

In GTS 2012, Schmitz (2012b; p.1048) gives a date of 67.29 +/- 1.11 ArAr (2σ; same +/- 1.11 with and without λ error) and cites Obradovich (1993). As shown above, the Obradovich date is 66.8 Ma, and when recalibrated to the Kuiper et al. (2008) standard it is 67.21 Ma. The difference between the recalibrated date calculated here, and that of Schmitz (2012b) is that Schmitz recalculated using a legacy FCT at 28.00, whereas I used 28.03 (see above).

----

### HORSESHOE CANYON Fm

**Horseshoe Canyon Fm (Eberth & Braman, 2012)**

The paralic to fully nonmarine Horseshoe Canyon Formation was deposited during both regression and transgression of the Bearpaw Seaway, and comprises up to ~250 m of channel sandstones, mudstones, and many coal units (Eberth & Braman, 2012).

It is important to Late Cretaceous chronostratigraphy as it is the primary unit from which the high-resolution magnetostratigraphy of C32r to C30n is constructed (Lerbekmo & Braman, 2002; 2005; Lerbekmo, 2009). Although it contains few radiometrically dated horizons, a combination of magnetostratigraphy and ammonite biostratigraphy can be used to position the various members.

However, the reader should note that there are conflicts between ammonite biostratigraphy, magnetostratigraphy, and radiometric dates in the lower part of the formation (see individual entries and magnetostratigraphy section).

The Horseshoe Canyon Fm was left undivided by Gibson (1977), however, later workers have subdivided it into various tongues (Hamblin, 2004), Units (Eberth, 2010), and finally formal Members (Eberth & Braman, 2012).

----

### Whitemud

**Whitemud Mbr (Eberth & Braman, 2012)**

The Whitemud comprises ~6-7 m of sandstones, siltstones, and mudstones which often weather a striking white color (Russell, 1983). The depositional environment of the stratigraphic equivalent Colgate Sandstone in Montana has most recently been suggested to represent mostly terrestrial depositional environment, with some estuarine influence (Flight, 2004; Behringer, 2008; Lerbekmo, 2009).

In Alberta, the Whitemud is considered as a member of the Horseshoe Canyon Fm (Eberth & Braman, 2012), whereas in western Saskatchewan it is ranked as a formation in its own right.

**Upper Contact (with Battle Formation)**

Marked by an unconformity, over which lie bentonitic shales of the Battle Fm (Eberth & Braman, 2012).

**Lower Contact (with Carbon Mbr, Horseshoe Canyon Fm)**

Marked by the first occurrence of white or light-colored, fine grained sandstone or siltstone (Eberth & Braman, 2012).

**Age**

Eberth & Braman (2012) state that the Whitemud Mbr is placed within the lower part of C30n (Lerbekmo & Braman, 2002; 2005), with an estimated age ~67.5 - 67 Ma (Ogg & Smith, 2004), and equivalent to the base of the Wodehouseia spinata palynozone (Nambudiri & Binda, 1991). Under the revised magnetozone definitions of Ogg (2012), this would become ~68.196 - 67.696 Ma, as shown here.

Lerbekmo (2009; Fig. 2) shows the base of the Whitemud in the very uppermost part of C30r for the Cypress Hills composite section, southeastern-most Alberta. However, in his text (and in prior publications Lerbekmo & Braman, 2002; 2005) Lerbekmo clearly states that the C30r-C30n occurs at the base of the Whitemud. This apparent conflict occurs because of the convention in drawing a magnetostratigraphic boundary halfway between the two measured points which give opposite polarities.

----

### Carbon Mbr

**Carbon Mbr (Eberth & Braman, 2012)**

Contains coal zones 11 ("Carbon": c.z.) and 12 ("Thompson" c.z.). Very variable in thickness (24 - 39 m).

**Upper Contact** (with Whitemud)

Eberth & Braman (2012) define the upper contact at the base of the first "distinctively white, clay-rich sandstone" of the Whitemud Mbr, noting that this is not clear in geophysical logs.

**Lower contact** (with Tolman Mbr)

Eberth & Braman (2012) note that the lower contact is very difficult to pick out, and is sometimes marked by a transitional zone. However, they provide the following definition: marked by the first evidence of either 1. abundant organic rich shales and coals or 2. multimeter-thick channel sandstones (Eberth & Braman, 2012).

**Age**

Lerbekmo & Braman (2002) show coals 11 and 12 as bounding chron C30r (68.369-68.196 Ma; Ogg, 2012), as shown here.

Eberth & Braman (2012) suggest that the Carbon Mbr ranges from the upper one-half of 31n to the lowermost 30n magnetozones, corresponding to an age of ~68.4-67.5 Ma; with the revised magnetostratigraphic zones of Ogg (2012) this age range changes to ~68.6-68 Ma. However, Lerbekmo (2009) makes clear that he considers the Whitemud - Carbon Mbr contact to occur at the C30r - C30n boundary, which would exclude the Carbon Mbr from C30n (as illustrated here).

Eberth & Braman (2012) also state that the base of the Carbon Member coincides with the onset of the Mancicorpus gibbus Palynozone, however the position of the base of the M. gibbus zone is variable between authors. Lerbekmo & Braman (2002) show the base of the M. gibbus zone occurring at the base of C31n in the Red Deer Valley section, but within C31r in the Cypress Hills section (the analysis of Lerbekmo & Braman 2005 only extends up to C31r, contra Eberth & Braman, 2012). Koppelhaus & Braman (2010) show the base of the *M. gibbus* zone at the uppermost extent of C31n.

In consideration of the above issues, here I tentatively plot the base of the Carbon Mbr as the uppermost part of C31n, but note that this is not particularly well-resolved.

----

### Tolman Mbr

**Tolman Mbr (Eberth & Braman, 2012)**

**Upper Contact** (with Carbon Mbr)

Eberth & Braman (2012) note that the upper contact is very difficult to pick out, and is sometimes marked by a transitional zone. However, they provide the following definition: marked by the first evidence of either 1. abundant organic rich shales and coals or 2. multimeter-thick channel sandstones.

**Lower contact** (with Morrin Mbr)

Placed at the inferred Maximum Flooding Surface of the Drumheller Marine Tongue, just above coal 10, typically represented by a multi-metre thick series of bentonite rich mudstones (Eberth & Braman, 2012).

**Age**

The lower contact is coincident with a radiometrically dated bentonite from just above coal 10, dated at 70.84 Ma (recalibrated, this article; see ash date entry; original date 70.4 Ma; Eberth & Deino, 2005; Eberth & Braman, 2012).

The age of the upper contact is more difficult to place. See comment on the Carbon Mbr for discussion.

----

### DMT

**Drumheller Marine Tongue (DMT)**

The maximum flooding surface of the DMT is equivalent to the bentonite-rich mudstones immediately above coal 10 (marking the boundary of the Morrin and Tolman Members), within which is a radiometrically dated bentonite dated at 70.84 Ma (recalibrated, this article; see ash date entry; original date 70.4 Ma; Eberth & Deino, 2005; Eberth & Braman, 2012).

Eberth & Braman (2012), and Lerbekmo & Braman (2002) place the DMT at the Maastrichtian-Campanian boundary, 70.6 Ma and coincident with the onset of the C31r magnetozone. However, redefinition of the Campanian-Maastrichtian boundary by Ogg & Hinnov (2012) results in the DMT being moved into the Lower Maastrichtian (shown here), although the new definitions of the C31r magnetozone (Ogg, 2012) does not cause conflict with the radiometric date (above).

----

### 70.9

**Eberth & Deino (2005); recalibration, Fowler (this article)**

Bentonite ~1-2 m above coal 10, at the top of the Morrin Mbr (Eberth & Braman, 2012)

70.44 Ma (error not known; Eberth & Deino, 2005)

70.89 Ma (recalibration; Fowler, this article, see below)

**Standard**

Not currently known (I do not have the initial reference), but can be calculated by recalibration performed on another date given by Eberth & Deino (2005). The age of an ash 8 m above the base of the Bearpaw Shale was given as 74.98 Ma by Eberth & Braman (2012), with a recalibrated date of 75.5 Ma given by Eberth (Tyrrell talk in 2011).When recalibrated to the Kuiper et al. (2008) standard and Min et al., (2000) decay constant, and assuming an original FCT standard of 28.02 and Min et al. (2000) decay constant, this yields a date of 75.458 Ma, i.e. the same as the date given in the Eberth 2011 lecture. It is likely that the analyses in Eberth & Deino (2005) were conducted using the same standards, which is what I have used for the recalibration, below).

**Recalibration**

Legacy dates; FCT at 28.02 (Renne et al., 1998); legacy λT at 5.463 E-10/y +/- 1.07 E-11/y (1σ; Min et al., 2000).

70.44 Ma (Eberth & Deino, 2005)

1st recalibration; FCT at 28.201 +/- 0.023 Ma (1σ; Kuiper et al., 2008), and λT at 5.463 E-10/y +/- 1.07 E-11/y (1σ; Min et al., 2000)

70.89 Ma (Fowler, this article)

2nd recalibration (for reference); FCT at 28.294 +/- 0.294 Ma (1σ), and λT at 5.531 E-10/y +/- 1.35 E-12/y (1σ; both Renne et al., 2011).

71.11 Ma (Fowler, this article)

----

### Morrin Mbr

**Morrin Mbr** (Eberth & Braman, 2012)

**Upper contact** (with Tolman Mbr)

Placed at the inferred Maximum Flooding Surface of the Drumheller Marine Tongue, just above coal 10, typically represented by a multi-metre thick series of bentonite rich mudstones (Eberth & Braman, 2012).

The upper contact is coincident with a radiometrically dated bentonite from just above coal 10, dated at 70.84 Ma (recalibrated, this article; see ash date entry; original date 70.4 Ma; Eberth & Deino, 2005; Eberth & Braman, 2012).

**Lower contact** (with Horsethief Mbr)

Placed at the top of the uppermost subbituminous coal in coal zone 8-9 (Eberth & Braman, 2012).

Lerbekmo & Braman (2012) show coal zone 8 occurring within C32n.3n and coal zone 9 within C32n.2r, which is further illustrated as being equivalent to the uppermost B. reesidei or lowermost B. jenseni ammonite zones.

Davies et al. (2014) report a detrital U-Pb date of 71.923 +/- 0.068 Ma for an ash-rich volcaniclastic sandstone ~30-40 cm below the Danek Edmontosaurus bonebed, itself ~4 m above #9 Big Island Coal Seam, near Edmonton, Alberta (Eberth & Bell, 2014). The #9 Big Island Coal Seam (Edmonton) is correlated through the subsurface to the #8-9 coal zone (Drumheller) located ~250 km south (Chen et al., 2005; Eberth & Bell, 2014). Hence, the detrital U-Pb date effectively occurs in the equivalent of the lowermost Morrin Mbr. This places a maximum age of 71.923 Ma on deposition of the lowermost Morrin Mbr, with a likely age somewhat younger (Davies et al., 2014), which is consistent with magnetostratigraphy (Lerbekmo & Braman, 2012). Eberth & Bell (2014) suggest an age of ~71.0 - 71.3 Ma for the bonebed overlying the detrital U-Pb date, however, this estimate is offered within the older stratigraphic framework of GTS 2004 (Gradstein et al., 2004), rather than the updated GTS 2012 (Gradstein et al., 2012), as used here. Thus this estimate should be revised to ~71.5 - 71.8 Ma, which is consistent with the U-Pb date.

----

### Horsethief Mbr

**Horsethief Mbr (Eberth & Braman, 2012)**

Previously the upper part of "Unit 1" (Eberth, 2010)

**Upper contact** (Morrin Mbr)

Placed at the top of the uppermost subbituminous coal in coal zone 8-9 (Eberth & Braman, 2012).

Lerbekmo & Braman (2012) show coal zone 8 occurring within C32n.3n and coal zone 9 within C32n.2r, which is further illustrated as being equivalent to the uppermost B. reesidei or lowermost B. jenseni ammonite zones.

Davies et al. (2014) report a detrital U-Pb date of 71.923 +/- 0.068 Ma for an ash-rich volcaniclastic sandstone ~30-40 cm below the Danek *Edmontosaurus* bonebed, itself ~4 m above #9 Big Island Coal Seam, near Edmonton, Alberta (Eberth & Bell, 2014). The #9 Big Island Coal Seam (Edmonton) is correlated through the subsurface to the #8-9 coal zone (Drumheller) located ~250 km south (Chen et al., 2005; Eberth & Bell, 2014). Hence, the detrital U-Pb date effectively occurs in the equivalent of the lowermost Morrin Mbr. This places a maximum age of 71.923 Ma on deposition of the lowermost Morrin Mbr, with a likely age somewhat younger (Davies et al., 2014), which is consistent with magnetostratigraphy (Lerbekmo & Braman, 2012). Eberth & Bell (2014) suggest an age of ~71.0 - 71.3 Ma for the bonebed overlying the detrital U-Pb date, however, this estimate is offered within the older stratigraphic framework of GTS 2004 (Gradstein et al., 2004), rather than the updated GTS 2012 (Gradstein et al., 2012), as used here. Thus this estimate should be revised to ~71.5 - 71.8 Ma, which is consistent with the U-Pb date.

**Lower contact** (Drumheller Mbr)

Base placed at either the base of the laterally extensive multi-metre thick Drumheller bentonite zone (on most well-logs), or at the top of coal zone 6-7 (Eberth & Braman, 2012)

Lerbekmo & Braman (2002) place both coal zones 6 and 7 within C32n.4n-5n (undifferentiated).

----

### Drumheller Mbr

**Drumheller Mbr (Eberth & Braman, 2012)**

**Upper contact** (Horsethief Mbr)

Contact placed at either the base of the laterally extensive multi-metre thick Drumherller bentonite zone (on most well-logs), or at the top of coal zone 6-7 (Eberth & Braman, 2012)

Lerbekmo & Braman (2002) place both coal zones 6 and 7 within C32n.4n-5n (undifferentiated).

**Lower contact**

The lower contact is variable geographically. In the east and south the Drumheller Mbr interfingers or rests sharply on the marine shales and sandstones of the Bearpaw Fm (Eberth & Braman, 2012). In the north and west the base of the Drumheller Mbr conformably rests on coaly deposits of the Strathmore Mbr, with the contact between the two equivalent to the maximum flooding surface of the lower tongue of the Bearpaw Fm (Eberth & Braman, 2012).

Age of the lower contact is variable dependent on geographic position. In outcrop around the town of Drumheller, the base of the Drumheller Mbr falls within C32r.1r, but becomes progressively older westwards such that it occurs in the upper part of C33n (Lerbekmo & Braman, 2002, 2005; Lerbekmo & Lehtola, 2011; Eberth & Braman, 2012). Here I follow the stratigraphic positioning of Eberth & Braman (2012), including with regards to the position of the Strathmore Mbr (see individual entry).

----

### 73.5 +/- 0.4

**Lerbekmo, 2002**

Dorothy bentonite, Bearpaw Formation

~60 m below base of the Horseshoe Canyon Fm (Lerbekmo, 2002)

**73.5 +/- 0.4** Ma (Rb-Sr; biotite; Lerbekmo, 2002)

**Variable cited age**

Lerbekmo & Braman (2002) give an age of 73.2 Ma for the Dorothy Bentonite (citing H. Baadsgard and JF Lerbekmo, unpublished data). However, the Dorothy Bentonite is described in more detail in Lerbekmo (2002), where he gives a date of 73.5 +/- 0.4 Ma (H. Baadsgard, pers. comm. 1999), and states that this is a Rb-Sr date, based on purified biotite.

Both Lerbekmo & Braman (2002) and Lerbekmo (2002) were published in the Canadian Journal of Earth Sciences. From the submission / acceptance dates it is likely that the second date is more accurate (73.5 +/- 0.4 Ma, Lerbekmo, 2002), since Lerbekmo (2002) was submitted 28 January 2002, accepted 13 August 2002; and Lerbekmo & Braman (2002) was submitted 19th June 2001, accepted 25th October 2001. Hence here I use the more recently published 73.5Ma date (compared to Koppelhaus and Braman, 2010, who use the slightly older 73.2 Ma date), although if the pers. comm. was in 1999 (as Lerbekmo 2002 stated) then it should have been available for citing in the Lerbekmo & Braman (2002) article.

**Stratigraphy**

Eberth & Braman (2012) show that in surface exposures (supp. info., section #1) the Dorothy bentonite occurs in the upper tongue of the Bearpaw Formation, ~46m below the base of the Drumheller Mbr of the Horseshoe Canyon Fm, just above the uppermost part of the Strathmore Mbr of the Horseshoe Canyon Fm, thereby constraining the age of the Strathmore tongue (which is only known from subsurface analysis).

**Age - ammonites & magnetostratigraphy issues**

There are issues with the new standardized dates provided by Ogg & Hinnov (2012) in the Geological Timescale (Gradstein et al., 2012), mainly concerning the position of the *B. cuneatus* ammonite zone relative to magnetochron C32r and the position of the Dorothy bentonite.

Lerbekmo & Braman (2002, 2005) and Lerbekmo et al. (2003) show the Dorothy bentonite occurring within C33n.1n (compare fig. 2 of Lerbekmo, 2002, with fig. 14 of Lerbekmo & Braman 2002). They then state that Tsujita (1995) recovered the ammonite *Baculites cuneatus* from 5 m above the Dorothy bentonite, and thus they illustrate the Dorothy bentonite as just below or within the *B. cuneatus* ammonite zone.

If the identification of *B. cuneatus* just above the Dorothy bentonite is correct, then we would expect the age of the base of the *B. cuneatus* zone (73.91 Ma; Ogg & Hinnov, 2012) to be roughly the same age or slightly younger than the Dorothy bentonite (Rb-Sr date of 73.5 +/- 0.4 Ma; see above). Taking into account the error in the Rb-Sr date, the ages are close to what would be expected, but it is therefore suspected (at least using the new definition of the *B. cunratus* zone) that the Dorothy bentonite is probably closer to 73.9 Ma in age.

The magnetostratigraphy is more problematic. If the base of *B. cuneatus* truly occurs in the uppermost C33n (C33n.1n), then this is a problem as the new ages of the top of the top of C33n (74.309 Ma; Ogg, 2012) and the base of *B. cuneatus* zone (73.91 Ma; duration 0.28 m.y.; Ogg & Hinnov, 2012), do not overlap.

----

### Strathmore

**Strathmore Mbr**

The Strathmore Mbr is a tongue of coastal to shallow marine sediments which has been studied almost entirely in the subsurface due to lack of outcrop (Eberth & Braman, 2012).

**Age**

The Strathmore Mbr is shown to occur within the upper C33n by Eberth & Braman (2012), which I follow here.

----

### BEARPAW Fm

**Bearpaw Shale**

The Bearpaw Shale is a marine shale which occurs across northern US and southern Canada. In Canadian sections, thickness is at its maximum in Saskatchewan where it is up to 350 m, but decreases westwardly where it's subdivided and variably intertongues with the Horseshoe Canyon Fm (Braman et al., 1999).

The upper contact is variable geographically. In the east and south the Drumheller Mbr of the Horseshoe Canyon Fm interfingers or rests sharply on the Bearpaw Fm (Eberth & Braman, 2012). In the north and west the base of the Drumheller Mbr conformably rests on coaly deposits of the Strathmore Mbr, with the contact between the two equivalent to the maximum flooding surface of the lower tongue of the Bearpaw Fm (Eberth & Braman, 2012).

The maximum flooding surface is thought to occur within the *Baculites compressus* zone (Obradovich, 1993; 74.21 - 73.91 Ma; Ogg & Hinnov, 2012), although this may not ally completely with the latest stratigraphic work (see Eberth & Braman, 2012).

The basal contact of the Bearpaw Fm with the underlying Dinosaur Park Fm is considered conformable (e.g. Braman et al., 1999; Eberth, 2005).

**Age**

Age of the upper contact is variable dependent on geographic position. In outcrop around the town of Drumheller, the base of the Drumheller Mbr falls within C32r.1r, but becomes progressively older westwards such that it occurs in the upper part of C33n (Lerbekmo & Braman, 2002, 2005; Lerbekmo & Lehtola, 2011; Eberth & Braman, 2012). Here I follow the stratigraphic positioning of Eberth & Braman (2012).

Ammonite fragments from the "Finnegan Sandstone" in the lower half of the Bearpaw Shale were identified as *Baculites compressus* (*robinsoni*) by Tsujita (1995; Eberth, 2005).

The lower contact of the Bearpaw Fm with the underlying Lethbridge Coal Zone of the Dinosaur Park Fm occurs within the upper third of C33n (Lerbekmo & Braman, 2002). This is more precisely identified as C33n.3n by Lerbekmo (2005). This placement is consistent with Ar / Ar dates of 75.46 Ma occurring ~8m above the Dinosaur Park Fm-Bearpaw contact, and 76.10 Ma which occurs ~14 m below the contact (see individual entries).

----

### 75.46 ± 0.24

**"Bearpaw Tuff"**

(Eberth, 2011: Feb 4th public Lecture, Tyrell Museum; pers. comm. 2017; in prep.)

83.25m above Oldman-DPFm contact (8m above DPFm-Bearpaw contact at 75.25m)

75.46 +/- 0.24 Ma (Ar/Ar, sanidine, n=13)

(other dates)

73.34 +/- 0.66 Ma (Ar/Ar, plagioclase, n=3)

74.19 +/- 0.94 Ma (K/Ar, biotite, n=1)

**Standard**

Sanidine from the Fish Canyon Tuff, reference age of 28.201 Ma (Kuiper et al., 2008).

Older notes:

Eberth (2005) cites Eberth et al. (1992) and Eberth & Deino (1992) as the sources for a radiometric date of 74.8 Ma for the base of the Bearpaw Fm. .

----

### BELLY RIVER Gp

**Belly River Group**

The Belly River Group is a mostly non-marine clastic wedge comprising the Foremost, Oldman, and Dinosaur Park Formations (Eberth, 2005). Accounts of each formation can be found in their individual entries.

There is a fairly complex history to the various terms used to refer to what is now known as the Belly River Group of southern Alberta; a detailed account is provided by Eberth (2005). In summary, until 1996, the Belly River Gp of southern Alberta was most typically referred to as the "Judith River" Formation or Group, as it is stratigraphically equivalent (or nearly so) with the Judith River Fm of Montana. However, common usage changed to Belly River Group after the work of Jerzykiewicz and Norris (1994), and Hamblin and Abrahamson (1996).

----

### DINO PARK Fm

**Dinosaur Park Fm**

Approximately 75m thick, the Dinosaur Park Fm is divided into three zones: the basal "sandy zone" (~40 m), the middle "muddy zone" (~15 m), and is capped by the "Lethbridge coal zone" (~15 m; Eberth, 2005).

The upper contact with the overlying marine Bearpaw Fm is conformable (Eberth, 2005). However, the lower contact with the underlying Oldman Fm is disconformable and diachronous, becoming younger to the south (Eberth, 2005).

Some stratigraphic complexity occurs in the Lethbridge Coal Zone where channels from within the Lethbridge Coal Zone incise down through the lowermost coal, into the top of the muddy zone (Ryan et al., 2010b). Therefore, paleontologists must be cautious when assessing fossil remains from the upper parts of the Dinosaur Park Fm.

**Age**

Age of the Dinosaur Park Fm is constrained by magnetostratigraphy and radiometric dates. The Dinosaur Park Fm is almost entirely of normal polarity, with the exception of a short reversed polarity section ~4 m thick that occurs at ~23 m from the base of the formation (Lerbekmo, 2005). The formation is therefore assigned to C33n.3n, C33n. 3r and C33n.4n (Lerbekmo, 2005).

Ar / Ar dates strongly constrain the age of the Dinosaur Park Fm. A date of 77.03 Ma occurs in the uppermost Oldman Fm, 5.5 m below the formational boundary; two dates (76.39 Ma, 76.10 Ma) occur within the Dinosaur Park Fm itself, and a date of 75.46 Ma has been recovered from the overlying Bearpaw Fm (see individual entries).

----

### 76.10 ± 0.5

**"LCZ Tuff"**

(Eberth, 2011: Feb 4th public Lecture, Tyrell Museum; pers. comm. 2017; in prep.)

61.5m above Oldman-DPFm contact (LCZ starts at 58m)

76.10 +/- 0.5 Ma (Ar/Ar, sanidine, n=2)

**Standard**

Sanidine from the Fish Canyon Tuff, reference age of 28.201 Ma (Kuiper et al., 2008).

----

### 76.39

**"Plateau Tuff"**

(Eberth, 2011: Feb 4th public Lecture, Tyrell Museum; pers. comm. 2017; in prep.)

36m above Oldman-DPFm contact

76.39 Ma (weighted mean)

(samples)

76.06 +/- 0.42 Ma (Ar/Ar, sanidine, n=10)

76.29 +/- 0.42 Ma (Ar/Ar, sanidine, n=11)

77.08 +/- 0.22 Ma (Ar/Ar, sanidine, n=6)

(other dates)

77.39 +/- 0.21 Ma (Ar/Ar, biotite, n=6)

75.05 +/- 0.4? Ma (K/Ar, biotite, n=3); may be same date as Horner & Currie (1994) suggested for the Alberta exposure of the Two Medicine Formation (renamed the Oldman Formation in Alberta). This date from Eberth & Deino (1992, abstract)

**Standard**

Sanidine from the Fish Canyon Tuff, reference age of 28.201 Ma (Kuiper et al., 2008).

**Notes**:

Eberth (2005) cites Thomas et al (1990) as having published a radiometric date of 76.1 Ma derived from a Ar / Ar dating of a bentonite from the middle of the DPFm (given by Thomas et al., 1990, as the Plateau Tuff, 76.11 +/- 0.2 Ma), and states without further reference that this has now been revised to 75.3 Ma. Eberth (2005) shows the 75.3 Ma date as approximately 25-30m from the top of the DPFm (~40m from the basal contact with the underlying Oldman Fm), occurring in the basalmost part of the "muddy zone". Thomas et al. (1990) show the Plateau Tuff as occurring 25m below the contact with the Bearpaw Shale. Thomas et al. (1990, including Eberth as a co-author) go to some lengths to suggest that their 76.1 Ma date is more accurate than the date of Eberth et al. (in press, presumably 75.3 Ma).

----

### OLDMAN Fm

**Oldman Fm**

The Oldman Fm comprises ~40m of terrestrial sandstones, mudstones, and thin coals (Eberth, 2005), although it can reach up to ~120 m thickness in southernmost Alberta (Ryan, 2003). The Oldman Fm was sourced sedimentologically in what is now Montana, contrasting with the overlying Dinosaur Park Fm, which was sourced in what is now northern Alberta (Eberth & Hamblin, 1993; Eberth, 2005).

The Oldman Fm was deposited during an overall regressive phase of the Bearpaw seaway. Eberth (2005) notes the maximum regression identified in the lower part of the Oldman Fm probably corresponds to the eustatic drop noted by Haq (1988) at ~77.5 Ma (this date is now probably ~1 + Ma older due to recalibration of radiometric dating standards).

The Oldman Fm is subdivided into three units, with a considerable hiatus probably existing between deposition of Unit 1 ("lower muddy") and Unit 2 (Comrey Sandstone). Most typical references to the Oldman Fm refer to exposures in Dinosaur Provincial Park, Alberta, as this is where most previous work has been conducted, but which only exposes outcrop referable to units 2 and 3.

The lower contact with the Foremost Fm is either conformable, or represents a short hiatus, depending on the unit defined as the uppermost part of the Foremost Fm. The Herronton sandstone (which immediately underlies Unit 1 of the Oldman Fm) is thought to be lithostratigraphically allied to the Foremost Fm (Eberth, 2005), but may represent the initial basal amalgamated sandstone part of the depositional cycle of which Oldman Fm Unit 1 is the uppermost part (as shown here). It is likely that a short hiatus occurs at the base of the Herronton sandstone, which then conformably grades into the fine mudstones of the basal Oldman Fm, Unit 1.

The upper contact with the overlying Dinosaur Park Fm is disconformable and diachronous, becoming younger to the south (Eberth, 2005).

**Age**

The upper contact is constrained by a date of 77.03 Ma which occurs in the uppermost Oldman Fm, 5.5 m below the formational boundary; two dates (76.39 Ma, 76.10 Ma) occur within the Dinosaur Park Fm itself; and a date of 75.46 Ma has been recovered from the overlying Bearpaw Fm (see individual entries).

Age of the lower contact is not well constrained in Alberta. However, in the Judith River Fm of Rudyard, MT (~2 km south of the US-Canada border), Ar / Ar dates have been recovered either side of the surface equivalent to the lower contact. A date of 79.52 Ma was recovered 4.8 m below the marker A coal (uppermost Taber coal zone, immediately below the Herronton Sandstone equivalent), and a date of 79.22 Ma was recovered 27 m above the top of Marker A coal approximately halfway through the equivalent to Oldman Fm Unit 1 (Goodwin & Deino, 1989; recalibrated, Fowler, this article).

----

### Unit 2 & 3

**Unit 2 & 3:**

Also known as the Comrey Sandstone (unit 2) and Upper Muddy (unit 3).

----

### 77.03

**"Field Station Tuff"**

(Eberth, 2011; Feb 4th, Tyrell Museum public lecture, pers. comm. 2017; in prep.).

5.5m below Oldman-DPFm contact

77.03 Ma (weighted mean)

(samples)

76.9 +/- 0.? Ma (Ar/Ar, sanidine, n=?)

76.9 +/- 0.? Ma (Ar/Ar, sanidine, n=?)

77.49 +/- 0.4? Ma (Ar/Ar, sanidine, n=?)

**Standard**

Sanidine from the Fish Canyon Tuff, reference age of 28.201 Ma (Kuiper et al., 2008).

Older notes:

Eberth (2005) cites Eberth et al. (1992) and Eberth & Deino (1992, an abstract) as the sources for a radiometric date of 76.5 Ma for the top of the Oldman Fm. The radiometric date is shown as occurring approximately 5m below the DPFm-Oldman contact in the generalised section of Eberth (2005).

Eberth and Hamblin (1993) provide a date of 76.5 +/- 0.5 Ma for a bentonite found 4m below the Oldman-DPFm discontinuity in Dinosaur Provincal Park. This may potentially be the same horizon as mentioned above, although three bentonite horizons are recorded at this level by Eberth et al. (1992).

----

### 77.09

**"Canal Creek Tuff"**

(Chiba et al., 2015).

Milk River area

77.09 Ma +/- 0.2 Ma (Ar/Ar, unspecified mineral)

The Canal Creek Tuff occurs within the upper Oldman Fm in southern Alberta, near the Milk River. This is thought to be time-equivalent to the lower part of the Dinosaur Park Formation in Dinosaur Park itself (Chiba et al., 2015). The tuff occurs ~4m above the McPheeters boned, from which remains of the ceratopsid dinosaur Centrosaurus apertus have been recovered (Chiba et al., 2015)

**Standard**

Not specified by Chiba et al. (2015) but assumed to be the Fish Canyon Tuff, reference age of 28.201 Ma (Kuiper et al., 2008).

----

### Unit 1

**Oldman Fm, Unit 1 (Eberth, 2005)**

Sometimes referred to as "lower muddy," or the "lower Oldman Fm."

Unit 1 comprises non-marine mudstones, sandstones and discontinuous coals (Eberth, 2005) that occur immediately above the Herronton sandstone (considered the uppermost unit of the Foremost Fm by Eberth, 2005).

**Stratigraphy**

Age of Unit 1 is constrained by radiometric dates acquired from equivalent strata exposed in Kennedy Coulee, Northern Montana (classified as the lower Judith River Fm; Goodwin and Deino, 1989; see individual entries).

A depositional hiatus occurs between the top of unit 1 and the base of unit 2 (Comrey sst), representing the regression of the Western Interior Seaway. This is represented here by the double headed arrow. The duration of this hiatus is uncertain and only constrained by the bounding radiometric dates.

----

### FOREMOST Fm

**Foremost Fm**

~170 m thick (almost all in the subsurface), paralic to nonmarine sandstones, shales, and coals (Eberth, 2005). The Foremost Fm gradationally overlies marine shales of the underlying Pakowki Fm, and interfingers with the Pakowki to the east. Coarsening upwards units representing progradational and aggradational stacking patterns capped by coal zones; the McKay coal zone occurs ~30 m from the base, and the Taber coal zone ~ 15 m below the base of the Herronton sandstone. Eberth (2005; pers. comm.) considers the Herronton sandstone as the uppermost unit of the Foremost Fm, based on petrological similarity to underlying sandstones; however, it is likely that the Herronton sandstone is the basal amalgamated channel deposit of the overlying Oldman Fm (specifically, "unit 1").

**Age**

The age of the Foremost Fm is constrained by radiometric dates from near the top and bottom of the unit (including a date from the equivalent horizon in Montana). However, the lower date conflicts slightly with ammonite biostratigraphy and magnetostratigraphy.

**Upper contact**

The upper contact is not well dated in Alberta, but a radiometric date of 79.52 +/- 0.2 Ma was recovered in the equivalent part of the Judith River Fm, 4.8 m below the marker A coal (uppermost Taber coal zone), ~1 mile south of the US-Canada border at Kennedy Coulee, Montana (Goodwin & Deino, 1989; recalibrated, Fowler, this article). It is likely that a short hiatus occurs at the base of the Herronton sandstone (overlying the Taber coal zone), which then conformably grades into the fine mudstones of the basal Oldman Fm, Unit 1.

**Lower contact**

Lerbekmo (1989; and Leahy & Lerbekmo, 1995) show the basal contact of the Foremost Fm with the underlying Pakowki Fm occurring in the uppermost C33r, with C33n occurring <10m above. This is problematic as the radiometric date recovered ~30 m above the base of the Foremost is 80.17 +/- 0.15 Ma (Eberth, 2005; recalibrated; see individual entry); this should be within C33n according to Lerbekmo (1989 and Lerbekmo & Leahy, 1995), but the base of C33n is defined as 79.900 by Ogg (2012), i.e. above the radiometric date.

The radiometric date may also cause issue with proposed ammonite biostratigraphy. Eberth (2005) suggests that the base of the Foremost Fm is correlative with the *Baculites asperiformis* zone (although no reference is given for an ammonite occurrence in Alberta that corroborates this, this relationship is shown in Leahy & Lerbekmo, 1995). Since Ogg & Hinnov (2012) define the base of the *B. asperiformis* zone as 80.21 Ma, and that the radiometric date from ~30 m above the base of the Foremost is 80.17 Ma (Eberth, 2005; see above), then this suggests that the lowermost 30 m of the Foremost Fm were deposited in a mere 40,000 years. This discrepancy may be explained if (as suspected; Leahy & Lerbekmo, 1995) *B. asperiformis* occurs slightly earlier in Canadian sections than is typical.

From both the above examples, it might therefore be expected for the radiometric date to be slightly younger. This issue remains unresolved.

----

80.17 ± 0.15

**Eberth (2005)**

Unnamed horizon

Altered volcanic ash, 30m above the base of the ~170 m thick Foremost Fm from a cored well 10 km south of Dinosaur Provincial Park

79.14 +/- 0.15 Ma (unspecified error type); (Ar / Ar ; Eberth, 2005, cites Deino, pers. comm., 1993)

80.17 +/- 0.15 Ma (1σ); (recalibration; this article; see below)

Eberth (2005) cites Deino (pers. comm. 1993) for a date of 79.14 +/- 0.15 Ma, for an "altered volcanic ash" recovered 30 m above the base of the Foremost Fm (170 m thick) from a cored well 10 km south of Dinosaur Provincial Park. This is an Ar / Ar date, and the same date is given in an abstract by Eberth & Deino (1992).

**Standard**

The standards used are not mentioned by Eberth 2005, nor by Eberth & Deino (1992), but in 1993 (the date of the pers. comm.) the FCT age used as standard in the industry was 27.84 Ma (Samson & Alexander, 1987; Renne et al., 1998), and this was the age used in previous radiometric analyses performed by Deino (e.g. Goodwin & Deino, 1989; Deino & Potts, 1990' Thomas et al., 1990). The decay constant used should be λT = 5.543 E-10/y (Steiger & Jaeger, 1977), as this was standard at the time, and did not change until Min et al. (2000). Furthermore, when referring to the Ar/Ar dates of Eberth & Deino (1992) and Eberth et al. (1992), Eberth & Hamblin (1993) refer the reader to Thomas et al. (1990) for the methodology of the analysis.

**Recalibration (Fowler, this article)**

Legacy dates; FCT at 27.84 (Samson & Alexander, 1987; Renne et al., 1998); legacy λT at 5.543 +/- 0.010 E-10/y (Steiger & Jaeger, 1977).

78.2 +/- 0.2 Ma (1σ); (Ar / Ar, sanidine, 4 crystals, ; Goodwin & Deino, 1989)

1st recalibration; FCT at 28.201 +/- 0.023 Ma (1σ; Kuiper et al., 2008), and λT at 5.463 E-10/y +/- 1.07 E-11/y (1σ; Min et al., 2000)

80.168 +/- 0.152 Ma (unknown error type); (recalibration, this article)

2nd recalibration (for reference); FCT at 28.294 +/- 0.294 Ma (1σ), and λT at 5.531 E-10/y +/- 1.35 E-12/y (1σ; both Renne et al., 2011).

80.415 +/- 0.152 Ma (unknown error type); (recalibration, this article)

----

# SASKATCHEWAN

## SW Plains

### FRENCHMAN Fm

**Frenchman Fm**

The Frenchman Fm is the Saskatchewan lithostratigraphic equivalent of the Lower Scollard Fm in Alberta, comprising 6-75 m (depending on underlying topography) of terrestrial channel sandstones and mudstones (Braman et al., 1999). Together the Lower Scollard and Frenchman form an eastward thinning clastic wedge (Dawson et al., 1994b).

**Age**

The upper contact of the Frenchman Fm is conformable with the overlying Ravenscrag Fm and is placed at or near the base of the Ferris coal, which generally marks the K-Pg boundary (Braman et al., 1999). In SW Saskatchewan, the K-Pg boundary occurs at the contact between the Frenchman Fm and the overlying Ravenscrag Fm (Lerbekmo ,1999; Lerbekmo & Braman, 2002; Lerbekmo, 2009). However, generally, as we move east through Saskatchewan the contact becomes an erosional disconformity, removing the K-Pg boundary (Lerbekmo, 1999).

The Frenchman Fm has an erosive contact with the underlying Battle Fm (Dawson et al., 1994), and can be shown to be younger at its base than the Scollard (Lerbekmo, 1999; Lerbekmo & Braman, 2002).

The base of the Frenchman Fm is illustrated as occupying the uppermost C30n magnetozone, with the main body residing in C29r (Lerbekmo & Braman, 2002; Lerbekmo, 1999). However, this is mainly due to the convention of drawing the boundary between magnetochrons as occurring halfway between the normal and reversed samples. In reality, the lowermost measured sample from the Frenchman Fm is of reversed polarity in all sampled sections except the Wood Mountain core (Lerbekmo, 1999) where a sample taken from the basal sandstone of the Frenchman Fm is of normal polarity. A small normal submagnetochron within C29r was detected at the top of the Frenchman Fm by Lerbekmo (1999), although this has not yet been noted by other workers.

----

### BATTLE Fm

**Battle Fm**

Description and stratigraphy of the Battle Fm in Saskatchewan follows that of the Whitemud in Alberta (see left).

----

### WHITEMUD Fm

**Whitemud Fm**

Description and stratigraphy of the Whitemud in Saskatchewan follows that of the Whitemud in Alberta (see left). However, it should be noted that Catuneanu & Sweet (1999) showed a diachronous relationship where Saskatchewan outcrop of the Whitemud was younger than in Alberta. This relationship was not noted by later workers and is not shown here.

Note that in Saskatchewan, the Whitemud is afforded Formation rank, whereas in Alberta it is a Member of the Horseshoe Canyon Fm.

----

### EASTEND Fm

**Eastend Fm**

The Eastend Fm comprises ~20-35 m of mixed marine and nonmarine sandstones and shales (Braman et al., 1999).

**Age**

Palynomorphs suggest the Eastend Fm was deposited during the earliest part of the Late Maastrichtian (Braman & Sweet, 1999).

Magnetostratigraphy shows that the Eastend Fm was deposited from C31n through to C30n, although this is geographically variable. Typically, the upper part of the Eastend Fm is of reversed polarity, assigned to C31r (Lerbekmo, 1985; Lerbemo & Braman, 2002), although in some rare sections the uppermost Eastend includes normal polarity beds assigned to C30n (Lerbekmo, 1999). The majority of the thickness of the Eastend Fm is of normal polarity, assigned to C31n (Lerbekmo, 1985; Lerbekmo & Braman, 2002).

----

### BEARPAW Fm

**Bearpaw Fm**

The Bearpaw comprises up to 350 m of marine shales, siltstone, and sandstone (Braman et al., 1999).

Contact with the overlying Eastend Fm is transitional, with the lower contact with the Dinosaur Park Fm is conformable (Braman et al., 1999).

**Age**

Only the upper part of the Bearpaw Fm is shown here, where the contact between the Bearpaw and overlying Eastend Fm is plotted within the lowermost part of C31n, as shown by Lerbekmo & Braman (2002).

The lower contact of the Bearpaw with limited exposures of the Dinosaur Park Formation (not plotted here) is shown occurring within C33n by Lerbekmo & Braman (2002).

# MONTANA

## West

### St MARY RIVER Fm

**St Mary River Fm, MT**

The St. Mary River Fm outcrops in central to northern Montana, USA, and southern Alberta, Canada and was deposited in an entirely terrestrial environmental setting, being a more landward equivalent of the Horseshoe Canyon Fm (Hamblin, 1998).

**Age**

Here I have simply copied across the stratigraphic range from the Canadian sections (Brinkman, 2003) as the Montana sections are not well-studied.

----

### HORSETHIEF Fm

**Horsethief Fm**

The Horsethief Fm is a shallow marine sandstone facies deposited during the regression of the Bearpaw Seaway. Although lithostratigraphically equivalent to the Fox Hills Fm in Eastern Montana, the Horsethief was deposited earlier representing the initial phase of the Fox Hills regression (Gill & Cobban, 1973).

**Age**

Stratigraphy of the Horsethief Fm in Montana is constrained by ammonite biostratigraphy (Gill & Cobban, 1973), and magnetostratigraphy of the correlative Blood Reserve Fm in Alberta (Lerbekmo & Lehtola, 2011).

Gill & Cobban (1973) illustrate the initial phase of the Fox Hills regression occurring during the *Baculites compressus* through *B. grandis* ammonite zones (74.21 - 70.44 Ma; Ogg & Hinnov, 2012), during which the strandline slowly migrates eastwards from the thrust front in western Montana, over a distance of ~150 km. The Horsethief Fm is the time transgressive unit deposited during this time. In their sampled section in southwestern Alberta, Lerbekmo & Lehtola (2011) show the base of the correlative Blood Reserve Fm as occurring during C32n.3n; this would place it around 71.8 Ma, within the *B. baculus* zone.

Age of the base of the overlying St. Mary River Fm is not well understood in Montana, but the base of the correlative unit in southwestern Alberta (also called the St. Mary River Fm) was placed as C32n.1r by Lerbekmo & Lehtola (2011), which is ~71.5 Ma. As a regressive sandstone, the Horsethief Fm is expected to be time transgressive at both its base and top, becoming younger to the east. As such a hiatus is expected between the top of the Horsethief and the overlying St, Mary River Fm.

Thus, here I have plotted the Horsethief as being time transgressive from the *B. compressus* zone through to the base of the overlying St. Mary River.

----

### TWO MEDICINE Fm

**Two Medicine Fm, MT**

Maximum thickness of Two Medicine Fm variable (~550m; Foreman et al., 2008; estimated as 650m by Horner & Currie, 1994; 410 m in the type area; Rogers, 1998; suggested as 600-1500 m by Shelton, 2007).

The Two Medicine Fm is an important unit, but its understanding is clouded by a stratigraphic complexity caused by geographic variability, patchy outcrop, and variable tectonic disruption. As such, a single generalized section of the Two Medicine Formation has not been published, although some approximations are given by Lorenz & Gavin (1984); Rogers (1994; 1998); Roberts (1999); Horner et al. (2001); Shelton (2007; Appendix C, unpublished; which is probably the best single reference figure), and Foreman et al. (2008). Definitions and names of internal units, stratigraphic boundaries, marker beds, and radiometrically dated horizons, vary between publications, and are not always cross referenced, making keeping track difficult. Here I have tried to give a reasonable approximation of the unit, but the reader should be aware that some boundaries may be time transgressive, and stratigraphic correlations between sections are still tentative.

**Subdivisions & discontinuities**

Horner et al. (2001) subdivide the Two Medicine Fm into five lithofaces based on the lithofacies defined by Lorenz & Gavin (1984). These lithofacies are generally not used explicitly by other workers (e.g. Rogers, 1998; Shelton, 2007), although the criteria upon which the boundaries are based are maintained. I have chosen to show the lithofacies here as they are convenient and facilitate easy reference. See individual entries for details of their stratigraphic positions.

Rogers (1994, 1998) describes two discontinuities within the Two Medicine Fm, correlating them west across into the Judith River Fm. The Lower Discontinuity (~81.1 Ma; recalibrated from Rogers et al., 1993) between lithofacies 2 and 3, represents the nonmarine expression of a fall in sea level associated with the maximum regression (R7) of the Colorado Shale (Rogers, 1994; 1998). The Upper Discontinuity (~77-78 Ma), between lithofacies 3 and 4, represents the nonmarine expression of the onset of transgression of the Bearpaw Seaway (Rogers, 1994), thought to be correlative with either the top of the Comrey sandstone (Oldman Fm, Alberta) at ~78 Ma, or the Oldman-Dinosaur Park Fm (DPFm) discontinuity which is ~77 Ma (Rogers, 1994, 1998; Ryan, 2002).

**Age**: Radiometric dates bracket the Two Medicine Fm as ~81.5 - ~75 Ma (Rogers et al., 1993; recalibrated by Fowler, this article).

**Lower contact**: The Two Medicine Fm overlies the Virgelle Fm, a marine sandstone that records regression of the Western Interior Seaway (R7; Rogers, 1998). The Virgelle is time transgressive, but the ammonite *Scaphites hippocrepis II* occurs within it (Gill & Cobban, 1973; Sageman et al., 2014), making the Virgelle no older than 82.00 Ma (Ogg & Hinnov, 2012). Radiometric dates retrieved by Rogers et al. (1993; recalibrated here) show the lower disconformity occurring ~ 81.1 Ma, suggesting that the base of the Two Medicine Fm occurs between 81.1 and 82 Ma.

**Upper contact**: The upper contact of the Two Medicine Fm is regionally variable, being overlain by either the marine Bearpaw Shale, or the shallow marine Horsethief Sandstone (Lorenz & Gavin, 1984). A radiometric date of 75.04 Ma (recalibrated from Rogers et al., 1993) from ~10 m below the top of the Two Medicine Fm gives an approximation for the age of the upper contact.

**Conflicting radiometric dates & sequence stratigraphy**

Some issues exist with radiometric dates when comparing both within the Two Medicine Fm, and between the Two Medicine and stratigraphic equivalents, making comparisons difficult. There are ~ 10 horizons within the Two Medicine Fm that have been Ar /Ar dated. However, they do not all use the same subject minerals. The preferred mineral, sanidine, was used for only two horizons (TM-4; Rogers et al., 1993; HH; Foreman et al., 2008), with all other dates using the less desirable biotite or plagioclase. This is possibly the source of an issue where stratigraphically successive horizons have inconsistent dates. For example, the plagioclase date for bentonite TM-6, ~10 m below the top of the formation, is 75.04 Ma, whereas the sanidine date for the underlying bentonite TM-4, ~60 m lower in section, is 75.03 Ma (i.e. younger). By comparison, the plagioclase date for TM-6 is 75.235 Ma (i.e. older, as expected). As such, comparisons within the Two Medicine Fm may best be made using plagioclase dates. However, sanidine is used for almost all radiometric dates in other formations. This might be the source of a problem with the age of the Upper Discontinuity, where a plagioclase date of 76.39 Ma from ~10.7 m below the discontinuity (recalibrated; Varricchio et al., 2010) is too young for either of the proposed correlations (top of the Comrey sandstone, Oldman Fm, Alberta; ~78 Ma; or Oldman-DPFm discontinuity, ~77 Ma). By comparison, a sanidine date of 78.03 Ma from ~65 m below the upper discontinuity (recalibrated; Foreman et al., 2008) would be consistent with either proposed correlation (although a better fit for the Oldman-DPFm).

**Note**: Foreman et al. (2008; p.374) suggest that bentonites in the Mesa Verde (WY) and Kaiparowits Fms (UT) are "at least coeval with, if not derived from the same eruptions" as bentonite horizons in the Two Medicine Fm. However, current values for recalibrated dates between the Two Medicine and Kaiparowits Fms and are not particularly similar, although they certainly overlap. This may be due to differing methodologies, but this remains to be tested.

----

### lithofacies 5

**Lithofacies 5**

Horner et al. (2001) describe lithofacies 5 as "all Two Medicine strata above the lacustrine carbonate sequences [lithofacies 4]".

----

### ( 75.04 ± 0.10 )

**Rogers et al. (1993); recalibration, Fowler (this article)**

TM-6

~10 m below top of Two Medicine Fm (Rogers et al., 1993)

~540 m above base of Two Medicine Fm (Foreman et al., 2008)

74.076 +/-0.095 Ma (1σ); or +/- 0.048 (1 SE); (Ar / Ar, plagioclase, 4 x 1 crystal; Rogers et al., 1993)

75.038 +/- 0.10 Ma (1σ); (plagioclase, recalibration, this article; see below)

The date is shown in parentheses as the plagioclase date of TM-6 (75.04 +/- 0.1 Ma ) is actually slightly older than the sanidine date for TM-4 (75.03 +/- 0.73 Ma; which is ~130 m lower in section), but younger than the plagioclase date for TM-4 (75.235 +/- 0.159 Ma). Although dates derived from sanidine are preferable in Ar / Ar analyses, it may be better to use plagioclase dates for comparison between bentonites TM-4 and TM-6.

**Standard**

Rogers et al. (1993) use the FCT at 27.84 Ma (Cebula et al., 1986) "intercalibrated in-house" with MMhb-I at 520.4 Ma (Samson & Alexander, 1987). Decay constant (λT) follows Steiger & Jaeger (1977), at 5.543 +/- 0.010 E-10/y.

Variation in reporting (Rogers et al., 1993) & consequent citation mistakes (Roberts et al., 2013)

Within the text body (p. 1071-1072; but not their data table, p1070-1071), Rogers et al. (1993) report most of their new dates using standard error (SE) rather than standard deviation 1σ (although 1σ is necessarily cited on single crystal analyses of sanidine and anorthoclase for TM-4). These measures are directly related, but not directly comparable. In their recalibration table of various Western Interior radiometric dates, Roberts et al. (2013; table 6.1) state directly the ages and error from the Rogers et al. (1993) body text, thus mixing 1σ values alongside SE without indicating which is which, or noting the incomparability of these values.

**Recalibration (Fowler, this article)**

Legacy dates; FCT at 27.84 (Samson & Alexander, 1987; Renne et al., 1998); legacy λT at 5.543 +/- 0.010 E-10/y (Steiger & Jaeger, 1977).

74.076 +/-0.095 Ma (1σ); or +/- 0.048 (1 SE); (Ar / Ar, plagioclase, 4 x 1 crystal; Rogers et al., 1993)

73.568 +/- 2.546 Ma (1σ); or +/- 1.139 (1 SE); (Ar / Ar, biotite, 5 x 1 crystal; Rogers et al., 1993)

75.376 +/- 0.384 Ma (1σ); (Ar / Ar, anorthoclase?, 1 crystal: no. 3572-01; Rogers et al., 1993)

76.586 +/- 2.966 Ma (1σ); (Ar / Ar, plagioclase, 1 crystal: no. 3572-02; Rogers et al., 1993)

1st recalibration; FCT at 28.201 +/- 0.023 Ma (1σ; Kuiper et al, 2008), and λT at 5.463 E-10/y +/- 1.07 E-11/y (1σ; Min et al., 2000)

75.038 +/- 0.10 Ma (1σ); (plagioclase)

74.523 +/- 2.58 Ma (1σ); (biotite)

76.355 +/- 0.384 Ma (1σ); (anorthoclase? 3572-01)

77.581 +/- 2.966 Ma (1σ); (plagioclase 3572-02)l

2nd recalibration (for reference); FCT at 28.294 +/- 0.294 Ma (1σ), and λT at 5.531 E-10/y +/- 1.35 E-12/y (1σ; both Renne et al. 2011).

75.271 +/- 0.097 Ma (1σ); (plagioclase)

74.755 +/- 2.586 Ma (1σ); (biotite)

76.591 +/- 0.390 Ma (1σ); (anorthoclase? 3572-01)

77.820 +/- 3.013 Ma (1σ); (plagioclase 3572-02)

----

### 75.03 ± 0.73

**Rogers et al. (1993); recalibration, Fowler (this article)**

TM-4

~480m above base of Two Medicine Fm

74.066 +/- 0.722 Ma (1σ); (Ar / Ar,sanidine, 1 crystal; Rogers et al., 1993)

74.270 +/-0.157 Ma (1σ); (Ar / Ar, plagioclase, 4 x 1 crystal; Rogers et al., 1993)

75.03 +/- 0. Ma (sanidine, Roberts et al., 2013)

75.028 +/- 0.731 Ma (1σ); (sanidine, recalibration, this article; see below)

75.235 +/- 0.159 Ma (1σ); (plagioclase, recalibration, this article; see below)

Although dates derived from sanidine are preferable in Ar / Ar analyses, it may be better to use plagioclase dates (see below) for comparison between bentonites TM-4 and TM-6 (which is ~130 m higher in section), since the plagioclase date of TM-6 (75.04 +/- 0.1 Ma ) is actually slightly older than the sanidine date for TM-4 (75.03 +/- 0.73 Ma), but younger than the plagioclase date for TM-4 (75.235 +/- 0.159 Ma).

**Standard**

Rogers et al. (1993) use the FCT at 27.84 Ma (Cebula et al., 1986) "intercalibrated in-house" with MMhb-I at 520.4 Ma (Samson & Alexander, 1987). Decay constant (λT) follows Steiger & Jaeger (1977), at 5.543 +/- 0.010 E-10/y.

Variation in reporting (Rogers et al., 1993) & consequent citation mistakes (Roberts et al., 2013)

Within the text body (p. 1071-1072; but not their data table, p1070-1071), Rogers et al. (1993) report most of their new dates using standard error (SE) rather than standard deviation 1σ (although 1σ is necessarily cited on single crystal analyses of sanidine and anorthoclase for TM-4). These measures are directly related, but not directly comparable. In their recalibration table of various Western Interior radiometric dates, Roberts et al. (2013; table 6.1) state directly the ages and error from the Rogers et al. (1993) body text, thus mixing 1σ values alongside SE without indicating which is which, or noting the incomparability of these values.

Foreman et al. (2008): Foreman et al. (2008) present this and other radiometric dates from Rogers et al. (1993) alongside their own new analyses which use a different standard (28.02 Ma; Renne et al., 1998), but do not note this difference.

**Recalibration (Fowler, this article)**

Legacy dates; FCT at 27.84 (Samson & Alexander, 1987; Renne et al., 1998); legacy λT at 5.543 +/- 0.010 E-10/y (Steiger & Jaeger, 1977).

74.066 +/- 0.722 Ma (1σ); (Ar / Ar,sanidine, 1 crystal: no. 3576-03; Rogers et al., 1993)

74.270 +/-0.157 Ma (1σ); or +/- 0.079 Ma (1 SE); (Ar / Ar, plagioclase, 4 x 1 crystal; Rogers et al., 1993)

70.292 +/- 6.871 Ma (1σ); or +/- 1.139 Ma (1 SE); (Ar / Ar, plagioclase, 1 crystal: no. 3576-04; Rogers et al., 1993)

1st recalibration; FCT at 28.201 +/- 0.023 Ma (1σ; Kuiper et al, 2008), and λT at 5.463 E-10/y +/- 1.07 E-11/y (1σ; Min et al., 2000)

75.028 +/- 0.731 Ma (1σ); (sanidine 3576-03)

75.235 +/- 0.159 Ma (1σ); (plagioclase)

71.205 +/- 6.960 Ma (1σ); (plagioclase 3576-04)

2nd recalibration (for reference); FCT at 28.294 +/- 0.294 Ma (1σ), and λT at 5.531 E-10/y +/- 1.35 E-12/y (1σ; both Renne et al. 2011).

75.260 +/- 0.733 Ma (1σ); (sanidine 3576-03)

75.468 +/- 0.159 Ma (1σ); (plagioclase)

71.427+/- 6.980 Ma (1σ); (plagioclase 3576-04)

----

### 76.02 ± 0.08

**Horner & Currie 1994**

Within the upper ~100m of the Two Medicine Formation, Alberta

75.05Ma +/- 0.08 Ma (unknown error type); (Eberth & Deino, pers. comm. 1992, to Horner & Currie, 1994)

76.025 +/- 0.081 Ma (unknown error type); (recalibration, this article)

Horner & Currie (1994) cite Eberth & Deino (pers. comm. 1992) as having recovered this date from a bentonite located "within a few metres" of the Devil's Coulee sites described in Horner & Currie's descriptions of *Hypacrosaurus stebingeri*. This is lithofacies 5.

Horner & Currie state that Devil's Coulee contains Canadian exposures of the Two Medicine Formation. However, Longrich (2009) states that the exposures in Devil's Coulee are of the Oldman Formation, albeit a part that is coeval with Dinosaur Park Formation exposed further east.

**Standard**

The standard and decay constant pairing used is not specified by Horner & Currie (1994), as the date is a pers. comm. from Eberth & Deino (1992). Here I have used the standard / decay constant pairing that Deino used in Goodwin & Deino (1989). These should have been the standards available at the time (1992).

**Recalibration**

Legacy dates; FCT at 27.84 (Samson & Alexander, 1987; Renne et al., 1998); legacy λT at 5.543 +/- 0.010 E-10/y (Steiger & Jaeger, 1977).

75.05 +/- 0.08 Ma (unknown error type); (Eberth & Deino, pers. comm. 1992, to Horner & Currie, 1994)

1st recalibration; FCT at 28.201 +/- 0.023 Ma (1σ; Kuiper et al, 2008), and λT at 5.463 E-10/y +/- 1.07 E-11/y (1σ; Min et al., 2000)

76.025 +/- 0.081 Ma (unknown error type); (recalibration, this article)

2nd recalibration (for reference); FCT at 28.294 +/- 0.294 Ma (1σ), and λT at 5.531 E-10/y +/- 1.35 E-12/y (1σ; both Renne et al. 2011).

76.260 +/- 0.081 Ma (unknown error type); (recalibration, this article)

----

### lithofacies 4

**Lithofacies 4 (Horner et al., 2001)**

Horner et al. (2001) state that lithofacies 4 is equivalent to the "anomalous lacustrine carbonate interval" of Rogers (1998).

Rogers (1998) states that this interval occurs ~240 m above bentonite WOFS/U5 (80.64 Ma), and ~130 m below bentonite TM-4 (75.03 Ma; dates recalibrated from Rogers et al., 1993, by Fowler, this article).

----

### ( 76.39 ± 0.32 )

**Varricchio et al. (2010); recalibration, Fowler (this article)**

TM-003 bentonite

~10.7 m below Upper Discontinuity; 4.3 m above bonebed TM-003 (Varricchio et al., 2010)

75.92 +/- 0.32 (1σ); (Ar / Ar, plagioclase, 4 samples; plateau age; Varricchio et al., 2010)

75.60 +/- 0.40 (1σ); (Ar / Ar, plagioclase, 4 samples; isochron age; Varricchio et al., 2010)

**76.39 +/- 0.32** Ma (1σ); (recalibration; Fowler, this article, see below)

Varricchio et al. (2010) state that the TM-003 bentonite (named here for convenience) occurs approximately 10.7 m below the Upper Discontinuity of Rogers (1994, 1998). The TM-003 locality is a few km west of Choteau, Montana.

Shelton (2007) mentions a radiometric date for a bentonite hoizon considered to be the same horizon as the TM-003 bentonite dated by Varricchio et al. (2010). Shelton states that the bentonite was dated twice using U / Pb SHRIMP method on biotites; however, it seems likely that Shelton (2007) is mistaken here as these bentonites are more typically dated using the Ar / Ar method on biotite, plagioclase, or (preferably) sanidine; by comparison, the U-Pb method requires zircons. Shelton reports retrieving ages of 76.0 +/- 0.6 Ma, and 77.0 +/- 0.7 Ma (not recalibrated) producing an average age of 76.5 Ma. These dates are comparable to the 75.6 and 75.9 Ma dates published by Varricchio et al. (2010), who commented that the dates given by Shelton (2007) were preliminary. It is also worth noting that Varricchio et al. (2010) provide dates based on plagioclase (shown here) alhtough they also performed an analysis on biotites, but got very variable results. Also, note that Shelton (2007) gives a date for an additional bentonite (here called the Justin's Peak bentonite) that is ~11.3 m below the TM-003 bentonite. See individual entry for further details.

**Problem**

The date is given in parentheses as it is inconsistent with the position of the Upper Discontinuity (10.7 m above the TM-003 bentonite) being correlative with the top of the Comrey Sandstone (unit 2) of the Oldman Fm, Alberta. Since a radiometric date at the top of the conformably overlying unit 3 of the Oldman is dated at 77.03 Ma (recalibrated; see individual entry) then the 76.39 +/- 0.32 Ma date retrieved by Varricchio et al. (2010) is therefore too young. This may be a result of methodological differences, or that the Oldman Fm dates use the more reliable sanidine as the study mineral. The TM-003 horizon should be reanalysed using sanidine, if possible.

**Standard**

Varricchio et al. (2010) state that they use the decay constants of Steiger & Jaeger (1977), and an FCT-3 (FCs-3) at 28.03 Ma, equivalent to 523.1 Ma for MMhb-1, with additional analytical data found in Miggins (2010; a USGS open-file report). However, we have not been able to locate the Miggins USGS open file report on the USGS website, and suspect that it was never actually published.

Note that 28.03 Ma is a slightly unusual date for the FCs standard, especially when associated with an MMhb-1 of 523.1 Ma. Renne et al (1998) intercalibrated an MMhb of 523.1 Ma with an FCs of 28.02 Ma; i.e. 0.01 Ma lower than the 28.03 Ma given by Varricchio et al. (2010). I expect that the 28.03 Ma used by Varricchio et al. (2010) is due to the analysis being performed at the USGS in Denver, CO, which historically used 28.03 Ma as the FCs age, based on the work of Obradovich (the reason why many other FCs ages in this chart are given as 28.03: these are all Obradovich analyses; this is rarely stated explicitly, but see Hicks et al., 2002). However, what is unique about the Varricchio et al. (2010) ages is the association of MMhb-1 at 523.1 Ma with FCs at 28.03 Ma; previous Denver USGS analyses have associated a 28.03 Ma age for the FCs with an MMhb-1 of 520.4 Ma (as stated in Hicks et al., 2002). I speculate that the newer MMhb-1 date was cited by Varricchio et al. (2010) without being intercalibrated in an independent analysis. Consultation of Miggins (2010) might confirm this, if it was ever published.

**Recalibration, Ar / Ar (Fowler, this article)**

Legacy date; FCT at 28.03 (see above); legacy λT at 5.543 +/- 0.010 E-10/y (Steiger & Jaeger, 1977)).

75.92 +/- 0.32 (1σ); (Ar / Ar, plagioclase, 4 samples; plateau age; Varricchio et al., 2010)

75.60 +/- 0.40 (1σ); (Ar / Ar, plagioclase, 4 samples; isochron age; Varricchio et al., 2010)

1st recalibration; FCT at 28.201 +/- 0.023 Ma (1σ; Kuiper et al, 2008); λT at 5.463 E-10/y +/- 1.07 E-11/y; 1σ (Min et al., 2000)

76.392 +/- 0.322 Ma (1σ); (plateau age; recalibration, this article)

76.070 +/- 0.403 Ma (1σ); (isochron age; recalibration, this article)

2nd recalibration (for reference); FCT at 28.294 +/- 0.294 Ma (1σ), and λT at 5.531 E-10/y +/- 1.35 E-12/y (1σ; both Renne et al. 2011).

76.628 +/- 0.323 Ma (1σ); (plateau age; recalibration, this article)

76.305 +/- 0.1404 Ma (1σ); (isochron age; recalibration, this article)

----

### 77.2

**Horner et al, 2001 (pers. comm. Rogers & Swisher):**

76.7 Ma (?Ar / Ar, Horner et al., 2001; pers. comm. from Rogers & Swisher)

77.204 Ma (recalibration, this article)

This date is taken from Horner et al. (2001) where the authors attribute the date to Rogers & Swisher (pers. comm.). The date is shown occurring within lithofacies 4, i.e. above the Upper Discontinuity of Rogers (1998). Error is not given, nor is the subject mineral on which the date is based.

**Standard**:

Although the standard and decay constant are not stated, Swisher (who performed the analysis) used an FCT standard of 28.02 in both preceding and succeeding analyses (Swisher et al., 1999; 2002), suggesting that this is probably also the standard used circa 2001. The decay constant used is unknown, but is unlikely to be Min et al (2000) as this was not wdiely used until much later. Hence I have used the then-standard decay constant of Steiger & Jaeger (1977).

**Recalibration**

Legacy date; FCT at 28.02 (Renne et al., 1998); legacy λT at 5.543 +/- 0.010 E-10/y (Steiger & Jaeger, 1977)).

76.7 Ma (?Ar / Ar, Horner et al., 2001; pers. comm. from Rogers & Swisher)

1st recalibration; FCT at 28.201 +/- 0.023 Ma (1σ; Kuiper et al, 2008); λT at 5.463 E-10/y +/- 1.07 E-11/y; 1σ (Min et al., 2000)

77.204 Ma (recalibration, this article)

2nd recalibration (for reference); FCT at 28.294 +/- 0.294 Ma (1σ), and λT at 5.531 E-10/y +/- 1.35 E-12/y (1σ; both Renne et al. 2011).

77.442 Ma (recalibration, this article)

----

### Upper Discontinuity

**Upper Discontinuity**

The Upper Discontinuity separates lithofacies 3 and 4, and demonstrates a shift towards more lacustrine sediments. The discontinuity itself is hypothesized to represent an abrupt increase in accommodation related to the transgression of the Bearpaw seaway (Rogers, 1994; 1998). However, it is not clear whether there is a correlative horizon within the more intensively studied Oldman or Dinosaur Park Formations (Alberta), or the Judith River Fm, Montana.

**Age of the upper discontinuity**

Age and correlation of the Upper Discontinuity are inconclusive, with often conflicting data from radiometric dates.

Rogers (1994) places the Upper Discontinuity at ~77 - 78 Ma, and considers that it represents the nonmarine expression of the onset of transgression of the Bearpaw Seaway. This could be correlative with the top of the Comrey Sandstone (unit 2) of the Oldman Fm, Alberta (and the top of the 'lower' Judith River Fm, Montana), or the Oldman - Dinosaur Park Fm contact, or even the sandy zone in the lower half of the Dinosaur Park Fm.

Ryan (2003, PhD) states that Rogers & Eberth (pers. comms.) consider the upper discontinuity to be equivalent to the Dinosaur Park Fm-Oldman disconformity in Canada, which is dated at ~77Ma (see individual entry). This view is closer to what is suggested by radiometric dates from the Judith River Fm (Rogers and Swisher, 1996) and a recent date from the Two Medicine Formation (Varricchio et al., 2010).

Rogers & Kidwell (2000; p. 134) state that the upper discontinuity occurs "only a few meters [?] beneath a bentonite bed [Ar / Ar] dated at 75.4 Ma [not recalibrated] in the Judith River Formation type area (Rogers and Swisher, 1996)". This 75.4 Ma date was only ever presented in the Rogers & Swisher (1996) GSA abstract; as such details of the analysis are not available. However I have recalibrated it to 76.4 Ma, based on the standard and decay constant pairing used by Rogers et al. (1993; see individual entry for details). Varricchio et al. (2010) recovered an age of 76.39 +/- 0.32 Ma (recalibrated; see individual entry) for the TM-003 bentonite which occurs in the Two Medicine Fm, approximately 10.7 m below the Upper Discontinuity.

Rogers et al. (2016) offer a date of 76.24 Ma for a bentonite bed in the type area for the Judith River Fm, ~5m below a "Mid Judith Discontinuity", which is shown one of their figures as questionably equivalent to the upper discontinuity below the lacustrine lithofacies 4 of the Two Medicine Formation. If these radiometric dates are accurate then it does not seem likely that the Mid Judith Discontinuity is equivalent to the Upper Discontinuity of the Two Medicine Fm. This issue remains unresolved.

It is important to note that the recalibrated radiometric dates of Rogers & Swisher (1996) and Varricchio et al. (2010) would place the discontinuity as stratigraphically equivalent to the lower part of the Dinosaur Park Formation rather than the top of the Comrey Sandstone in the underlying Oldman Fm. Furthermore the "Plateau Tuff" occurs 36 m above the Oldman - Dinosaur Park Fm contact and is dated at 76.39 Ma, nearly identical to the recalibrated date for the bentonite dated by Rogers & Swisher (1996).

Here I have positioned the Upper Discontinuity to be equivalent with the Oldman - Dinosaur Park Formation boundary.

----

### lithofacies 3

**Lithofacies 3, (Horner et al., 2001)**

Horner et al. (2001) describe lithofacies 3 as having been deposited during the progradation phase of the alluvial facies of the Two Medicine Fm (regression R8 of Rogers, 1998). It contains a number of radiometrically dated horizons which aid in its stratigraphic placement (see individual entries).

Bonebed TM-003 occurs ~15 m below the lacustrine facies of lithofacies 4 (Varricchio et al., 2010), at the uppermost part of Lithofacies 3,

Varricchio et al. (2010) suggest that the TM-003 bonebed occurs within the lacustrine facies, however, they state that "the top of the bonebed lies ~2 m below a sequence of laminated silty mudstones and 6.8 m below a charophytiferous limestone". Varricchio et al. (2010) also note that Lorenz & Gavin ilustrate the TM-003 bonebed horizon (then unnamed) as approximately one third of the way up through their 27 m thick "lake subfacies".

**Detrital zircons**

Varricchio et al. (2010) performed U-Pb radiometric dating on zircons extracted from the TM-003 bonebed. The youngest recovered zircon date was 78.4 +/- 0.13 Ma.

----

### 77.4 ± 0.5

**Shelton (2007); recalibration, Fowler (this article)**

Justin's Peak bentonite

~23 m below Upper Discontinuity; ~11.3 m below bonebed TM-003 (my inference, from Shelton, 2007)

76.9 +/- 0.5 Ma (unspecified error type); (?Ar / Ar, biotite, Shelton, 2007; Miggins pers. comm. to Shelton)

**77.4 +/- 0.5** Ma (unspecified error type); (recalibration; Fowler, this article, see below)

Shelton (2007) states that the Justin's Peak bentonite (named here for convenience) occurs approximately 3.52 m above the base of her measured section at the Justin's Peak locality. Based on the measured sections in appendix 1 of Shelton (2007), this places the Justin's Peak bentonite (and equivalents) ~11.3 m below the TM-003 bentonite, and therefore ~23 m below the Upper Discontinuity of Rogers (1994, 1998). The Justin's Peak locality is a few km W of Choteau, Montana.

**Standard**

Shelton (2007) reports three radiometric dates based on bentonites, and states that they were analysed using U / Pb SHRIMP method on biotites; however, it seems likely that Shelton (2007) is mistaken here as these bentonites are more typically dated using the Ar / Ar method on biotite, plagioclase, or (preferably) sanidine; by comparison, the U-Pb method requires zircons. Shelton's dates were reported as pers. comm. from Miggins (USGS).

Varricchio et al. (2010) later performed Ar / Ar analysis on one of the same bentonites, noting that Shelton's analysis had used preliminary dates. The analyses in Varricchio et al. (2010) were performed by Miggins, who is also a coauthor.

Hence, I suspect that the dates of Shelton (2007) were acquired through Ar / Ar analysis. No details of the standard or decay constant are given by Shelton, and since this is 2007, there are a variety of standards that might have been used. I have chosen to recalibrate the dates using the standards that Miggins used for the Varricchio et al. (2010) paper. Varricchio et al. (2010) state that they use the decay constants of Steiger & Jaeger (1977), and an FCT-3 (FCs-3) at 28.03 Ma, equivalent to 523.1 Ma for MMhb-1, with additional analytical data found in Miggins (2010; a USGS open-file report). However, we have not been able to locate the Miggins USGS open file report on the USGS website, and suspect that it was never actually published.

Note that 28.03 Ma is a slightly unusual date for the FCs standard, especially when associated with an MMhb-1 of 523.1 Ma. Renne et al (1998) intercalibrated an MMhb of 523.1 Ma with an FCs of 28.02 Ma; i.e. 0.01 Ma lower than the 28.03 Ma given by Varricchio et al. (2010). I expect that the 28.03 Ma used by Varricchio et al. (2010) is due to the analysis being performed at the USGS in Denver, CO, which historically used 28.03 Ma as the FCs age, based on the work of Obradovich (the reason why many other FCs ages in this chart are given as 28.03: these are all Obradovich analyses; this is rarely stated explicitly, but see Hicks et al., 2002). However, what is unique about the Varricchio et al. (2010) ages is the association of MMhb-1 at 523.1 Ma with FCs at 28.03 Ma; previous Denver USGS analyses have associated a 28.03 Ma age for the FCs with an MMhb-1 of 520.4 Ma (as stated in Hicks et al., 2002). I speculate that the newer MMhb-1 date was cited by Varricchio et al. (2010) without being intercalibrated in an independent analysis. Consultation of Miggins (2010) might confirm this, if it was ever published.

**Recalibration, Ar / Ar (Fowler, this article)**

Legacy date; FCT at 28.03 (see above); legacy λT at 5.543 +/- 0.010 E-10/y (Steiger & Jaeger, 1977)).

76.9 +/- 0.5 (unspecified error type); (?Ar / Ar, biotite n=?, Shelton, 2007; Miggins pers. comm. to Shelton)

1st recalibration; FCT at 28.201 +/- 0.023 Ma (1σ; Kuiper et al, 2008); λT at 5.463 E-10/y +/- 1.07 E-11/y; 1σ (Min et al., 2000)

77.378 +/- 0.503 Ma (unspecified error type); (recalibration, this article)

2nd recalibration (for reference); FCT at 28.294 +/- 0.294 Ma (1σ), and λT at 5.531 E-10/y +/- 1.35 E-12/y (1σ; both Renne et al. 2011).

77.617 +/- 0.504 Ma (unspecified error type); (recalibration, this article)

----

### 78.03 ± 0.19

**Foreman et al (2008)**

HH -"Hadro Hill bentonite"; (Foreman et al., 2008)

~265 m above base 2Med Fm; ~65 m below Upper Discontinuity; (Foreman et al., 2008)

77.52 +/-0.19 (1σ); (Ar / Ar, sanidine, 4 samples; Foreman et al., 2008)

75.8 +/- 0.7 (1σ); (Ar / Ar, plagioclase; Foreman et al., 2008)

78.03 +/- 0.? (recalibration; Roberts et al., 2013)

78.03 +/- 0.19 Ma (1σ); (recalibration; Fowler, this article, see below)

Foreman et al. (2008) state that the HH bentonite occurs approximately 65m below the upper discontinuity of Rogers (1994, 1998), ~ 265 m above the base of the formation. The HH locality is a few km SW of Cut Bank, Montana (close to the type section of the Two Medicine Fm), within ~2 km of the Shields Crossing locality (WOFS/U5) radiometrically dated by Rogers et al. (1993).

Note that the sanidine and plagioclase dates given by Foreman et al., (2008) are 1.7 m.y. different for the same horizon; most of the other dates for the Two Medicine Fm are plagioclase or biotite dates, rather than the preferred sanidine.

**Standard**

Fish Canyon Tuff sanidine from Colorado was used as a mineral standard, with a reference age of 28.02 Ma (Renne et al., 1998). Decay constant used is not stated, so I have recalibrated the date twice using both Steiger & Jaeger (1977; 5.543E-10), and Min et al. (2000; 5.463E-10).

**Recalibration, Ar / Ar (Fowler, this article)**

Legacy date; FCT at 27.84 (Samson & Alexander, 1987; Renne et al., 1998); legacy λT unspecified (see above).

77.52 +/-0.19 (1σ); (Ar / Ar, sanidine, 4 samples; Foreman et al., 2008)

75.8 +/- 0.7 (1σ); (Ar / Ar, plagioclase; Foreman et al., 2008)

1st recalibration; FCT at 28.201 +/- 0.023 Ma (1σ; Kuiper et al, 2008); λT at 5.463 E-10/y +/- 1.07 E-11/y; 1σ (Min et al., 2000)

78.014 +/- 0.191 Ma (1σ); (sanidine, legacy λT at 5.463 E-10/y +/- 1.07 E-11/y; 1σ; Min et al., 2000)

78.029 +/- 0.191 Ma (1σ); (sanidine, legacy λT at 5.543 +/- 0.010 E-10/y; Steiger & Jaeger, 1977).

76.283 +/- 0.704 Ma (1σ); (plagioclase, legacy λT at 5.463 E-10/y +/- 1.07 E-11/y; 1σ; Min et al., 2000)

76.298 +/- 0.705 Ma (1σ); (plagioclase, legacy λT at 5.543 +/- 0.010 E-10/y; Steiger & Jaeger, 1977).

2nd recalibration (for reference); FCT at 28.294 +/- 0.294 Ma (1σ), and λT at 5.531 E-10/y +/- 1.35 E-12/y (1σ; both Renne et al. 2011).

78.255 +/- 0.192 Ma (1σ); (sanidine, legacy λT at 5.463 E-10/y +/- 1.07 E-11/y; 1σ; Min et al., 2000)

78.270 +/- 0.192 Ma (1σ); (sanidine, legacy λT at 5.543 +/- 0.010 E-10/y; Steiger & Jaeger, 1977).

76.519 +/- 0.706 Ma (1σ); (plagioclase, legacy λT at 5.463 E-10/y +/- 1.07 E-11/y; 1σ; Min et al., 2000)

76.534 +/- 0.707 Ma (1σ); (plagioclase, legacy λT at 5.543 +/- 0.010 E-10/y; Steiger & Jaeger, 1977).

----

### 80.64 ± 0.22

**Rogers et al. (1993); recalibration, Fowler (this article)**

WOFS/U5; "Shield's Crossing bentonite": Foreman et al (2008)

~108m above base 2Med Fm (Rogers et al, 1993)

~100m above base 2Med Fm (Foreman et al., 2008)

~98m above base 2Med Fm (Rogers, 1998)

79.603 +/- 0.214 Ma (1σ); +/- 0.096 Ma (1 SE); (Ar / Ar, plagioclase, 5 x 1 crystals; Rogers et al., 1993)

**80.637 +/- 0.217** Ma (1σ); (recalibration; Fowler, this article, see below)

Rogers (1998) shows the WOFS/U5 bentonite occurring ~98 m above the base of the Two Medicine Fm, ~25m above the "Fluvial Disconformity", referred to elsewhere as the "Lower Discontinuity" (Rogers, 1994; Shelton, 2007; Foreman et al., 2008). The WOFS/U5 bentonite is part of a series of bentonites in the lower part of lithofacies 3 which are thought to be correlative with the Ardmore bentonites dated elsewhere in the Western Interior (Rogers, 1994).

**Standard**

Rogers et al. (1993) use the FCT at 27.84 Ma (Cebula et al., 1986) "intercalibrated in-house" with MMhb-I at 520.4 Ma (Samson & Alexander, 1987). Decay constant (λT) follows Steiger & Jaeger (1977), at 5.543 +/- 0.010 E-10/y.

**Variation in reporting (Rogers et al., 1993) & consequent citation mistakes (Roberts et al., 2013)**

Within the text body (p. 1071-1072; but not their data table, p1070-1071), Rogers et al. (1993) report most of their new dates using standard error (SE) rather than standard deviation 1σ (although 1σ is necessarily cited on single crystal analyses of sanidine and anorthoclase for TM-4). These measures are directly related, but not directly comparable. In their recalibration table of various Western Interior radiometric dates, Roberts et al. (2013; table 6.1) state directly the ages and error from the Rogers et al. (1993) body text, thus mixing 1σ values alongside SE without indicating which is which, or noting the incomparability of these values.

**Foreman et al. (2008)**

Foreman et al. (2008) present this and other radiometric dates from Rogers et al. (1993) alongside their own new analyses which use a different standard (28.02 Ma; Renne et al., 1998), but do not note this difference.

**Recalibration, Ar / Ar (Fowler, this article)**

Legacy dates; FCT at 27.84 (Samson & Alexander, 1987; Renne et al., 1998); legacy λT at 5.543 +/- 0.010 E-10/y (Steiger & Jaeger, 1977).

79.603 +/- 0.214 Ma (1σ); +/- 0.096 Ma (1 SE); (Ar / Ar, plagioclase, 5 x 1 crystals; Rogers et al., 1993)

72.025 +/- 5.300 Ma (1σ); (Ar / Ar, plagioclase, 1 crystal: no. 3574-05; Rogers et al., 1993)

79.862 +/- 0.302 Ma (1σ); (Ar / Ar, biotite, 1 crystal: no. 3570-05; Rogers et al., 1993)

78.266 +/- 0.146 Ma (1σ); +/- 0.073 Ma (1 SE); (Ar / Ar, biotite, 4 x 1 crystals; Rogers et al., 1993)

76.426 +/- 0.447 Ma (1σ); (Ar / Ar, biotite, 1 crystal: no. 3570-01; Rogers et al., 1993)

1st recalibration; FCT at 28.201 +/- 0.023 Ma (1σ; Kuiper et al, 2008), and λT at 5.463 E-10/y +/- 1.07 E-11/y (1σ; Min et al., 2000)

80.637 +/- 0.217 Ma (1σ); (plagioclase, 5 x 1 crystals)

72.960 +/- 5.369 Ma (1σ); (plagioclase, 1 crystal: no. 3574-05)

80.899 +/- 0.306 Ma (1σ); (biotite, 1 crystal: no. 3570-05)

79.283 +/- 0.148 Ma (1σ); (biotite, 4 x 1 crystals)

77.419 +/- 0.453 Ma (1σ); (biotite, 1 crystal: no. 3570-01)

2nd recalibration (for reference); FCT at 28.294 +/- 0.294 Ma (1σ), and λT at 5.531 E-10/y +/- 1.35 E-12/y (1σ; both Renne et al. 2011).

80.885 +/- 0.217 Ma (1σ); (plagioclase, 5 x 1 crystals)

73.187 +/- 5.384 Ma (1σ); (plagioclase, 1 crystal: no. 3574-05)

81.148 +/- 0.307 Ma (1σ); (biotite, 1 crystal: no. 3570-05)

79.527 +/- 0.148 Ma (1σ); (biotite, 4 x 1 crystals)

77.658 +/- 0.454 Ma (1σ); (biotite, 1 crystal: no. 3570-01)

----

### 80.81 ± 0.28

**Rogers et al. (1993); recalibration, Fowler (this article)**

TM-3

"Seven Mile Hill bentonite" : Foreman et al (2008)

~105m above base 2Med Fm

79.715 +/- 0.075 Ma (1σ); +/- 0.031 (1 SE); (Ar / Ar, biotite 6 x 1 crystals; Rogers et al., 1993)

80.751 +/- 0.076 Ma (1σ); (recalibration, biotite 6 x 1 crystals; Fowler, this article)

79.771 +/-0.275 Ma (1σ); +/- 0.112 (1 SE); (Ar / Ar, plagioclase, 6 x 1 crystals; Rogers et al., 1993)

**80.807 +/-0.279** Ma (1σ); (recalibration, plagioclase, 6 x 1 crystals; Fowler, this article)

Neither the plagioclase nor biotite date was preferred by Rogers et al. (1993) as they are concordant. I arbitrarily chose to plot the 80.807 date (80.81, 2 d.p.).

The TM-3 bentonite overlies the crystal tuff RT/TM-7 horizon, and both have yielded radiometric dates (Rogers et al., 1993; Rogers, 1998; Roberts, 1999; see individual entries). RT/TM-7 and TM-3 immediately overlie the Fluvial / Lower Discontinuity (Rogers, 1994; 1998; Shelton, 2007) that caps lithofacies 2 (Horner et al., 2001; lithofacies "b" of Lorenz and Gavin, 1984).The TM-3 bentonite is part of a series of bentonites in the lower part of lithofacies 3 which are thought to be correlative with the Ardmore bentonites dated elsewhere in the Western Interior (Rogers, 1994).

Standard

Rogers et al. (1993) use the FCT at 27.84 Ma (Cebula et al., 1986) "intercalibrated in-house" with MMhb-I at 520.4 Ma (Samson & Alexander, 1987). Decay constant (λT) follows Steiger & Jaeger (1977), at 5.543 +/- 0.010 E-10/y.

Variation in reporting (Rogers et al., 1993) & consequent citation mistakes (Roberts et al., 2013)

Within the text body (p. 1071-1072; but not their data table, p1070-1071), Rogers et al. (1993) report most of their new dates using standard error (SE) rather than standard deviation 1σ (although 1σ is necessarily cited on single crystal analyses of sanidine and anorthoclase for TM-4). These measures are directly related, but not directly comparable. In their recalibration table of various Western Interior radiometric dates, Roberts et al. (2013; table 6.1) state directly the ages and error from the Rogers et al. (1993) body text, thus mixing 1σ values alongside SE without indicating which is which, or noting the incomparability of these values.

Foreman et al. (2008)

Foreman et al. (2008) present this and other radiometric dates from Rogers et al. (1993) alongside their own new analyses which use a different standard (28.02 Ma; Renne et al., 1998), but do not note this difference.

Recalibration, Ar / Ar (Fowler, this article)

Legacy dates; FCT at 27.84 (Samson & Alexander, 1987; Renne et al., 1998); legacy λT at 5.543 +/- 0.010 E-10/y (Steiger & Jaeger, 1977).

79.715 +/- 0.075 Ma (1σ); +/- 0.031 (1 SE); (Ar / Ar, biotite 6 x 1 crystals; Rogers et al., 1993)

79.771 +/-0.275 Ma (1σ); +/- 0.112 (1 SE); (Ar / Ar, plagioclase, 6 x 1 crystals; Rogers et al., 1993)

1st recalibration; FCT at 28.201 +/- 0.023 Ma (1σ; Kuiper et al, 2008), and λT at 5.463 E-10/y +/- 1.07 E-11/y (1σ; Min et al., 2000)

80.751 +/- 0.076 Ma (1σ); (biotite 6 x 1 crystals)

80.807 +/-0.279 Ma (1σ); (plagioclase, 6 x 1 crystals)

2nd recalibration (for reference); FCT at 28.294 +/- 0.294 Ma (1σ), and λT at 5.531 E-10/y +/- 1.35 E-12/y (1σ; both Renne et al. 2011).

80.999 +/- 0.076 Ma (1σ); (biotite 6 x 1 crystals)

81.056 +/-0.279 Ma (1σ); (plagioclase, 6 x 1 crystals)

----

### 81.08 ± 0.19

**Rogers et al 1993**

RT/TM-7

~105m above base 2Med Fm, Choteau area

80.044+/- 0.190 Ma (1σ) +/- 0.078 (1 SE); (Ar / Ar, biotite, 6 x 1 crystals; Rogers et al., 1993)

81.084 +/-0.192 Ma (1σ); (recalibration, biotite, 6 x 1 crystals; Fowler, this article))

80.002 +/-0.280 Ma (1σ); +/- 0.114 (1 SE); (Ar / Ar, plagioclase, 6 x 1 crystals; Rogers et al., 1993)

**81.041 +/- 0.284** Ma (1σ); (recalibration, plagioclase, 6 x 1 crystals; Fowler, this article)

Neither the plagioclase nor biotite date was preferred by Rogers et al. (1993) as they are concordant. I arbitrarily chose to plot the 81.084 date (81.08, 2 d.p.).

The RT/TM-7 horizon is a crystal tuff that is overlain by a bentonite (TM-3), both of which have yielded radiometric dates (Rogers et al., 1993; Rogers, 1998; Roberts, 1999; see individual entries). RT/TM-7 immediately overlies the Fluvial / Lower Discontinuity (Rogers, 1994; 1998; Shelton, 2007) that caps lithofacies 2 (Horner et al., 2001; lithofacies "b" of Lorenz and Gavin, 1984).

**Standard**

Rogers et al. (1993) use the FCT at 27.84 Ma (Cebula et al., 1986) "intercalibrated in-house" with MMhb-I at 520.4 Ma (Samson & Alexander, 1987). Decay constant (λT) follows Steiger & Jaeger (1977), at 5.543 +/- 0.010 E-10/y.

**Variation in reporting (Rogers et al., 1993) & consequent citation mistakes (Roberts et al., 2013)**

Within the text body (p. 1071-1072; but not their data table, p1070-1071), Rogers et al. (1993) report most of their new dates using standard error (SE) rather than standard deviation 1σ (although 1σ is necessarily cited on single crystal analyses of sanidine and anorthoclase for TM-4). These measures are directly related, but not directly comparable. In their recalibration table of various Western Interior radiometric dates, Roberts et al. (2013; table 6.1) state directly the ages and error from the Rogers et al. (1993) body text, thus mixing 1σ values alongside SE without indicating which is which, or noting the incomparability of these values.

**Foreman et al. (2008)**

Foreman et al. (2008) present this and other radiometric dates from Rogers et al. (1993) alongside their own new analyses which use a different standard (28.02 Ma; Renne et al., 1998), but do not note this difference.

Neither date was preferred by Rogers et al. (1993) as they are concordant. I arbitrarily chose to plot the 80.807 date (80.81, 2 d.p.).

**Recalibration, Ar / Ar (Fowler, this article)**

Legacy dates; FCT at 27.84 (Samson & Alexander, 1987; Renne et al., 1998); legacy λT at 5.543 +/- 0.010 E-10/y (Steiger & Jaeger, 1977).

80.044+/- 0.190 Ma (1σ) +/- 0.078 (1 SE); (Ar / Ar, biotite, 6 x 1 crystals; Rogers et al., 1993)

80.002 +/-0.280 Ma (1σ); +/- 0.114 (1 SE); (Ar / Ar, plagioclase, 6 x 1 crystals; Rogers et al., 1993)

1st recalibration; FCT at 28.201 +/- 0.023 Ma (1σ; Kuiper et al, 2008), and λT at 5.463 E-10/y +/- 1.07 E-11/y (1σ; Min et al., 2000)

81.084 +/-0.192 Ma (1σ); (biotite, 6 x 1 crystals)

81.041 +/- 0.284 Ma (1σ); (plagioclase ,6 x 1 crystals)

2nd recalibration (for reference); FCT at 28.294 +/- 0.294 Ma (1σ), and λT at 5.531 E-10/y +/- 1.35 E-12/y (1σ; both Renne et al. 2011).

81.333 +/-0.193 Ma (1σ); (biotite, 6 x 1 crystals)

81.290 +/- 0.284 Ma (1σ); (plagioclase ,6 x 1 crystals)

----

### Lower Discontinuity

**Lower Discontinuity**

The Lower Discontinuity occurs between lithofacies 2 and 3 at ~81.1 Ma (recalibrated from Rogers et al., 1993), and represents the nonmarine expression of a fall in sea level associated with the maximum regression (R7) of the Colorado Shale (Rogers, 1994; 1998).

The Lower Discontinuity is shown as either roughly contemporaneous, or slightly younger than the Ardmore bentonite (Claggett Shale) by Rogers (1998), and Rogers & Kidwell (2000). The Ardmore bentonite is dated at 81.21 +/- 0.17 Ma (recalibrated from Hicks et al., 1995). Thus, here I have plotted the Lower Discontinuity as representing 80.3 to 80.1 Ma.

----

### lithofacies 1 & 2

**Lithofacies 1 & 2 (Horner et al., 2001)**

Referred to as lithofacies a and b by Lorenz & Gavin (1984), who show these facies are directly overlain by a volcanic crystal tuff in their composite section based on outcrop near Choteau.

**Lithofacies 2, (Horner et al., 2001)**

Mudstone, equivalent to the "Claggett shaley interval" of Lorenz & Gavin (1984), stratigraphically correlative with the Claggett Shale to the east (Horner et al., 2001). Immediately overlain by a crystal tuff in the Choteau area, which was radiometrically dated by Rogers et al. (1993) as 81.08 Ma (recalibration; Fowler, this article).

**Lithofacies 1, (Horner et al., 2001)**

An unfossiliferous sandstone dominated interval representing proximal shoreline deposits lying immediately above the Virgelle Sandstone, and stratigraphically equivalent to the Eagle Fm further east.

----

### VIRGELLE Fm

**Virgelle Sandstone**

The Virgelle Fm is a marine sandstone that records regression of the Western Interior Seaway (R7; Rogers, 1998).

**Upper contact**

The Virgelle is overlain by the Two Medicine Fm. The Virgelle is time transgressive, but the ammonite *Scaphites hippocrepis II* occurs within it (Gill & Cobban, 1973; Sageman et al., 2014), making the Virgelle no older than 82.00 Ma (Ogg & Hinnov, 2012). Radiometric dates retrieved by Rogers et al. (1993; recalibrated here) show the lower disconformity of the Two Medicine Fm occurring at ~ 81.1 Ma, suggesting that the base of the Two Medicine Fm occurs between 81.1 and 82 Ma.

**Lower contact:**

Lower contact with the Telegraph Creek Fm shown by Sageman et al. (2014) as occurring between the *S. hippocrepis I* (82.70 - 82.00 Ma) and *S. hippocrepis II* (82.00 - 81.53 Ma) ammonite zones (Ogg & Hinnov, 2012).

----

### TELEGRAPH CREEK Fm

**Telegraph Creek**

A regressive marine sandstone (Braman, 2001).

**Upper contact**

Upper contact with Virgelle Sandstone shown by Sageman et al. (2014) as occurring between the *S. hippocrepis I* and *S. hippocrepis II* ammonite zones.

**Lower contact**

Basal contact with the Kevin Mbr of the Marias River Shale shown by Sageman et al. (2014) to occur at the boundary between *D. erdmanni* and *D. bassleri* ammonite zones.

*D. bassleri* specimens were recovered from the lower Telegraph Creek Fm in the type area by Cobban (1950), constraining the base of the unit to this ammonite zone.

----

## Central

### JUDITH RIVER Fm

**Judith River Fm, Central, North, and East MT**

The Judith River Fm is a series of terrestrial channel sandstones, overbank fines, and coaly units, exhibiting occasional marine influence (Rogers, 1993; Horner et al., 2001). In northern Montana, the Judith River Fm is more or less continuously exposed over ~300 km along the Milk River and its tributaries, stretching from the Canadian border north of Rudyard, east through Fresno reservoir, Havre, Malta, as far as Tampico. In central Montana the Judith River Fm is exposed in its type area of the Missouri River breaks (Rogers et al., 2016). Correlation between these areas has been difficult, and it is probably best to consider each geographic area separately.

The Judith River Fm in the type area has recently been formally subdivided into a series of members (Rogers et al., 2016), notably a sand-dominated basal McClelland Ferry Mbr and overlying mud-dominated Coal Ridge Mbr, separated by a distinctive "kick" in subsurface SP logs, named the Mid Judith Discontinuity. However, it is not clear how this type section correlates with exposures of the Judith River Fm along the US-Canada border, from which most of the diagnostic vertebrate fossils have been recovered.

In contrast to the type area, exposures of the Judith River Fm close to the US-Canada border are recognized as direct lithostratigraphic equivalents to Canadian the Oldman, and Foremost Fms of southern Alberta, and can be readily identified as such in outcrop (Schott et al., 2009; Freedman Fowler and Horner, 2015; note that lithostratigraphic equivalents of the Oldman Fm are thicker in Montana than in Alberta; Eberth, 2005).

Rogers et al. (2016) state that the distinctive well-log "kick" is detectable in the subsurface near Havre (Montana) and into southern Canada, but there is very little discussion as to which of the well-defined lithostratigraphic boundaries within the Oldman and Dinosaur Park Fms it is equivalent to. Rogers et al. (2016) state that the discontinuity occurs higher in section than the exposures in Kennedy Coulee, near Rudyard. These exposures comprise the Foremost through to Unit 1 of the Oldman Fm; therefore, if the radiometric dates from the type area are reliable, then the "kick" would seem to be equivalent to a horizon within the Dinosaur Park Formation, possibly the top of the sandy zone (see individual entry). In the only statement suggesting correlation, Rogers et al. (2016; p. 126) state that "the Oldman Formation [is an ...] approximate age equivalent to the McClelland Ferry Member [... and that ...] the overlying Dinosaur Park Formation [is an ...] approximate age equivalent to the Coal Ridge Member". However, radiometric dates from immediately below the discontinuity (76.24 and 76.17 Ma; Rogers et al., 2016) are younger than a radiometric date from the middle of the Dinosaur Park Fm (76.39 Ma; see individual entry). This would make the suggested correlation therefore unlikely, however, accuracy of the radiometric dates may be the problem here.

Thus, lack of clarity on the correlative horizon in Canadian units, and lack of a lithostratigraphic definition for the US-Canada border exposures of the Judith River Fm mean that the newly defined members are therefore of limited use for surficial regional correlation and they are not used here outside of the type area. Instead I refer to the lithostratigraphically correlative Foremost, Oldman, and Dinosaur Park Fms, as defined by Eberth (2005), which offer much greater resolution and are well understood.

The Judith River Fm was suggested by Rogers (1994, 1998) to share many bounding surfaces with the more inland equivalent Two Medicine Formation in western Montana, helping to correlate between them. However, radiometric dates published by Rogers et al. (2016) show some of these correlations (particularly the Upper Discontinuity of the Two Medicine Fm) are no longer likely (see individual entry). Correlation of the Judith River Fm with the more landward Two Medicine Fm is therefore in a state of flux.

**Age**

Despite being studied since the mid 19th century, the upper and lower contacts of the Judith River Fm are still not well documented nor understood, although some details are offered for specific sections by Goodwin & Deino (1989), Rogers (1993), and Rogers et al. (2016). Moreover, some important units, such as the Foremost Fm equivalent, are restricted to western exposures close to the Rocky Mountains (Horner et al., 2001). As a result, the base of the Judith River Fm is highly diachronous across Montana. The internal stratigraphy is therefore currently undergoing important and necessary revisions (Eberth & Rogers, pers. comm.).

A number of radiometric dates help constrain the Judith River Fm, and are particularly useful in dating the contact between the Foremost and Oldman Fm equivalents near the town of Rudyard in northern Montana (see individual entries). Radiometric dates from the type area published by Rogers et al. (2016) raise conflicts with previous correlations based on bounding surfaces (Rogers, 1994, 1998).

----

### Missouri Breaks

**Missouri River Breaks**

Although the Missouri River Breaks sections have been known since the 19th century, relatively little has been published regarding their stratigraphy or paleontology. Measured sections of the upper and lower contacts (with the Bearpaw Fm and Parkman Sandstone, respectively) were published by Rogers (1993). Some sections are illustrated in Rogers (1998), but these are relatively low resolution, with few details. More detailed (published) sections are given by Rogers & Brady (2010).

A recent update to Judith River Fm stratigraphy in the type area was published by Rogers et al. (2016), whereupon the authors erect three new members; the McClelland Ferry, Coal Ridge, and Woodhawk Members.

**Age & correlation**

Age of the Missouri Breaks sections is largely determined by Ar / Ar dates reported by Rogers et al. (2016; see individual entries).

Correlation of the Missouri Breaks section with exposures close to the US-Canada border is problematic. Rogers (1993) describes three depositional discontinuities within the upper part of the Missouri Breaks section of the Judith River Fm. Rogers (1998) correlates these with transgressive lag surfaces formed during transgression of the Bearpaw seaway, occurring after the 76.4 Ma date, and therefore correlative with lithofacies 4 and possibly 5 of the Two Medicine Fm. However revised radiometric dates of Rogers (2016) precludes this possibility. Further discussion is given in the main Judith River Fm entry.

Age of the lower contact of the Judith River Fm with the underlying Parkman Sandstone is shown to occur within the R8 regression of Rogers (1998), however more precise dating has not yet been published.

----

### 75.21 ± 0.12

**Rogers et al. (2016)**

Bentonite PPF1-03

5 m above top of Judith RIver Fm in base of Bearpaw Fm

**75.21 +/- 0.12** Ma (1σ); (Ar / Ar, sanidine, 12 crystals; Rogers et al., 2016)

**Stratigraphy**

Bentonite bed PPF1-03 occurs 5 m above the top of the Judith River Fm, at the base of the overlying Bearpaw Fm (Rogers et al., 2016).

**Standards**

Rogers et al. (2016) state that they use FCT at 28.201 +/- 0.023 Ma (1σ; Kuiper et al., 2008) and λT at 5.463 E-10/y +/- 1.07 E-11/y (1σ; Min et al., 2000).

**Recalibration (Fowler, this article)**

Legacy dates; FCT at 28.201 +/- 0.023 Ma (1σ; Kuiper et al., 2008) and λT at 5.463 E-10/y +/- 1.07 E-11/y (1σ; Min et al., 2000)..

75.21 +/- 0.12 Ma (1σ); (Ar / Ar, sanidine, 12 crystals; Rogers et al., 2016)

Recalibration (for reference); FCT at 28.294 +/- 0.294 Ma (1σ), and λT at 5.531 E-10/y +/- 1.35 E-12/y (1σ; both Renne et al., 2011).

75.443 +/- 0.120 Ma (1σ); (recalibration, this article)

----

### 76.17 ± 0.07

**Rogers et al. (2016)**

Bentonite WHB1-11

108 m below top of Judith RIver Fm, ~10 m below Mid Judith discontinuity

**76.17 +/- 0.07** Ma (1σ); (Ar / Ar, sanidine, 30 crystals; Rogers et al., 2016)

**Stratigraphy**

Bentonite bed WHB1-11 occurs 108 m below the top of the Judith River Formation in alluvial sediments of the McClelland Ferry Member, ~10 m below the mid-Judith discontinuity." (Rogers et al., 2016)

**Standards**

Rogers et al. (2016) state that they use FCT at 28.201 +/- 0.023 Ma (1σ; Kuiper et al., 2008) and λT at 5.463 E-10/y +/- 1.07 E-11/y (1σ; Min et al., 2000).

**Recalibration (Fowler, this article)**

Legacy dates; FCT at 28.201 +/- 0.023 Ma (1σ; Kuiper et al., 2008) and λT at 5.463 E-10/y +/- 1.07 E-11/y (1σ; Min et al., 2000)..

76.17 +/- 0.07 Ma (1σ); (Ar / Ar, sanidine, 30 crystals; Rogers et al., 2016)

Recalibration (for reference); FCT at 28.294 +/- 0.294 Ma (1σ), and λT at 5.531 E-10/y +/- 1.35 E-12/y (1σ; both Renne et al., 2011).

76.406 +/- 0.070Ma (1σ); (recalibration, this article)

----

### 76.24 ± 0.18

**Rogers et al. (2016)**

Bentonite STI-03

84.5 m above base of Judith RIver Fm, ~5 m below Mid Judith discontinuity

**76.24 +/- 0.18** Ma (1σ); (Ar / Ar, sanidine, 10 crystals; Rogers et al., 2016)

**Stratigraphy**

Bentonite bed ST1-03 occurs 84.5 m above the base of the Judith River Formation in alluvial sediments of the McClelland Ferry Member, "immediately" below the mid-Judith discontinuity (show ~5 m below the discontinuity; Rogers et al., 2016)

**Standards**

Rogers et al. (2016) state that they use FCT at 28.201 +/- 0.023 Ma (1σ; Kuiper et al., 2008) and λT at 5.463 E-10/y +/- 1.07 E-11/y (1σ; Min et al., 2000).

**Recalibration (Fowler, this article)**

Legacy dates; FCT at 28.201 +/- 0.023 Ma (1σ; Kuiper et al., 2008) and λT at 5.463 E-10/y +/- 1.07 E-11/y (1σ; Min et al., 2000)..

76.24 +/- 0.18 Ma (1σ); (Ar / Ar, sanidine, 10 crystals; Rogers et al., 2016)

Recalibration (for reference); FCT at 28.294 +/- 0.294 Ma (1σ), and λT at 5.531 E-10/y +/- 1.35 E-12/y (1σ; both Renne et al., 2011).

79.476 +/- 0.181 Ma (1σ); (recalibration, this article)

----

### 76.4

Rogers & Swisher (1996)

~halfway through Judith River Fm in type area, MT

75.4 Ma (Ar / Ar, Rogers & Swisher, 1996; no error is stated)

**76.4** Ma (recalibration, this article)

The 75.4 Ma radiometric date is cited by numerous authors (e.g. Rogers and Kidwell, 2000; Horner et al., 2001), however, it has never been formally published, only being presented in an abstract by Rogers & Swisher (1996). As such, few details are available about the horizon from which the samples were taken, nor the methods used during the analysis.

The sample horizon is a bentonite that occurs in the type area for the Judith River Fm (Rogers & Swisher, 1996; Rogers & Kidwell, 2000). However, the stratigraphic position is only given as "a few meters" above the second (or upper) discontinuity, identified by Ray Rogers in various papers and abstracts concerning the Two Medicine - Judith River clastic wedge (e.g. Rogers & Kidwell, 2000). A schematic measured section given in Rogers & Bradley (2010; Fig. 11) shows the upper discontinuity occurring at ~85 m above the base of the ~170 m thick Judith River Fm. Presumably the radiometric date was recovered from a bentonite a few meters above this position.

**Standard**:

The bentonite was analysed using the Ar / Ar method, but the standard and decay constant pairing used is not stated in the abstract, nor in any subsequent article which cites the date. However, the same authors (Rogers et al., 1993) used an FCT of 27.84 Ma (Samson & Alexander, 1987), with the decay constant λ of Steiger & Jaeger (1977). It is possible that Rogers and Swisher (1996) might have used a new revision of the FCT based on Renne et al. (1994) who recover a FCT of 27.95 +/- 0.18 Ma in an intercalibration analysis, equivalent to Mmhb-1 of 522.5 Ma. However, the Renne et al. (1994) revision was not widely used and I am going to assume here that the analysis was conducted using an FCT age of 27.84, and a λ of 5.543E-10.

**Recalibration**

Legacy date; FCT at 27.84 (Samson & Alexander, 1987); legacy λT at 5.543 +/- 0.010 E-10/y (Steiger & Jaeger, 1977).

75.74 Ma (Ar / Ar; Rogers & Swisher, 1996)

1st recalibration; FCT at 28.201 +/- 0.023 Ma (1σ; Kuiper et al., 2008); λT at 5.463 E-10/y +/- 1.07 E-11/y; 1σ (Min et al., 2000)

76.4 Ma (recalibration, this article)

2nd recalibration (for reference); FCT at 28.294 +/- 0.294 Ma (1σ), and λT at 5.531 E-10/y +/- 1.35 E-12/y (1σ; both Renne et al., 2011).

76.6 Ma (recalibration, this article)

----

## North

### Havre

**Judith River Fm, near Havre, Northern MT**

Exposures of the Judith River Fm near Havre (North-central MT) have not been studied.

It is possible that the Havre sections include stratigraphic equivalents of the Foremost, Oldman, and Dinosaur Park fms of Alberta. Ryan (2003) states that Eberth (pers. comm.) suggests that some exposures near Havre may be of the Dinosaur Park Fm, or a temporal equivalent. A similar view has been expressed by J. Horner (pers. comm.). Therefore I have here chosen to depict these units as upper Oldman Fm (Unit 2 & 3), although this is tenuous and the section may include both older and younger units.

----

### Rudyard

**Rudyard beds, Judith River Fm**

The Rudyard beds refer to exposures of the Judith River Fm north of the town of Rudyard, MT. Most exposures occur along the Milk River and its tributaries, with the most well studied outcrop occurring in Kennedy Coulee, ~1-3 km south of the US-Canada border, which preserves ~50-60 m thickness of nonmarine sandstones, overbank fines, and coals (Goodwin & Deino, 1989; Freedman Fowler & Horner, 2015). These are lithostratigraphically correlative with the upper 20 m or so of the Foremost Fm through to the lower Oldman Fm (Unit 1) of Alberta.

**Age**

Stratigraphic control of Kennedy Coulee is best represented by two radiometric dates. A date of 79.52 Ma was recovered 4.8 m below the marker A coal (uppermost Taber coal zone, immediately below the Herronton Sandstone equivalent), and a date of 79.22 Ma was recovered 27 m above the top of Marker A coal approximately halfway through the equivalent to Oldman Fm Unit 1 (Goodwin & Deino, 1989; recalibrated, Fowler, this article).

Fish teeth collected from the Judith River Fm of Kennedy Coulee are referable to the ray *Pseudomyledaphus* sp., which is otherwise only known from the Foremost Fm and Unit 1 of the Oldman Fm, Alberta (Freedman Fowler & Horner, 2015). The overlying Unit 2 and 3 of the Oldman Fm yield a different species of ray, *Myledaphus* *bipartitus* (Freedman Fowler & Horner, 2015). This supports the correlation of the Rudyard beds with the uppermost Foremost Fm and Unit 1 of the Oldman Fm, Alberta.

----

### 79.22 ± 0.2

**Goodwin & Deino (1989); recalibration, Fowler (this article)**

Bentonite 84MG8-3-4

27 m above the top of Marker A coal

78.2 +/- 0.2 Ma (1σ); (Ar / Ar, sanidine, 4 crystals, ; Goodwin & Deino, 1989)

*78.71 +/- 0. Ma (*miscalculated recalibration; Roberts et al., 2013)

**79.216 +/- 0.2** Ma (1σ); (recalibration; this article; see below)

Other (non-preferred) dates recovered for 84MG8-4 include:

78.20 +/- 0.21 Ma (Ar / Ar; biotite, 5 crystals; Goodwin & Deino, 1989)

78.1 +/- 0.6 Ma (K /Ar; biotite; Goodwin & Deino, 1989)

**Stratigraphy**

Ash date from bentonite "84MG8-3-4": located 27m above the top of the "Marker A coal" (bottom of Kennedy Coulee) which is equivalent to the Taber coal (Goodwin, pers. comm.) of the upper Foremost Fm, S. Alberta. It therefore occurs in the lower part of the Judith River Fm, in the equivalent of the "Unit 1" (Eberth, 2005) part of the Oldman Fm in Southern Alberta (also sometimes referred to as "lower muddy"; see individual entry).

**Standards**

Goodwin & Deino state that they use λT = 5.543 E-10/y (Steiger & Jaeger, 1977), and a fluence monitor of MMhb-1 at 520.4 Ma (Samson & Alexander, 1987) which should be equivalent to the FCT at 27.84 Ma (Samson & Alexander, 1987; Renne et al., 1998). This is corroborated by a more explicit description (Rogers et al., 1993) of the methodology of the Berkeley laboratory at this time as "Ages were calculated using a J value calculated from six replicate analyses of individual grains of the coirradiated monitor mineral Fish Canyon Tuff sanidine with a reference age of 27.84 Ma (modified from Cebula et al. 1986) intercalibrated in-house with Minnesota hornblende MMhb-I with a published age of 520.4 Ma (Samson & Alexander, 1987)."

*Erroneous recalibration (Roberts et al., 2013)

Roberts et al. (2013) recalibrate the dates from Goodwin & Deino (1989), however they input an incorrect original (legacy) FCT standard of 28.02 Ma (i.e. from Renne et al., 1998), producing recalibrated dates that are incorrect by nearly half a million years (see below). For the recalculation to be correct, the legacy standard must be the value of FCT that was equivalent to the MMhb-1 at 420.4 Ma, which is FCT = 27.84 Ma (Samson & Alexander, 1987; see Renne et al., 1998).

The recalibrated dates of Roberts et al. (2013) were replicated (therefore confirmed) by rerunning the legacy values through the recalibration spreadsheet provided by the Earth-Time institute.

**Recalibration (Fowler, this article)**

Legacy dates; FCT at 27.84 (Samson & Alexander, 1987); legacy λT at 5.543 +/- 0.010 E-10/y (Steiger & Jaeger, 1977).

78.2 +/- 0.2 Ma (1σ); (Ar / Ar, sanidine, 4 crystals, ; Goodwin & Deino, 1989)

1st recalibration; FCT at 28.201 +/- 0.023 Ma (1σ; Kuiper et al., 2008), and λT at 5.463 E-10/y +/- 1.07 E-11/y (1σ; Min et al., 2000)

79.216 +/- 0.203 Ma (1σ); (recalibration, this article)

2nd recalibration (for reference); FCT at 28.294 +/- 0.294 Ma (1σ), and λT at 5.531 E-10/y +/- 1.35 E-12/y (1σ; both Renne et al., 2011).

79.460 +/- 0.097 Ma (1σ); (recalibration, this article)

----

### 79.52 ± 0.2

**Goodwin & Deino (1989); recalibration, Fowler (this article)**

Bentonite 85MG7-16-1

4.8 m below the top of Marker A coal

78.5 +/- 0.2 Ma (1σ); (Ar / Ar, sanidine, 3 crystals, ; Goodwin & Deino, 1989)

*79.02 +/- 0.2 Ma (*miscalculated recalibration; Roberts et al., 2013)

**79.520 +/- 0.2** Ma (1σ); (recalibration; this article; see below)

Other (non-preferred) dates recovered for 84MG7-16-1 include:

79.46 +/- 0.27 Ma (1σ); (Ar / Ar; biotite, 8 crystals; Goodwin & Deino, 1989)

78.3 +/- 0.7 Ma (1σ); (K / Ar; biotite; Goodwin & Deino, 1989)

77.2 +/- 0.8 Ma (1σ); (K / Ar, biotite; Goodwin & Deino, 1989)

**Stratigraphy**

Ash date from bentonite "85MG7-16-1" found in a thick coal 4.8m below the top of "Marker A coal" (bottom of Kennedy Coulee) which is equivalent to the Taber coal (Goodwin, pers. comm) of the upper Foremost Fm, S. Alberta. Therefore this horizon is effectively part of the Taber coal zone.

Goodwin & Deino (1989) consider the Ar / Ar sanidine date more reliable, as the biotite crystals showed signs of moderate potassium (K) leaching. However, they continue to cite the 79.46 +/- 0.27 biotite - 40Ar/39Ar date throughout the paper, and this should be considered a maximum possible age for the horizon.

This date is important as it effectively places an age constraint on the Montana-JRFm equivalent of the Taber coal zone. This is even more important as after deposition of the Taber coal zone, there is a major marine regression that defines the boundary between the Taber coal zone and Herronton sst / Oldman Fm.

**Standards**

Goodwin & Deino state that they use λT = 5.543 E-10/y (Steiger & Jaeger, 1977), and a fluence monitor of MMhb-1 at 520.4 Ma (Samson & Alexander, 1987) which should be equivalent to the FCT at 27.84 Ma (Samson & Alexander, 1987; Renne et al., 1998). This is corroborated by a more explicit description (Rogers et al., 1993) of the methodology of the Berkeley laboratory at this time as "Ages were calculated using a J value calculated from six replicate analyses of individual grains of the coirradiated monitor mineral Fish Canyon Tuff sanidine with a reference age of 27.84 Ma (modified from Cebula et al. 1986) intercalibrated in-house with Minnesota hornblende MMhb-I with a published age of 520.4 Ma (Samson & Alexander, 1987)."

*Erroneous recalibration (Roberts et al., 2013)

Roberts et al. (2013) recalibrate the dates from Goodwin & Deino (1989), however they input an incorrect original (legacy) FCT standard of 28.02 Ma (i.e. from Renne et al., 1998), producing recalibrated dates that are incorrect by nearly half a million years (see below). For the recalculation to be correct, the legacy standard must be the value of FCT that was equivalent to the MMhb-1 at 420.4 Ma, which is FCT = 27.84 Ma (Samson & Alexander, 1987; see Renne et al., 1998).

The recalibrated dates of Roberts et al. (2013) were replicated (therefore confirmed) by rerunning the legacy values through the recalibration spreadsheet provided by the Earth-Time institute.

**Recalibration (Fowler, this article)**

Legacy dates; FCT at 27.84 Ma (Samson & Alexander, 1987); legacy λT at 5.543 +/- 0.010 E-10/y (Steiger & Jaeger, 1977).

78.2 +/- 0.2 Ma (1σ); (Ar / Ar, sanidine, 4 crystals, ; Goodwin & Deino, 1989)

1st recalibration; FCT at 28.201 +/- 0.023 Ma (1σ; Kuiper et al., 2008), and λT at 5.463 E-10/y +/- 1.07 E-11/y (1σ; Min et al., 2000)

79.520 +/- 0.203 Ma (1σ); (recalibration, this article)

2nd recalibration (for reference); FCT at 28.294 +/- 0.294 Ma (1σ), and λT at 5.531 E-10/y +/- 1.35 E-12/y (1σ; both Renne et al., 2011).

79.765 +/- 0.203 Ma (1σ); (recalibration, this article)

----

### Malta

**Malta beds, Judith River Fm**

The Malta beds are an informal term for exposures of the Judith River Fm near Malta, East-central MT.

**Stratigraphy**

There are very few studies concerning the geology of the Judith River Fm in the area surrounding Malta, MT. Indeed, measured sections are only published in MS theses by Malik (1990) and LaRock (2000).

In various schematic diagrams of the Judith River Wedge published by Ray Rogers (e.g. Rogers, 1998), exposures of the Judith River Fm east of the Missouri River Breaks (longitudinally equivalent to Malta) are shown as stratigraphically below a discontinuity, and therefore probably equivalent to the Oldman Fm (~77 Ma and older), but not the Dinosaur Park Fm (younger than ~77 Ma), in southern Alberta.

Fish teeth collected from Malta Judith River Fm dinosaur quarries are referable to the ray *Myledaphus* *bipartitus*, which, in Alberta, is only found in and above Unit 2 (Comrey Sandstone), with the underlying Foremost Fm and Unit 1 of the lower Oldman Fm exclusively yielding a different taxon: *Pseudomyledaphus* sp. (Freedman Fowler & Horner, 2015). This supports the assignment of the Malta Judith River Fm localities to the Comrey Sandstone Zone of the Oldman Fm (Freedman Fowler & Horner, 2015).

Therefore, here I have chosen to plot the Judith River Fm of the Malta area as equivalent to the upper Oldman Fm only (Unit 2, Comrey sst; and unit 3, "upper muddy").

----

## East

### FORT UNION Fm

**Notes on the individual members:**

full time duration for the individual members of the Fort Union Fm are not shown here. Instead it is only shown that the combined Lebo & Tullock Mbrs in MT are equivalent to the Ludlow Mbr, ND

----

### 66.06 ± 0.04

**Swisher et al. (1993)**

IrZ coal

present within the iridium clay that marks the K-Pg

65.16 +/- 0.04 Ma (1 SE); (Ar / Ar, sanidine, 9 crystals, Swisher et al., 1993)

65.16 +/- 0.39 Ma (1 SE); (Ar / Ar, plagioclase, 4 crystals, Swisher et al., 1993)

65.99 +/- 0.12 Ma (Ar / Ar, sanidine; Kuiper et al., 2008, recalibrated from Swisher et al., 1993)

**66.06 +/- 0.04** (1 SE); (Ar / Ar, sanidine, recalibrated here from Swisher et al., 1993)

**Standard**

Swisher et al. (1993) use the FCT and λT pairing of Samson & Alexander (1987), and Steiger & Jaeger (1977).

**Notes**

This is essentially the age of the K-Pg boundary.

There is a slight issue in that the IrZ horizon is also sampled and dated by Sprain et al. (2014) who retrieved a similar age (66.043 +/- 0.010 / 0.043 Ma). However, Sprain et al. (2014) use the Ar / Ar standard-constant pairing of Renne et al. (2011), which typically yields ages ~0.25 m.y. older than the Kuiper et al. (2008) standards used in this chart. If recalibrated to the Kuiper et al. (2008) standard the Sprain et al. (2014) date becomes 65.836 Ma, ie. slightly younger than analyses that use the Kuiper et al.(2008) standard. This is not a major issue, however, it is of interest because the only radiometric date recovered from within the Hell Creek Fm is 66.289 Ma for the Null Coal, published in the same paper by Sprain et al. (2014); this is important as it occurs 0.246 m.y. before the K-Pg boundary date, therefore demonstrating that the upper third of the Hell Creek Fm was deposited over this relatively short time (see individual entry for discussion).

Note that the recalibration here (66.06 Ma) of the date from Swisher et al. (1993) does not match precisely with a previous recalibration (65.99 Ma; Kuiper et al., 2008). Through retrocalculation it can be shown that this previous recalibration (65.99 Ma) does not incorporate change in the decay constant. This information is included here not as a criticism, but simply to explain the small difference (0.07 Ma) in the recalibrated dates, which might be of greater significance to other works.

**Recalibration (Fowler, this article)**

Legacy dates; FCT at 27.84 (Samson & Alexander, 1987); legacy λT at 5.543 +/- 0.010 E-10/y (Steiger & Jaeger, 1977).

65.16 +/- 0.04 Ma (1SE); (Ar / Ar, sanidine, 9 crystals; Swisher et al., 1993)

1st recalibration; FCT at 28.201 +/- 0.023 Ma (1σ; Kuiper et al., 2008), and λT at 5.463 E-10/y +/- 1.07 E-11/y (1σ; Min et al., 2000)

66.006 +/- 0.04 Ma (1SE); (recalibration, this article)

2nd recalibration (for reference); FCT at 28.294 +/- 0.294 Ma (1σ), and λT at 5.531 E-10/y +/- 1.35 E-12/y (1σ; both Renne et al., 2011).

66.213 +/- 0.04 Ma (1SE); (recalibration, this article)

----

### HELL CREEK Fm

**Hell Creek Fm, MT**

In Eastern Montana, the Hell Creek Fm comprises 80-90m of terrestrial floodplain sandstones and mudstones (Brown, 1907; Flight, 2004; Hartman et al., 2014; although Sheehan et al., 2000, suggest ~120 m). Generally, the Hell Creek Fm (and regional equivalents) thins to the north, and thickens to the south. More localized variations may be dependent on incision depth of the Basal Sand, which can incise up to ~15 m through the Fox Hills Formation (Flight, 2004). However, variation in measured thickness can also be due to differences in definition of the basal formational contact; for example (according to Collier & Knechtel, 1939), Brown (1907) includes the white Colgate Sandstone within the Hell Creek Basal Sand, whereas it is currently considered the uppermost part of the Fox Hills Fm.

**Lithostratigraphy**

Subdivisions of the Hell Creek Fm have been suggested by many workers. Brown (1907) and Flight (2004) both consider a "Lower" Hell Creek to comprise the basal contact through to the top of the Basal Sand. Although justifiable on lithostratigraphic grounds, this is not a useful division since under this definition, the lower Hell Creek means effectively the same thing as the Basal Sand.

Here I illustrate lower, middle, and upper subdivisions of the Hell Creek Formation as outlined by Fowler (2016; and in part Flight, 2004). This is correlative with the subdivision of the Hell Creek into "thirds" by Horner et al. (2011). Lower, middle, and upper units are based on depositional cycles each of which comprises a basal scour (depositional hiatus), followed by an amalgamated channel sandstone, fining upwards into fine grained floodplain deposits. The lower cycle comprises the Basal Sandstone and ~15 m of overlying fines; the middle cycle comprises the Jen Rex Sand and ~ 20 m of overlying fines; the upper cycle (composed of two shorter cycles) comprises the Apex Sand and ~ 5 m of overlying fines, followed by the 10 Meter Sand and ~ 7 m of overlying fines. Named amalgamated channel sands are described in detail by Hartman et al. (2014).

**Age**

Based on radiometric dates and magnetostratigraphy, it is likely that the Hell Creek Fm of Montana represents the final 1 m.y. or less of the Cretaceous.

In Montana, the upper formational contact with the overlying Fort Union Fm is roughly coincident with the K-Pg boundary, and is therefore ~66.0 Ma (Kuiper et al., 2008; Gradstein et al., 2012). The contact occurs at the base of the z-coal, part of a complex of coals which contain numerous radiometrically dated ashes (see individual entry).

Age determination of the basal contact (and therefore duration of the unit) has been more difficult to place precisely, but can be roughly constrained based on magnetostratigraphy and possibly comparison to the age of the Battle Fm of Alberta. Lerbekmo (2009) demonstrated that the Fox Hills Formation is of reversed polarity immediately below the contact with the Colgate Sandstone (similarly observed in the Canadian equivalent Whitemud Formation; Lerbekmo, 1985). Lerbekmo assigns this reversed polarity zone to C30r (68.196 to 68.369 Ma; Ogg, 2012). This would be consistent with biostratigraphic analysis of ammonites from the Fox Hills Formation of North Dakota (Landman and Waage, 1993; Landman et al., 2004). If these magnetostratigraphic zone designations are correct, then the base of the Hell Creek Fm can therefore be constrained to no older than ~68.0 Ma. This may be further constrained by an unusual 5m thick lithofacies that occurs immediately above the Colgate Sandstone in very limited areas of Hell Creek itself (Fowler, 2016). This unit bears palynomorphs allied to the Battle Fm of Alberta (Lerbekmo, 2009) and is lithologically comparable to the Battle Fm of Alberta and Saskatchewan, to which it is here tentatively correlated (Fowler, 2016). The Battle Fm in Alberta bears a radiometric date of 66.97 Ma (recalibrated here; see individual entry). Thus, if this unit is indeed the Battle Fm equivalent, then it may further constrain the age of the Hell Creek Fm to less than a million years in duration, which is supported by other chronostratigraphic data (see below).

Within the Hell Creek Fm there are two horizons that provide additional stratigraphic control. Firstly, the 30n-29r magnetostratigraphic boundary (66.398 Ma; Ogg, 2012) occurs ~30 m below the upper contact / K-Pg boundary (~66.0 Ma; see above), at the base of (or within) the Apex Sand which forms the basal unit of the upper third. Second, an Ar / Ar radiometric date has been recovered from an ash preserved in the "null coal" which occurs in the uppermost part of the middle third of the Hell Creek Fm, immediately below the Apex Sand (also therefore ~30 m below the upper contact). The radiometric date is 0.246 m.y. older than the K-Pg boundary IrZ coal (Sprain et al., 2014; see individual entries for discussion of recalibration). Both of these additional data suggest that the upper third of the Hell Creek Fm represents <300ky; if this is typical of Hell Creek Fm depositional / accommodation rates, then the entire formation was probably deposited in a million years or less.

----

### upper

**Upper third**

The upper Hell Creek is informally defined as two depositional cycles (Horner et al., 2011; Fowler, 2016) comprising the Apex Sand and ~5 m of overlying fines, followed by the 10 Meter Sand and ~ 7 m of overlying fines (named amalgamated channel sands are described in detail by Hartman et al., 2014).

**Age**

Age of the upper Hell Creek Fm is constrained by radiometric dates and magnetostratigraphy.

First, the 30n-29r magnetostratigraphic boundary (66.398 Ma; Ogg, 2012) occurs ~30 m below the upper contact / K-Pg boundary (~66.0 Ma; see above), at the base of (or within) the Apex Sand which forms the basal unit of the upper third. Second, an Ar / Ar radiometric date has been recovered from an ash preserved in the "null coal" which occurs in the uppermost part of the middle third of the Hell Creek Fm, immediately below the Apex Sand (also therefore ~30 m below the upper contact). The radiometric date is 0.246 my older than the K-Pg boundary IrZ coal (Sprain et al., 2014; see individual entries for discussion of recalibration) which occurs at the top of the upper third and is dated at ~66.0 Ma (Kuiper et al., 2008; Sprain et al., 2014; see individual entry).

The ceratopsid dinosaur *Triceratops* *prorsus* has only been recovered from the upper third of the Hell Creek Formation and stratigraphic equivalents in Canada, with specimens of *Triceratops* recovered from lower in the formation exhibiting a different morphology (Scannella et al., 2014). *T.* *prorsus* should therefore be considered an index taxon of this latter ~250 ky of the Late Cretaceous.

----

### middle

**Middle third**

The middle Hell Creek is informally defined as one depositional cycle (Horner et al., 2011; Fowler, 2016) comprising the Jen Rex Sand and ~20 m of overlying fines (named amalgamated channel sands are described in detail by Hartman et al., 2014).

**Age**

Age of the middle Hell Creek Fm is constrained by a radiometric date and magnetostratigraphy. First, the 30n-29r magnetostratigraphic boundary (66.398 Ma; Ogg, 2012) occurs ~30 m below the upper formational contact / K-Pg boundary (~66.0 Ma; see above), at the base of (or within) the Apex Sand which forms the basal unit of the upper third. Second, an Ar / Ar radiometric date has been recovered from an ash preserved in the "null coal" which occurs in the uppermost part of the middle third of the Hell Creek Fm, immediately below the Apex Sand (also therefore ~30 m below the upper contact). The radiometric date is 0.246 m.y. older than the K-Pg boundary IrZ coal (Sprain et al., 2014; see individual entries for discussion of recalibration).

Age of the base of the middle third (the base of the Jen Rex Sand) is unknown. The basal contact is therefore drawn here as roughly half way between the base of the lower third and the top of the middle third.

The ceratopsid dinosaur *Triceratops* *horridus* is only known from the lower part of the middle third (Scannella et al., 2014; see individual entry).

----

### ~66.3

**Sprain et al. (2014)**

Null Coal

~30 m below top of Hell Creek Fm (uppermost part of the Middle Hell Creek Formation); ~0-5 m below base of Apex sandstone.

66.289 +/- 0.051 Ma (1σ); (Ar / Ar; 2 samples, 1 x 21 crystals, 1 x 33 crystals; Sprain et al., 2014)

66.082 +/- 0.051 Ma (1σ); (recalibrated; this article; see below)

~30 m below the K-Pg boundary (incorrectly shown as ~50 m in the figure of Sprain et al., 2014; pers. obs.; Sprain pers. comm.).

**Standard**

Sprain et al. (2014) state that the calculated age is based on the calibration of Renne et al. (2011) which must therefore be recalibrated to the Kuiper et al. (2008) / Min et al. (2000) standard in order to be directly comparable to other dates on this chart.

C30n - C29r boundary & Hell Creek Fm age

This radiometric date is very important for two reasons;

1. it constrains the age of the base of magnetozone C29r: the Null coal occurs 0-5 m below the base of the ~5 m thick Apex sandstone, which marks the base of the Upper Hell Creek Formation in the type area of Montana (Hartman et al., 2014). Although polarity of the Apex sandstone itself is not testable, polarity below the Apex sandstone is normal (ie. C30n; including up to 5 m of mudstones that sometimes occur between the Null coal and base of the Apex sandstone; Fowler, dissertation) and polarity above the Apex sandstone is reversed (i.e. C29r; LeCain et al., 2014). Therefore, the boundary between C30n and C29r occurs either at the base of the Apex sandstone, or somewhere within the sandstone itself.

2. It is the only radiometric date from within the Hell Creek Fm, and permits us a better understanding of how much time the entire formation represents.

The Null coal date reported by Sprain et al. (2014) is 0.246 m.y. older than the date they give for the IrZ (66.043 +/- 0.010 / 0.043 Ma), thereby demonstrating that the upper third of the Hell Creek Fm was deposited in the final ~0.246 m.y. of the Cretaceous. This is important, however, there is a slight issue in that when the dates of Sprain et al. (2014) are recalibrated to the standards of Kuiper et al., (2008), then the K-Pg is recovered at 65.836 Ma, and the Null coal at 66.082 Ma. This conserves the ~0.246 m.y. gap, but moves the K-Pg boundary up past the 66.06 Ma date offered here (recalibrated from Swisher et al., 1993). As the analyses of Sprain et al., (2014) were all conducted in the same laboratory, then they are probably very reliable with respect to each other; any issues with the actual numbers are probably only reflective of the differing standards. Hence here I show the date as ~66.3 Ma, demonstrating that it is ~0.25 m.y. older than the K-Pg itself.

**Recalibration**

Original dates; FCT at 28.294 +/- 0.294 Ma (1σ), and λT at 5.531 E-10/y +/- 1.35 E-12/y (1σ; both Renne et al., 2011).

66.289 +/- 0.051 Ma (1σ); (Ar / Ar; 2 samples, 1 x 21 crystals, 1 x 33 crystals; Sprain et al., 2014)

Recalibration; FCT at 28.201 +/- 0.023 Ma (1σ; Kuiper et al., 2008), and λT at 5.463 E-10/y +/- 1.07 E-11/y (1σ; Min et al., 2000)

66.082 +/- 0.051 Ma (1σ); (linear recalibration, this article)

----

### lower

**Lower Hell Creek**

The lower Hell Creek (lower third) is here informally defined as one depositional cycle (Horner et al., 2011; Fowler, in prep) comprising the Basal Sand and ~15 m of overlying fines (named amalgamated channel sands are described in detail by Hartman et al., 2014). The Basal Sand is typically ~5 m thick, but can be up to 25 m, or represented only as a surface of non-deposition. In areas where the Basal Sand is especially thick (e.g. the new type section; Hartman et al., 2014; see Fowler, 2016, for comment) it might add an additional 20 m or so to the overall thickness of the formation (typically ~85 m).

**Age**

Age determination of the basal contact can be roughly constrained based on magnetostratigraphy and possibly comparison to the age of the Battle Fm of Alberta. Lerbekmo (2009) demonstrated that the Fox Hills Formation is of reversed polarity immediately below the contact with the Colgate Sandstone (similarly observed in the Canadian equivalent Whitemud Formation; Lerbekmo, 1985). Lerbekmo assigns this reversed polarity zone to C30r (68.196 to 68.369 Ma; Ogg, 2012). This would be consistent with biostratigraphic analysis of ammonites from the Fox Hills Formation of North Dakota (Landman and Waage, 1993; Landman et al., 2004). If these magnetostratigraphic zone designations are correct, then the base of the Hell Creek Fm can therefore be constrained to no older than ~68.0 Ma. This may be further constrained by an unusual 5m thick lithofacies that occurs immediately above the Colgate Sandstone in very limited areas of Hell Creek itself (Fowler, 2016). The Battle Fm in Alberta bears a radiometric date of 66.97 Ma (recalibrated here; see individual entry).

Age of the base of the overlying Jen Rex Sand is unknown. The upper contact is therefore drawn here as roughly half way between the base of the lower third and the top of the middle third.

----

### BATTLE Fm

**?Battle Fm**

In very limited areas of Hell Creek itself, an unusual 5m thick lithofacies comprising dark organic-rich shale and purple-mauve mudstones occurs immediately above the tidal flat lithofacies which overlies the Colgate Sandstone (Fowler, 2016). This unit bears palynomorphs allied to the Battle Fm of Alberta (Lerbekmo, 2009) and is lithologically comparable to the Battle Fm of Alberta and Saskatchewan, to which it is here tentatively correlated (Fowler, 2016).

Here the unit is shown within the Hell Creek Fm, rather than as a formation in its own right.

**Age**

Lerbekmo (2009) assigned the unit to C30n, the same as the underlying Colgate Sandstone and overlying lower third of the Hell Creek Fm. In Alberta, the Battle Fm bears a radiometric date of 66.97 Ma (recalibrated here; Hicks et al., 2003; see individual entry), which is how I have plotted it here.

Although only encountered rarely, the unit may prove to be of great importance as if it is truly correlative with the Battle Fm of Alberta and Saskatchewan, then it helps place an additional constraint on the duration of the overlying Hell Creek Fm.

----

### "Tidal flats"

**"Tidal flats": uppermost part of Colgate depositional cycle**

A tidal flat lithofacies occurs between the top of the Fox Hills Formation (Colgate Sandstone or Timber Lake equivalent facies), and beneath the Basal Sand of the Hell Creek Formation (Flight, 2004; Fowler, 2016). The distinctive facies was not mentioned by Brown (1906) in his definition of the Hell Creek Formation, nor in the description of the new type section (Hartman et al., 2014), such that it is not typically considered in publications concerning Hell Creek Formation stratigraphy. The tidal flat facies is not present at the type section locality due to deep incision of the Hell Creek Basal Sand (pers. obs.; contra Hartman et al., 2014).

**Lithostratigraphy & Sequence Stratigraphy**

The tidal flat facies creates some problems depending on the formation to which it is assigned. In the only explicit discussion of the facies, Flight (2004) includes it as the basalmost unit of the Hell Creek Formation (later followed by Behringer, 2008). However, the tidal flat facies is considered as the final part of the depositional cycle that includes incision and deposition of the Colgate Sandstone (Flight, 2004, Fowler, 2016). Hence, under Flight's (2004) definition, the Hell Creek and Fox Hills Formations are conformable, but a hiatus exists within the Hell Creek Formation, between the top of the tidal flat facies and base of the overlying Basal Sand.

**Age & Duration**

As with the underlying Colgate Sandstone, the tidal flat facies falls within the lower part of magnetozone C30n (Lerbekmo, 2009), and an upper age limit is constrained only by comparison to the Whitemud Fm, Canada (see entry for Colgate and Whitemud). The tidal flat facies is shown here as a single cell thickness, which equates to 100k.y., but this is for convenience's sake; the true duration is unknown.

----

### FOX HILLS Fm

**Fox Hills Fm**

Maximum thickness of 18m in study area (Flight, 2004).

**Lithostratigraphy**

The Fox Hills Formation is a variable collection of facies deposited during regression of the Western Interior Seaway, mostly comprising sets of coarsening upward deltaic facies (with the exception of the uppermost unit, the Colgate Sandstone).

In the type area of South Dakota, the Fox Hills has been subdivided into the Trail City, Timber Lake, and Iron Lightning members, each composed of two defined lithofacies (Waage, 1968). In Eastern Montana, the Fox Hills is notably thinner than in the Dakotas, and although it bears comparable facies, its only defensible division is that the Colgate Sandstone is considered as the uppermost member. Collier and Knechtel (1939) define a Lower Mbr of the Fox Hills Formation, but their lithological descriptions of this and the overlying Colgate Mbr do not match with my personal experience, nor the descriptions of Thom & Dobbin (1924), Waage (1968), or Flight (2004), in that their Colgate Sandstone is described as "light brown", whereas the defining characteristic of the Colgate Sandstone is that it is conspicuously white, or greyish-white (see refs above).

For these reasons I have chosen to follow the lithostratigraphic terminology of Flight (2004; despite this being a master's thesis), since it is the best documented and defensible account for the Fox Hills of Eastern Montana. However, my own work (Fowler, 2016) addresses the Fox Hills stratigraphic nomenclature issue, and may informally apply some of the members described by Waage (1968).

**Upper contact**

The upper contact is complex, depending on which units are present. Technically, the uppermost unit of the Fox Hills is the estuarine valley-fill deposit of the Colgate Sandstone (Flight, 2004). Conformably overlying the Colgate Sandstone is a grey siltstone and organic-rich horizon, which are assigned to the base of the Hell Creek Formation by Flight (2004; see individual entry), despite being the final part of the Colgate depositional cycle. This organic horizon is then usually overlain by the Basal Sand of the Hell Creek Formation, which locally may incise down through the Fox Hills Formation, as deep as the Bearpaw shale.

**Lower contact**

The lower contact with the underlying marine Bearpaw Shale is conformable and gradually occurs through transitional facies, which are variably assigned to either the uppermost Bearpaw / Pierre Shale, or the lowermost Fox Hills. For example, in Colorado, the transitional facies is considered the uppermost part of the Pierre Shale (Upper Transitional Member), whereas in Montana and the Dakotas the transitional facies is considered the lowermost part of the Fox Hills. This transitional facies is called the Trail City Member in South Dakota (Waage, 1968), but is not named in Montana although facies descriptions in Waage (1968) match observed outcrop.

**Age**

Age of the non-Colgate Sandstone part of the Fox Hills Formation is constrained by magnetostratigraphy and ammonite occurrences to the *B. clinolobatus* zone (70.44 - 69.91 Ma; Ogg & Hinnov, 2012) within magnetozone C31r (71.449 - 69.269 Ma; Ogg & Hinnov, 2012).

Progradation of the deltaic Fox Hills Sandstone (excluding the estuarine Colgate Sandstone) is not uniform west to east. Gill & Cobban (1973) document development of the Sheridan Delta (lower Fox Hills Formation) in the Upper Campanian - Lower Maastrichtian of Wyoming and Montana. They show progradation of the Lennep Sandstone beginning earlier in western Montana, around the time of the *B. eliasi* ammonite zone (uppermost Campanian; 72.74 - 72.05 Ma; Ogg & Hinnov, 2012). Later, the Sheridan Delta progrades northeasterly into eastern Montana from an origin in north-central Wyoming, beginning during the *B. baculus* ammonite zone, with marine conditions (the Bearpaw Shale) persisting in some areas until the beginning of the *B. grandis* ammonite zone (Lower Maastrichtian; 71.13- 70.44 Ma; Ogg & Hinnov, 2012). As such, in eastern Montana the upper part of the Fox Hills Formation (excluding the Colgate Member) is probably no younger than the *B. clinolobatus* zone, and possibly slightly older.

This does not agree with the named magnetostratigraphic subzones of Lerbekmo (2009) who took magnetostratigraphic samples from the Manaige Spring section of the Marina Road at Hell Creek itself (north of Jordan, Montana), and recovered the Colgate Sandstone as normal polarity (assigned to C30n), with the underlying Fox Hills Fm as mostly reversed polarity, with a short normal subchron about halfway through. These are assigned to 30r, and 31n.1n and 31n.1r. The new ages (Ogg & Hinnov, 2012) for C30r (68.369 - 68.196 Ma) and C31n (69.269 - 68.369 Ma) are incompatible with the new range for *B. clinolobatus* (70.44 - 69.91 Ma; Ogg & Hinnov, 2012) identified by Gill & Cobban (1973; see above). Given that a hiatus probably exists at the base of the Colgate Mbr (Lerbekmo, 2009; Fowler, 2016), then it seems likely that the reversed zones identified in the Fox Hills Fm by Lerbekmo (2009) actually correspond with C31r (71.449 - 69.269 Ma; Ogg & Hinnov, 2012).

----

### Colgate sst

**Colgate Mbr, Fox Hills Fm**

The Colgate Sandstone is interpreted as an incised valley filled with estuarine deposits (Flight, 2004; Behringer, 2008).

As classically defined (Calvert, 1912; Thom & Dobbin, 1924), the Colgate Sandstone is a conspicuous white to grayish green, glauconitic, volcanic-rich sandstone, up to 15m thick in the area immediately south of Ft. Peck Lake (Flight, 2004), although a more typical thickness is 2-3 m (Fowler, pers. obs.).

This conspicuous white sandstone facies typically fines upwards into a grey siltstone (<5 cm up to 1 m thick; Fowler, pers. obs.), which is overlain by an organic-rich horizon interpreted as a tidal flat deposit (typically <2cm thickness, but sometimes up to 25 cm; Flight, 2004; Fowler, pers. obs). Often the classic white sandstone facies is absent altogether, but the thin grey siltstone and organic horizon are commonly present in this stratigraphic position, unless removed by scouring of the overlying Basal Sand of the Hell Creek Formation.

**Lithostratigraphy**

The Colgate Sandstone has been treated inconsistently regarding its definition and classification, leading to problems with lithostratigraphic nomenclature.

Firstly, the overlying siltstone and organic horizon are not considered as part of the Colgate Sandstone despite being part of the same depositional cycle (Flight, 2004; Fowler, 2016). Instead they are included as the basal beds of the Hell Creek Formation by Flight (2004) and Behringer (2008), as is shown here. This is confusing, as Brown (1907) clearly designated the Hell Creek Basal Sand to be the lowermost unit within the Hell Creek Formation, and named it as such (followed by Hartman et al., 2014, in the new stratotype). However, according to Collier and Knechtel (1939), Brown (1907) included the then unnamed Colgate Sandstone as part of the Hell Creek Formation Basal Sand. This situation has been somewhat rectified in that most current parties regard the Colgate Sandstone as the uppermost member of the Fox Hills Formation (thereby changing the original definition of Brown, 1907), but the problem with the grey siltstone and tidal flat facies remains. Fowler (2016) suggests that these facies should be removed from the Hell Creek Formation and placed with the Colgate Sandstone either as its own formation, or as the uppermost member of the Fox Hills Sandstone.

**Age**

Age of the Colgate Sandstone is constrained by magnetostratigraphy and by comparison to constraints for the Whitemud Fm, Canada. Thus, I have plotted the Colgate as synchronous with Whitemud deposition.

Working north of Jordan, Montana, Lerbekmo (2009) places the C30r-C30n boundary at the base of the Colgate Mbr, which therefore occurs in C30n, making it no older than 68.196 Ma (base of C30n; Ogg, 2012). Lerbekmo (2009) then suggests that the Colgate Sandstone is correlated to the Whitemud (Mbr / Fm) of Alberta and Saskatchewan, which is similarly dated by magnetostratigraphy as lowermost C30n (Lerbekmo & Braman, 2002; 2005; Lerbekmo, 2009; Eberth & Braman, 2012), and overlain by a radiometric date from the Battle Fm of 66.97 +/- 0.10 Ma (recalibration of Hicks et al., 2003; this article; see entry). It seems likely that the reversed zone that immediately underlies the Colgate Sandstone is not, in fact, C30r, but C31r, and that C30r and C31n are missing as a result of a considerable hiatus at the base of the Colgate Sandstone (Lawton 2008; Fowler, 2016). This is consistent with ammonite biostratigraphy of Gill & Cobban (1973; see Fox Hills note).

Eberth & Braman (2012) state that the Whitemud Mbr is placed within the lower part of C30n (Lerbekmo & Braman, 2002; 2005), with an estimated age ~67.5 - 67 Ma (Ogg & Smith, 2004), and equivalent to the base of the *Wodehouseia spinata* palynozone (Nambudiri & Binda, 1991). Under the revised magnetozone definitions of Ogg (2012), this would become ~68.196 - 67.696 Ma, as shown here.

----

### BEARPAW Fm

**Bearpaw shale**

183-348m thick, thinning to the West (Weimer, 1960; Jenkin, 1990; from Flight, 2004)

**Upper contact**

The upper contact with the overlying Fox Hills Sandstone is conformable and gradually occurs through transitional facies, which are variably assigned to either the uppermost Bearpaw / Pierre Shale, or the lowermost Fox Hills. For example, in Colorado, the transitional facies is considered the uppermost part of the Pierre Shale (Upper Transitional Member), whereas in Montana and the Dakotas the transitional facies is considered the lowermost part of the Fox Hills. This transitional facies is called the Trail City Member in South Dakota (Waage, 1968), but is not named in Montana although facies descriptions in Waage (1968) match observed outcrop.

The lower contact is not plotted in this chart.

----

# N. DAKOTA

### HELL CREEK

**Hell Creek Fm**

The Hell Creek Fm in ND is ~100-60m thick from West to East, comprising fluvial sandstones and mudstones, with occasional marine influenced units (Murphy et al., 2002).

As in Montana, age of the base of the Hell Creek Fm is poorly constrained, although the upper contact and relationship with respect to the K-Pg boundary is more tightly constrained based on magnetostratigraphy of the C29r - C30n boundary.

**Southwest ND, (Hicks et al., 2002)**

In SW ND the K-Pg boundary is coincident with the Hell Creek - Fort Union formational contact with the C29r-C30n boundary is extrapolated as occurring at 26m below the K-Pg boundary (Hicks et al., 2002).

**South-central ND (Lund et al., 2002)**

In the area immediately south of Bismarck, the C29r-C30n boundary occurs ~2-3m below the base of the Ludlow Mbr of the Ft Union Fm, placing the K-Pg boundary within the Ft Union (Lund et al., 2002).

The lower contact of the Hell Creek Fm occurs within C30n, and so is drawn here the same as that of Montana (ignoring the possible Battle Fm and basal tidal flat facies).

----

### Cantapeta tongue

**Cantapeta tongue**

A marine-brackish tongue, identified by Murphy et al. (2002), which occurs 16m below the top of the Hell Creek Fm, 40m above the Breien Mbr. The Cantapeta tongue outcrops only in the Eastern ND sections near Bismarck, ND.

The Cantapeta tongue has not been analysed magnetostratigraphically, but it is shown as occurring at the top of C30n in a general section from Lund et al. (2002), which is what I follow here.

----

### Breien Mbr

**Breien Mbr**

The Breien Mbr is a marine tongue 1.5-8.5m thick occurring 1.5-9m (av, 7m) above the Fox Hills-Hell Creek contact in south-central ND (Hoganson & Murphy, 2002). The unit varies lithologically with burrowed green-grey glauconitic sandstones & mudstones, or interbedded burrowed sands and muds. Ophiomorpha burrows occur throughout (Hoganson & Murphy, 2002).

The Breien Mbr occurs within C30n (Lund et al., 2002).

Few stratigraphically informative fossils are known from this unit. A single ammonite fragment has been identified as either Discoscaphites cf D. conradi or Jeletkytes cf. J. nebrascensis. Fossils of the oyster Crassostrea subtrigonalis are commly associated with ophiomorpha burrows.

----

### FOX HILLS Fm

**Fox Hills**

**Age**

Gill & Cobban (1973) document development of the Sheridan Delta (Fox Hills Formation) in the Upper Campanian - Lower Maastrichtian of Wyoming and Montana, and the Dakotas. They show the Sheridan Delta prograding northeasterly into eastern Montana from an origin in north-central Wyoming, beginning during the Baculites baculus ammonite zone (lowermost Maastrichtian; 72.05 - 71.13 Ma; Ogg & Hinnov, 2012), progressing through into SW North Dakota by the B. grandis zone (Lower Maastrichtian; 71.13- 70.44 Ma; Ogg & Hinnov, 2012), and through into central North Dakota by the B. clinolobatus zone (uppermost Lower Maastrichtian; 70.44 - 69.91 Ma; Ogg & Hinnov, 2012).

I have therefore followed Gill & Cobban (1973) in showing the Fox Hills Fm of North Dakota as having been deposited from the B. grandis through B. clinolobatus ammonite zones. However it should be noted that Landman & Waage (1993) show the Fox Hills Fm of South Dakota to be rather younger than what is suggested here for North Dakota.

----

# S. DAKOTA

### HELL CREEK Fm

**Hell Creek Fm**

Due to lack of good chronostratigraphic control for the South Dakota sections, the age of the South Dakota Hell Creek is only constrained by the ammonites found in the underlying Fox Hills Fm. The underlying Iron Lightning Mbr (including Colgate sst equivalent) of the Fox Hills Fm contains no ammonites, but the further underlying Timber Lake Mbr has yielded *Jeletzyktes nebrascensis* (see individual member entries).

This constrains the age of the Hell Creek as no older than *J. nebrascensis* zone, and considering the amount of deposition between the base of this zone in the lower-Fox Hills Trail City Mbr (in which the *J. nebrascensis* zone begins~68.2 Ma, see entry), likely it is rather younger.

Thus here I have simply copied across the age of the lower, middle, and upper depositional cycles of the Hell Creek Fm in Montana.

----

### FOX HILLS Fm

**Fox Hills Fm**

South Dakota includes the type locality for the Fox Hills Fm (Meek and Hayden, 1861). Ammonites collected from these strata afford good stratigraphic control (Landman & Waage, 1993). These are addressed in the entries for individual members.

----

### Iron Lightning Mbr

**Iron Lightning Mbr**

The deltaic Iron Lightning Mbr comprises the Bullhead lithofacies (proximal subaqueous delta front) overlain by the Colgate lithofacies (estuarine & fluvial sandstones and siltstones; Landman & Waage, 1993).

The Iron Lightning Mbr contains no preserved ammonite fossils. Here it is assumed to continue the *J. nebrascensis* zone through from the underlying Timber Lake Mbr. The top of the Iron Lightning Mbr is plotted to coincide with the top of the Colgate Sandstone in Montana.

----

### Timber Lake Mbr

**Timber Lake Mbr**

The Timber Lake Mbr is a grey to greenish grey sandstone that weathers to a conspicuous yellow to yellow orange, with often large concretions that weather to red and yellow (Waage, 1968). It varies locally in grain size, clay content, induration, bedding, and concretions (Waage, 1968). This is the lithology that is classically associated with the Fox Hills Formation, especially in Montana.

**Age**

The *J. nebrascensis* ammonite zone begins just below the base of the Timber Lake Mbr (Landman & Waage, 1993). The age of the base of this zone is not well understood (see entry).

----

### Trail City Mbr

**Trail City Mbr**

(Landman & Waage, 1993)

The Trail City Mbr is a light-grey weathering clayey silt (Waage, 1968).

**Age**

The *Haploscaphites nicolleti* zone continues through the Trail City Mbr encompassing most of the unit.

Other fossil cited within this zone by Landman & Waage (1993) include:

*Jeletzkytes spedeni*

*Discoscaphites conradi*

*Discoscaphites gulosus*

*Sd. lenticularis*

*B. columna*

*S. tegulatus* (var 2)

The base of the *H. nicolleti* zone lies in the underlying Elk Butte Mbr of the Pierre Shale.

The very uppermost part of the Trail City mbr lies within the *J. nebrascensis* zone, which continues into the overlying Timber Lake Mbr.

Harrell & Martin (2014) described a mosasaur inferred to be from the Trail City Mbr based on the surrounding medium-grain-size matrix. The mosasaur was associated with specimens of the ammonites *H. nicolleti J. spendeni*, *D. conradi*, and *D. rossi*.

----

### Elk Butte Mbr

**Elk Butte Mbr**

The top of the Elk Butte Mbr contains *H. nicolleti* zonal fossils. Landman & Waage (1993) show the top of the overlying Trail City Mbr of the Fox Hills Fm as coinciding with the top of the *H. nicolleti* zone. Hence the top of the Elk Butte Mbr only represents the early part of the *H. nicolleti* zone.

Other fossil cited within this zone by Landman & Waage (1993) include:

*J. spedeni*

*D. conradi*

*D. gulosus*

*Sd. lenticularis*

*B. columna*

*S. tegulatus* (var 2)

----

### Morbridge Mbr

**Morbridge Mbr**

The middle of the exposed Morbridge Mbr (as shown in Landman & Waage, 1993) contains the uppermost part of the *B. clinolobatus* zone,

The Morbridge extends above the *B. clinolobatus* zone to include an unspecified zone containing *H. melloi*, *J*. sp, and *S. tegulatus*. The age of the upper contact wth the Elk Butte Mbr is not known (see Elk Butte entry), and is here shown roughly.

----

# WYOMING

## South-Central

### ALMOND Fm

**Almond Fm, WY**

Roehler (1990) states that the upper part of the Almond Fm yields *Baculites baculus*, and that a marine equivalent low in the unit yields *B. reesidei*, thereby constraining the unit.

Regarding terrestrial vertebrate fossils Farke (2004) states that the low stratigraphic position of fossils within the formation suggests that they are probably Late Campanian in age.

Deibert & Breithaupt (2006) described some dinosaur footprints from the Almond Fm. They include a stratigraphic column where the terrestrial part of the Almond (approximately the lower half) is shown as latest Campanian.

Here I follow the stratigraphic placement of Roehler (1990).

----

## East-Central

### LANCE Fm

**Lance Fm, WY:**

In the type area, the Lance Fm comprises ~770m of terrestrial floodplain sandstones and mudstones (Clemens, 1963).

**Age**

There are few analyses of the type section of the Lance Fm. No magnetostratigraphic work has been conducted, indeed there are few published measured sections.

Jeletzky and Clemens (1965) reported a fragment of a scaphite ammonite from the Lance Formation approximately 330 m above the top of the Fox Hills Formation in eastern Wyoming; however, the specimen was not sufficiently identified to be used in biostratigraphic analysis.

As such, chronostratigraphic control is non-existent and here I have simply copied across the age range from the lower through upper depositonal cycles of the Hell Creek Fm in Montana.

----

### FOX HILLS Fm

**Fox Hills Fm, WY**

(Landman & Waage, 1993)

The Fox Hills Fm lacks formal member divisions in the Wyoming sections. Landman & Waage (1993) informally separate it into the following units:

(Lance Fm) - overlying

FH -"Colgate"-like sands and thinly interbedded sand & shale

FH - Ledgy thin-bedded sand

FH - Bluff-forming bioturbated sand

FH -Transitional silt and silty sand

(Pierre Shale Fm) - underlying

Here, Landman & Waage (1993) consider the transitional facies as the basalmost part of the Fox Hills Fm in Wyoming. This has implications for correlations with Fox Hills units in other states, as for example, Landman & Cobban (2003) consider the transitional unit as the uppermost part of the Pierre Shale in Colorado. Thus the upper Pierre in CO and lower Fox Hills in WY may be equivalent facies, although this is a lithostratigraphic correlation, and ammonite biostratigraphy shows that the prograding deltaic facies of the Fox Hills Fm is of variable age across the Western Interior.

**Age**

Biostratigraphically informative fossils are only known from the Upper Pierre Shale and "Bluff-forming bioturbated sand". Stratigraphic positions of overlying units are unknown and shown here for display only (although ammonite fragments are known from the Lance, see accompanying note).

----

### B.f. sst

**Fox Hills Fm, WY**

"Bluff forming bioturbated sand" (Landman & Waage, 1993):

Landman & Waage (1993) note that the H. birkelundi zone (69.91 - 69.30 Ma; Ogg & Hinnov, 2012) is recognised just below the base of the "bluff forming bioturbated sand" at the top of the underlying "transitional silt and silty sand" (informal members). The *H. birkelundi* zone continues through to the top of the "bluff forming bioturbated sand".

----

### PIERRE SHALE

**Pierre Shale, WY:**

Landman & Waage (1993) state that in the Red Bird / Lance Creek area, the upper part of the Pierre Shale lies within the *Baculites clinolobatus* zone (70.44 - 69.91 Ma; Ogg & Hinnov, 2012). The boundary with the overying Fox Hills Fm is drawn at the upper bound of the *B. clinolobatus* zone, as shown here.

----

# COLORADO

## North West

### WILLIAMS FORK Fm

**Williams Fork Fm, CO**

The Williams Fork Fm comprises mostly coastal plain deposits, and is of variable thickness; up to 1100-1600 m, thinning westerly to ~366 m at the UT-CO border (Hettinger & Kirschbaum, 2002). It intertongues with the marine Lewis Shale to the east (Brownfield & Johnson, 2008). Gradationally overlain by the Lewis Shale in the east, and overlain unconformably by the Eocene Wasatch Fm to the west (Hettinger & Kirschbaum, 2002; Diem & Archibald, 2005; Brownfield & Johnson, 2008).

**Age**

Age of the Williams Fork Fm varies slightly east-west, most importantly affecting age of the upper contact.

**Ammonites**

The ammonite *Exiteloceras jenneyi* (75.08 - 74.60 Ma; Ogg & Hinnov, 2012) occurs at the top of the underlying Iles Formation; *Didymoceras cheyennense* (74.60 - 74.21 Ma; Ogg & Hinnov, 2012) occurs in the lower part of the Williams Fork Formation; and Baculites reesidei (73.63 - 73.27 Ma; Ogg & Hinnov, 2012) has been recovered from a marine shale (below the Twenty Mile Sandstone Member) in the upper part of the Williams Fork Formation (Newman, 1987; Brownfield & Johnson, 2008).

In western exposures (Yampa coal field) where the Lewis Shale conformably overlies the Williams Fork Fm, *B. eliasi* (72.74 - 72.05 Ma; Ogg & Hinnov, 2012) is present at the base of the Lewis Shale (Brownfield & Johnson, 2008).

In the east, Diem and Archibald (2005) show the upper boundary of the Williams Fork Fm as roughly correlative with the *B. baculus* zone (basalmost Maastrichtian, 72.05 Ma; Ogg & Hinnov, 2012). This is based on pollen recovered by Cullins (1971), which was noted as similar to pollen from the Fox Hills Fm and basal part of the Lance Fm, WY. Gill and Cobban (1973) show the progradation of the Fox Hills Fm of Wyoming beginning during the *B. baculus* zone (earlier than in Montana).

**Radiometric date**

Brownfield & Johnson (2008) report a K - Ar date of 72.5 ± 5.1 Ma from the Yampa bed in the lowermost Williams Fork Fm (see individual note), which should reside within the *D. cheyennense* ammonite zone (74.60 - 74.21 Ma; Ogg and Hinnov, 2012; see above). Although these ages do not overlap, the large error of the radiometric date permits it to occur within the *D. cheyennense* zone.

Hence here I have plotted the Williams Fork Fm as occurring between the *D. cheyennense* and *B. baculus* ammonite zones.

----

### 72.5 ± 5.1

**Brownfield & Johnson (2008)**

Yampa bed tonstein

72.5 ± 5.1 Ma (K / Ar; low-K plagioclase [andesine])

Brownfield & Johnson (2008) describe the Yampa bed tonstein, a regionally persistent diagenetically altered ash, that occurs extremely low in the Williams Fork Fm, 0-80 m above the top of the Trout Creek Sandstone (uppermost member of the underlying Iles Fm).

Regarding age, Brownfield and Johnson (2008; p. 15) state that a number of different radiometric dating methods were attempted but that only K / Ar was successful:

"... using K-Ar methods on andesine, the unit was dated at 72.5 ± 5.1 Ma (Richard Marvin, U.S. Geological Survey, written commun., 1983)."

No further details of the analysis are given. From this it should be concluded that the 72.5 Ma date resulted from an analysis performed in 1983 or earlier (the Yampa bed itself was discovered in 1977).

Note that Hofmann et al. (2011) give a different age of 72.2 ± 0.1 Ma, and attribute this to Brownfield & Johnson (2008). Indeed in their abstract, Brownfield & Johnson (2008) state that:

"The Yampa Bed is dated at 72.2 ± 0.1 mega-annum (Ma) using the K-Ar method"

However, this date is not mentioned again. Instead, the 72.5 ± 5.1 Ma date is given twice (p. 15 & 30). I do not know why the date given in the abstract differs from the main text, but Brownfield & Johnson (2008) make a specific point on p. 15 about the high analytical error of the andesine used for the K-Ar method, so it seems unlikely that the 0.1 Ma error in the slightly younger date cited by Hofmann et al. (2011) is correct.

**Ammonite inconsistency**

Note that a 72.5 Ma age for the lowermost Williams Fork Formation is inconsistent with ammonite biostratigraphy (see main comment), although this is countered by the large analytical error for this date (±5.1 Ma). Reanalysis of the Yampa bed tonstein using modern Ar / Ar methodology is desirable to eliminate this issue, and refine the age of the Williams Fork Fm.

----

### Iles Fm

**Iles Fm, CO**

**Age**

The uppermost member, the Trout Creek sst, contains *Exiteloceras jennyi* (75.08 - 74.60 Ma; Ogg & Hinnov, 2012); *B. perplexus* (79.01 - 78.34 Ma; Ogg & Hinnov, 2012) occurs in the uppermost part of the underlying Mancos Shale (Newman, 1987; Brownfield & Johnson, 2008).

Also known as the Mount Garfield Formation (see Hettinger & Kirschbaum, 2002), and a lateral equivalent (at least in part) of the Neslen Formation, Utah.

----

## Denver area

### SEQUENCE D1

**Sequence D1, Denver Basin**

The Sequence D1 is lithologically diverse and geographically variable in thickness, comprising up to ~650 m of debris flows and alluvial fans, through to distal more distal facies including lower engergy fluvial, paludal, and lacustrine deposits (Raynolds, 2002; Hicks et al., 2003).

**Age**

Sequence D1 rests disconformably on top of the Laramie Fm. The time represented by the disconformity is relatively short, but increases easterly from probably significantly less than 100ky to more than 125Ky over a distance of about 30km (my extrapolation from Hicks et al, 2003).

In the Castle Pines core the base of D1 has a reversed geomagnetic polarity, assigned to the top of C30r (Hicks et al., 2003; Raynolds & Johnson, 2003). This more or less fixes the basal age of the D1 sequence as C30r has a very short duration of ~175ky (68.369 - 68.196 Ma; Ogg, 2012) such that little time is likely to be missing.

The Kiowa core was taken ~30km ESE of the Castle Pines core, and in this section C30r was not detected in sequence D1 (Hicks et al., 2003), such that the base of the D1 sequence is slightly younger to the east.

The upper contact of the D1 sequence with the overlying D2 sequence is shown occurring within C28n by Hicks et al. (2003), hence it is not shown here.

----

### 66.14 ± 0.13

**Hicks et al. (2003); recalibration, Fowler (this article)**

Sample RSB0139

5.5m below the palynological K-T boundary

65.73 +/- 0.13 Ma (1σ); (Ar / Ar, sanidine, 7 samples: 7 x 1 crystal; Hicks et al., 2003)

**66.14 +/- 0.13** Ma (1σ); (recalibration; Fowler, this article; see below)

**Standard**

Hicks et al. (2003) state that the methods used are presented in detail in Obradovich (2002), who states that the monitor mineral used was a sanidine from the Taylor Creek Rhyolite (TCR) standard, assigned an age of 28.32 Ma (relative to MMhb-1 of 520.4). The decay constant used is not stated, but is likely to be λT = 5.543 +/- 0.010 E-10/y (Steiger & Jaeger, 1977).

**Note**

Raynolds & Johnson (2003) illustrated the date as 65.74 +/- 0.43 and cited it as from either Hicks et al (2003) or Obradovich (2002), although I have been unable to find this exact date in either publication.

**Recalibration**

A legacy FCT value of 28.03 was used, as this was given by Hicks et al (2002) as equivalent of the TCR at 28.32 (see note on TCR standard). The unusual standard is due to the particular methods of Obradovich, who ran the analysis. Legacy decay constant was assumed to have been λT = 5.543 +/- 0.010 E-10/y (Steiger & Jaeger, 1977).

For a discussion of the issues surrounding recalibration and comparison of the TCR and FCT standards in Obradovich analyses from the 1990's through to ~2002, see the Ar-Ar notes elsewhere on this chart.

Legacy dates; FCT at 28.03; legacy λT at 5.543 +/- 0.010 E-10/y.

65.73 +/- 0.13 Ma (1σ); (Ar / Ar, sanidine, 7 samples: 7 x 1 crystal; Hicks et al., 2003)

1st recalibration; FCT at 28.201 +/- 0.023 Ma (1σ; Kuiper et al, 2008), and λT at 5.463 E-10/y +/- 1.07 E-11/y (1σ; Min et al., 2000)

66.14 +/- 0.13 Ma (1σ); (recalibration, this article)

2nd recalibration (for reference); FCT at 28.294 +/- 0.294 Ma (1σ), and λT at 5.531 E-10/y +/- 1.35 E-12/y (1σ; both Renne et al. 2011).

75.271 +/- 0.097 Ma (1σ); (plagioclase)

----

### 66.37 ± 0.21

**Hicks et al. (2003); recalibration, Fowler (this article)**

Sample RSB0157

30m below palynlogical K-T boundary

65.96 +/- 0.21 Ma (1σ); (Ar / Ar, sanidine, 7 samples: 4 x 1 crystal, 3 x 2 crystal; Hicks et al., 2003)

**66.37 +/- 0.21** Ma (1σ); (recalibration; Fowler, this article; see below)

**Standard**

Hicks et al. (2003) state that the methods used are presented in detail in Obradovich (2002), who states that the monitor mineral used was a sanidine from the Taylor Creek Rhyolite (TCR) standard, assigned an age of 28.32 Ma (relative to MMhb-1 of 520.4). The decay constant used is not stated, but is likely to be λT = 5.543 +/- 0.010 E-10/y (Steiger &, 1977).

**Recalibration**

A legacy FCT value of 28.03 was used, as this was given by Hicks et al (2002) as equivalent of the TCR at 28.32 (see note on TCR standard). The unusual standard is due to the particular methods of Obradovich, who ran the analysis. Legacy decay constant was assumed to have been λT = 5.543 +/- 0.010 E-10/y (Steiger & Jaeger, 1977).

For a discussion of the issues surrounding recalibration and comparison of the TCR and FCT standards in Obradovich analyses from the 1990's through to ~2002, see the Ar-Ar notes elsewhere on this chart.

Legacy dates; FCT at 28.03; legacy λT at 5.543 +/- 0.010 E-10/y.

65.96 +/- 0.21 Ma (1σ); (Ar / Ar, sanidine, 7 samples: 4 x 1 crystal, 3 x 2 crystal; Hicks et al., 2003)

1st recalibration; FCT at 28.201 +/- 0.023 Ma (1σ; Kuiper et al, 2008), and λT at 5.463 E-10/y +/- 1.07 E-11/y (1σ; Min et al., 2000)

66.368 +/- 0.21 Ma (1σ); (Fowler, this article)

2nd recalibration (for reference); FCT at 28.294 +/- 0.294 Ma (1σ), and λT at 5.531 E-10/y +/- 1.35 E-12/y (1σ; both Renne et al. 2011).

66.576+/- 0.212 Ma (1σ); (Fowler, this article)

----

### LARAMIE Fm

**Laramie Fm**

The Laramie Fm comprises 60-300 m of fluvial sandstone, musdstones, and coal units, notably thinning to the east (Raynolds, 2002).

**Age**

Samples taken from the Laramie Fm are entirely normal in polarity in both the Castle Pines and Kiowa cores (Hicks et al., 2003). This is attributed to C31n for the Castle Pines core, and is probably the same age in the Kiowa core, although the lack of any remnant of C30r means that this cannot be confirmed.

Regardless here I have plotted the Laramie Fm to be of equal age across the Denver area.

----

### FOX HILLS Fm

**Fox Hills Fm**

In the Castle Pines core, the Fox Hills Fm is of entirely reversed polarity, assigned to C31R by Hicks et al. (2003).

The upper contact with the overlying Laramie Fm is shown occurring across the C31r - C31n boundary (Hicks et al., 2003)

The lower contact with the underlying Pierre Shale occurs within C31r (Hicks et al., 2003).

----

### FOX HILLS Fm

**Fox Hills Fm**

In the Kiowa core, the Fox Hills Fm is entirely of normal polarity, assigned to the lower part of C31n (Hicks et al., 2003).

The upper contact with the overlying Laramie Fm is shown occurring within C31n (Hicks et al., 2003)

The lower contact with the underlying Pierre Shale occurs across the C31r - C31n boundary (Hicks et al., 2003).

----

# UTAH

## Wasatch Plateau

### NORTH HORN Fm

**North Horn Fm**

403m thick in the area of North Horn Mountain (Difley & Ekdale, 2002). Approximately 75km NNE, in the region of Price Canyon / Book Cliffs, the formation is considerably thicker: up to 700m (Olsen et al., 1995; Yi & Cross, 1997). Up to half of this thickness are Paleocene sediments not fully illustrated here. Approximatey 50km west, at the Big Mountain section of the Gunniston Plateau (Axhandle Basin), the North Horn Fm is up to 1000m thick, although again, much of this is Palaeocene sediment (Talling et al., 1994).

**Lithostratigraphy**

Speiker (1946) divided the North Horn into 4 units. More recently, Difley & Ekdale (2002, shown here) divide it into 3 units. Only Units 1 & 2A are Cretaceous. Unit 2B contains Palaeocene palynomorphs, hence the boundary between Cretaceous and Paleocene rocks exists at the contact between units 2A and 2B. Unit 3 is not shown here. Talling et al. (1994) also divide the North Horn Fm of the Axhandle Basin into three units, although these are not equivalent to those of Difley and Ekdale (2002).

**Sequence stratigraphy**

In their sequence stratigraphic analysis of the North Horn Fm and underlying units in the area of Price Canyon, Olsen et al. (1995) suggest that the Cretaceous part of the North Horn Formation consists of 2 unconformity bound sequences (their sequence 4 and 5). Sequence 4 consists of the basal lag of the North Horn Fm (~10-25m thick; previously considered the uppermost bed of the underlying Price River Fm) and ~45m of overlying fines, including carbonates. This is equivalent with lithostratigraphic subunit 1A, and potentially the lowermost parts of 1B (as shown here). Sequence 5 comprises the remainder of the North Horn Fm, beginning with low accommodation amalgamated channel sands, a middle unit of isolated channels and overbank fines, and an upper unit of high-water table coals and lacustrine deposits. In sequence 5, the first productive playnomorph horizon detected by Olsen et al. (1995) was 270 m from the base of the sequence and yielded middle Paleocene palynomorphs.

Due to the sometimes indistinct nature of the boundary between sequences 4 and 5, Olsen et al. (1995) suggest that only a very short hiatus exists here. However, a considerable hiatus is indicated to exist within sequence 5 (presumably between lithostratigraphic subunits 2A and 2B), implied by later analyses of palynomorphs, microvertebrates, and stratigraphic reanalysis (see below).

**Biostratigraphy & Magnetostratigraphy**

Yi and Cross (1997) conducted a palynostratigraphic analysis in Price Canyon, and had better luck finding palynomorphs in the Cretaceous parts of the North Horn Fm than Olsen et al. (1995). Yi and Cross (1997) concluded that the North Horn Formation contains at least 2 hiatuses detectable by palynomorph analysis. Based on the absence of the Upper Maastrichtian *Wodehouseia* spp. and *Kurtzipites trispissatus* they suggest that the Cretaceous portion of the North Horn Fm represents only the lower to "middle" Maastrichtian, and that any upper Maastrichtian and lower Palaeocene deposits were either never emplaced, or were eroded away before deposition of the overlying Middle Palaeocene sediments.

Cross and Yi (1997) illustrate a regional cross section where they show the North Horn Formation having a significant hiatus (mid Maastrichtian to mid Paleocene) in the east (e.g. Price Canyon: their study section), but continuous deposition in western sections (which would include the North Horn Mountain section studied by Difley). The most recent work at North Horn Mountain is consistent with the view that hiatus exists here also (see below), so illustration of a continuous section in Cross and Yi (1997) may be due to the fact that their figure was modified from those of earlier studies (Franczyk et al., 1990; Fouch et al., 1983), rather than being based entirely on their own, or new data.

Talling et al. (1994) conducted a magnetostratigraphic analysis for North Horn Fm exposures in the Axhandle basin (~50km from the type section at North Horn Mountain) and found the Cretaceous portion of the North Horn to be of reversed polarity, correlating this with C31r (71.449 to 69.269 Ma; Ogg, 2012). Talling et al. (1994) also note that absence of the common Late Maastrichtian charophyte *Platychara compressa* from the North Horn Fm of the Axhandle basin suggests that it is pre-late Maastrichtian in age, and that ostracods from the lower part of the North Horn Fm of the Axhandle basin (Petes Canyon section) were tentatively identified as Late Campanian to early Maastrichtian in age (citing a pers. comm. in 1989 from R. M. Forester).

**K-Pg boundary in the North Horn Fm?**

The boundary between Cretaceous and Palaeocene rocks occurs 2m below the subunit 2A / 2B contact where there is a change from Cretaceous to Paleocene aspect palynomorphs (Difley, 2007). However, contrary to the historical view, there is no evidence to suggest that deposition of the North Horn Fm was continuous across the K-Pg boundary itself.

Based on the identification of purported shocked quartz (relating to the K-Pg bolide impact, and found at other K-Pg sections globally), the K-Pg boundary was placed between units 2A and 2B by Difley & Ekdale (2002). However, with further analysis (Difley, 2007), it was found that the shocked quartz was not impact-derived (also suggested in Difley and Ekdale, 1999), and thus, was not evidence in support of deposition across the K-Pg boundary.

Furthermore, preliminary magnetostratigraphic analysis of the transition between subunits 2A and 2B (10 samples) have shown normal polarity up to 4.5m either side of the subunit 2A / 2B contact. Since the K-Pg boundary lies entirely within a reversed polarity zone: C29r (Ogg, 2012), then it cannot be present within the North Horn Fm, where it is represented instead by an unconformity. This is consistent with the findings of Talling et al. (1994) working in the Axhandle Basin, ~50km from the type section at North Horn Mountain. Additionally, in their study of North Horn Fm mammals, Cifelli et al. (1999) similarly noted that there was no palaeontological or geological evidence to suggest continuous deposition across the K-Pg boundary.

It has been suggested (Difley, 2007) that the normal polarity zone recorded in subunit 2B corresponds to C30n (65.9-67.7 Ma; Ogg & Smith, 2004). However it seems reasonable to consider that this may represent the older C31n (67.8-68.7 Ma; Ogg & Smith, 2004). Indeed, if the presence of *Alamosaurus* is used as any biostratigraphic indication, the 69 Ma datum (+/- 0.9; Lehman et al., 2006) for *Alamosaurus*-bearing rocks of the Javelina Fm, TX, would indicate that this older chron might be more likely. However, unit 1 of the North Horn Fm has not been subjected to magnetostratigraphic analysis, so it is not yet known what the polarity of *Alamosaurus*-bearing rocks in Utah might be.

Despite their importance to our understanding of the age of the North Horn Fm, neither the sequence stratigraphic analysis of Olsen et al (1995) nor the palynostratigraphic work of Yi & Cross (1997) is cited in any of the works of Difley (2007) or Difley and Ekdale (1999; 2002a; 2002b). As a result, the hypothesis that the North Horn Formation preserves continuous deposition across the K-Pg boundary, and that the lower part of the North Horn is upper Maastrichtian in age (which lacks any supporting evidence) has been perpetuated in palaeontological studies (e.g. Sampson & Loewen, 2005).

----

### Unit 2

**North Horn Fm, Unit 2**

~70m thick (Difley & Ekdale, 2002)

Divided in two at the K-Pg boundary.

"consists chiefly of evenly and thinly bedded layers of dark-colored organic-rich shale, siltstone, or thin coal, and limestone, with fine-grained bioturbated sandstones."

(Difley & Ekdale, 2002)

----

### 2B

**Subunit 2B (Difley & Elkdale, 2002)**

~30m thick

Palynostratigraphic evidence (Yi and Cross, 1997) suggests that Paleocene deposits of the North Horn Fm are no older than middle Paleocene, hence the depiction here is not to scale.

----

### 2A

**Subunit 2A (Difley & Elkdale, 2002)**

~40m thick.

Dinosaur bodyfossils are limited to a partial ornithischian jaw near the base of the subunit, and various indeterminate fragments occurring in the lower half. Porous dinosaur eggshell types persist higher in the subunit, to 6m below the K-Pg disconformity.

----

### Unit 1

**North Horn Fm, Unit 1**

~165m thick (Difley & Ekdale, 2002)

"*Alamosaurus*" remains are known from the lower part of 1B.

I have correlated this unit with other SW units that contain "*Alamosaurus*". This causes a likely separation of Units 1 & 2. Unit 1 might well be somewhat younger than I have shown here, but there is no solid evidence to place it anywhere specific. The presence of *Torosaurus* *utahensis* is probably supportive of this unit being somewhat older than the Hell Creek and Lance Fms.

Since there is no definition of Edmontonian in NALMA ages, then it is entirely possible that the mammalian assemblages of Cifelli et al. (1999) are near-Lancian, but not actually Lancian. Alternatively, the Lancian might well be considered to be rather older than the restricted range seen here.

Unit 1 is considered to represent an arid environment within the intermontane basin. Sedimentologically it represents a notably different environment to the overlying unit 2, which is more coaly.

"Unit 1 (Upper Maastrichtian) consists predominantly of banded, variegated, smectitic clay mudstone and siltstone with scattered, locally common, Cretaceous caliche and iron oxide nodules. The mudstone is interbedded with fine-grained, intensely bioturbated sandstone sheets and lenses that frequently pinch out over a short distance laterally. Dark gray molluscan limestones are rare to locally common."

(Difley & Ekdale, 2002)

----

### 1C

**Subunit 1C (Difley & Elkdale, 2002)**

~55m thick.

Contains no diagnostic dinosaur bodyfossils, although 6 different eggshell morphologies are known, including 2 new kinds not seen in 1B. 2 other eggshelltypes disappear halfway through the subunit.

----

### 1B

**Subunit 1B (Difley & Elkdale, 2002)**

~100m thick.

This is the only subunit to contain "*Alamosaurus*" remains, which have been recovered from exposures extending laterally for "several km" at about the same stratigraphic horizon. The *Alamosaurus* quarry (Gilmore, 1946b) is located approximately 30m from the base of the formation (ie, ~20m into subunit 1B as 1A is ~10m thick).

Also the subunit from which *Torosaurus* *utahensis* was recovered.

----

### 1A

**Subunit 1A (Difley & Elkdale, 2002)**

~10m thick.

Only dinosaur bonescraps and footprints are known from subunit 1A

----

## East Central

### NESLEN Fm

**Neslen** **Formation**

Thickness of ~100m (320 ft; Hettinger & Kirschbaum. 2002) comprises a basal sand, followed by Palisade, Ballard, and Chesterfield coal zones, separated by zones of sandstone, siltstone, and mudstone. The Neslen was historically interpreted as being terrestrial in origin, however, Spear & Kirschbaum (2012, abstract) suggest that some of the sand units of the upper part of the Neslen Formation (between coal zones) represent shallow marine deposition during transgressive phases.

The upper and lower contacts (Farrer Formation / Castlegate Sandstone, and Sego Sandstone, respectively) of the Neslen Fm are not precisely defined (Hettinger & Kirschbaum, 2002). Kirschbaum & Hettinger (2004) interpreted the basal contact of the Neslen to represent a sequence boundary, where incisions into the underlying shoreface / tidal Sego Sandstone are filled with basal Neslen Fm tidal sandstones. Where present, the upper contact of the Neslen Fm with the superceding Farrer Fm is gradational (Franczyk et al., 1990; Hettinger & Kirschbaum, 2002), however in places the Bluecastle Tongue of the Castlegate Sandstone disconformably overlies or incises into the upper part of the Neslen Fm (Hettinger & Kirschbaum, 2002).

**Age**

Kirschbaum & Hettinger (2004) constrain the age of the Sego to Neslen Formations to the Campanian based on the occurrence of ammonite fossils, mostly in the laterally equivalent Mount Garfield Fm (Iles Fm) of Colorado.

*D. cheyennense* (base= 74.60 Ma; Ogg & Hinnov, 2012): strata "well above" Rollins (Trout Creek equivalent) Sandstone Mbr, Mount Garfield Fm (Madden, 1989); (Rollins Mbr is laterally equivalent to the base of the overlying Farrer Fm; Kirschbaum & Hettinger, 2004).

*D. stevensoni* (base= 75.64 Ma; Ogg & Hinnov, 2012): distal sands, Cozzette Mbr, Mount Garfield Fm (lateral equivalent of upper Neslen Fm; Kirschbaum & Hettinger, 2004)

*Didymoceras nebrascense* (base= 76.27 Ma; Ogg & Hinnov, 2012): "just above the Corcoran Mbr, Mount Garfield Fm (Gill & Hail, 1975)" (lateral equivalent of the middle part of the Neslen Fm; Kirschbaum & Hettinger, 2004).

*Baculites scotti* (base= 76.94 Ma; Ogg & 2012): upper part of the Sego Sandstone (E. Book Cliffs; Gill & Hail, 1975).

As such, the Neslen is shown here to range from the base of the *D. nebrascense* zone, to just above the base of the *D. stevensoni* zone.

**Correction**

Thomson et al. (2013) suggest that the Palisade coal zone of the Neslen Formation is equivalent to the *D. nebrascense* ammonite biozone, and cite a radiometric date (74.13 Ma +/- 0.28) from Izett et al. (1998) for the *D. nebrascense* ammonite zone. This date is then recalibrated to the Ar / Ar standards of Renne et a. (2010) to give a date of 75.15 +/- 0.29Ma. There are two problems: First, this new revised date does not fall within the *D. nebrascense* zone of Ogg & Hinnov (2012). This could be atributed to the fact that Ogg & Hinnov (2012) are working with the Ar / Ar methodology of Kuiper et al. (2008) rather than Renne et al. (2010). However, although comparable, if anything, dates produced using the Renne et al., (2010) method are marginally older than Kuiper et al. (2008), so it is problematic that 75.15 Ma (even taking into account error) is younger than expected. Second, (less importantly) the error margin given by Thomson et al. (2013; ie. 74.13 Ma +/- 0.28) is incorrectly cited from Izett et al. (1998), who actually give a spread of dates with error ranging from 0.14 to 0.21.

----

## Kaiparowits Plateau

### KAIPAROWITS Fm

**Kaiparowits Fm**

Thickness up to ~860m (Roberts et al., 2005)

**Lithostratigraphy**

The Kaiparowits Fm is informally divided into lower, middle, and upper units (Roberts et al., 2005; 2013).

**Sequence stratigraphy**

Lawton et al. (2003) published a terrestrial sequence stratigraphic interpretation of the Kaiparowits Fm, and underlying Wahweap and Straight Cliffs Fms outcropping across the Kaiparowits, Paunsaugunt, and Markagunt plateaus in the SW corner of Utah. They found that the Kaiparowits Fm forms a single 3rd order depositional sequence. Following this, as a result of lithostratigraphic methods, the prominent amalgamated channel complex at the base of this sequence has been defined as the uppermost part of the underlying Wahweap Fm (Capping Sandstone Mbr). This may cause some confusion when discussing the age and duration of different formations, since the Capping Sandstone Mbr of the Wahweap might not be related to the deposition of the rest of the Wahweap, and in this interpretation, is much closer in age to the Kaiparowits Fm.

However, this is a complex problem under active study. The current consensus is that a sequence boundary and hiatal surface) occurs at the top of the Capping Sandstone Mbr (Little, 1997; Titus et al., 2013).

**Chronostratigraphy**

Roberts et al. (2005) state that 8 bentonites were found through the Kaiparowits, but only four were selected for radiometric analysis (those with the most phenocryst-rich samples; shown here), two of which were closely positioned stratigraphically and yielded the same date (middle unit). Stratigraphic positions of the four undated bentonites are not figured in Roberts et al. (2005) but are figured in Roberts (2007). Later. Roberts et al. (2013) illustrate the stratigraphic position of ten bentonites; one in the lower unit (dated at 76.46 Ma), six in the middle unit (one "in progress", three dated at 75.97 Ma, 75.51 Ma, and another 75.51 Ma ), and three in the upper unit (one dated at 74.69 Ma). For more information, see comments on the inidividual dates.

Imhof and Albright (2003, JVP abstract) presented a preliminary magnetostratigraphic analysis covering an unspecified 90m section of the Kaiparowits. the entire 90m was found to be of positive polarity. This probably corresponds to C33n based on correlations made to the magnetostratigraphic column using the radiometric dates as reference. A more detailed analysis has not yet been published.

----

### upper

**Upper Kaiparowits Fm**

The upper unit of the Kaiparowits Fm is ~320 m thick (Roberts et al., 2013).

The upper boundary with the overlying Caanan Peak Fm is not precisely dated, but is estimated to occur at the top of the *Didymoceras cheyennense* ammonite zone (Roberts et al., 2005), which I show here. An Ar / Ar date of 74.69 Ma occurs ~50 m below the upper contact.

The lower boundary of the upper unit occurs at approximately 530m above the base of the Kaiparowits Fm (Roberts et al., 2013). A radiometric date of 75.51 Ma occurs ~30 m below the contact between the upper and middle units (Roberts et al., 2013; see individual entry).

----

### 74.69 ± 0.18

**Roberts et al. (2005, 2013)**

Ash KBO-37

790m above base of Kaiparowits Fm; ~70m below top of Kaiparowits Fm

"Ash bed #8" of Roberts (2007)

74.21 Ma +/- 0.18 (Ar/Ar, sanidine, n=15; Roberts et al., 2005)

**74.69** Ma +/- 0.18 (recalibration; Roberts et al., 2013; see below)

**Standard**

In the original analysis Roberts et al. (2005) used sanidine from the Fish Canyon Tuff (FCT) with a reference age of 28.02 Ma (Renne et al., 1998). The decay constant used is not mentioned by Roberts et al. (2005), but in his dissertation Roberts (2005; and in Roberts et al., 2013) shows that the analysis used a λT of 5.543 E-10/y (Steiger & Jaeger, 1977).

**Recalibration (Roberts et al., 2013)**

Roberts et al., (2013) recalibrated the older date using the 28.201 Ma FCT fluence monitor standard (Kuiper et al., 2008), and a decay constant of 5.463 E-10/y (Min et al., 2000).

**Revised dates used by Sampson et al. (2010)**

The recalibrated dates of Roberts et al. (2013) were used implicitly in the biostratigraphic analysis of Sampson et al. (2010), however, it is not stated that the date is recalibrated and only old references are cited (e.g. Roberts et al., 2005). This is important as the other dates used for comparison (e.g. those from the Dinosaur Park Formation, Alberta) are not recalibrated, misaligning the Kaiparowits Formation (and its fauna) with other formations, with important implications for biogeographic and speciation hypotheses.

**Recalibration (Fowler, this article)**

The dates cited by Roberts et al. (2013) use the Kuiper et al. (2008) FCT standard (28.201 Ma), which is used in this sheet and plotted here. However, for comparison I have recalibrated the dates using the differing Renne et al. (2011) FCT standard (28.294 Ma).

Legacy dates; FCT at 28.201 +/- 0.023 Ma (1σ; Kuiper et al, 2008), and λT at 5.463 E-10/y +/- 1.07 E-11/y (1σ; Min et al., 2000).

74.69 +/- 0.18 Ma (Ar/Ar, sanidine, n=15; Roberts et al., 2005; 2013)

Recalibration (for reference); FCT at 28.294 +/- 0.036 Ma (1σ), and λT at 5.531 E-10/y +/- 1.35 E-12/y (1σ; both Renne et al. 2011).

74.93 +/- 0.18 Ma (Fowler, this article)

----

### middle

**Middle Kaiparowits Fm**

The middle unit of the Kaiparowits is ~420 m thick (Roberts et al., 2013).

The upper boundary of the middle unit occurs at approximately 530m above the base of the Kaiparowits Fm (Roberts et al., 2013). A radiometric date of 75.51 Ma occurs ~30 m below the contact between the upper and middle units (Roberts et al., 2013; see individual entry).

The lower boundary occurs at ~110 m above the formational base (Roberts et al., 2013). An Ar / Ar date of 75.97 Ma occurs ~70 m above the contact between the lower and middle units, with an Ar / Ar date of 76.46 Ma occuring ~ 30 m below the contact (Roberts et al., 2013; see individual entry).

**Detrital zircon date**

In Jinnah et al (2009), the 04JL05 horizon sampled for detrital zircons is shown as occurring ~123 m above the base of the Kaiparowits Fm. Hence, using the older Roberts et al. (2005) stratigraphic definition (as do Jinnah et al., 2009), this would be considered as the top of the lower unit; whereas in the more recent chart (Roberts et al., 2013), it would be considered as the lowermost part of the middle Kaiparowits Fm.

----

### 75.51 ± 0.15

**Roberts et al. (2005, 2013)**

Ash KBC-144

~490m above base of Kaiparowits Fm

"Ash bed #4" of Roberts (2007)

75.02 Ma +/- 0.15 (Ar/Ar, sanidine, n=27; Roberts et al., 2005)

**75.51** Ma +/- 0.15 (recalibrated, Zanno et al., 2011; Roberts et al., 2013; see below)

Ash KBC-109

~420m above base of Kaiparowits Fm

"Ash bed #5" of Roberts (2007)

75.02 Ma +/- 0.15 (Ar/Ar, sanidine, n=23; Roberts et al., 2005)

75.51 Ma +/- 0.15 (recalibrated, Zanno et al., 2011; Roberts et al., 2013; see below)

**Standard (original analysis; Roberts et al., 2005; p. 310)**

In the original analysis Roberts et al. (2005) used sanidine from the Fish Canyon Tuff (FCT) with a reference age of 28.02 Ma (Renne et al., 1998). The decay constant used is not mentioned by Roberts et al. (2005), but in his dissertation Roberts (2005; and in Roberts et al., 2013) shows that the analysis used a λT of 5.543 E-10/y (Steiger & Jaeger, 1977).

"Samples irradiated... at Oregon State University TRIGA reactor"

"40Ar-39Ar extractions performed at the Berkeley Geochronology Center"

**Recalibrated dates (Sampson et al., 2010; Zanno et al., 2011; Roberts et al., 2013)**

Revised dates were used implicitly in the biostratigraphic analysis of Sampson et al. (2010) and stated explicitly by Zanno et al. (2011). These revisions correspond to recalculation using the revised FCT age of Kuiper et al. (2008), although this is not stated and only old references (Roberts et al., 2005; Roberts, 2007; Jinnah et al., 2009) are cited by either Sampson et al. (2010), or Zanno et al. (2011). However, using the recalibration excel shet provided by either Earthtime or Paul Renne (see below), recalibrating the original dates (Roberts et al., 2005; which used the 28.02 FCT age), using the 28.201 FCT age (Kuiper et al., 2008; available at the time of Sampson et al., 2010) yields the same ages as cited in Zanno et al., (2011), confirming that the revised dates of Sampson et al. (2010) and Zanno et al. (2011) were based on the Kuiper et al (2008) standard. This was confirmed by the publication of Roberts et al. (2013).

**Recalibrated dates (D. Fowler, this article)**

The dates cited by Zanno et al., (2011; and later, Roberts et al., 2013) use the Kuiper et al. (2008) FCT standard (28.201 Ma), which is used in this sheet and plotted here. However, for comparison I have recalibrated the dates using the differing Renne et al. (2011) FCT standard (28.294). The results are the same for both ashes as the input data were the same.

Legacy dates; FCT at 28.201 +/- 0.023 Ma (1σ; Kuiper et al, 2008), and λT at 5.463 E-10/y +/- 1.07 E-11/y (1σ; Min et al., 2000).

75.51 +/- 0.15 Ma (Ar/Ar, sanidine, n=27; Roberts et al., 2005; 2013)

Recalibration (for reference); FCT at 28.294 +/- 0.036 Ma (1σ), and λT at 5.531 E-10/y +/- 1.35 E-12/y (1σ; both Renne et al. 2011).

75.75 +/- 0.15 Ma (Fowler, this article)

----

### 75.97 ± 0.18

**Zanno et al. (2011); Roberts et al. (2013)**

KP-07

presumably "Ash bed #2" of Roberts (2007)

190m above base of Kaiparowits Fm (Roberts et al., 2013)

75.97 Ma +/- 0.18 Ma (1σ); (Ar/Ar, sanidine, n=2; Zanno et al., 2011; Roberts et al., 2013)

**76.26 Ma +/- 0.10** Ma (1σ); (U-Pb, Roberts et al., 2013)

**Standard**

Roberts et al. (2013) state that the standard used for the analysis was the Fish Canyon Tuff at 28.201 Ma (Kuiper et al., 2008). λT is shown to be 5.463 E-10/y +/- 1.07 E-11/y (Min et al., 2000).

**Mistaken reference**

Zanno et al. (2011) cite Jinnah et al. (2009) as the source for this 75.97 +/- 0.18 radiometric date from ~185m above the base of the Kaiparowits Fm. However, Jinnah et al. (2009) make no mention of a radiometric date from 185m above the base of the Kaiparowits Fm. Jinnah et al (2009) do mention a similarly aged date of 75.96 +/- 0.14, but this is the unrecalibrated date for ash KDR-5, in the lower Kapiarowits. The date was finally published with analytical details by Roberts et al., (2013).

**Recalibrated dates (D. Fowler, this article)**

The dates cited by Zanno et al., (2011; and later, Roberts et al., 2013) use the Kuiper et al. (2008) FCT standard (28.201 Ma), which is used in this sheet and plotted here. However, for comparison I have recalibrated the dates using the differing Renne et al. (2011) FCT standard (28.294).

Legacy dates; FCT at 28.201 +/- 0.023 Ma (1σ; Kuiper et al, 2008), and λT at 5.463 E-10/y +/- 1.07 E-11/y (1σ; Min et al., 2000).

75.97 +/- 0.18 Ma (Ar/Ar, sanidine, n=2; Zanno et al., 2011; Roberts et al., 2013)

Recalibration (for reference); FCT at 28.294 +/- 0.036 Ma (1σ), and λT at 5.531 E-10/y +/- 1.35 E-12/y (1σ; both Renne et al. 2011).

----

### lower

**lower Kaiparowits Fm**

110 m thick (Roberts et al., 2013).

**Differences in definition of lower-middle unit boundary**

The contact between lower and middle units is shown at the top of a thick sand unit, approximately 170m above the formational base by Roberts et al. (2005). However, Roberts et al. (2013) show the boundary between the lower and middle units occurring at ~110 m above the formational base. The more recent definition is followed here.

**Effect on position of radiometric date**

In both Roberts et al., (2005), and (2013), and in Jinnah et al. (2009), the radiometrically dated ash KDR-05 (76.46 Ma; Roberts et al., 2013) is shown as occurring 80m above the formational base . Hence, in the older Roberts et al. (2005) strat chart, this radiometric date occurs in the middle of the lower unit; whereas in the more recent chart, it is near the top of the lower unit.

**Effect on position of detrital zircon date**

In Jinnah et al. (2009), the 04JL05 horizon sampled for detrital zircons is shown as occurring ~123 m above the base of thre Kaiparowits Fm. Hence, using the older Roberts et al. (2005) stratigraphic definition (as do Jinnah et al., 2009), this would be considered as the top of the lower unit; whereas in the more recent chart (Roberts et al., 2013), it would be considered as the lowermost part of the middle Kaiparowits. See middle Kaiparowits entry for details of the analysis.

----

### 76.46 ± 0.14

**Roberts et al. (2005, 2013)**

Ash KDR-5

~80m above base of Kaiparowits Fm

"Ash bed #1" of Roberts (2007)

75.96 Ma +/- 0.14 (Ar/Ar, sanidine, n=21; Roberts et al., 2005)

76.46 +/- 0.14 (recalibrated, see below; Sampson et al., 2010; Zanno et al., 2011; Roberts et al., 2013)

**Standard (original analysis; Roberts et al., 2005; p. 310)**

In the original analysis Roberts et al. (2005) used sanidine from the Fish Canyon Tuff (FCT) with a reference age of 28.02 Ma (Renne et al., 1998). The decay constant used is not mentioned by Roberts et al. (2005), but in his dissertation Roberts (2005; and in Roberts et al., 2013) shows that the analysis used a λT of 5.543 E-10/y (Steiger & Jaeger, 1977).

Roberts et al. (2005, p. 310) also state that "samples irradiated... at Oregon State University TRIGA reactor" and "40Ar-39Ar extractions performed at the Berkeley Geochronology Center"

**Recalibrated dates (Sampson et al., 2010; Zanno et al., 2011; Roberts et al., 2013)**

Revised dates were used implicitly in the biostratigraphic analysis of Sampson et al. (2010) and stated explicitly by Zanno et al. (2011). These revisions correspond to recalculation using the revised FCT age of Kuiper et al. (2008), although this is not stated and only old references (Roberts et al., 2005; Roberts, 2007; Jinnah et al., 2009) are cited by either Sampson et al. (2010), or Zanno et al. (2011). However, using the recalibration excel sheet provided by either Earthtime or Paul Renne (see below), recalibrating the original dates (Roberts et al., 2005; which used the 28.02 FCT age) using the 28.201 FCT age (Kuiper et al., 2008; available at the time of Sampson et al., 2010) yields the same ages as cited in Zanno et al., (2011), confirming that the revised dates of Sampson et al. (2010) and Zanno et al. (2011) were based on the Kuiper et al (2008) standard. This was confirmed by the publication of Roberts et al. (2013).

**Recalibrated dates (D. Fowler, this article)**

The dates cited by Zanno et al., (2011; and later, Roberts et al., 2013) use the Kuiper et al. (2008) FCT standard (28.201 Ma), which is used in this sheet and plotted here. However, for comparison I have recalibrated the dates using the differing Renne et al. (2011) FCT standard (28.294).

Legacy dates; FCT at 28.201 +/- 0.023 Ma (1σ; Kuiper et al, 2008), and λT at 5.463 E-10/y +/- 1.07 E-11/y (1σ; Min et al., 2000).

75.96 +/- 0.14 Ma (Ar/Ar, sanidine, n=21; Roberts et al., 2005; 2013)

Recalibration (for reference); FCT at 28.294 +/- 0.036 Ma (1σ), and λT at 5.531 E-10/y +/- 1.35 E-12/y (1σ; both Renne et al. 2011).

76.70 +/- 0.14 Ma (Fowler, this article)

----

### WAHWEAP Fm

**Wahweap Fm**

360-460m thick, averages ~400m (Eaton 2002)

**Lithostratigraphy**

Two different classifications have been proposed for the internal lithostratigraphy of the Wahweap Fm. here I follow the more generally accepted nomenclature of Eaton (1991) who divided the formation into four informal members: the lower, middle, upper, and capping sandstone. Doelling (1997) alternatively proposed two informal units: an upper member consisting of cliff forming sandstone, and a lower member comprising interbedded sandstone and mudstone. Jinnah et al. (2009) suggests that the difference between the two schemes represents regional variation in facies and thickness.

**Sequence Stratigraphy**

The capping sandstone that forms the uppermost part of the Wahweap is sequence stratigraphically unrelated to the rest of the formation. Instead, the capping sandstone Mbr forms the basal amalgamated channel complex belonging to the overlying Kaiparowits depositional sequence (Lawton et al., 2003; Jinnah & Roberts, 2011). Similarly, the Drip Tank Mbr of the Straight Cliffs Fm that immediately underlies the Wahweap Fm has been interpreted to be the basal amalgamated channel complex of the Wahweap depositional sequence, with the remainder of the sequence comprised of the lower, middle and upper members of the Wahweap Fm (Lawton et al., 2003; although see individual note, below).

However, this is a complex problem under active study. The current consensus is that a sequence boundary and hiatal surface) occurs at the top of the Capping Sandstone Mbr (Little, 1997; Titus et al., 2013).

This situation can cause some confusion when discussing the age of the Wahweap Fm, since it contains parts of two depositional sequences (hence two discrete packages of relatively continuous deposition), but neither is complete within the Formation itself (as defined lithostratigraphically). Moreover, it is likely that considerable hiatus exists between the two sequences. As such it is best understood by graphical representation, as shown here.

**Chronostratigraphy**

The age of the Wahweap Formation and sequence is not well known. A bentonite from the lowermost part of the middle Wahweap (~40m above base of the Formation; although see chart entry) yielded an ash date of 80.1 +/-0.3 (Jinnah et al., 2009). Detrital zircon analysis has placed maximum ages on some Wahweap units. Sample 01JL05 was collected ~20m above the base of the lower mbr, and yielded detrital zircons with an age of 82Ma +/- 2 (Jinnah et al., 2009).

Jinnah et al. (2009) suggested that the Wahweap ranged from ~80.6-80.3Ma to ~77.5-76.1Ma, basing this estimate on an extrapolation of average sedimentation rate (calculated based on the thickness of sediment between the 80.1Ma ash date in the middle Wahweap, and the 75.96Ma ash date in the lower Kaiparowits).

Albright and Titus (2016) analysed the magnetostratigraphy of the Straight Cliffs and Wahweap fms. They showed the lower and middle Mbrs as reversed polarity (C33r). The upper Mbr is reversed polarity for most of its thickness, but the uppermost ~20 m are of normal polarity, which Albright & Titus asign to the C33r - C33n boundary. Albright & Titus (2016) then revise the C33r-C33n boundary to 78.91 Ma based on the presence of an Ar / Ar date of 79.9 +/-0.3 Ma in the middle mbr.

----

### capping sst mbr

**Capping sandstone mbr, Wahweap Fm:**

Exact age of the capping sandstone is not known. Detrital zircons extracted from a channel sandstone ~2 m above the base of this unit (below a conglomeritic layer) yielded a U-Pb SHRIMP age of 77Ma +/- 2 (Jinnah et al., 2009).

Albright & Titus (2016) performed a magnetostratigraphic analysis on the Wahweap and Straight Cliffs fms. They show the basal age of the capping sandstone Mbr as ~77Ma, which is followed here.

Chronostratigraphic indicators (radiometric dates, playnomorphs and other biostratigraphic indicators) are therefore more likely to be in closer alignment with the Kaiparowits Fm than to other members of the Wahweap Fm.

----

### upper

**upper Mbr:**

Albright & Titus (2016) show the C33r-C33n boundary occurs ~20m below the upper contact of the ~120 m thick upper Mbr.

----

### 79.9 ± 0.3

**Jinnah (2013); Roberts et al. (2013)**

Bentonite SS07B (Roberts et al., 2013)

60m above base of Wahweap Fm (Jinnah, 2013; Roberts et al., 2013)

**79.9 +/- 0.3** (Ar-Ar, sanidine, n=2; Jinnah, 2013)

Jinnah et al. (2013) state that the SS07B bentonite occurs ~10 m stratigraphically higher within the same "Star Seep" section as the CF05B tuff, dated at 80.6 Ma (J innah et al., 2009; recalibrated here). Based on the illustration of the Star Seep section given by Jinnah (2013) this would place the SS07B bentonite as ~ 60 m above the base of the Wahweap Fm, at roughly the middle of the middle member.

**Standard**

Fish Canyon Tuff standard of 28.201 (Kuiper et al., 2008).

----

### lower

**lower Mbr, Wahweap Fm**

The age of the lower Mbr is not precisely known. Detrital zircons recovered from the lower Mbr yield a youngest age of 82Ma +/-2 (Jinnah et al., 2009), therefore the unit must be younger than this.

Jinnah et al. (2009) estimate the base of the unit at ~80.4-80.6Ma (here shown as 80.5). This is based on an extrapolation of average sedimentation rate which was calculated based on the thickness of sediment between the 80.1Ma ash date in the middle Wahweap, and the 75.96Ma ash date in the lower Kaiparowits (original unrecalibrated dates).

Jinnah et al., (2009) acknowledge that this sedimentation rate might be unreliable given that there is likely a reasonable hiatus between the Wahweap and Kaiparowits depositional sequences (e.g. Lawton et al., 2003).

Albright and Titus (2016) analysed the magnetostratigraphy of the Straight Cliffs and Wahweap fms. They showed the DripTank Mbr and upper part of the underlying John Henry Mbr as normal polarity, occurring at the top of C34n, with the overlying lower Mbr of the Wahweap Fm as reversed polarity, belonging to C33r. Albright & Titus illustrate the hiatus between the Drip Tank and lower Mbr as of imprecisely known duration.

----

### 80.6 ± 0.15

**Jinnah et al. (2009); Roberts et al. (2013)**

Bentonite CF05-B

~40m above base of Wahweap Fm (Jinnah et al., 2009)

50m above base of Wahweap Fm (Roberts et al., 2013)

80.1 Ma +/- 0.3 (sanidine; Jinnah et al., 2009)

80.1 Ma +/- 0.15 (Roberts et al., 2013; slight difference in error cited, see below)

80.6 Ma (Jinnah, 2013; no error given, but should be the same)

**80.63** Ma +/- 0.15 (Roberts et al., 2013; recalibrated, see below)

**Standard**

Jinnah et al., (2009) use the FCT age of 28.02 Ma (Renne et al., 1998). This is recalibrated to the Kuiper et al. (2008) standard of 28.201 by Jinnah et al., (2013), and Roberts et al. (2013).

**Inconsistencies**

Although Jinnah et al. (2009) state that the dated bentonite occurs in the Middle Mbr, ~40m from the base of the formation, in their generalised section it is shown occurring ~62m above the basal contact, and in their JVP abstract (Jinnah et al., 2007) the horizon is cited as being 54m above the base. Jinnah (2013) do not give specific reference to the stratigraphic position of CF05-B but show it occurring ~20 m above the base of the middle member, ~55 m above the base of the Wahweap Fm.. However, from their description of the relative position of the SS07B Bentonite (~10 m above CF05-B, and at 60 m above the base), it is assumed here that CF05-B is 50m above the base of the Wahweap, which matches the position given by Roberts et al. (2013).

There are some inconsistencies with this reported date. Jinnah et al. (2009) report the date as 80.1 Ma +/- 0.3 (1σ). However, Roberts et al. (2013) cite the Jinnah et al. (2009) report, but give the date as 80.1 +/- 0.15, also 1σ (it is noted here that if 0.15 was 1σ, 0.3 would be 2σ, so perhaps this is the source of error). The origin of this error is not clear. The recalibrated date given by Jinnah (2013) is 80.6 Ma, but no error is given (it should be about the same as before). However, the recalibrated date given by Roberts et al. (2013) is 80.63 Ma +/- 0.15, hence a difference of 0.03 Ma, and (again) the difference in error.

----

### STRAIGHT CLIFFS Fm

**Straight Cliffs Fm**

The Straight Cliffs Fm comprises 300-500 m of marine and nonmarine units, subdivided into the Drip Tank, John Henry, Smoky Hollow, and Tibbet Canyon Mbrs (Lawton et al., 2003).

**Age**

The Straight Cliffs Fm is Turonian through to Santonian in age (Lawton et al., 2003; Albright & Titus, 2016). Notes on the age of each member are given in individual entries.

----

### Drip Tank

**Drip Tank Mbr**

The Drip Tank Mbr of the Straight Cliffs Fm comprises up to 114 m of amalgamated channel sandstones and conglomerates (Lawton et al., 2003). The basal contact of the Drip Tank Mbr is described as either sharp and erosive, or interfingering with the underlying John Henry Mbr (Lawton et al. 2003).

Lawton et al. (2003) performed a sequence stratigraphic analysis of the uppermost Straight Cliffs, Wahweap, and Kaiparowits Fms. They show the Drip Tank Mbr of the Straight Cliffs Fm as the basal amalgamated channel unit of a depositional sequence otherwise comprising the Lower, Middle, and Upper Mbrs of the Wahweap Fm. However, based on changes in channel depth, abundance of lateral-accretion bedding, paleocurrent and sandstone composition, Lawton & Chistensen (2005, an abstract only) suggest that the sequence boundary lies at the top of the Drip Tank Mbr; i.e. that it is not part of the Wahweap sequence. Although, for example, Jinnah & Roberts (2011) fully agree with this interpretation, it is yet to be published (to my knowledge). Furthermore, it is not noted how this interpretation may affect the identity of the sharp erosive contact found at the base of the Drip Tank Mbr, nor the apparent lack therefore, of an amalgamated channel unit at the base of the overlying Wahweap depositonal sequence.

**Age**

Some additional support for the interpretation of Lawton & Christensen (2005) may come from Lawton et al., (2003) who state that the Drip Tank Member is latest Santonian in the Kaiparowits Plateau, which is closer in age to the underlying John Henry Mbr (Coniacian - Santonian) than the overlying basal units of the Wahweap Fm (lowermost Middle Campanian). Lawton et al., (2003) note that at the Henrieville Creek locality, the lower part of the Drip Tank contains palynomorphs of the middle Coniacian–latest Santonian *Proteacidites retusus* Zone (Nichols 1995, 1997).

Jinnah & Roberts (2011, p.280) state that "Lawton and Christensen (2005) place the Drip Tank sequence boundary at the top of the amalgamated sandstone unit rather than at the base. We fully agree with this assessment, and suggest herein that the Drip Tank sequence boundary is actually correlative with the eustatic , 80 Ma sequence boundary that has been recognized across the Western Interior Basin (Van Wagoner et al. 1990; Rogers 1998) and in global sea-level curves (Haq et al. 1987)." However, this would seem to be contradicted by the 80.63 Ma Ar / Ar date which occurs in the overlying Wahweap Fm, ~40-62 m above the base of the overlying Wahweap Fm (see individual entry).

Albright and Titus (2016) analysed the magnetostratigraphy of the Straight Cliffs and Wahweap fms. They showed the DripTank Mbr and upper part of the underlying John Henry Mbr as normal polarity, occurring at the top of C34n, with the overlying lower unit of the Wahweap Fm as reversed polarity, belonging to C33r. Following this, here the Drip Tank is shown as no younger than the uppermost limit of C34n (sensu Ogg, 2012).

Whichever interpretation is correct, the DripTank Mbr may represent a considerable amount of time, possibly even having a lower part more close in age to the John Henry Mbr, and an upper part related to the overlying Wahweap Fm.

----

### John Henry Mbr

**John Henry Mbr**

Up to 340 m of sandstones, mustones, and coals, dominantly marine on the southeast side of the Kaiparowits Plateau and nonmarine to the west (Eaton et al., 1999).

The base of the John Henry Member has been dated as lower Coniacian and the top as no younger than Upper Santonian based on marine molluscs (Eaton, 1991).

Albright and Titus (2016) analysed the magnetostratigraphy of the Straight Cliffs and Wahweap fms. They showed the DripTank Mbr and upper part of the underlying John Henry Mbr as normal polarity, occurring at the top of C34n (as depicted here).

The John Henry Member interfingers with basal sndstones of the overlying Drip Tank Mbr, but unconformably overlies the Smoky Hollow Member according to (Peterson, 1969). Duration of the unconformity is uncertain, however (Eaton et al., 1999).

----

### 87.29 ± 0.58

**Eaton et al. (1999); recalibration, Fowler (this article)**

euhedral biotite bed

~100 m below Drip Tank Mbr (Lawton et al., 2003)

Approximately the middle of the "upper" Mbr of the Straight Cliffs Fm (Eaton et al., 2001)

86.72 +/- 0.58 Ma (Ar/Ar, unknown mineral; Eaton et al., 1999; 2001)

**87.29 +/- 0.58** Ma (recalibration; Fowler, this article, see below)

**"upper" Straight Cliffs Fm, UT:**

Eaton (2006) notes an ash date in the "upper" mbr of the Straight Cliffs Fm exposed in Cedar Canyon, SW UT (note this is not the type area: the Kaiparowits Plateau, which is more commonly cited). The "upper" mbr is probably coeval, at least in part, with the John Henry Mbr, and is similarly underlain by the Smoky Hollow Mbr (Eaton et al., 2001), so for simplicity this radiometric date is shown here within the John Henry Mbr.

This same radiometric date is cited by Lawton et al. (2003, p.391), who state "the Drip Tank lies 100 m stratigraphically above a biotite tuff with a late Coniacian 40Ar/39Ar age (86.72 1 0.58 Ma; Eaton et al. 2001)."

Although Eaton et al. (2001) is often given as the source of the radiometric date (e.g. Lawton et al., 2003), the actual source is a GSA abstract (Eaton et al., 1999). This is important as it brings the age of the analysis closer to 1998, when Renne et al. modified the accepted age of the equivalent FCT from 27.84 to 28.02 Ma, hence important for recalibration purposes.

**Recalibration**

A legacy FCT value of 28.02 is assumed to have been used, as this should be the FCT equivalent date at the time of the original analysis (Eaton et al., 1999). However, it is possible that the previous standard age for FCT equivalent (27.84; Samson & Alexander., 1987) was used by Eaton et al., (1999), if the analysis was conducted before the publication of Renne et al. (1998), or if this newer standard was not used anyway.

Legacy dates; FCT at 28.02 (Renne et al. 1998); legacy λT at 5.543 +/- 0.010 E-10/y (Steiger & Jaeger, 1977).

86.72 +/- 0.58 Ma (Ar/Ar, unknown mineral; Eaton et al., 1999; 2001)

1st recalibration; FCT at 28.201 +/- 0.023 Ma (1σ; Kuiper et al, 2008), and λT at 5.463 E-10/y +/- 1.07 E-11/y (1σ; Min et al., 2000)

87.29 +/- 0.58 Ma (Fowler, this article)

2nd recalibration (for reference); FCT at 28.294 +/- 0.036 Ma (1σ), and λT at 5.531 E-10/y +/- 1.35 E-12/y (1σ; both Renne et al. 2011).

87.56 +/- 0.58 Ma (Fowler, this article)

----

### Smoky Hollow

**Straight Cliffs Fm, Smoky Hollow Mbr**

Interbedded nonmarine sandstones, mudstones, and coals, up to 40 m thick in the type area of the Kaiparowits Plateau, but up to 100 m further north east (Eaton, 1991).

**Eaton & Cifelli (1988):**

"The Smoky Hollow Member is nonmarine in origin and is considered to be late Turonian. Its age is constrained by the middle Turonian date for the underlying Tibbet Canyon Member and an early Coniacian age, based on marine molluscs, for the base of the overlying John Henry Member."

**Age**

Eaton (1991) states that the Smoky Hollow Mbr is Middle-Late Turonian. This is corroborated by a Middle Turonian radiometric date reported by Jinnah (2013; see individual entry) and / or Titus et al. (2013).

----

### 91.86

**Titus et al. (2013)**

91.86 +/- 0.34 Ma (U-Pb; zircon; n=5; Titus et al., 2013)

91.88 +/- 0.7 Ma (Ar / Ar; sanidine; n=20; Titus et al., 2013)

Titus et al. (2013) state that the sampled bentonite occurs 53.5 m above the contact with the Tibbet Canyon Member, 3.5 m below the base of the Calico bed (not shown here).

The Ar / Ar analysis was performed by A. Deino of the Berkeley Geochronology Lab; U-Pb thermal ionization mass spectrometry was peformed at Massachusetts Institute of Technology by S. Bowring (Titus et al., 2013)

**Standard**

Titus et al. state that the standard and decay constant pairing used for the Ar / Ar date are those of Kuiper et al. (2008).

----

### 91.9

**Jinnah (2013), revised from O'Connor et al. (2009)**

91.9 Ma (presumably Ar / Ar; no error given, Jinnah, 2013)

Jinnah (2013) notes a radiometric date of "approximately 91.9 Ma" from the Smoky Hollow Mbr of the Straight Cliffs Fm, citing the date as a revision of O'Connor et al. (2009; Western Interior meeting abstract). No error is given. It is not clear if this revision is a recalibration. No indication is given by Jinnah (2013) regarding the stratigraphic position of this radiometric date within the Smoky Hollow Mbr.

It is possible that this is the same date as the 91.86 U-Pb date presented by Titus et al. (2013). This is given its own entry in the chart.

**Standard**

Jinnah (2013) mentions the Smoky Hollow date within the context of other Ar / Ar dates that he had recalibrated to the FCT standard of Kuiper et al. (2008); This might imply that this is the standard also used in the 91.9 Ma date, which might be expected from an analysis presented in 2009. However, it is also possible that this refers to the U-Pb date presented by Titus (2013).

----

### Tibbett Canyon

**Tibbet Canyon Mbr**

Up to 190 m of sandstones (~56 m in type area) representing regressive marine and brackish deposition (Eaton, 1991; Eaton et al., 2001).

**Age**

Eaton et al. (2001) state that the upper part of the Tibbet Canyon Mbr is Middle Turonian, based on the presence of the molluscs Inoceramus cuvieri and *Collignoniceras woollgari* (92.90 to 92.08 Ma; Ogg & Hinnov, 2012). This is consistent with a Middle Turonian radiometric age from the overlying Smoky Hollow Mbr (Jinnah et al., 2013; see individual entry).

Eaton & Cifelli (1988) and Eaton (1991) state that the Tibbet Canyon Member contains abundant remains of the middle Turonian inoceramid bivalve *Inoceramus howelli*, the range of whichi spans ammonite zones from *Prionocyclus hyatti* to *Scaphites warreni* (91.60 - 90.65 Ma: Kauffman et al, 1993; Ogg & Hinnov, 2012).

----

# NEW MEXICO

## San Juan Basin

### OJO ALAMO Fm

**Ojo Alamo Fm**

The Ojo Alamo Fm comprises nonmarine sandstones and mudstones (Bauer, 1916). Thickness is strongly variable, generally based on the thickness of the Kimbeto Mbr which can vary between 10 - 82 m thick, whereas the Naashoibito Mbr is 10 - 25 m in thickness (Powell, 1973). Both units are heavily dominated by channel sandstones, and it can be difficult to distinguish between them.

**Age**

Age of the Naashoibito Mbr is controversial and discussed in the individual entry.

Lucas et al. (2009) show an unconformity between the Kimbeto and Naashoibito Mbrs, with the Kimbeto Mbr occurring entirely within the Paleogene, occupying the C29r - C29n boundary. This is what is shown here.

----

### Naashoibito

**Naashoibito Mbr**

The Naashoibito Mbr of the Ojo Alamo Fm comprises ~10-25 m of nonmarine sandstones and mudstones (Bauer, 1916). The unit has a fairly complex nomenclatural history, but is now generally agreed to be a member of the Ojo Alamo Fm (sensu Bauer, 1916).

**Age**

Age of the Naashoibito is not well understood and based largely on biostratigraphy with debated magnetostratigraphy.

Remains of the sauropod dinosaur *Alamosaurus* are common in the Naashoibito, and *Alamosaurus* is of relatively restricted geographic and stratigraphic range such that it has been used as a biostratigraphic indicator of the Lancian LVA or uppermost Maastrichtian (e.g. Lehman, 2001; Williamson & Weil, 2008). However, remains attributed to *Alamosaurus* from the Javelina Fm of Big Bend TX, have been dated at 69.0 +/- 0.9 Ma (Lehman et al., 2006; see individual entry), which falls within the lowermost upper Maastrichtian, or the Edmontonian LVA.

The Naashoibito Mbr is either completely reversed in polarity, or may possibly contain a short normal polarity interval (Lucas et al., 2009). This is sometimes asserted to correlate with C29r (e.g. Cifelli et al., 2004). However, it is also possible that this reversed zone may pertain to C30r (which is extremely short), or C31r, which would be consistent with the radiometric date reported from the [possibly correlative Javelina Fm TX, and a tentative U-Pb date (see comment below).

Ceratopsid dinosaur remains recovered from the Naashoibito Mbr include a nasal horn attributed to *Ojoceratops* (Sullivan & Lucas, 2010). Nasal horns of Maastrichtian ceratopsids have been shown to be biostratigraphically informative within the Hell Creek Fm of Montana, and regional equivalents (Scannella et al., 2014). A small nasal horn would not be expected within C29r as this time zone exclusively yields *Triceratops* *prorsus*, which has a much larger nasal horn (Scannella et al., 2014). A small nasal horn is observed in ceratopsids which occur stratigraphically lower than the C29r zone, and are attributed to *Triceratops* *horridus*, or *Triceratops* sp. (Scannella et al., 2014). Hence the small nasal horn of the *Ojoceratops* specimen suggests that either the Naashoibito Mbr bears a different lineage of ceratopsid dinosaurs, or that it is not correlative with the uppermost Maastrichtian sediments of the Hell Creek Fm, and equivalents.

**Potential detrital radiometric dates**

Discovery of “reworked volcanic detritus” within Naashoibito Member sandstones of the Kirtland Formation (Fassett pers. comm. to Lucas 10/2002) could potentially end dispute concerning the age of the unit. Preliminary U-Pb analysis of extracted zircon crystals (conducted by J. D. Obradovich: USGS, Denver, CO) retrieved a 70 Ma age for the horizon.

This is contrasted with a preliminary detrital Ar / Ar date reported recently in two meetings abstracts. Mason et al. (2013a, b) took three samples from different horizons within the dinosaur-bearing Naashoibito Mbr, recovering a population of detrital sanidines with variable ages, with the youngest group from one sample giving an age of either 66.5 +/- 0.2 Ma (Mason et al., 2013a; NMG Apr 2013), or 67.0 +/- 0.1 (Mason et al., 2013b; GSA Rocky Mountain Section, May 2013). Both Mason et al. (2013a) and (2013b) report that "all Ar/Ar data are at 1σ, relative to a 40K total decay constant of 5.543-10/a [Steiger & Jaeger, 1977] and Fish Canyon sanidine at 28.294 Ma [Renne et al., 2011]", which is a non-standard pairing. I have recalibrated these dates using the standard A/Ar pairings:

Legacy dates; FCT at 28.294 +/- 0.294 Ma (1σ; Renne et al., 2011).); legacy λT at 5.543 +/- 0.010 E-10/y (Steiger & Jaeger, 1977).

66.5 +/- 0.2 Ma (1σ); (Ar/Ar, detrital sanidine; Mason et al., 2013a)

67.0 +/- 0.1 Ma (1σ); (Ar/Ar, detrital sanidine; Mason et al., 2013b)

1st recalibration; FCT at 28.201 +/- 0.023 Ma (1σ; Kuiper et al., 2008), and λT at 5.463 E-10/y +/- 1.07 E-11/y (1σ; Min et al., 2000)

66.294 +/- 0.199 Ma (1σ); (Fowler, this article)

66.792 +/- 0.100 Ma (1σ); (Fowler, this article)

2nd recalibration (for reference); FCT at 28.294 +/- 0.294 Ma (1σ), and λT at 5.531 E-10/y +/- 1.35 E-12/y (1σ; both Renne et al., 2011).

66.502 +/- 0.200 Ma (1σ); (Fowler, this article)

67.002 +/- 0.100 Ma (1σ); (Fowler, this article)

Mason et al. (2013a, b) state that variations in age populations between samples could indicate unconformities within the Naashoibito itself. The discrepancy between the reported ages is not yet resolved, and awaits full publication of the new analyses.

I have taken a relatively conservative view and plotted the Naashoibito as correlated with the 69 Ma U-Pb date from Texas. Based on comparison of the nasal horn morphology of the dinosaur *Ojoceratops* (Sullivan & Lucas, 2010) with that of *Triceratops* (Scannella et al., 2014), I expect that the Naashoibito is probably only slightly older than the Hell Creek Fm of Montana (~67-66 Ma), probably around 68 Ma.

----

### 70

**Reworked volcanic detritus**

Discovery of “reworked volcanic detritus” within Naashoibito Member sandstones of the Kirtland Formation (Fassett pers. comm. to Lucas 10/2002) could potentially end dispute concerning the age of the unit. Preliminary U-Pb analysis of extracted zircon crystals (conducted by J. D. Obradovich: USGS, Denver, CO) retrieved a 70 Ma age for the horizon.

However, see main entry for discussion of this date with comparison to more recent Ar / Ar analysis.

----

### KIRTLAND Fm

**Kirtland Fm**

Bauer (1916) describes the Kirtland Shale (Fm) as comprising a lower shale (now recognized as the Hunter Wash Mbr) 271 feet (82 m) thick; the Farmington sandstone Mbr, 0-455 ft (0-139 m) thick, and an upper shale (now recognized as the Denazin Mbr) 40 - 110 ft (12 - 33 m) thick

**Upper contact**

Here I follow the original definition of the Kirtland Fm (Bauer, 1916) which defines the upper contact as between the Denazin Mbr ("upper shale" of his usage) and the Lower Conglomerate of the Naashoibito Mbr of the overlying Ojo Alamo Fm.

**Lower contact**

Bauer (1916; and later Bauer & Reeside, 1921; Reeside, 1924) considered the Fruitland-Kirtland boundary as being gradational, but showed the boundary as occurring at the top of a moderately thick sandstone that occurs above the last persistent coal. This was mostly followed by researchers until Fassett & Hinds (1971) who redefined the boundary as occurring at the top of the last persistent coal. This definition was later considered ambiguous by Hunt and Lucas (1992), but is important as it was used for many of the USGS maps from the late 1970's, which remain in use today; and also because thin stringer coals in the overlying Hunter Wash Mbr of the Kirtland Fm have sometimes been misidentified as the Fruitland-Kirtland boundary, thus overestimating the thickness of the Fruitland Fm (e.g. Lindsay et al., 1981). In a GSA abstract, Hunt (1986) was the first to refer to the uppermost Fruitland Fm boundary sandstone of Bauer (1916) as the "Bisti member", reassigning it as the lowermost member of the Kirtland Fm (formalized to Bisti Member by Hunt & Lucas, 1992, and subsequently changed to "Bisti Bed" by Lucas et al., 2006). Hunt & Lucas (2003) subdivided the Fruitland Fm into a lower Neh-nah-ne-zad Mbr and an upper Fossil Forest Mbr. I follow this latest revision of the stratigraphic nomenclature.

**Age**.

The Kirtland Fm is upper Campanian, based on radiometric dates, and magnetostratigraphy of limited use.

Lucas et al. (2009) show that the Fruitland and Kirtland Fms are mostly of normal polarity, assigned to C33n. However, the upper part of the Denazin Mbr is shown to be reversed polarity, assigned to C32r (Lucas e al., 2009). These assignments are consistent with the radiometric dates recovered by Fassett & Steiner (1997).

**Radiometric dates**

Brookins & Rigby (1987) reported a number of K / Ar dates from the Kirtland Formation. When plotted in Google Earth, these ashes occur within exposures of the Hunter Wash Member, although one (sample JKR-93) plots very close to the Fruitland Formation boundary. This is a little odd as Brookins and Rigby refer to JKR-93 as the middle ash, and it yielded a younger age than the stratigraphically higher ashes:

JKR-93, upper middle ash: sanidine concentrate: 69.8 ± 2.5 Ma.

JKR-54, highest ash: sanidine concentrates: 72.4 ± 3.1 to 74.4 ± 2.6 Ma; biotite concentrates: 73.2 ± 2.7 to 76.1 ± 2.8 Ma.

JKR-62, lowest ash: sanidine concentrate: 75.0 ± 2.7 Ma.

More recently, Fassett & Steiner (1997) published five Ar / Ar radiometric dates from the Fruitland & Kirtland Fms. These dates are all based on sanidine crystals and are more reliable, accurate, and precise than the dates of Brookins and Rigby (1987). It is notable that one of Brookins and Rigby's ashes (JKR-62) plots in almost the exact same geographic location as Fassett & Steiner's Ash 2, with a similar retrieved date. See individual entries for the recalibrated Ar / Ar dates from Fassett & Steiner (1997).

----

### 73.49 ± 0.25

**Fassett & Steiner (1997); recalibration, Fowler (this article)**

Ash J

4.9 m below top of the Denazin Mbr

73.04 +/- 0.25 Ma (Ar/Ar, sanidine, single crystal; Fassett & Steiner, 1997)

*72.66 +/- 0.25 Ma (*miscalculated recalibration of Roberts et al., 2013; see below)

**73.49 +/- 0.25** Ma (95% confidence interval); (recalibration; Fowler, this article, see below)

**Stratigraphy**

21cm thick layer, 384m above base of Fruitland Fm, 4.9m below base of overlying Ojo Alamo sandstone.

"overlain by a chocolate brown mudstone very near the the top of the Farmington Mbr of the Kirtland Shale at 36 degrees 21.86'N 108 degrees 07.88'W" (Fassett & Steiner, 1997).

"Near the top of the Kirtland Formation, in the highest part of the De-na-zin Member, lie two other ashes, Ash H, dated at 73.37 ± 0.28 Ma and Ash J, dated at 73.04 ± 0.25 Ma." (Sullivan & Lucas, 2006).

**Standard**

Fassett & Steiner (1997) do not explicitly state the standard used, but note that the samples were processed by Obradovich using "methodology described in Obradovich, 1993", which states ""Sanidine from the Oligocene Taylor Creek Rhyolite [TCR] (Duffield and Dalrymple, 1990) was used as the monitor mineral... All ages were calculated by normalizing the age of the TCR to a value of 520.4 Ma for the McClure Mountain hornblende monitor MMhb-1". Hicks et al. (2002) note that Obradovich had been exclusively using the TCR at 28.32 Ma as his standard since 1990, and had independently arrived at a normalised age of of 28.03 Ma for the FCT, which (as they note) is very close to the accepted FCT 28.02 Ma of Renne et al. (1998). Decay constant λT is not given explicitly, but from the values of λβ and λε given in the chart (p.243) , can be confirmed as 5.543 +/- 0.010 E-10/y (Steiger & Jaeger, 1977).

***Erroneous recalibration (Roberts et al., 2013)**

Roberts et al. (2013) recalibrate the dates from Fassett & Steiner (1997), however they input the incorrect original (legacy) decay constant (λ) and standard, producing recalibrated dates that are incorrect by nearly a million years (see below). First, the legacy λ used by Roberts et al. (2013) is 4.962E-10/y, which was presumably copied from the bottom of the chart on p243 of Fassett & Steiner (1997), where it is clearly referred to as the value of λβ (ie. the probability of β- decay of 40K to 40Ca), and which is printed below the value λε (0.581 E-10/y; probability of electron capture or β+ of 40Kto 40Ar). In this case, the correct λ value to use for recalibration is 5.543 E-10/y (Steiger & Jaeger, 1977), which is the total (λT) of λβ plus λε. Second, Roberts et al. (2013) correctly state that the legacy standard used by Fassett & Steiner (1997) for fluence monitoring was the TCR at 28.32 Ma; however Roberts et al. (2013) then use this number directly for their recalibration to the new FCT standard (28.201; Kuiper et al., 2008). This is incorrect as recalculation must use the same standard mineral (e.g. FCT) for both legacy and recalibrated dates. For the recalculation to be correct, the legacy standard must therefore be the value of FCT that was equivalent to the TCR at 28.32 at the time of the 1997 analysis, which is either FCT = 27.84 Ma or ~28.03 (see below), both of which produce recalibrated ages ~1 million years older than the dates presented by Roberts et al. (2013).

The recalibrated dates of Roberts et al. (2013) were replicated (therefore confirmed) by rerunning the legacy values through the recalibration spreadsheet provided by the Earth-Time institute.

**Recalibration**

A legacy FCT value of 28.03 was used, as this was given by Hicks et al (2002) as equivalent of the TCR at 28.32 (see note on TCR standard). The unusual standard is due to the particular methods of Obradovich, who ran the analysis. For a discussion of the issues surrounding recalibration and comparison of the TCR and FCT standards in Obradovich analyses from the 1990's through to ~2002, see the Ar-Ar notes elsewhere on this chart.

Legacy dates; FCT at 28.03 (Hicks et al., 2002; see above); legacy λT at 5.543 +/- 0.010 E-10/y (Steiger & Jaeger, 1977).

73.04 +/- 0.25 Ma (95% confidence interval); (Ar/Ar, sanidine, single crystal; Fassett & Steiner, 1997)

1st recalibration; FCT at 28.201 +/- 0.023 Ma (1σ; Kuiper et al, 2008), and λT at 5.463 E-10/y +/- 1.07 E-11/y (1σ; Min et al., 2000)

73.49 +/- 0.25 Ma (95% confidence interval); (Fowler, this article)

2nd recalibration (for reference); FCT at 28.294 +/- 0.036 Ma (1σ), and λT at 5.531 E-10/y +/- 1.35 E-12/y (1σ; both Renne et al. 2011).

73.72 +/- 0.25 Ma (95% confidence interval); (Fowler, this article)

----

### 73.83 ± 0.18

**Fassett & Steiner (1997):**

Ash H

<5 m above base of the Denazin Mbr (Sullivan et al., 2005; see below)

73.37 +/- 0.18 Ma (95% confidence interval); (Ar/Ar, sanidine, 2 samples: both single crystal; Fassett & Steiner, 1997)

*72.698 +/- 0.18 Ma (*miscalculated recalibration of Roberts et al., 2013; see below)

**73.83 +/- 0.18** Ma (95% confidence interval); (recalibration; Fowler, this article, see below)

Original analysis conducted by JD Obradovich, USGS, CO, USA

**Stratigraphy**

20cm thick layer, 359m above base of Fruitland Fm, "collected from the base of a chocolate brown mudstone in the Farmington Sandstone Mbr of the Kirtland Shale 36 degrees 21.63'N 108 degrees 36'W" (Fassett & Steiner, 1997). When plotted, these coordinates do not plot within the Denazin Mbr, indeed they plot far west of any Fruitland-Kirtland exposures, suggesting that the "36'W" part requires two more figures, either as (36.xx'W) or (xx.36'W). Given that there are no exposures of the Denazin Mbr anywhere along strike of 36'-37' W, then it is suspected that the missing data be (xx.36 W).

Sullivan et al. (2005b) state that "Ash H is near the base of the De-na-zin Member, less than 5 m above its contact with the underlying Farmington Member", but do not give a precise location. However, this is itself slightly contradicted by Sullivan & Lucas (2006) who state: "Near the top of the Kirtland Formation, in the highest part of the De-na-zin Member, lie two other ashes, Ash H, dated at 73.37 ± 0.28 Ma and Ash J, dated at 73.04 ± 0.25 Ma." (Sullivan & Lucas, 2006). Also note that the error for ash H is slightly misquoted here.

**Standard**

Fassett & Steiner (1997) do not explicitly state the standard used, but note that the samples were processed by Obradovich using "methodology described in Obradovich, 1993", which states ""Sanidine from the Oligocene Taylor Creek Rhyolite [TCR] (Duffield and Dalrymple, 1990) was used as the monitor mineral... All ages were calculated by normalizing the age of the TCR to a value of 520.4 Ma for the McClure Mountain hornblende monitor MMhb-1". Hicks et al. (2002) note that Obradovich had been exclusively using the TCR at 28.32 Ma as his standard since 1990, and had independently arrived at a normalised age of of 28.03 Ma for the FCT, which (as they note) is very close to the accepted FCT 28.02 Ma of Renne et al. (1998). Decay constant λT is not given explicitly, but from the values of λβ and λε given in the chart (p.243) , can be confirmed as 5.543 +/- 0.010 E-10/y (Steiger & Jaeger, 1977).

***Erroneous recalibration (Roberts et al., 2013)**

Roberts et al. (2013) recalibrate the dates from Fassett & Steiner (1997), however they input the incorrect original (legacy) decay constant (λ) and standard, producing recalibrated dates that are incorrect by nearly a million years (see below). First, the legacy λ used by Roberts et al. (2013) is 4.962E-10/y, which was presumably copied from the bottom of the chart on p243 of Fassett & Steiner (1997), where it is clearly referred to as the value of λβ (ie. the probability of β- decay of 40K to 40Ca), and which is printed below the value λε (0.581 E-10/y; probability of electron capture or β+ of 40Kto 40Ar). In this case, the correct λ value to use for recalibration is 5.543 E-10/y (Steiger & Jaeger, 1977), which is the total (λT) of λβ plus λε. Second, Roberts et al. (2013) correctly state that the legacy standard used by Fassett & Steiner (1997) for fluence monitoring was the TCR at 28.32 Ma; however Roberts et al. (2013) then use this number directly for their recalibration to the new FCT standard (28.201; Kuiper et al., 2008). This is incorrect as recalculation must use the same standard mineral (e.g. FCT) for both legacy and recalibrated dates. For the recalculation to be correct, the legacy standard must therefore be the value of FCT that was equivalent to the TCR at 28.32 at the time of the 1997 analysis, which is either FCT = 27.84 Ma or ~28.03 (see below), both of which produce recalibrated ages ~1 million years older than the dates presented by Roberts et al. (2013).

The recalibrated dates of Roberts et al. (2013) were replicated (therefore confirmed) by rerunning the legacy values through the recalibration spreadsheet provided by the Earth-Time institute.

**Recalibration**

A legacy FCT value of 28.03 was used, as this was given by Hicks et al (2002) as equivalent of the TCR at 28.32 (see note on TCR standard). The unusual standard is due to the particular methods of Obradovich, who ran the analysis. For a discussion of the issues surrounding recalibration and comparison of the TCR and FCT standards in Obradovich analyses from the 1990's through to ~2002, see the Ar-Ar notes elsewhere on this chart.

Legacy dates; FCT at 28.03 (Hicks et al., 2002; see above); legacy λT at 5.543 +/- 0.010 E-10/y (Steiger & Jaeger, 1977).

73.37 +/- 0.18 Ma (95% confidence interval); (Ar/Ar, sanidine, 2 samples: both single crystal; Fassett & Steiner, 1997)

1st recalibration; FCT at 28.201 +/- 0.023 Ma (1σ; Kuiper et al, 2008), and λT at 5.463 E-10/y +/- 1.07 E-11/y (1σ; Min et al., 2000)

73.83 +/- 0.18 Ma (95% confidence interval); (Fowler, this article)

2nd recalibration (for reference); FCT at 28.294 +/- 0.036 Ma (1σ), and λT at 5.531 E-10/y +/- 1.35 E-12/y (1σ; both Renne et al. 2011).

74.05 +/- 0.18 Ma (95% confidence interval); (Fowler, this article).

----

### 74.57 ± 0.62

**Fassett & Steiner (1997); recalibration, this article.**

Ash 4

74.11 Ma +/- 0.62 (95% confidence interval); (Ar/Ar, sanidine, multiple crystals; Fassett & Steiner, 1997)

*73.72 Ma +/- 0.62 (*miscalculated recalibration of Roberts et al., 2013; see below)

**74.57 Ma +/- 0.62** (95% confidence interval); (recalibration; Fowler, this article; see below)

Analysis conducted by JD Obradovich, USGS, CO, USA

34cm thick layer, 181m from base of Fruitland Fm; "...top of a coal bed at the top of the Fruitland Fm", Hunter Wash (Fassett & Steiner, 1997).

Sullivan and Lucas (2006) revised the strarigraphic position of ash 4, stating that "Ash 4 is stratigraphically higher [than Ash 2] in the south-facing cut-bank of Hunter Wash at UTM 12 S, 754040E, 4022208N.". This places ash 4 in the middle of the Hunter Wash Mbr of the Kirtland Fm.

**Standard**

Fassett & Steiner (1997) do not explicitly state the standard used, but note that the samples were processed by Obradovich using "methodology described in Obradovich, 1993", which states ""Sanidine from the Oligocene Taylor Creek Rhyolite [TCR] (Duffield and Dalrymple, 1990) was used as the monitor mineral... All ages were calculated by normalizing the age of the TCR to a value of 520.4 Ma for the McClure Mountain hornblende monitor MMhb-1". Hicks et al. (2002) note that Obradovich had been exclusively using the TCR at 28.32 Ma as his standard since 1990, and had independently arrived at a normalised age of of 28.03 Ma for the FCT, which (as they note) is very close to the accepted FCT 28.02 Ma of Renne et al. (1998). Decay constant λT is not given explicitly, but from the values of λβ and λε given in the chart (p.243) , can be confirmed as 5.543 +/- 0.010 E-10/y (Steiger & Jaeger, 1977).

***Erroneous recalibration (Roberts et al., 2013)**

Roberts et al. (2013) recalibrate the dates from Fassett & Steiner (1997), however they input the incorrect original (legacy) decay constant (λ) and standard, producing recalibrated dates that are incorrect by nearly a million years (see below). First, the legacy λ used by Roberts et al. (2013) is 4.962E-10/y, which was presumably copied from the bottom of the chart on p243 of Fassett & Steiner (1997), where it is clearly referred to as the value of λβ (ie. the probability of β- decay of 40K to 40Ca), and which is printed below the value λε (0.581 E-10/y; probability of electron capture or β+ of 40Kto 40Ar). In this case, the correct λ value to use for recalibration is 5.543 E-10/y (Steiger & Jaeger, 1977), which is the total (λT) of λβ plus λε. Second, Roberts et al. (2013) correctly state that the legacy standard used by Fassett & Steiner (1997) for fluence monitoring was the TCR at 28.32 Ma; however Roberts et al. (2013) then use this number directly for their recalibration to the new FCT standard (28.201; Kuiper et al., 2008). This is incorrect as recalculation must use the same standard mineral (e.g. FCT) for both legacy and recalibrated dates. For the recalculation to be correct, the legacy standard must therefore be the value of FCT that was equivalent to the TCR at 28.32 at the time of the 1997 analysis, which is either FCT = 27.84 Ma or ~28.03 (see below), both of which produce recalibrated ages ~1 million years older than the dates presented by Roberts et al. (2013).

The recalibrated dates of Roberts et al. (2013) were replicated (therefore confirmed) by rerunning the legacy values through the recalibration spreadsheet provided by the Earth-Time institute.

**Recalibration**

A legacy FCT value of 28.03 was used, as this was given by Hicks et al (2002) as equivalent of the TCR at 28.32 (see note on TCR standard). The unusual standard is due to the particular methods of Obradovich, who ran the analysis. For a discussion of the issues surrounding recalibration and comparison of the TCR and FCT standards in Obradovich analyses from the 1990's through to ~2002, see the Ar-Ar notes elsewhere on this chart.

Legacy dates; FCT at 28.03 (Hicks et al., 2002; see above); legacy λT at 5.543 +/- 0.010 E-10/y (Steiger & Jaeger, 1977).

74.11 +/- 0.62 Ma (95% confidence interval); (Ar/Ar, sanidine, multiple crystals; Fassett & Steiner, 1997)

1st recalibration; FCT at 28.201 +/- 0.023 Ma (1σ; Kuiper et al, 2008), and λT at 5.463 E-10/y +/- 1.07 E-11/y (1σ; Min et al., 2000)

74.57 +/- 0.62 Ma (95% confidence interval); (Fowler, this article)

2nd recalibration (for reference); FCT at 28.294 +/- 0.036 Ma (1σ), and λT at 5.531 E-10/y +/- 1.35 E-12/y (1σ; both Renne et al. 2011).

74.80 +/- 0.63 Ma (95% confidence interval); (Fowler, this article)

----

### 75.02 ± 0.13

**Fassett & Steiner (1997); recalibrated, this article.**

Ash 2

Lower-middle part of the Hunter Wash Mbr, Kirtland Fm (see below)

74.56 +/- 0.13 Ma (95% confidence interval); (Ar/Ar, sanidine, 3 samples: 2 x single crystal, 1 x "multiple crystals"; Fassett & Steiner, 1997)

*74.17 Ma +/- 0.13 (*miscalculated recalibration of Roberts et al., 2013; see below)

**75.02 Ma +/- 0.13** (recalibration, this article, see below)

**Locality & stratigraphy correction:**

Fassett & Steiner (1997) state that Ash 2 was an 18cm thick layer, 136m from base of Fruitland Fm in the "Upper part of the stratigraphically lowest Fruitland Fm", "Hunter Wash: 36 degrees 17.74' N , 108 degrees 13.46' W". This was corrected by Lucas et al. (2006) who state "Ash 2 [...] is in the lower part of the Hunter Wash Member of the Kirtland Formation, not in the Fruitland Formation. The dated sample came from a thin layer of clayey ash in a coal bed in the north-facing cut bank of Hunter Wash at UTM zone 12, 749213E, 4020100N, NAD 27"; and by Sullivan and Lucas (2006) who state. "Ash 2 is in the north facing cut-bank of the wash at UTM 12 S, 729213E, 4020100N (NAD 27), in the middle part of the Hunter Wash Member.". From the stratigraphic chart published with Sullivan & Lucas (2006), Ash 2 derives from a little less than halfway through the Hunter Wash Mbr, in the lower part of the silt/mud unit that overlies the Bisti bed (which forms the lowermost bed of the Mbr). This accounts for the slight difference in stratigraphic position suggested by Lucas et al (2006) and Sullivan & Lucas (2006). Sullivan & Lucas (2006) also cite a slightly incorrect age for Ash 2: they cite 74.55 +/- 0.29, compared to 74.55 +/- 0.13 of Fassett & Steiner (1997) and Lucas et al (2006). I have plotted the GPS coordinates of the ash, and it occurs low in the Hunter Wash Mbr, probably just above the Bisti Bed.

**Standard**

Fassett & Steiner (1997) do not explicitly state the standard used, but note that the samples were processed by Obradovich using "methodology described in Obradovich, 1993", which states ""Sanidine from the Oligocene Taylor Creek Rhyolite [TCR] (Duffield and Dalrymple, 1990) was used as the monitor mineral... All ages were calculated by normalizing the age of the TCR to a value of 520.4 Ma for the McClure Mountain hornblende monitor MMhb-1". Hicks et al. (2002) note that Obradovich had been exclusively using the TCR at 28.32 Ma as his standard since 1990, and had independently arrived at a normalised age of of 28.03 Ma for the FCT, which (as they note) is very close to the accepted FCT 28.02 Ma of Renne et al. (1998). Decay constant λT is not given explicitly, but from the values of λβ and λε given in the chart (p.243) , can be confirmed as 5.543 +/- 0.010 E-10/y (Steiger & Jaeger, 1977).

***Erroneous recalibration (Roberts et al., 2013)**

Roberts et al. (2013) recalibrate the dates from Fassett & Steiner (1997), however they input the incorrect original (legacy) decay constant (λ) and standard, producing recalibrated dates that are incorrect by nearly a million years (see below). First, the legacy λ used by Roberts et al. (2013) is 4.962E-10/y, which was presumably copied from the bottom of the chart on p243 of Fassett & Steiner (1997), where it is clearly referred to as the value of λβ (ie. the probability of β- decay of 40K to 40Ca), and which is printed below the value λε (0.581 E-10/y; probability of electron capture or β+ of 40Kto 40Ar). In this case, the correct λ value to use for recalibration is 5.543 E-10/y (Steiger & Jaeger, 1977), which is the total (λT) of λβ plus λε. Second, Roberts et al. (2013) correctly state that the legacy standard used by Fassett & Steiner (1997) for fluence monitoring was the TCR at 28.32 Ma; however Roberts et al. (2013) then use this number directly for their recalibration to the new FCT standard (28.201; Kuiper et al., 2008). This is incorrect as recalculation must use the same standard mineral (e.g. FCT) for both legacy and recalibrated dates. For the recalculation to be correct, the legacy standard must therefore be the value of FCT that was equivalent to the TCR at 28.32 at the time of the 1997 analysis, which is either FCT = 27.84 Ma or ~28.03 (see below), both of which produce recalibrated ages ~1 million years older than the dates presented by Roberts et al. (2013).

The recalibrated dates of Roberts et al. (2013) were replicated (therefore confirmed) by rerunning the legacy values through the recalibration spreadsheet provided by the Earth-Time institute.

**Recalibration**

A legacy FCT value of 28.03 was used, as this was given by Hicks et al (2002) as equivalent of the TCR at 28.32 (see note on TCR standard). The unusual standard is due to the particular methods of Obradovich, who ran the analysis. For a discussion of the issues surrounding recalibration and comparison of the TCR and FCT standards in Obradovich analyses from the 1990's through to ~2002, see the Ar-Ar notes elsewhere on this chart.

Legacy dates; FCT at 28.03 (Hicks et al., 2002; see above); legacy λT at 5.543 +/- 0.010 E-10/y (Steiger & Jaeger, 1977).

74.56 +/- 0.13 Ma (95% confidence interval); (Ar/Ar, sanidine, 3 samples: 2 x single crystal, 1 x multiple crystals; Fassett & Steiner, 1997)

1st recalibration; FCT at 28.201 +/- 0.023 Ma (1σ; Kuiper et al, 2008), and λT at 5.463 E-10/y +/- 1.07 E-11/y (1σ; Min et al., 2000)

75.02 +/- 0.13 Ma (95% confidence interval); (Fowler, this article)

2nd recalibration (for reference); FCT at 28.294 +/- 0.036 Ma (1σ), and λT at 5.531 E-10/y +/- 1.35 E-12/y (1σ; both Renne et al. 2011).

75.26 +/- 0.13 Ma (95% confidence interval); (Fowler, this article)

**Correlation with old K-Ar date of Brookins & Rigby (1987)**

When plotted on Google Earth, Ash JKR-62 of Brookins & Rigby (1987) plots almost on top of the locality of Ash 2 (Fassett & Steiner, 1997; Sullivan & Lucas, 2006), suggesting that they may correspond to the same sampled ash bed. The date from Brookins and Rigby (1987) of 75.0 +/- 2.7 compares well with the recalibrated date of 75.02 +/- 0.13, although the error in the Brookins & Rigby date is considerable higher, and would encompass the full duration of the Fruitland and Kirtland Fms.

----

### FRUITLAND Fm

**Fruitland Fm**

The Fruitland Fm comprises ~68 m of nonmarine sandstones, mudstones, and thick coals, divided into the Fossil Forest and Neh-nah-ne-zad Mbrs (Lucas et al., 2006).

**Upper contact**

Bauer (1916; and later Bauer & Reeside, 1921; Reeside, 1924) considered the Fruitland-Kirtland boundary as being gradational, but showed the boundary as occurring at the top of a moderately thick sandstone that occurs above the last persistent coal. This was mostly followed by researchers until Fassett & Hinds (1971) who redefined the boundary as occurring at the top of the last persistent coal. This definition was later considered ambiguous by Hunt and Lucas (1992), but is important as it was used for many of the USGS maps from the late 1970's, which remain in use today; and also because thin stringer coals in the overlying Hunter Wash Mbr of the Kirtland Fm have sometimes been misidentified as the Fruitland-Kirtland boundary, thus overestimating the thickness of the Fruitland Fm (e.g. Lindsay et al., 1981). In a GSA abstract, Hunt (1986) was the first to refer to the uppermost Fruitland Fm boundary sandstone of Bauer (1916) as the "Bisti member", reassigning it as the lowermost member of the Kirtland Fm (formalized to Bisti Member by Hunt & Lucas, 1992, and subsequently changed to "Bisti Bed" by Lucas et al., 2006). I follow this latest revision of the stratigraphic nomenclature.

**Lower contact**

The lower contact is conformable, and records a transition from the marine Pictured Cliffs Sandstone, to the non-marine Fruitland Formation. It is not controversial, but has a number of definitions depending on the facies present at the top of the Pictured Cliffs Sandstone (Lucas et al., 2006). It is defined as either: 1. the first shale or coal above a massive Pictured Cliffs Sandstone; 2. the first carbonaceous sandstone or mudrock above the highest *Ophiomoprha*-bearing sandstone of the Pictured Cliffs Sandstone; 3. in intertonguing areas, the boundary must be defined to exclude ophiomorpha-bearing sandstones from the Fruitland.

**Age**

The Fruitland Fm is well constrained by Ar / Ar dates (Fassett & Steiner, 1997). A date of 76.03 Ma was recovered from 29 m above the base of the Fruitland Fm, whereas a date of 75.02 Ma was recovered from the lower part of the overlying Hunter Wash Mbr of the Kirtland Fm (Fassett & Steiner, 1997; recalibrated, this article; see individual entries).

----

### 76.03 ± 0.41

**Fassett & Steiner (1997); recalibrated, this article.**

Ash DEP (Dog Eye Pond)

29m above base of Fruitland Fm

75.56 +/- 0.41 Ma (95% confidence interval); (Ar/Ar, sanidine, 1 sample, multiple crystals; Fassett & Steiner, 1997)

*75.16 Ma +/- 0.41 Ma (*miscalculated recalibration of Roberts et al., 2013; see below)

**76.03 Ma +/- 0.41** Ma (95% confidence interval); (recalibration, this article, see below)

Analysis conducted by JD Obradovich, USGS, CO, USA

18cm thick, 29m above base of Fruitland Fm. "Upper part of the stratigraphically lowest Fruitland Fm" Hunter Wash, NM, 36 degrees 11.35' N , 108 degrees 10.28' W

**Standard**

Fassett & Steiner (1997) do not explicitly state the standard used, but note that the samples were processed by Obradovich using "methodology described in Obradovich, 1993", which states ""Sanidine from the Oligocene Taylor Creek Rhyolite [TCR] (Duffield and Dalrymple, 1990) was used as the monitor mineral... All ages were calculated by normalizing the age of the TCR to a value of 520.4 Ma for the McClure Mountain hornblende monitor MMhb-1". Hicks et al. (2002) note that Obradovich had been exclusively using the TCR at 28.32 Ma as his standard since 1990, and had independently arrived at a normalised age of of 28.03 Ma for the FCT, which (as they note) is very close to the accepted FCT 28.02 Ma of Renne et al. (1998). Decay constant λT is not given explicitly, but from the values of λβ and λε given in the chart (p.243) , can be confirmed as 5.543 +/- 0.010 E-10/y (Steiger & Jaeger, 1977).

***Erroneous recalibration (Roberts et al., 2013)**

Roberts et al. (2013) recalibrate the dates from Fassett & Steiner (1997), however they input the incorrect original (legacy) decay constant (λ) and standard, producing recalibrated dates that are incorrect by nearly a million years (see below). First, the legacy λ used by Roberts et al. (2013) is 4.962E-10/y, which was presumably copied from the bottom of the chart on p243 of Fassett & Steiner (1997), where it is clearly referred to as the value of λβ (ie. the probability of β- decay of 40K to 40Ca), and which is printed below the value λε (0.581 E-10/y; probability of electron capture or β+ of 40Kto 40Ar). In this case, the correct λ value to use for recalibration is 5.543 E-10/y (Steiger & Jaeger, 1977), which is the total (λT) of λβ plus λε. Second, Roberts et al. (2013) correctly state that the legacy standard used by Fassett & Steiner (1997) for fluence monitoring was the TCR at 28.32 Ma; however Roberts et al. (2013) then use this number directly for their recalibration to the new FCT standard (28.201; Kuiper et al., 2008). This is incorrect as recalculation must use the same standard mineral (e.g. FCT) for both legacy and recalibrated dates. For the recalculation to be correct, the legacy standard must therefore be the value of FCT that was equivalent to the TCR at 28.32 at the time of the 1997 analysis, which is either FCT = 27.84 Ma or ~28.03 (see below), both of which produce recalibrated ages ~1 million years older than the dates presented by Roberts et al. (2013).

The recalibrated dates of Roberts et al. (2013) were replicated (therefore confirmed) by rerunning the legacy values through the recalibration spreadsheet provided by the Earth-Time institute.

**Recalibration**

A legacy FCT value of 28.03 was used, as this was given by Hicks et al (2002) as equivalent of the TCR at 28.32 (see note on TCR standard). The unusual standard is due to the particular methods of Obradovich, who ran the analysis. For a discussion of the issues surrounding recalibration and comparison of the TCR and FCT standards in Obradovich analyses from the 1990's through to ~2002, see the Ar-Ar notes elsewhere on this chart.

Legacy dates; FCT at 28.03 (Hicks et al., 2002; see above); legacy λT at 5.543 +/- 0.010 E-10/y (Steiger & Jaeger, 1977).

75.56 +/- 0.41 Ma (95% confidence interval); (Ar/Ar, sanidine, 1 sample, multiple crystals; Fassett & Steiner, 1997)

1st recalibration; FCT at 28.201 +/- 0.023 Ma (1σ; Kuiper et al, 2008), and λT at 5.463 E-10/y +/- 1.07 E-11/y (1σ; Min et al., 2000)

76.03 +/- 0.41 Ma (95% confidence interval); (Fowler, this article)

2nd recalibration (for reference); FCT at 28.294 +/- 0.036 Ma (1σ), and λT at 5.531 E-10/y +/- 1.35 E-12/y (1σ; both Renne et al. 2011).

76.27 +/- 0.41 Ma (95% confidence interval); (Fowler, this article)

----

### PICTURED CLIFFS sst

**Pictured Cliffs sst**

The Pictured Cliffs sandstone contains the ammonite *Baculites scotti* (Rowe et al., 1992). The range of *B. scotti* is 76.94 - 76.27 Ma (Ogg & Hinnov, 2012). This fits well with the ash date for the lowermost Neh-nah-ne-zad Mbr of the Fruitland Fm (76.03 Ma, Fassett & Steiner, 1997).

Here I show the Pictured Cliffs sandstone as occupying the range of the ammonite *B. scotti*.

----

### MENEFEE Fm

**Menefee Fm**

The Menefee Fm comprises ~200-250 m of alluvial sandstones, mudstones, and coals (Beaumont & Hoffman, 1992; Williamson, 1996). The Menefee is subdivided into the Cleary Coal Mbr, Allison Mbr (devoid of coals), and Upper Coal Mbr (Beaumont & Hoffman, 1992). This chart is mainly concerned with the Allison Mbr, which is up to 183 m thick and bears most of the vertebrate fossils recovered from the Menefee Fm.

**Age**

Age of the Menefee Fm is relatively poorly understood. Recent reviews of the fauna provide the best indication of age. Heckert et al. (2007) suggest that the fauna of the Allison Mbr is in the range of 80-83.5 Ma (lower Campanian). This is consistent with the regional cross section presented by Molenaar et al. (2002), which shows the Menefee Fm limited to the lower Campanian.

----

### MORENO HILL Fm

**Moreno Hill Fm**

**Age**

Here I follow Molenaar (2002) who shows the Moreno Hill Fm ranging from the middle of the middle Turonian, to the top of the upper Turonian.

----

# TEXAS

## Big Bend NP

### BLACK PEAKS Fm

**Black** **Peaks**

The Black Peaks Fm is a mudstone dominated unit in contrast to the underlying Javelina Fm which is sandstone dominated. The contact between the two formations has been debated (e.g. Schiebout et al., 1987), and the most recent works (e.g. Lehman et al., 2006) place the contact at the top of the last laterally extensive sandstone. However, this approach means that it is likely that the basalmost mudstones of the Black Peaks Fm actually represent the fine grained deposits from the last depositional cycle of the Javelina Fm. The result of this is that under the formational definition of Lehman et al. (2006), there is technically no depositional hiatus between the Javelina Fm and the overlying Black Peaks Fm (as shown here).

**Age**

Age of the Black Peaks Fm is problematic and based upon fossils and magnetostratigraphy.

The position of the boundary between Cretaceous and Paleogene rocks had been troublesome to locate. Presence of Paleogene mammals in the bases of coarse channel deposits (Lehman & Coulson, 2002) demonstrates that the upper part of the Black Peaks Fm is Paleogene. Dinosaur fossils recovered from mudstone immediately below the Paleogene mammal-bearing channel deposits (Lehman et al., 2006) strongly suggest that the basal contact of the Paleogene channeling represents the boundary between Cretaceous and Paleogene rocks, but it does not necessarily imply that deposition was continuous across the K-Pg boundary itself.

Indeed, magnetostratigraphy and radiometric dates show that significant hiatuses must exist within the Javelina and Black Peaks Fms. Lehman (1990) shows that the lower 30m of the Javelina Fm is of normal polarity, and that the upper part of the Javelina and base of the overlying Black Peaks Fm, is of reversed polarity. This is suggested by Lehman (1990) as representing C30n and C29r (66.398 - 65.688 Ma; Ogg, 2012). However, Lehman et al. (2006) later published a radiometric date of 69.0 +/- 1 Ma for a tuff 60 m above the base of the Javelina Fm, ie. within the reversed polarity zone of Lehman (1990). This means that the reversed polarity sediments around the 69 +/- 1 Ma tuff cannot be C29r, and must therefore correspond to either C30r (68.369 - 68.196 Ma), C31r (71.449 - 69.269 Ma; Ogg, 2012), or an unrecognized reversed subchron or cryptochron.

This means that since the Paleogene mammal-bearing channel deposits of the Black Peaks Fm must be truly Paleogene then significant hiatuses must occur within the reversed polarity zone of the upper Javelina and Black Peaks Fms. There a few possibilities: first the entire upper part of the Javelina and lower Black Peaks Fm may be C30r or C31r and the overlying Paleogene channelling would belong to C29r. Under this interpretation the only hiatus occurs at the base of the Palegene channelling. Alternatively, deposition may be continuous across the K-Pg boundary, however this interpretation would require that at least one hiatus must therefore occur somewhere within the reversed zone between the 69 Ma datum and the base of the Paleogene channelling. Numerous amalgamated channel deposits wiithin this zone show that this is possible.

Biostratigraphy may assist in determining whether deposition is continuous across the K-Pg boundary. In the northern US and Canada, the ceratopsid dinoaur *Triceratops* *prorsus* occurs exclusively in the Cretaceous portion of the C29r zone, with different species occurring in the preceding C30n zone (Scannella et al., 2014). Thus if the reversed zone at the base of the Black Peaks Fm is truly C29r then we might expect to find *T. prorsus* fossils. At the moment, diagnostic ceratopsid fossils have not been recovered from this horizon, and ceratopsids from the underlying Javelina Fm appear to be more basal (and therefore, likely but not necessarily stratigraphically older) than *T. prorsus*, which is supportive of their likely age as C30r or C31r.

Here the Black Peaks Fm is shown with a hiatus between the lower dinosaur-bearing mudstones, and the upper Paleogene mammal-bearing unit.

----

### JAVELINA Fm

**Javelina Fm**

The Javelina Fm comprises~100-120m of fluvial and lacustrine mudstones and sandstones (Lehman, 1989).

**Age of the Javelina:**

Historically the Javelina has been thought to been deposited in the latest Maastrichtian, mainly based on biostratigraphy which proposed that presence of the dinosaurs *Alamosaurus*, *Tyrannosaurus rex* and *Torosaurus* was indicative of a Lancian Land Vertebrate Age (stemming from Lawson, 1976). More recently, a U-Pb date of 69 +/- 0.9 Ma was published from 60 m above base of the Javelina Fm, 90 m below the first Paleogene fossils (Lehman et al., 2006). This would place the dated horizon in the lowermost Upper Maastrichtian, and approximately in the middle of the Edmontonian LVA (i.e. older than the Lancian).

The Javelina Fm is often considered to represent continuous deposition up to and through the K-Pg boundary (e.g. Atchley et al., 2004). If this were the case, then this would mean that the 90m of deposits overlying the 69 Ma horizon represented the 3 m.y. leading up to the K-Pg boundary, and that the ~60 m below might represent 2 m.y. (if average rates of deposition were assumed). This would seem to be an unusually long period of time for such a thin unit, although not impossible. Alternatively, considerable hiatuses (up to 2 m.y.) are suggested to occur within the Javelina Fm (Nordt et al., 2003). This would be consistent with the findings of Fowler (in prep) which proposes that the recently named ceratopsid *Bravoceratops* (collected from the basalmost part of the Javelina Fm), is probably upper Campanian in age.

Magnetostratigraphic analysis (Lehman, 1990b) recovers the basal ~30 m of the Javelina Fm as normal polarity, with the remainder reversed polarity. Combined with the radiometric date, the best fit is that the upper reversed zone corresponds to C31r (71.449 - 69.269 Ma; Ogg, 2012), and the lower part either C32n, or possibly C33n if Fowler (in prep) is correct about faunal similarity to the Campanian.

It is possible that the overlying basal mudstones of the Black Peaks Fm actually represent the fine grained deposits from the last depositional cycle of the Javelina Fm (as illustrated by Atchley et al., 2004). This is further discussed in the Black Peaks entry, but it would mean that there would be technically no depositional hiatus between the Javelina Fm and the overlying Black Peaks Fm.

The Javelina is therefore plotted here as fixed over the 69 Ma radiometric date, but should be considered to have an unknown age for the lower contact, and effectively also an unknown age for the top of the mud unit which conformably overlies the uppermost amalgamated channel unit of the Javelina Fm.

----

### 69.0 ± 0.9

**Lehman et al. (2006)**

Distal tuff

60 m above base of Javelina Fm, 90 m below first Tertiary fossils.

**69.0 +/- 0.9** Ma (2σ); (U-Pb; euhedral monazite; University of Texas, Austin; Lehman et al., 2006).

Lehman et al. (2006) note that unlike typical U-Pb analyses, their date is only based upon the 235U - 207Pb decay system, due to problems with the 238U - 206Pb age being too high.

Although Lehman et al. (2006) state the date places the *Alamosaurus* fauna (within the Javelina Fm) as Lancian to late Edmontonian (NALVA), a date of 69.0 Ma should perhaps be considered middle Edmontonian.

----

### AGUJA Fm

**The Aguja Fm, Big Bend, TX**

The Aguja Fm is 130-285m thick (Sankey, 2001).

The basal contact occurs between the Lower Shale Mbr and the Pen Fm and is uncontroversial. Few diagnostic vertebrate fossils are known from the Lower Shale Mbr.

Controversy surrounds the stratigraphy and age of the Upper Shale Mbr, from which most diagnostic vertebrate material has been recovered. This is important as specimens from this member might include some of the earliest known North American representatives of important dinosaur clades such as the Lambeosaurinae (Wagner & Lehman, 2009), and Chasmosaurinae (Lehman, 1989; Forster et al., 1993). See individual entries.

**Age of the Aguja:**

Age of the Aguja is controversial, with conflicting ages being suggested by biostratigraphy, magnetostratigraphy and radiometric dates. This is discussed in the notes accompanying the individual subunits. Here I show what I think is the most likely age range based on the available data.

----

### Upper Shale Mbr

**Upper Shale Mbr, Aguja Fm**

The Upper Shale Mbr is approximately 120m thick (Lehman, 1989).

**Age & duration**

Age of the Upper Shale Mbr is problematic, despite a number of chrono- and biostratigraphic indicators. Although it has been previously suggested that it might be late Campanian to early Maastrichtian in age (e.g. Nordt et al., 2003; Sankey, 2010), most current data supports a middle Campanian age (e.g. Wagner & Lehman, 2009), although its exact position remains undetermined.

Age of the Upper Shale Mbr is constrained by ammonite biostratigraphy of underlying units and radiometric dates recovered from the uppermost part of the Upper Shale Mbr. Presence of the ammonite *Baculites maclearni* (80.67 - 80.21 Ma; Ogg & Hinnov, 2012) in the underlying Rattlesnake Mountain sst and Terlingua Creek sst mbrs (Rowe et al., 1992; Lehman & Tomlinson, 2004; see individual member notes for details) demonstrates that these units were deposited during the lowermost part of the middle Campanian, within the uppermost part of magnetochron C33r (83.640 - 79.900 Ma; Ogg, 2012).

Sankey & Gose (2001) show that the base of the Upper Shale Mbr is of reversed polarity, and is overlain by a short normal polarity interval, another reversed interval, then another normal interval. The uppermost part of the Upper Shale Mbr is shown as normal polarity by Lehman (1990b). Despite the above ammonite correlation suggesting a C33r / Middle Campanian age, Sankey & Gose (2001; and later publications e.g. Sankey, 2006; 2010) correlate the basal part of the Upper Shale Mbr with C32r (74.309 - 73.649 Ma; Ogg, 2012), asserting a late Campanian age. This placement is unlikely or impossible for two reasons; A U-Pb date from the uppermost Aguja Fm of 76.9 +/- 1.2 Ma (Befus et al., 2008) shows that the base of the Upper Shale Mbr cannot be any younger than this. Secondly, if the C32r age is correct then it requires that a significant hiatus of ~5.7 m.y. occurs between the top of the Terlingua Creek Sandstone Mbr and the base of the Upper Shale Mbr, but no evidence of this is presented.

It is likely that Sankey & Gose's (2001) assignment of C32r was based on the lack of short-duration polarity fluctuations below C32r in the coarse-scale, standardized magnetostratigraphy (Ogg, 2012). However, a number of short duration 'cryptochron' reversals were detected at the base of C33n by Montgomery et al. (1998). Although these are not yet official (i.e. in GTS 2012; Ogg, 2012), if these short reversals are accepted (as considered elsewhere in many publications by J. F. Lerbekmo) then it might mean that the base of the Upper Shale Mbr might correlate with lower C33n. As things stand it is not possible to tell.

The upper part of the Upper Shale Mbr is of normal polarity (Lehman, 1990b). It is expected that this correlates with some part of C33n (79.900- 74.309 Ma; Ogg, 2012), and this is consistent with the 76.9 Ma radiometric date given by Befus et al. (2008).

As such, it is suggested here that the base of the Upper Shale Mbr correlates with the lower part of C33n.

----

### 72.6 ± 1.5

**Breyer et al. (2007)**

Olivine rich basalt block

~Uppermost part of Upper Shale Mbr, Aguja Fm (see below)

72.6 +/- 1.5 Ma (2σ); (U-Pb, 4 zircon samples; SHRIMP-RG; Breyer et al., 2007)

Breyer et al. (2007) give a date of 72.6 Ma for a basalt block recovered from high in the Upper Shale Mbr of the Aguja Fm. Subsequent work by Befus et al. (2008) suggests that the period of phreatomagmatic volcanism occurred after deposition of what would traditionally be considered as the Upper Shale Mbr of the Aguja Fm.

See note on phreatomagmatic volcanism for discussion.

----

### Phreatomagmatic volcanism

**Phreatomagmatic volcanism (Breyer et al., 2007; Befus et al., 2008)**

uppermost Upper Shale Mbr, Aguja Fm

Breyer et al. (2007) and Befus et al. (2008) describe phreatomagamatic volcanism and associated pyroclastic deposits in a geographically restricted area within Big Bend National Park, from what is technically the uppermost part of the Upper Shale Mbr of the Aguja Fm.

**Radiometric dates are younger than most fossil-bearing strata**

Two U-Pb radiometric dates have been reported from these pyroclastic deposits; 72.6 Ma and 76.9 Ma (Breyer et al., 2007; Befus et al., 2008; respectively). These are the only radiometric dates reported from the Aguja Fm, and so it seems reasonable to report these dates as representing the age of the Upper Shale Mbr. However, Befus et al. (2008) present a model showing that two explosive volcanic events created craters within what would traditionally be considered as the Upper Shale Mbr; i.e. that the volcanism occurred after the Upper Shale Mbr had been deposited. As such, it is possible if not likely that diagnostic vertebrate fossils from the Upper Shale Mbr (e.g. *Agujaceratops*; Forster et al., 1993) might be much older than 76.9 Ma, and much closer to the age of the underlying Terlingua Creek Mbr, dated by ammonite biostratigraphy as 80.67 - 80.21 Ma (*B. mclearni* zone; see individual entry).

**Overlying lacustrine fossils**

Lacustrine deposits overlying the 72.6 Ma date have yielded a number of vertebrate fossils, including dinosaur fragments, a crocodile tooth, and turtle remains (Breyer et al., 2007). The dinosaur and crocodilian remains were undiagnostic, but turtle remains were identified to genus level as *Aspideretes*, *Bothremys*, and *Adocus*. The local stratigraphic distribution of these taxa was identified as Aguja Fm (*Bothremys*); Upper Shale Mbr, Aguja Fm (*Adocus*), and Aguja & Black Peaks Fm (*Aspideretes*), hence it was decided that the lacustrine deposits were part of the Upper Shale Mbr of the Aguja Fm. However, these taxa have much broader stratigraphic ranges elsewhere, all ranging up to the Late Maastrichtian (e.g. Gaffney et al., 2006; Holroyd et al., 2014). As such, presence of these turtle taxa are not particularly informative, and is more likely to demonstrate that environmental or preservational conditions were less favorable to these taxa during the Javelina Fm.

----

### 76.9 ± 1.2

**Befus et al. (2008)**

Volcanic bombs

~Uppermost part of Upper Shale Mbr, Aguja Fm (see below)

76.9 +/- 1.2 Ma (2σ); (U-Pb, 3 zircon samples; SHRIMP-RG, Stanford; Befus et al., 2008)

Befus et al. (2008) give a U-Pb date of 76.9 +/- 1.2 Ma for three samples of volcanic bombs removed from the uppermost Upper Shale Mbr of the Aguja Fm at Pena Mountain, Big Bend region, Texas.

See note on Upper Shale Mbr, Aguja Fm, for discussion.

**Note**

This date was incorrectly cited as 77.5 Ma by Loewen et al. (2013b), who cite Roberts et al. (2013) as their source. I cannot find reference to the 77.5 Ma date in Roberts et al. (2013), who instead give the 76.9 +/- 1.2 Ma age from Befus et al. (2008); as a U-Pb date this should not be subject to recalibration.

----

### Terlingua Creek sst Mbr

**Terlingua Creek Sandstone Mbr**

**Age**

Rowe et al. (1992) state that some poorly preserved specimens of *Baculites maclearni* (80.67 - 80. 21 Ma; Ogg & Hinnov, 2012) are known from the Terlingua Creek sst mbr. The Terlingua Creek Sandstone Mbr is therefore a little younger than the Rattlesnake Mt mbr (lower in the Aguja Fm), which also contains *B. maclearni*.

----

### McKinney Sp. Marine Tongue (PEN Fm)

**McKinney Spring Marine Tongue, Pen Fm**

The McKinney Spring Marine Tongue of the Pen Fm splits the Aguja and San Carlos Fms. Ammonites from the overlying and underlying Terlingua Creek and Rattlesnake Mountain Mbrs constrain age of the McKinney Spring Marine Tongue to the *Baculites* *maclearni* zone (see individual notes).

----

### Rattlesnake Mt. Mbr

**Rattlesnake Mountain Sandstone Mbr, Aguja Fm**

The Rattlesnake Mountain Mbr is approximately 10-15m thick (Lehman, 1989).

**Age & Duration**

The age of this unit is constrained by its ammonite fauna, and provides an anchor point for the age of the Aguja Fm in general.

Lehman & Tomlinson (2004) describe a marine turtle recovered from the Rattlesnake Mt. Mbr, and note the presence of biostratigraphically informative ammonites within the unit:

"The Rattlesnake Mountain sst Mbr has yielded biostratigraphically significant inoceramid and ostreid bivalves, and the ammonites *Pachydiscus* *paulsoni*, *Baculites* *maclearni*, and *Hoplitoplacenticeras* cf. *H*. *plasticum*. These strongly suggest a middle Campanian age assignment for this unit."

The *B. maclearni* zone is defined as 80.67 - 80. 21 Ma by Ogg & Hinnov (2012).

----

# MEXICO

## CO - Parras basin

### DIFUNTA GROUP

**Difunta Gp, nr Saltillo, Mexico**

The Difunta Group is approximately 4000m thick (Eberth et al, 2004) and in the Parras Basin comprises the Rancho Nuevo, Las Encinas, Cerro Grnade, Las Imagenes, Can del Tule, Cerro Huerta, and Cerro del Pueblo Fms (Murray et al., 1962; Kirkland et al., 2000; Soegaard et al., 2003; Eberth et al., 2004).

See individual entries for stratigraphic position data.

----

### LAS ENCINAS Fm

**Las Encinas Fm**

The Las Encinas Fm is shown by Eberth et al. (2004) to straddle the K-Pg boundary. The exact positioning of the unit is not stated, and is here shown as a representation of the author's diagram.

----

### CERRO GRANDE Fm

**Cerro Grande Fm**

Ifrim et al. (2010) show the base occuring approximately one third of the way through C30n; top shown at or slightly below the C30n-C29r boundary.

Kirkland et al. (2000) state that a diverse marine invertebrate fauna from the lower two thirds of the unit indicates a Maastrichtian age.

----

### LAS IMAGENES Fm

**Las Imagenes Fm**

Shown as approximately half way through C31n to a third way through C30n by Ifrim et al. (2010).

----

### CANON DEL TULE Fm

**Canon del Tule Fm**

The thickness of the Canon del Tule Fm is shown to be at least 150m by Eberth et al. (2004). It is up to 600 m thick at the tyope section (Murray et al., 1962; Kirkland et al., 2000)

**Magnetostrat**

Eberth et al. (2004) show the base of the Canon del Tule Fm as being just below the upper bound of the 31r.3r zone. The upper bound of the formation is unknown, but it is shown to include the 31r.2r magnetozone (Eberth et al., 2004). Ifrim et al. (2010) show the top of the Canon del Tule Fm occurring near the top of the C31r zone, at approximately the same position as the base of the *Hoploscaphites* *nicolletii* ammonite zone (as shown here).

**Marine fossils**

Kirkland et al. (2000) state that a diverse marine invertebrate fauna indicates a Maastrichtian age for the unit.

----

### CERRO HUERTA Fm

**Cerro Huerta Fm**

The Cerro Huerta Fm is up to 978m thick at the type section (Murray et al., 1962; Kirkland et al., 2000).

**Magnetostrat**

Eberth et al (2004) show the base of the Cerro Huerta Fm as coincident with the base of the 32n.1r zone. The upper boundary with the overlying Canon Tule Fm occurs near the upper boundary of the 31r.3r zone, but does not contain a normal polarity zonation and thus is shown here as coincident with the upper bound of this magnetochron. the 31r.3r zone is correlative with the Drumheller Marine Tongue (Lerbekmo & Braman, 2002).

----

### CERRO DEL PUEBLO Fm

**Cerro del Pueblo Fm**

162m thick at principal reference section, although up to ~540m thick at other sections (Eberth et al, 2004), including 310 m thick in the type area west of Saltillo (Murray et al., 1962; Kirkland et al., 2000)

**Magnetostrat**

Eberth et al (2004) show the base of the Cerro del Pueblo Fm occurring just above the base of the 32n.3r magnetozone, and the upper boundary with the overlying Cerro Huerta as being coincident with the upper boundary of the 32n.2n magnetozone.

**Ammonite biostratigraphy**

Kirkland et al. (2000) state that marine facies withn the Cerro del Pueblo Fm show stratigraphic overlap of the ammonite *Sphenodiscus* with the bivalve *Inoceramus* *vanuxemi*, stating that this is correlative with the *Baculites* *reesidei* and *B. jenseni* ammonite zones of thw Western Interior. This would place the age of the Cerro del Pueblo Fm as upper Campanian (Ogg & Hinnov, 2012).

Here the Cerro del Pueblo Fm is shown occupying the time represented by the *B. reesidei* and *B. jenseni* ammonite zones. Although this is shown here as not agreeing with the magnetostratigraphic placement of Eberth et al. (2004; see above), this will robably be reconciled in the future when more precise placement of the 32n.3r cryptochron is esablished (see discussion in note for the 73.5 Ma radiometric date, Drumheller Mbr, Horseshoe Canyon Fm, Alberta).

----

### PARRAS SHALE

**Parras Shale**

The Parras shale comprises over 700 m of dark grey to black calcareous marine shales with thin sandstones and siltstones (Soegaard et al., 2003).

**Magnetosratigraphy**

The magnetostratigraphic analysis of Eberth et al., (2004) sampled the upper ~300 m of the Parras Shale, recovering a mostly normal polarity interval, with only a short reversal occurring at ~220 m. They correlated this to 32n.5n, 32n.4r, and 32n.4n, based on Lerbekmo & Braman (2002), which compares with the definition of C32n of 73.649 - 71.449 Ma (Ogg, 2012). This might be contradicted by more recently published biostratigraphic data.

**Biostratigraphy**

Ifrim et al., (2013) described the first fossil assemblage collected from the Parras Shale. This assemblage of ammonites is mostly endemic, but comparison to Western Interior and European ammonite zones shows that the assemblage is correlated to the upper part of the Lower Campanian, equivalent to the *S. hippocrepis III* zone of the Western Interior (81.53 - 81.28 Ma; Ogg & Hinnov, 2012).

Thus, the new biostratigraphic data (Ifrim et al., 2013) probably disagrees with the designation of magnetozones within C32n (Eberth et al., 2004). It is possible that the ammonites were sampled from a part of the Parras Shale stratigraphically lower than the section sampled by Eberth et al. (2004), but this is not stated by Ifrim et al. (2013). There is a possible problem with this older interpretation in that the lower Campanian falls entirely within the reversed polarity C33r. Although some normal subzones have been detected within C33r (see magnetostratigraphic column), none are particularly long in duration, such that they might not be likely to correspond to the normal zones detected at the top of the Paarras Shale by Eberth et al. (2004). It is possible that the normal zones described by Eberth et al. (2004) may correspond to two of the many long normal zones within the succeeding C33n, but this is speculative. more work on the age of the Parras Shale is needed.

These conflicting ages make plotting of the Parras Shale a problem, and have repercussions for the age of the overlying Difunta Group. The plot here incorporates the biostratigraphic data of Ifrim et al. (2013), but should be considered tentative in terms of placement of the upper contact with the Difunta group.

----

## Michoacan state

### UNNAMED Fm

**Unnamed Fm, Michoacan state**

An unnamed unit is included here as it has yielded holotypic dinosaur remains (Ramirez-Velasco et al., 2012) which have been plotted in the faunal section.

The unnamed unit comprises a basal conglomerate, overlain by fluvial sandstones, siltstones, volcanicalstics, and some limestones (Benammi et al., 2006)

**Age**

Precise chronostratigraphic work has not yet been conducted. However, in an abstract, Benammi et al. (2006) assign the unit to the upper part of magnetochron C34n, and cite a radiometric date of 84 +/- 2.8 Ma (the method of analysis is not given).

The unit is shown here as occurring within the Santonian, pending more detailed data becoming available.

# MAGNETOSTRAT

## Integrated

**High resolution magnetostratigraphy**

This column shows the high resolution paleomag where available. This is still very incomplete and is mainly drawn from the high-resolution magnetostratigraphic analyses of J.F. Lerbekmo.

Significantly, Lerbekmo detects and names many more subchrons than are represented in standardized magnetostratigraphy provided in The Geological Timescale (Ogg, 2012). Moreover, some chrons have slightly different boundary ages and durations (for example, C32r). Details of this are given in the notes accompanying individual chrons (etc).

Very short duration "tiny wiggles" in polarity are also known as "cryptochrons" (see Lerbekmo & Evans, 2012). It should be noted that Bouligand et al. (2006) suggested that these tiny wiggles in polarity profiles were due to field intensity variation, rather than true polarity change.

----

### C29n

**C29n:**

top=64.958

base=65.688

Ogg (2012)

top=64.432

base=65.118

Ogg & Smith (2004)

----

### C29r

**C29r:**

top=65.688

base=66.398

Ogg (2012)

top=65.118

base= 65.861

Ogg & Smith (2004)

Lerbekmo et al. (1996) describe a short normal subchron (29r.1n) occurring coincident with the iridium anomaly at the K-Pg boundary, typically occurring ~1 m below the anomaly, and extending one or more meters above it. Subchron 29r.1n has been detected in the Scollard and Frenchman Fms, Canada, the Hell Creek Fm, Montana, and in at least two cores from the Atlantic Ocean drilled as part of the deep sea drilling project (Lerbekmo et al., 1996; Lerbekmo, 1999; Lerbekmo, 2014; although not detected in the Hell Creek Fm by LeCain et al., 2014). A similarly placed normal subchron was detected 15-20m below the K-T boundary in the Scollard Fm, Red Deer River, Alberta (Lerbekmo & Coulter, 1985). It is not yet clear whether this represents slight inconsistency in placement, or two separate normal polarity horizons, or that a small hiatus exists at the top of the Frenchman Fm.

On this chart, a single normal horizon is shown at the K-Pg boundary although it should be noted that this may change slightly.

----

### 29r.1n

**29r.1n**

Lerbekmo et al. (1996) describe a short normal subchron (29r.1n) occurring coincident with the iridium anomaly at the K-Pg boundary, typically occurring ~1 m below the anomaly, and extending one or more meters above it. Subchron 29r.1n has been detected in the Scollard and Frenchman Fms, Canada, the Hell Creek Fm, Montana, and in at least two cores from the Atlantic Ocean drilled as part of the deep sea drilling project (Lerbekmo et al., 1996; Lerbekmo, 1999; Lerbekmo, 2014; although not detected in the Hell Creek Fm by LeCain et al., 2014). A similarly placed normal subchron was detected 15-20m below the K-T boundary in the Scollard Fm, Red Deer River, Alberta (Lerbekmo & Coulter, 1985). It is not yet clear whether this represents slight inconsistency in placement, or two separate normal polarity horizons, or that a small hiatus exists at the top of the Frenchman Fm.

On this chart, a single normal horizon is shown at the K-Pg boundary although it should be noted that this may change slightly.

----

### C30n

**C30n:**

top=66.398

base=68.196

(Ogg, 2012)

top= 65.861

base=67.696

(Ogg & Smith, 2004)

Lerbekmo (1999) found no evidence for short-duration reversals within C30n.

----

### C30r

**C30r:**

top=68.196

base=68.369

(Ogg, 2012)

top=67.696

base=67.809

(Ogg & Smith, 2004)

Shown by Lerbekmo & Braman (2002) as occurring between coal seams 11 and 12 at the top of the Horseshoe Canyon Fm, Alberta. Lerbekmo (2009), Lerbekmo & Braman (2002, 2005), and Lerbekmo & Coulter (1985) show the C30r-C30n boundary occurring at the base of the Whitemud (Saskatchewan) and regional equivalent, the Colgate Sandstone (Montana).

----

### C31n

**C31n:**

top=68.369; base=69.269

(Ogg, 2012)

top=67.809; base=68.732

(Ogg & Smith, 2004)

Ogg & Smith (2004) comment:

"base of chron 31n constrained by Ar-Ar ages to ~69.0 +/- 0.5Ma (2-sigma)

Lerbekmo (2009) illustrates and names 31n.1n, 31n.1r and 31n.2n, for subzones detected in the Fox Hills Fm of Montana, and the Eastend Fm of Saskatchewan. I think it is likely that the Montana occurrences are actually part of C31r, based on ammonite biostratigraphy of the Fox Hills Fm (Gill & Cobban, 1973; see Fox Hills entry). Indeed, in the original source of the Lerbekmo (2009) magnetostratigraphic data (Lerbekmo, 1985) the reversed polarity part of the Eastend Fm is assigned to C31r, consistent with my previous point. I therefore find no evidence that there are short subzones within C31n, although it would not be surprising if they are merely as yet undetected.

----

### C31r

**C31r:**

top=69.269

base=71.449

(Ogg, 2012)

top=68.732

base=70.961

(Ogg & Smith, 2004)

Ogg & Smith (2004) comment:

"base of chron 31r constrained by Ar-Ar ages to ~70.45 +/- 0.65 Ma (2-sigma)"

Lerbekmo & Braman (2002; 2005) illustrate a series of short subchrons within C31r recorded within the upper part (now Tolman Mbr; Eberth & Braman, 2012) of the Horseshoe Canyon Fm, Alberta. C31r.2n is correlated with the Drumheller Marine Tongue and an associated radiometric date (see notes) but other subchrons are not constrained by independent biostratigraphic or chronostratigraphic indicators. As such their placement here is based on simple metre-scale measurements within the Horseshoe Canyon Formation, as reported by Lerbekmo & Braman (2002; 2005), and are thus tentative.

----

### 31r.1n

**31r.1n**

Subdivided by Lerbekmo & Braman (2005) into seven tightly clustered sub-subzones;

31r.1n-1n

31r.1n-1r

31r.1n-2n

31r.1n-2r

31r.1n-3n

31r.1n-3r

31r.1n-4n

----

### 31r.2n

**31r.2n**

Coincident with the maximum flooding surface of the Drumheller Marine Tongue (Horseshoe Canyon Fm, Alberta; Lerbekmo & Braman, 2002), which is dated at 70.84 Ma by an ash (recalibrated, this article; see ash date entry; original date 70.4 Ma; Eberth & Deino, 2005; Eberth & Braman, 2012).

Subdivided by Lerbekmo & Braman (2005) into three tightly clustered sub-subzones observed only in the CPOG Strathmore core:

31r.2n-1n

31r.2n-1r

31r.2n-2n

----

### 31r.3r

**31r.3r**

Short reversed polarity subchron below the Drumheller Marine Tongue (DMT), Horseshoe Canyon Fm, Alberta (Lerbekmo & Braman, 2002).

Duration is constrained by the age of the overlying 31r.2n (radiometric date 70.84 Ma; see entry for 31r.2n) and the top of the underlying 32n zone (defined as 71.449 Ma; Ogg, 2012). This means that despite this interval being represented by a relatively thin <10m of section by Lerbekmo & Braman (2002), it is calculated here as being ~600k.y. in duration. However, this might be expected given that prior to deposition of the DMT, there might be a regressive unit and hence a condensed section.

----

### C32n

**C32n**

top=71.449

base=73.649

(Ogg, 2012)

top=70.961

base=72.979

(Ogg & Smith, 2004)

Ogg & Smith (2004) comment:

"base of chron 31r constrained by Ar-Ar ages to ~70.45 +/- 0.65 Ma (2-sigma)"

Lerbekmo & Braman (2002; 2005) and Lerbekmo & Lehtola (2011) show 11 subchrons of C32n occurring in the Horseshoe Canyon and Bearpaw Fms Alberta. There are few good chronostratigraphic horizons to aid in positioning these subchrons, and ammonite data from Lerbekmo & Braman (2002) and Lerbekmo & Lehtola (2011) conflicts with new ammonite ranges defined by Ogg & Hinnov (2012). Some of these problems may be rectified if hiatuses within the Horseshoe Canyon Fm are recognised, but without good chronostratigraphic control, this will be difficult. These issues are noted where they occur. As such the C32n subchron positions presented here are tentative, and expected to change.

----

### 32n.1n to 32n.3n

**32n.1n to 32n.3n**

Lerbekmo & Braman (2002; 2005) show five subchrons (32n.1n to 32n.3n) sandwiched between 31r.3r (just beneath the Drumheller Marine Tongue), and Drumheller coal zone #8-9.

A detrital zircon U-Pb date of 71.923 +/-0.068 Ma published by Davies et al. (2014) was recovered from ~4 m above Big Island coal seam (#9) in Northern Alberta that is equivalent to Drumheller coal zone #8-9. This information is used to position Drumheller coal zone #8-9 at ~ 71.6 Ma (see note for Horsethief Mbr, Horseshoe Canyon Fm), thus subchrons 32n.1n to 32n.3n (uppermost part) are tentatively constrained as occurring from 71.6 to 71.449 Ma (top of C32n; Ogg 2012).

----

### 32n.3r

**32n.3r**

Short reversed polarity subchron that occurs roughly half way between Drumheller coal zone #6-7 and #8-9 (Horseshoe Canyon Fm, Alberta; Lerbekmo & Braman, 2002; 2005)

----

### 32n.4r

**32n.4r**

Short reversed polarity subchron that occurs slightly below Drumheller coal zone #6-7 (Horseshoe Canyon Fm, Alberta; Lerbekmo & Braman, 2005).

Lerbekmo & Braman (2002) and Lerbekmo & Lehtola (2011) show 32n.4r as occurring near the base of the B. reesidei ammonite zone. However, the new definition of this zone by Ogg & Hinnov (2012) make this impossible to plot here.

----

### 32n.5r

**32n.5r**

Short reversed polarity subchron that occurs between Drumheller coals #0 and #1 (Horseshoe Canyon Fm, Alberta; Lerbekmo & Braman, 2002; 2005). This is shown as occurring between the *B. reesidei* and *B. cuneatus* ammonite zones by Lerbekmo & Braman (2002) and Lerbekmo & Lehtola (2011).

----

### C32r

**C32r**:

top=73.649

base=74.309

(Ogg, 2012)

Lerbekmo & Braman (2002), and Lerbekmo & Lehtola (2011) show C32r as a short chron beginning within the *B. cuneatus* ammonite zone, and ending before the *B. reesidei* ammonite zone. The short duration of these ammonite zones as defined by Ogg & Hinnov (2012) renders C32r a very short chron.

Furthermore, Lerbekmo & Braman (2002, 2005) show a fairly short normal subchron occurring at the base of the *B. cuneatus* ammonite zone, which they name as 33n.1n.

This short normal subchron also contains the Dorothy bentonite, which has been radiometrically dated at 73.5Ma +/- 0.4 (Rb-Sr; biotite; Lerbekmo, 2002). This presents a problem as this date does not fit within the range of either *B. cuneatus* or C33n as stated in GTS 2012 (Ogg, 2012; Ogg & Hinnov, 2012; respectively). Hence here I have moved the upper boundary of 33n up such that the radiometric date and ammonite zone correlate as shown by Lerbekmo and Braman (2002; 2005). This squeezes the duration of C32r.

I suspect that these chron positions and ammonite dates may change, and as such they are tentative as shown here.

----

### 33n.1n

**33n.1n**

Lerbekmo & Braman (2002, 2005) show a fairly short normal subchron occurring at the base of the *B. cuneatus* ammonite zone, which they name as 33n.1n.

This short normal subchron also contains the Dorothy bentonite, which has been radiometrically dated at 73.5Ma +/- 0.4 (Rb-Sr; biotite; Lerbekmo, 2002). This presents a problem as this date does not fit within the range of either *B. cuneatus* or C33n as stated in GTS 2012 (Ogg, 2012; Ogg & Hinnov, 2012; respectively). Hence here I have moved the upper boundary of 33n up such that the radiometric date and ammonite zone correlate as shown by Lerbekmo and Braman (2002; 2005).

----

### C33n

**C33n**

top=74.309

base=79.900

(Ogg, 2012)

The C33r-C33n boundary is revised to 78.91 Ma by Albright & Titus (2016) based on the magnetostratigraphy of the middle to upper mbrs of the Wahweap Fm. The C33r-C33n boundary occurs near the top of the upper Mbr, above an Ar / Ar date of 79.9 +/-0.3 Ma in the middle mbr, hence the age of the boundary was revised to be younger (as shown here).

**subzones within 33n**

Chron 33n is usually noted for being a long uninterrupted normal polarity zone. However, a number of reversals have been found in at least the upper and lower parts. I have not yet found a high resolution study that definitely covers the middle.

At the top of C33n, Lerbekmo & Braman (2002) noted a reversal roughly halfway through the Bearpaw Fm and identified it as subchron 33n.1r. Lerbekmo (2005) and Lerbekmo et al (2003) note a slightly older reversal near the base of the Bearpaw, named as subchron 33n.2r.

Lerbekmo (2005) notes that Hicks et al (1999) misidentify a reversal in the B. compressus zone as chron 32r. Instead Lerbekmo suggests that this must be 33n.3r. Lerbekmo asserts that this must be the same reversal as he found approximatey 22m from the base of the 75m thick DPFm. This does not seem to correlate however (using new dates from Eberth, 2005), and it is possible that this represents a further 33n.4r reversal zone. One of these reversals may match to that detected in the Kirtland Fm NM by Butler & Lindsay (1985), or this may represent yet another newly identified short reversal..

Montgomery et al (1998) detected a number of short reversals in C33n directly above the boundary with the underlying C33r. The extent to which these reach up into C33n is not clear, however they do fall entirely with the *Belemnitella* *mucronata* zone. This zone is variably equivalent to just the lower Campanian (Christiansen, 1996), or the middle to upper Campanian (Ogg et al, 2004). It is also not clear from Montgomery et al's (1998) diagram how far into the *B*. *mucronata* zone these magnetic reversals occur.

**Older dates**

The boundary between 33r and 33n is dated as 79.34Ma by Hicks et al (1995)

base=79.543 (Ogg & Smith, 2004)

----

### 33n.3r

**33n.3r**

Lerbekmo (2005) shows a short reversal 20-23 m above the base of the Dinosaur Park Fm, Alberta, which he then names as 33n.3r.

The age of 33n.3r is constrained by an overlying radiometric age of 76.39 Ma for an ash 36 m above the base of the Dinosaur Park Fm (Eberth, 2011; see individual entry).

----

C33r

**C33r**:

top=79.900

base=83.640

(Ogg, 2012)

**subzones in C33r:**

In a study of the British chalk, Montgomery et al. (1998) detected two short normal subzones near the top of C33r. As yet, I have not found reference to these in magnetostratigraphic analyses of North American sections. The work of Montgomery et al. (1998) wil be incorporated into a future version of the chart.

Three 33r normal subzones are illustrated but not named in Leahy & Lerbekmo (1995), occurring close together in the *B. obtusus* zone, between ~40-60m from the base of the ~70m thick lower mbr of the Pakowiki Fm. A swarm of four normal polarity subzones was detected in at approximately the same level in C33r by Montgomery et al (1998). Here I have shown only 3 subzones at this level, mainly due to limitations in the resolution of the chart.

The lowermost normal subzone occurs at the top of the Deadhorse Coulee Mbr of the Milk River Fm (Leahy & Lerbekmo, 1995). A similarly aged normal subchron was also detected by Montgomery et al (1998).

The exact placing and thicknesses of subchrons shown here are provisional. It should be noted that Montgomery et al (1998) note that some of their detected subchrons may be due to magnetic overprinting. given that many of their subchrons correlate well with the work of others, it lends support to their method being correct.

----

### C34n

**C34n:**

top=83.64

base=125.93

(Ogg, 2012)

C34n is a superchron, referred to as the "Cretaceous Long Normal-Polarity Chron", and extends from the Early Aptian (125.93 Ma) to the Santonian-Campanian Boundary (83.64 Ma) a duration of ~35 m.y. (Ogg, 2012).

**Subzones in 34n:**

The single reversed subzone in 34n is shown by Leahy & Lerbekmo, (1995) but not specifically named. It is positioned at the base of the Deadhorse Coulee Mbr of the Milk River Fm, in the uppermost part of C34n, within the *D. bassleri* ammonite zone.

In a study of the British chalk, Montgomery et al. (1998) detected a number of reversed subzones within C34n. As yet, I have not found reference to these in magnetostratigraphic analyses of North American sections. The work of Montgomery et al. (1998) will be incorporated into a future version of the chart.

----

## GTS 2012

**Magnetostratigraphy (Ogg, 2012)**

This column shows the paleomagnetostratigraphic chrons defined in The Geologic Timescale (Ogg, 2012). A column incorporating more independent studies is available to the left.

Note that an alternative set of chron boundary definitions is available in Gee and Kent (2007), although this is not used here.

----

### C29n

**C29n**

Top=64.958 Ma

Base=65.688 Ma

Ogg (2012)

Top=64.432 Ma

Base=65.118 Ma

Ogg & Smith (2004)

----

### C29r

**C29r**

Top=65.688 Ma

Base=66.398 Ma

Ogg (2012)

(previously; Ogg & Smith, 2004)

Top=65.118 Ma

Base= 65.861 Ma

Schoene et al. (2015) place the C30n-C29r boundary at ~66.288 +/- 0.027 Ma, based on a U-Pb radiometric date within a "transitional" polarity horizon in the Jawhar Fm, Deccan Traps, India.

Sprain et al. (2014) calculate an age of 66.177 +/- 0.032 / 0.044 Ma for the base of C29r, based on Ar-Ar dating of the "Null Coal tephra" (66.289 +/- 0.051 Ma) which occurs slightly below the C30n - C29r boundary in the Hell Creek Fm, Montana. Firstly, this Ar-Ar analysis uses the Renne et al. (2011) values for the FCT standard and λT, leading to slightly older ages than the Kuiper et al. (2008), Min et al. (2000) values used in GTS 2012 (Gradstein et al., 2012) and here (see notes for individual radiometric dates in main chart). Also, this date for C30n-C29r depends upon a measured section of Archibald et al. (1982) where the Null Coal occurs ~21 m below the C30n, which is significantly different from more recent measured sections by myself (at the same locality), and LeCain et al. (2014), where the Null Coal occurs immediately beneath the Apex Sandstone, ~ 5 m beneath the first mudstone from which the C29r reversal can be detected. Thus, I am skeptical about this date for C30n-C29r. However, this study does the raise the issue that if the 66.289 Ma age for the normal-polarity Null Coal is correct, then the C30n-C29r boundary must be younger than this (highlighted by Sprain et al., 2014, as the conservative hypothesis).

----

### C30n

**C30n**

Top=66.398 Ma

Base=68.196 Ma

(Ogg, 2012)

Top= 65.861 Ma

Base=67.696 Ma

(Ogg & Smith, 2004)

----

### C31n

**C31n**

Top=68.369 Ma

Base=69.269 Ma

(Ogg, 2012)

Top=67.809 Ma

Base=68.732 Ma

(Ogg & Smith, 2004)

Ogg & Smith (2004) comment:

"base of chron 31n constrained by Ar-Ar ages to ~69.0 +/- 0.5Ma (2-sigma)"

----

### C31r

**C31r**

Top=69.269 Ma

Base=71.449 Ma

(Ogg, 2012)

Top=68.732 Ma

Base=70.961 Ma

(Ogg & Smith, 2004)

Ogg & Smith (2004) comment:

"base of chron 31r constrained by Ar-Ar ages to ~70.45 +/- 0.65 Ma (2-sigma)"

----

### C32n

**C32n**

Top=71.449 Ma

Base=73.649 Ma

(Ogg, 2012)

**Defined** **subchrons**

C32n.1n

Top=71.449 Ma

Base=71.689 Ma

(Ogg, 2012)

C32n.1r

Top=71.689 Ma

Base=71.939 Ma

(Ogg, 2012)

C32n.2n

Top=71.939 Ma

Base=73.649 Ma

(Ogg, 2012)

**(old information)**

Top=70.961 Ma

Base=72.979 Ma

(Ogg & Smith, 2004)

----

### C32r

**C32r**

Top=73.649 Ma

Base=74.309 Ma

(Ogg, 2012)

**Defined subchrons**

C32r.1r

Top=73.649 Ma

Base=73.949 Ma

(Ogg, 2012)

C32r.1n

Top=73.949 Ma

Base=74.049 Ma

(Ogg, 2012)

C32r.2r

Top=74.049 Ma

Base=74.309 Ma

(Ogg, 2012)Top=73.649 Ma

Base=74.309 Ma

(Ogg, 2012)

----

### C33n

**C33n**

Top=74.309 Ma

Base=79.900 Ma

(Ogg, 2012)

----

### C33r

**C33r**

Top=79.900 Ma

Base=83.640 Ma

(Ogg, 2012)

----

### C34n

**C34n**

Top=83.64 Ma

Base=125.93 Ma

(Ogg, 2012)

C34n is a superchron, referred to as the "Cretaceous Long Normal-Polarity Chron", and extends from the Early Aptian (125.93 Ma) to the Santonian-Campanian Boundary (83.64 Ma), a duration of ~35 m.y. (Ogg, 2012).

----

# NALVA

**North American Land Vertebrate Ages (NALVAs)**

North American Land Vertebrate Ages have undergone many revisions since Russell (1964; 1975) proposed the Aquilian, Judithian, Edmontonian, Lancian for the Late Cretaceous. Lillegraven & McKenna (1986) redefined the Aquilian, Judithian, and Lancian based on mammal fossils (reviewed and updated by Cifelli et al., 2004). Other vertebrates (including dinosaurs) were included again in the definition of the new NALVA the "Kirtlandian" by Sullivan & Lucas (2003; 2006), and the concept further expanded by Lucas et al. (2012) who erect and redefine many new LVAs covering the entire North American Cretaceous (Fencelakean, Mussentuchian, Cashenranchian, Buffalogapian, Comobluffian).

Although still in use by mammal workers, the utility of NALVAs is debatable, at least from a stratigraphic perspective. Indeed, some workers have abandoned their usage altogether, relying instead on magnetostratigraphy and radiometric dating.

Here I have included NALVAs mainly as guidelines to show comparably aged units. The newer NALVAs proposed by Lucas et al. (2012) are not yet in general use and so are not featured here.

**Definitions**

In their original definition, Russell (1964) recognized that characteristic faunas were separated by gaps in the record. Here, however, the boundaries between NALVAs (see individual entries) are defined by first appearances of particular taxa. As such there should not technically be stratigraphic gaps or hiatuses between NALVAs.

**Terminology**

With the recent reintroduction of non-mammalian vertebrates into definitions of the Judithian (etc.), here I use the more inclusive term NALVA, rather than the mammal-only North American Land Mammal Ages (NALMA).

----

### PUERCAN

**Puercan NALVA**

Defined by the first appearance of the mammal *Protungulatum donnae*, although the Lancian-Puercan boundary is drawn essentially at the K-Pg boundary (Cifelli et al., 2004). The basal boundary with the Lancian is drawn here at the K-Pg boundary.

----

### LANCIAN

**Lancian NALVA**

Although many studies exist which describe microfossil faunas from Lancian-age sediments, Cifelli et al. (2004) state that defining the beginning of the Lancian is problematic. Specifically they note questionable occurrences of some critical taxa in units that are considered Edmontonian. They specifically state that they do not propose a first appearance datum for the Lancian, but suggest that Batodon (eutherian), *Glasbius* (marsupial), and *Essonodon* (multituberculate) would be the best candidates.

In their redefinition of NALVAs, Lucas et al. (2012) redefine the onset of the Lancian NALVA as the first appearance of the chasmosaurine ceratopsid dinosaur *Triceratops* *horridus*. However, as shown here and by Scannella et al. (2014), it is likely that *T*. *horridus* first occurs in the lower part of the middle third of the Hell Creek Formation (the *Triceratops* species recovered from the lower third of the Hell Creek Fm is here referred to as *T*. sp1).

Regardless, here I depict the Lancian as of equal duration to the Hell Creek Fm of Montana, which is in keeping with the general meaning and use of the term. Although it may seem preferable to equate it to the Lance Fm of Wyoming, in fact there is little stratigraphic work conducted on the type Lance Fm, such that we do not have much idea as to whether it is comparable in duration to regional equivalents (Fowler, in prep; see individual entry).

----

### EDMONTONIAN

**Edmontonian NALVA**

Although they recognize that there is a significant temporal separation between Judithian and Lancian NLAVAs, Cifelli et al. (2004) state that formal recognition of an "Edmontonian" age (Russell, 1964; 1975) is not yet possible, and follow the convention of Lillegraven & McKenna (1986) in placing it in inverted commas. Note that Cifelli et al. (2004) do not discuss the Kirtlandian.

Lucas et al. (2012) define the base of the Edmontonian as the first appearance of the hadrosaurid *Edmontosaurus*.

Here I necessarily follow the definition of Lucas et al. (2012).

----

### KIRTLANDIAN

**Kirtlandian NALVA**

The Kirtlandian is a relatively newly defined NALVA, having been erected by Sullivan & Lucas (2003) to give a name to a stratigraphic gap that existed between the Judithian and Edmontonian NALVAs, as originally recognized by Russell (1964; 1975).

In their rediagnosis of the Kirtlandian, Sullivan & Lucas (2006) state that the beginning of the Kirtlandian is defined as the first appearance of the chasmosaurine ceratopsid dinosaur *Pentaceratops* *sternbergii*. The beginning of the succeeding Edmontonian (heretofore defined imprecisely; Cifelli et al., 2004), is defined by the first appearance of *Edmontosaurus* *regalis* (Sullivan & Lucas, 2006).

----

### JUDITHIAN

**Judithian NALVA**

Cifelli et al. (2004) state that the basis for the Judithian NALVA is a fauna recovered from near the top of the Judith River Fm in Choteau and Blaine counties, Montana, published by Sahni (1972). However, this is not particularly useful in helping define the base of the Judithian. Cifelli et al. (2004) note that Judithian faunas have been collected from the Foremost Fm, Alberta.

Lucas et al. (2012) define the beginning of the Judithian as the first appearance of the pachycephalosaurid dinosaur *Colepiocephale* *lambei*.

Given the ambiguity in precisely placing either of these definitions, here I have illustrated the base of the Judithian as correlating with the base of the Foremost Fm, Alberta. This positioning is roughly correlative with both definitions given above, although slightly counterintuitive since it extends the Judithian into sediments older than the surface exposures of the Judith River Fm in Montana.

----

### AQUILIAN

**Aquilian NALVA**

Cifelli et al. (2004, p.23) state that the Aquilian is "characterized on the basis of a mammalian fauna from Verdigris Coulee, in upper parts of the Milk River Formation, Alberta (Lillegraven and McKenna 1986)", further stating notable first appearances of the mammal taxa Mesodma (multituberculate), *Eodelphis* (stagodontid marsupial), and *Paranyctoides* (lipotyphlan insectivore).

Lucas et al. (2012) explicitly define the base of the Aquilian as the first appearance of the eutherian mammal *Paranyctoides* *maleficus*. This essentially follows the definition.

In accordance with these definitions, here I show the base of the Aquilian correlated with the base of the Deadhorse Coulee (uppermost) Mbr of the Milk River Fm.

----

# Dinosauria

## Chasmosaurinae

### *Kosmoceratops richardsoni*

***Kosmoceratops richardsoni* (Sampson et al., 2010)**

**Kaiparowits Fm, UT**

The *Kosmoceratops* holotype comprises a complete skull (UMNH VP 17000; Sampson et al., 2010). A referred specimen comprises the posterior part of a frill from a smaller, presumably juvenile specimen (UMNH VP 16878l Loewen et al., 2013b).

The stratigraphic position of *Kosmoceratops* specimens was plotted by Loewen et al. (2013b) relative to a series of radiometrically dated bentonites, the precise positions and ages of which are most recently summarized in Roberts et al. (2013). The relevant bentonites and *Kosmoceratops* specimens are summarized below:

490m 75.51 Ma

420m 75.51 Ma

~200m Referred specimen UMNH VP 16878

190m 75.97 Ma

~170m Holotype specimen UMNH VP 17000

80m 76.46 Ma

Thus the two *Kosmoceratops* specimens were recovered slightly above and slightly below a bentonite dated at 75.97 Ma and are shown here as a single cell at 75.9 Ma.

----

### *Vagaceratops (Chasmosaurus) irvinensis*

***Vagaceratops (Chasmosaurus)*** ***irvinensis* (Holmes et al., 2001)**

**Dinosaur Park Fm, Alberta, CAN**

Holmes et al describe *Chasmosaurus* *irvinensis* from holotype (NMC 41357) which is a near complete skull; referred specimens are TMP 87.45.1 (skull missing most of the parietal and right squamosal) and TMP 98.102.8 (exploded skull, only possible to reconstruct the parietal border). Sampson et al. (2010) renamed the taxon as *Vagaceratops* *irvinensis*.

**Stratigraphy**

Vagaceratops was collected from the uppermost part of the Dinosaur Park Formation, Alberta, just below the Lethbridge Coal Zone (Holmes et al., 2001).

The holotype (CMN 41357) and one of the referred specimens (TMP 98.102.8) were found outside of Dinosaur Provincial Park, so their stratigraphic position is not as precisely known as the third specimen (TMP 1987.45.1). Holmes et al. (2001; p. 1433) state that the holotype (CMN 41357) derives from a layer "within 20m of the Lethbridge Coal Zone". The referred specimens derive from "within 20m and 10m, respectively, of the Lethbridge Coal Zone" (Holmes et al., 2001; p. 1433) although it is not made clear which of the referred specimens is which. However, Currie & Russell (2005) and Mallon et al. (2014) provide some stratigraphic data for the referred skull TMP 1987.45.1, showing the quarry (no. 184) at 712 m above sea level, and 50.4 m (± 7 m) above the Oldman - Dinosaur Park formational contact (respectively). This places V. irvinensis ~11.1 m below a 76.10 Ma bentonite (which occurs 61.5 m above the formational contact; 3.5 m into the Lethbridge Coal Zone) and 24.4 m above a 76.39 Ma bentonite (occurring at 36 m above the formational contact; dates from Eberth, 2011; recalibrated here).

Hence here I show *V. irvinensis* occurring at 76.1 - 76.2 Ma.

----

### *Chasmosaurus belli*

***Chasmosaurus belli* (Lambe, 1914 =*Monoclonius* *belli*, Lambe, 1902)**

**Dinosaur Park Fm, Alberta, CAN**

*Chasmosaurus belli* was originally described as *Monoclonius belli* by Lambe (1902) based on a relatively complete parietal (CMN 491), including the midline of the posterior bar. After a series of changes, the taxonomy was stabilized by Lambe (1914) who changed it to *Chasmosaurus belli* while describing a new skull and skeleton (CMN 2245). Since this time many specimens have been referred to *C. belli*. However, only six specimens have the diagnostic parietal posterior bar intact; holotype CMN 491 (Lambe, 1902), CMN 2245 (Lambe, 1914), AMNH 5402 (Lull, 1933); NHMUK R4948 (Maidment and Barrett, 2011); ROM 843 (Godfrey and Holmes, 1995), and YPM 2016 (Lull, 1933).

Stratigraphic position is only known for four specimens of *C. belli* that exhibit the posterior parietal bar, all of which derive from the middle to upper part of the Dinosaur Park Formation (given here in stratigraphic order), straddling a 76.39 Ma bentonite at 36 m above the Oldman - Dinosaur Park Formation contact, and below a 76.10 Ma bentonite at 61.5 m. NHMUK R4948 was recovered from an estimated 20.1 m above the Oldman - Dinosaur Park Formation contact (Mallon et al., 2012). YPM 2016 was recovered at 684 m above sea level (Currie and Russell, 2005), estimated at 24.5 m above the Oldman - Dinosaur Park Formation contact (Currie and Russell, 2005; Mallon et al., 2012). ROM 843 was recovered from 682 m above sea level (Currie and Russell, 2005), estimated at 33.4 m above the Oldman - Dinosaur Park Formation contact (Mallon et al., 2012). Finally, CMN 2245 was recovered at 692 m above sea level (Currie and Russell, 2005), estimated at 41.0 m above the Oldman - Dinosaur Park Formation contact (Mallon et al., 2012).

Hence here I show *C. belli* occurring between 76.5 and 76.3 Ma.

----

### "*Chasmosaurus priscus*"

**"*Chasmosaurus priscus*" (Longrich, 2015)**

**Dinosaur Park Fm, Alberta, CAN**

Many chasmosaurine specimens have been assigned to *Chasmosaurus* *russelli* since its description by Sternberg (1940). Most recently Longrich (2010; 2015) reassigned many to either "*Mojoceratops* *perifania*" or "*Chasmosaurus* *priscus*". Specimens assigned to Mojoceratops are problematic in that they are either immature and / or lack any stratigraphic data. Most authors consider Mojoceratops as a junior synonym of *Chasmosaurus* *russelli* (e.g. Madment & Barrett, 2010), however as is becoming clear, the holotype of *Chasmosaurus* *russelli* might not be related to the other specimens assigned to the taxon.

Revision of this problem is beyond the scope of this note, but is the subject of Fowler (2016). Here I follow Longrich (2015) in his renaming of one of the referred specimens (CMN 2280 ) as "*Chasmosaurus* *priscus*"; I only include it in inverted commas as it may need further refinement, and it is not clear if *C.* *priscus* will have priority over (for example) *C. kaiseni*, *Mojoceratops perifania*, or some combination of these.

Regardless, the holotype of "*C. priscus*", CMN 2280, is the only specimen referred to *C. russelli* which preserves the diagnostic posterior border of the parietal which also has stratigraphic data. It is therefore desirable to anchor any taxonomy to this specimen, and disregard more historical spcimens which lack data.

CMN 2280 was recovered from 667 m above sea level (Currie and Russell, 2005), estimated at 15.9 m above the Oldman - Dinosaur Park Formation contact (Mallon et al., 2012), and therefore ~21.1 m below a bentonite dated at 76.39 Ma that occurs 36 m above the formational contact, and ~21.4 m above a bentonite dated at 77.03 Ma which occurs 5.5m below Oldman-DPFm contact.

I therefore plot "*C. priscus*" occurring between 76.7 and 77.6 Ma

----

### Aff. *Chasmosaurus* sp.

**Aff. *Chasmosaurus* sp. (Fowler et al., in prep)**

New material from the Judith River Fm of Montana suggests that a *Chasmosaurus*-like taxon is present in the Montanan equivalent to Unit 1 of the lower Oldman Fm (Fowler et al., in prep).

----

### *Triceratops prorsus*

**cf*. Triceratops prorsus***

**Upper Hell Creek Fm, MT**

The holotype of *T. prorsus* (YPM 1822) was collected from an unrecorded horizon within the Lance Fm of Wyoming (Hatcher et al., 1907). However Scannella et al. (2014) report that more recently collected specimens of this morphotype are present in the upper third of the Hell Creek Fm, Montana. Hence here I show cf. *T. prorsus* as restricted to the upper Hell Creek Fm only.

----

### *Triceratops* sp.2

***Triceratops* sp.2**

**Upper part of the middle Hell Creek Fm, MT**

Scannella et al. (2014) show that *Triceratops* specimens from the upper part of the middle third of the Hell Creek Fm exhibit a morphology intermediate between cf. *T*. *horridus* (lower part of the middle third) and *T*. *prorsus* (upper third). This morphotype is referred to here as *Triceratops* sp.2.

----

### *Triceratops horridus*

**cf**. ***T. horridus***

**Lower part of the middle Hell Creek Fm, MT**

The holotype of *T*. *horridus* (YPM 1820) was collected from an unrecorded horizon within the Lance Fm of Wyoming (Hatcher et al., 1907). However Scannella et al. (2014) report that more recently collected specimens of this morphotype are present in the lower part of the middle third of the Hell Creek Fm, Montana. Hence here I show cf. *T*. *horridus* as restricted to the lower part of the middle Hell Creek Fm only.

----

### *Triceratops* sp.1

***Triceratops* sp.1**

**Lower Hell Creek Fm, MT**

*Triceratops* specimens collected from the lower third of the Hell Creek Fm are probably not referable to *T. horridus* (Scannella et al., 2014), and are here designated *Triceratops* sp.1 pending further research.

----

### *Ojoceratops fowleri*

***Ojoceratops* *fowleri* (Sullivan & Lucas, 2010)**

**Naashoibito Mbr, Ojo Alamo Fm, NM**

Holotype SMP VP-1865 is a complete left squamosal; associated and isolated material referred to cf. *O. fowleri* includes squamosal and parietal material, premaxillae, occipital condyle, rostral, nasal, and a predentary (Sullivan & Lucas, 2010).

Material referred to *O. fowleri* has been recovered mostly from the lower half of the Naashoibito Mbr of the Ojo Alamo Fm, NM (Sullivan & Lucas, 2010). Although the material is undoubtedly from a narrow stratigraphic interval, the taxon is here plotted as a large unknown range as the stratigraphic position of the Naashoibito Mbr is not well constrained.

----

### *Eotriceratops xerinsularis*

***Eotriceratops xerinsularis*** **(Wu et al., 2007)**

**Carbon Mbr, Horseshoe Canyon Fm, Alberta, CAN**

The only specimen is the holotype TMP 2002.57.7, which was collected from 13.5m above the base of the 20-25m thick Carbon Mbr (previously Unit 5), in between coal zones 11 and 12: the site is 9m above the base of coal zone 11, and 6m below the base of coal zone 12 (Wu et al., 2007; Eberth et al., 2013).

----

### Laramie *'Triceratops'*

**Laramie *'Triceratops'***

**Laramie Fm, CO**

Carpenter & Young (2002) illustrate DMNH 48617, a *Triceratops* skull collected from the Laramie Fm of Colorado. The specimen is not yet formally described, but is important as it is stratigraphically older than most other specimens attributed to *Triceratops*. DMNH 48617 exhibits a small nasal horn as expected based on the work of Scannella et al. (2014).

The range shown here is the same as the Laramie Fm itself as no further stratigraphic details are as yet published.

----

"*Torosaurus*" *utahensis*

**"*Torosaurus*" *utahensis* (Gilmore, 1946b)**

**North Horn Fm, UT; possibly Javelina Fm, TX**

The holotype (USNM 15583) comprises a partial skull (lacrimal, postorbital horn, jugal, epijugal, quadratojugal, squamosal) collected from the North Horn Fm, Utah (Gilmore, 1946b; Sullivan et al., 2005a). Referred material removed from the holotype are an incomplete posterior parietal (USNM 494472), and 13 frill epiossifications (USNM 494473); the paratype (USNM 15875) comprises a right squamosal and partial parietal (Sullivan et al., 2005a). Additional referred material from the North Horn Fm includes posterior parietal material, surangular, dentaries, fragmentary maxillae, premaxillae, and pterygoids, and various postcrania (Sullivan et al., 2005a).

Various material has also been referred to *T. utahensis* from the Kirtland and Ojo Alamo Fms, NM, the Javelina Fm, TX, and the Frenchman Fm, Saskatchewan. New Mexico material was reassigned to other taxa (Sullivan et al., 2005a; Sullivan & Lucas, 2010). The Saskatchewan material remains enigmatic and may be pathological. Texas material (e.g. Lawson, 1976; Hunt & Lehman, 2008) is included here as possible *T. utahensis* material.

Here I maintain *T. utahensis* as a defined taxon. However, with the realization of significant change through ontogeny in triceratopsin ceratopsids (Scannella & Horner, 2010), and that the holotype and much of the referred material is either fragmentary or immature, it would not be surprising if the type and referred material required rediagnosis, especially as the stratigraphic range of this material is poorly understood.

Material from the North Horn Fm was collected from the lower third of subunit 1B (Difley & Ekdale, 2002). Material from the Javelina Fm, TX is mostly concentrated in the middle of the unit, within 15 m above or below the 69 Ma datum (Wick & Lehman, 2013). Here I show a relatively restricted possible range, based on the proximity of the Javelina material to the 69 Ma datum, although it is possible that this may legitimately be expanded.

----

### *Coahuilaceratops magnacuerna*

***Coahuilaceratops* *magnacuerna* (Loewen et al., 2010)**

**Cerro del Pueblo Fm, Coahuila, MEX**

Holotype CPC 276 comprises a disarticulated skull including the rostral, premaxilla, maxilla, nasals, postorbital horns, anterior parts of the parietosquamosal frill, both dentaries, and unprepared postcrania (Loewen et al., 2010). Referred specimen CPC 277 comprises a predentary, dentary, and unprepared postcrania.

The holotype was collected from the middle facies of the Cerro del Pueblo Fm, ~230m from the base of the formation. Here I show the possible range of the taxon as the full duration of the Cerro del Pueblo Fm.

----

### *Arrhinoceratops brachyops*

***Arrhinoceratops* *brachyops* (Parks, 1925; Mallon et al., 2014)**

**Morrin Mbr, Horseshoe Canyon Fm, Alberta, CAN**

Known from two good skulls, the holotype, ROM 796 (complete cranium; Parks, 1925; Tyson, 1981) and ROM 1439 (nearly complete skull with associated postcranium; Mallon et al., 2014) from the Horseshoe Canyon Fm ("Edmonton Fm" in Parks, 1925). Both skulls are relatively small.

A number of other partial skull specimens have been attributed to *Arrhinoceratops*. Mallon et al. (2014) include ROM 1439, and WL 123 (CMN), both from the Horseshoe Canyon Fm. Eberth et al. (2013) include five "questionable" occurrences, three of which were excluded from the *Arrhinoceratops* hypodigm by Mallon et al. (2014) as they were considered either undiagnostic beyond Chasmosaurinae sp. (TMP 1983.012.0010; 1998.085.0001) or are more likely to pertain to *Anchiceratops* (CMS 3-1924, CMN).

**Stratigraphy**

Mallon et al. (2014) shows the two good skulls (holotype ROM 796, and ROM 1439) as occurring in the upper part of the Horsethief Mbr. Eberth et al. (2013) states that ROM 796 is from the lower portion of the Morrin Mbr, below the Drumheller Marine Tongue. Tanke (2010) states that if the original elevation data is correct, thenROM 796 derives from 15ft above coal #9 of the Horseshoe Canyon Fm, and "below the oyster bed" (ie. in the base of the Morrin Mbr, agreeing with Eberth et al., 2013).

Here I have plotted the lowermost occurrence as ROM 1439, occurring imediately below coal zone #8-9, and the uppermost occurrence as ROM 796, occurring immediately above coal #9.

----

### Almond Fm chasmosaurine

**?*Arrhinoceratops* sp.? (Farke, 2004)**

**Almond Fm, WY, USA**

Farke (2004) describes some unassociated chasmosaurine remains consisting of one indeterminable frill fragment (AMNH 3656) and a crushed but reasonably complete anterior half of a skull, sadly lacking any remains of the frill (AMNH 3652).

The Almond Fm chasmosaurine is morphologically closest to *Arrhinoceratops*, although still has similarities with *Anchiceratops*.

**On stratigraphy of Almond Fm fossils:**

"The low stratigraphic position of fossils within the formation suggests that they are probably Late Campanian in age"

This stratigraphic assertion correlates well with Farke's diagnosis that the skull most closely resembles either Arrhinoceratops or Anchiceratops. Here the Almond Fm chasmosaurine is plotted as the same range as the Almond Fm.

----

### *Medusaceratops lokii*

***Medusaceratops* *lokii* (Ryan et al., 2010a)**

**Oldman Fm, Alberta, CAN**

Holotype specimen is WDC-DJR-001, a partial parietal; paratype is WDC-DJR-002, another partial parietal (Ryan et al., 2010a). Unusually, these specimens are not held in a public repository and are instead present at the commercial Wyoming Dinosaur Center"; this partly reflects the original commercial nature of these specimens, the history of which can be found in Ryan et al. (2010a).

The holotype material of *Medusaceratops* was originally referred to *Albertaceratops* (Ryan, 2007), only later was it transferred to its own taxon (*Medusaceratops* *lokii*; Ryan et al., 2010a) on the basis of slight differences in the shape of the epiparietals. Moreover, despite their morphological similarity, *Medusaceratops* was proposed to represent a basal chasmosaurine, whereas *Albertaceratops* is a basal centrosaurine. Here I have plotted *Medusaceratops* is plotted as as a separate taxon from *Albertaceratops*, but this distinction and *Medusaceratops'* position within Chasmosaurinae is unconvincing. the phylogenetic position of *Medusaceratops* is possibly influenced by the differing system of epiparietal numbering used between the specimens.

**Stratigraphy**

The Mansfield bonebed, from which *Medusaceratops* derives, is neither figured by Ryan et al. (2010a), nor its stratigraphic position within the Judith River Fm explained or measured. It is understood through personal experience at Kennedy Coulee, MT that the Mansfield bonebed probably occurs within the mudstones that overlie the Herronton Sandstone equivalent present in this area. Although this remains to be confirmed by detailed stratigraphic analysis of the bonebed, it is what is assumed here when plotting its position, and is consistent with the limited data provided in Ryan et al. (2010a).

The stratigraphic range of *Medusaceratops* is constrained by radiometric dates from sections of the Judith River Fm in Montana which are stratigraphically equivalent to the Foremost and Oldman Fms of Alberta. An underlying radiometric date of 79.52 Ma (Goodwin & Deino, 1989; recalibrated here) occurs 4.8m below the top of the marker A coal in the Judith River Fm of northern Montana. An overlying radiometric date of 79.22 Ma (Goodwin & Deino, 1989; recalibrated here) occurs 27m above the top of the Marker A coal bed., ina mudstone unit which overlies the Herronton Sandstone (which itself overlies the Marker A / taber coal zone). As the *Medusaceratops* locality (Mansfield bonebed) is assumed here to also occur in the mudstone unit, then it is likely to be closer in age to 79.22 Ma rather than 79.52 Ma, Hence, here *Medusaceratops* is shown as occurring at 79.3 Ma.

----

### *Regaliceratops peterhewsi*

***Regaliceratops* *peterhewsi* (Brown & Henderson, 2015)**

**St Mary River Fm, Alberta, CAN**

The only specimen is the holotype TMP 2005.055.0001, a nearly complete skull missing only the rostral and lower jaws (Brown & Henderson, 2015).

The holotype was collected from the uppermost 30 m of the St Mary River Fm (Brown & Henderson, 2015). Host matrix of the holotype yielded the palynomorph *Scollardia* *trapaformis*, which is otherwise known only to range from the Carbon Mbr of the Horseshoe Canyon Fm through to the Battle Fm (Braman & Sweet, 1999; Brown & Henderson, 2015). The Kneehills Tuff (which occurs elsewhere within the Battle Fm, and has been radiometrically dated in other sections) also forms the upper bound of the St Mary River Fm.

Thus here I have plotted the possible occurrence of *Regaliceratops* as from the base of the Carbon Mbr of the Horseshoe Canyon Fm, through to the dated Kneehills Tuff horizon (69.97 Ma) from the middle of the Battle Fm.

----

### *Anchiceratops ornatus*

***Anchiceratops* *ornatus* (Brown, 1914a) syn A. longirostris (Sternberg, 1929)**

**Uppermost Horsethief to lowermost Tolman Mbrs, Horseshoe Canyon Fm, Alberta**

Mallon et al. (2011) and Eberth et al. (2013) give slightly differing accounts of the stratigraphic range of *Anchiceratops*, mainly due to Mallon et al. (2011) considering some specimens as generically indeterminate.

Eberth et al. (2013) state that the lowest specimen is CMN 10681, from the base of the Horsethief Mbr, just above coal #7. Mallon et al. (2011) state that this specimen is generically indeterminate, as its cranial material comprises only premaxillae and a rostral. As such it is not plotted here as the lowermost occurrence of *Anchiceratops*, but is instead plotted as Chasmosaurinae indet. (see individual entry).

The next lowest specimens are five skulls collected between coal seams #8 and #9 at the top of the Horsethief Mbr (Mallon et al., 2011; Eberth et al., 2013). These are plotted here as the lowermost occurrence of *Anchiceratops*.

A number of specimens (including the holotypes of *A. ornatus*; AMNH 5251; and *A. longirostris*; CMN 8535) are recorded from the uppermost Morrin or lowermost Tolman Mbrs (Mallon et al., 2011; Eberth et al., 2013). These represent the highest specimens for which either diagnostic material is known (ie. the posterior end of a parietal), or for which good stratigraphic data is available.

The highest specimens attributed to *Anchiceratops* are TMP 1983.001.0001 and possibly CMN 12-1915. TMP 1983.001.0001 is a mostly complete, small-sized skull that unfortunately is missing the posterior part of the frill (Mallon et al., 2011). It was collected from the basal sandstone (or just above) of the Tolman Mbr. CMN 12-1915 comprises the lateral border of a right squamosal and perhaps a small part of adjacent parietal, associated with large triangular frill epiossifications. The stratigraphic position of CMN 12-1915 is imprecisely known (which might be why it was not mentioned by Eberth et al., 2013; or maybe the specimen number was changed), but Mallon et al. (2011) assessed the scant locality data and concluded that it probably came from unit 4 (now called the Tolman Mbr; Eberth & Braman, 2012).

I have taken a conservative approach and plotted the range of *Anchiceratops* to be between coal seam #8 (uppermost Horsethief Mbr) up to the lowermost part of the Tolman Mbr.

***Anchiceratops* from the St. Mary River Fm?**

Langston (1975) describes fragmentary material from the St. Mary River Fm, Alberta, which he assigns to *Anchiceratops*. This includes a piece of frill bearing a large "almost equilateral" [triangular] epoccipital (NMC (CMN) 9829), and two postorbital horncore fragments (NMC (CM) 10645, 9590), the first of which bears a hollow in its base (which from the description sounds like a cornual sinus). None of the specimens are figured, and I have not seen them personally. The specimens were surface collected in the area of badlands called "Scabby Butte" (~27 km NNW of Lethbridge, Alberta; Langston, 1975). Maximum relief of Scabby Butte exposures is ~30 m (Langston, 1976), and the locality is as a whole considered as low in the St Mary River Fm (Brinkman, 2003). It is therefore possible that the fragmentary material overlaps stratigraphically with *Anchiceratops*, or may be slightly older.

***Anchiceratops* from the Dinosaur Park Fm?**

Langston (1959) describes some frill fragments from the uppermost Dinosaur Park Fm, Alberta, and suggests they have affinity with *Anchiceratops*. Later, Longrich (2014) redescribed these fragments as a new taxon, *Pentaceratops* *aquilonius*. These fragments overlap stratigraphically with the occurrence of *Utahceratops* *gettyi*, and are not considered here to represent *Anchiceratops*; see *Utahceratops* note for discussion.

----

### Chasmosaurinae indet.

**Chasmosaurinae indet. (aff. *Anchiceratops*; Mallon et al., 2011; Eberth et al., 2013)**

**Lowermost Horsethief Mbr, Horseshoe Canyon Fm, Alberta**

Eberth et al. (2013) state that the lowest specimen of *Anchiceratops* is CMN 10681, from the base of the Horsethief Mbr, just above coal #7. Mallon et al. (2011) state that this specimen is generically indeterminate, as its cranial material comprises only premaxillae and a rostral. As such it is not plotted here as the lowermost occurrence of *Anchiceratops*, but is instead plotted here as Chasmosaurinae indet..

However, in chasmosaurines the premaxilla is observed to undergo evolutionary change through as little as a million years (e.g. Scannella et al., 2014), and notably undergoes subtle change over ~1.5 m.y. in chasmosaurines of the Fruitland and Kirtland Fms, NM (Fowler, pers. obs.). With this in mind, it is possible that with further study CMN 10681 may be diagnostic, although caution is advised as the premaxilla also undergoes significant change through ontogeny (Scannella et al., 2014).

----

aff. *Anchiceratops*

**aff. *Anchiceratops* (Mallon et al., 2011; Eberth et al., 2013)**

**Middle Drumheller Mbr, Horseshoe Canyon Fm, Alberta**

Eberth et al. (2013) state that TMP.2008.035.0001 was collected from the Drumheller Mbr, 3 m above coal #5. Mallon et al. (2011) state that this specimen is currently unprepared but comprises a parietal frill with large triangular epiossifications, and so provisionally assign it to *Anchiceratops*.

Pending a detailed description of TMP.2008.035.0001, here I have taken a conservative approach, assigning it to aff. *Anchiceratops*; given the ~1.5 m.y. age difference between TMP.2008.035.0001 and other definitive *Anchiceratops* specimens, and that over similar length of time morphological change is observed in other chasmosaurine taxa (Sampson & Loewen, 2010; Mallon et al., 2012; Scannella et al., 2014; Fowler, in prep).

----

### Kirtland Taxon C

**Kirtland Taxon C (Fowler, 2016)**

**De-na-zin Mbr, Kirtland Fm, NM, USA**

Limited chasmosaurine remains are known from the De-na-zin Mbr, the uppermost member of the Kirtland Fm, NM. However, some of the material is morphologically distinct from, but forms morphological intermediates between, preceding and succeeding forms such as *Pentaceratops* and *Anchiceratops* (Fowler, in prep).

A reasonable amount of material is known but it is all fragmentary and diagnostic elements are few. The most important specimen is NMMNH P33906, which comprises little more than a few skull fragments, but critically includes some pieces of the diagnostic parietal median longitudinal bar (Fowler, 2016). Other material includes an associated brow horn and squamosal (NMMNH 21098; Lehman, 1993); a small, probably juvenile, partial squamosal and parietal (NMMNH 25084; Lehman, 1980; Rowe et al., 1980), and other as yet undescribed material. These fragments are not complete enough to diagnose a new taxon with any confidence.

**Stratigraphy**

Here I have plotted the position of the parietal fragment NMMNH P33906 as it is the most diagnostic element. The precise stratigraphic position has not been measured in the field, but the locality coordinates (on file at NMMNH; see Fowler, in 2016) plot in the middle of the Denazin Mbr, between ash dates 73.83 Ma (<5 m above base of Mbr) and 73.49 Ma (4.9 m below top of Mbr; dates recalibrated by Fowler, this article; originally from Fassett & Steiner, 1997; see individual notes).

----

### Kirtland Taxon B

**Kirtland Taxon B (Fowler, 2016)**

**Hunter Wash Mbr, Kirtland Fm, NM, USA**

Holotype is a fragmentary skull (NMMNH 27468), which includes a nearly complete parietal that preserves the diagnostic posterior edge (Fowler, 2016). Taxon B is diagnosed by a deeper midline embayment than seen in preceding chasmosaurines *Utahceratops*, *Pentaceratops*, and Taxon A; the posterior lateral rami are also much thicker antero-posteriorly, and the fenestrae are probably slightly reduced in size. In these respects, Taxon B is transitional between preceding chasmosaurines and the succeeding *Anchiceratops* (Fowler, 2016).

**Stratigraphy**

NMMNH 27468 was recovered from the middle part of the Hunter Wash Mbr of the Kirtland Fm (Fowler, 2016). The precise stratigraphic position has not been measured in the field, but the locality coordinates (on file at NMMNH; see Fowler, 2016) plot ~80 m above the base of the Hunter Wash Mbr. Age of the Hunter Wash Mbr is constrained by two ash horizons dated at 75.02 Ma (136 m above the base of the underlying Fruitland Fm, low to middle part of the Hunter Wash Mbr, Kirtland Fm; see individual note for discussion) and 74.57 Ma (181 m above the base of the underlying Fruitland Fm, upper part of the Hunter Wash Mbr; dates recalibrated by Fowler, this article; originally from Fassett & Steiner, 1997). Taxon B falls ~40 m below the 74.57 Ma horizon.

Taxon B is therefore plotted from 74.5 to 74.6 Ma, reflecting the position of the ash in the Hunter Wash Mbr. It is probable that this positioning may move up or down by 0.1 Ma when the stratigraphic position is better constrained.

**Further material**

An undescribed chasmosaurine skull (NMMNH P50000) was also recovered from high in the Hunter Wash Mbr, and may pertain to Taxon B. However, unfortunately NMMNH P50000 lacks the posterior midline of the parietal, hence comparisons to the holotype cannot be made. A new specimen comprising an immature skull and postcranium was also recovered from the upper part of the Hunter Wash Mbr, and is currently under study (Cantrell et al., 2014).

----

### Kirtland Taxon A

**Kirtland Taxon A (Fowler, 2016)**

**Hunter Wash Mbr, Kirtland Fm, NM, USA**

Holotype is a fragmentary skull (SMP VP 1500), which includes a nearly complete parietal that preserves the diagnostic posterior edge (Fowler, 2016). Taxon A is diagnosed by a deeper U-shaped midline embayment than seen in preceding chasmosaurines *Utahceratops* and *Pentaceratops*, but less so than in the superseding Taxon B; the posterior lateral rami are also slightly thicker antero-posteriorly, and meet at a more acute angle than in preceding taxa *Utahceratops* and *Pentaceratops*. In these respects, Taxon A is transitional between preceding chasmosaurines and the succeeding Taxon B, and *Anchiceratops* (Fowler, 2016).

**Stratigraphy**

SMP VP1500 was recovered from the lower part of the Hunter Wash Mbr of the Kirtland Fm (Fowler, 2016). The precise stratigraphic position has not been measured in the field, but the precise GPS coordinates (see Fowler, 2016) show that it occurs upper ~9 m (30 ft) above a prominent coal horizon that is considered by some workers to represent the boundary between the Fruitland and Kirtland Fms (see Fruitland & Kirtland Fms notes). Age of the Hunter Wash Mbr is constrained by two ash horizons dated at 75.02 Ma (136 m above the base of the underlying Fruitland Fm, low to middle part of the Hunter Wash Mbr, Kirtland Fm; see individual note for discussion) and 74.57 Ma (181 m above the base of the underlying Fruitland Fm, upper part of the Hunter Wash Mbr; dates recalibrated by Fowler, this article; originally from Fassett & Steiner, 1997).

The position of SMP VP1500 is therefore plotted here as 75.0 - 75.1 Ma, one cell below the lowermost radiometric date.

**Further material**

Fossil material from chasmosaurinae or ceratopsidae indet. is relatively common in the lower part of the Kirtland Fm, such that it is likely that more material from Taxon A currently exists in collections. However, since the most diagnostic element is the posterior border of the parietal, then most currently known material is not technically referable.

Finally, an important issue exists in that the stratigraphic position of the holotype and paratype of *Pentaceratops sterbergii* are not known. See note on *Pentaceratops* and Fowler (2016) for discussion and resolution of this issue.

----

### aff. *Pentaceratops* n.sp.

**aff. *Pentaceratops* n.sp. (Fowler, 2016)**

**Fruitland Fm, NM, USA**

Fowler (2016) removes a number of specimens from *Pentaceratops* *sternbergii* and moves them into aff. *Pentaceratops* n.sp., along with newly described material (MNA Pl.1747, complete skull and partial postcranium; UKVP 16100, complete skull; NMMNH P-37880, partial right lateral ramus of parietal posterior bar). The new taxon differs from P. sternbergii in position of the central embayment of the parietal relative to various frill epiossification loci, related to the overall stratigraphic trend of a deepening embayment (Fowler, 2016).

Both specimens were collected from the uppermost part of the Fruitland Fm, with MNA Pl. 1747 recovered ~145 m above the base of the formation (Rowe et al., 1980). Plotting of the locality data on Google earth and comparing with USGS geological maps confirms this position (see Fowler, 2016). However, because the resolution of the chart is not sufficient to separate these from the upper Fruitland Fm *P. sternbergii*, I have instead plotted aff. Pentaceratops n.sp as the lowermost part of the Kirtland Fm, thereby separating it from the probably preceding *P. sternbergii*, and superseding Taxon A (see individual entries).

----

### *Pentaceratops sternbergii*

***Pentaceratops* *sternbergii* (Osborn, 1923)**

**Fruitland Fm, NM, USA**

The taxon *Pentaceratops* *sternbergii* presents a number of problems related to the inadequacy of the holotype and associated locality data, and the definition of the Fruitland-Kirtland formational boundary. These are resolved in Fowler (2016) where the original hypodigm of *Pentaceratops* *sternbergii* is conserved with other specimens attributed to other taxa (Taxon A, B, C, and aff *Pentaceratops* n.sp.; see individual entries). Under this revision material referred to *Pentaceratops* *sternbergii* is restricted to the holotype (AMNH 6325, nearly complete skull, missing the mandible and the posterior half of the parietal and squamosals; Osborn, 1923), and other specimens that formed the original hypodigm of Hatcher et al. (1907; AMNH 1624, nearly complete skull, missing mandible and the medial part of the parietal; AMNH 1625, nearly complete frill, missing anterior end of the parietal and right squamosal, and most of the left squamosal. Referred to as cf. *Pentaceratops* *sternbergii*.

AMNH 6325, 1624, and 1625 were all collected by C. H. Sternberg in 1922 and 1923 from the Fruitland Formation, San Juan Basin, New Mexico (See Fowler, in prep). The precise stratigraphic position of these quarries cannot be ascertained, but should be within the Fruitland Fm. Here I have plotted the occurrence of *P. sternbergii* as the uppermost part of the Fruitland Fm. Note that the related taxon aff. *Pentaceratops* n.sp. occurs immediately above *P. sternbergii* in the uppermost Fruitland, probably at a higher resolution than can be shown in the chart, hence it is shown as the lowermost Kirtland Fm.

----

### *Utahceratops gettyi*

***Utahceratops* *gettyi* (Sampson et al., 2009)**

**Kaiparowits Fm, UT**

Material attributed to *Utahceratops* *gettyi* has been recovered from seven localities in the lower to middle Kaiparowits Fm (including two multi individual bonebeds; Sampson et al., 2010; Loewen et al., 2013b). There are potential complications in that some of the characters that diagnose *Utahceratops* *gettyi* are not observable in all referred specimens. This is especially prevalent in the stratigraphically lowest specimens, some of which overlap stratigraphically with other named chasmosaurine taxa from the Dinosaur Park Fm, CAN. More detailed discussion can be found below, and in Fowler (2016).

**Stratigraphy**

The stratigraphic position of specimens referred to *U. gettyi* were plotted by Loewen et al. (2013b) relative to a series of radiometrically dated bentonites, the precise positions and ages of which are most recently summarized in Roberts et al. (2013). Referred specimens have been recovered from a stratigraphic range of ~225 m in the lower to middle Kaiparowits Fm.

The stratigraphic position of the holotype specimen (UMNH VP 16784, locality UMNH 960) is shown by Roberts et al. (2013) as occurring in the lower part of the middle unit of the Kaiparowits Fm, slightly above an ash dated at 75.97 Ma (the distance in meters is unclear); however the same locality is shown by Loewen et al. (2013b) as occurring ~175m above the base of the Kaiparowits Fm, slightly below the same radiometric date. The most diagnostic specimens derive from a bonebed ~105m higher than the holotype in the middle Kaiparowits Fm, ~280m above the base (locality UMNH 942; Sampson et al., 2010). This material includes a well preserved posterior parietal midline (UMNH 16671) and premaxilla (UMNH VP 16672; Sampson et al., 2010). The highest referred specimen, UMNH VP 12198 (locality UMNH 145; Loewen et al., 2013b), was collected in the middle Kaiparowits Fm, ~330m from the base, ~125 m below ash KBC-109 (75.51 Ma; Roberts et al., 2013). The lowermost referred specimen is UMNH VP 16758 which occurs in the lower Kaiparowits Fm, ~100m above the base, and ~20m above ash KDR-5 (76.46 Ma; Roberts et al., 2013; Loewen et al., 2013b).

Here I have plotted the full range of the referred material, although a more conservative approach might only plot the horizon from which the most diagnostic posterior parietal midline (UMNH 16671) was recovered.

**Dinosaur Park Fm, CAN: "*Chasmosaurus*" *russelli*, and "*Pentaceratops* *aquilonius*"**

It is possible that two chasmosaurine taxa named from the upper part of the Dinosaur Park Fm, Canada, might be conspecific with *Utahceratops* *gettyi*.

The holotype specimen of *Chasmosaurus* *russelli* (NMC 8800; a complete skull) was collected from high in the Dinosaur Park Fm (bound by radiometric dates of 76.39 Ma, middle Dinosaur Park Fm, and 76.10 Ma, uppermost Dinosaur Park Fm) from an area outside of Dinosaur Provincial Park itself (Sternberg, 1940; recently restudied with greater resolution by Campbell et al., 2013). The lowermost specimens attributed to *Utahceratops* (UMNH VP 12225, 16758; Loewen et al., 2013b) occur ~20 m above an ash date of 76.46 Ma and thus may potentially overlap stratigraphically with the *C. russelli* holotype.

Similarly, "*Pentaceratops* *aquilonius*" was recently described based on two parietosquamosal frill fragments from the uppermost Dinosaur Park Formation, within the Lethbridge coal zone (Longrich, 2014; previously referred to *Anchiceratops*; Langston, 1959). The radiometric date of 76.10 Ma for the lower part of the Lethbridge Coal Zone suggests that *P. aquilonius* may stratigraphically overlap the four oldest occurrences of *Utahceratops* (including the holotype). However, "*P. aquilonius*" is very scrappy, and not considered diagnostic by Fowler (2016), although it is clearly a member of the *Utahceratops* lineage, rather than the *Chasmosaurus* lineage, the latter of which is typically recovered from the Dinosaur Park Fm.

It is possible therefore that "*Chasmosaurus*" *russelli* or "*Pentaceratops* *aquilonius*" may pertain to the same taxon as the stratigraphically lower examples of *Utahceratops*. Here though, I do not plot "*P. aquilonius*", and "*Chasmosaurus*" *russelli* is considered as separate pending further research.

----

### *"Chasmosaurus" russelli*

**"*Chasmosaurus*" *russelli* (Sternberg, 1940; Campbell, 2016)**

**Dinosaur Park Fm, CAN**

Holotype specimen CMN 8800 (was GSC 8800; Sternberg, 1940) comprises a complete skull. A number of specimens have been referred to *C. russelli* since description of the holotype, but all of these have recently been separated from *C. russelli* in various papers by N. Longrich (2010; 2015). Some of this taxonomy is discussed by Fowler (2016), and is beyond the scope of this chart note. However, Fowler (2016) suggests that "*Chasmosaurus*" *russelli* be moved within the *Utahceratops* lineage as many characters of the posterior frill are unlike that of the *Chasmosaurus* lineage, and much closer to *Utahceratops* (etc).

The holotype of *C. russelli* was collected from high in the Dinosaur Park Fm from an area outside of Dinosaur Provincial Park itself (Sternberg, 1940; recently restudied with greater resolution by Campbell et al., 2016). Campbell et al. (2016), state that it was collected from the top of the Dinosaur Park Formation, immediately below the Lethbridge Coal Zone, in the same stratigraphic interval as *Chasmosaurus* ("*Vagaceratops*") *irvinensis*.

Hence here it is plotted as the highest level within the Dinosaur Park Fm, immediately below the Lethbridge Coal Zone.

----

### *Spiclypeus shipporum*

***Spiclypeus* *shipporum* (Mallon et al, 2016)**

**Judith River Fm, MT**

Holotype specimen CMN 57081 is a partial skull and postcranium comprising a premaxilla and rostral, possible nasal horn, brow horns, partial maxilla, jugals, dentaries, squamosals, and the posterior border of the parietal.

**Stratigraphy**

The holotype of *Spiclypeus* was collected from the Judith River Fm ~8km WSW of Winifred in central Montana (Section 1, Township 20N, Range 17E; Mallon et al., 2016). Mallon et al (2016) state that this is in the lower part of the Coal Ridge Member of the Judith River Formation (sensu Rogers et al., 2016). However, no measured section is given (other than 11 m of sedimentary log taken at the immediate site of the fossil quarry).

Reference to Google Earth and published type sections (Rogers et al., 2016) suggests that this stratigraphic placement needs to be reassessed. Rogers et al published a measured section (92-JRT-15) ~20km NNW of the *Spiclypeus* quarry. In the published photo, measured section (Rogers et al., 2016) and Google Earth image, it can be clearly seen that at 92-JRT-15 the cliffs comprise a basal grey-brown shale (Claggett Shale) a pale sandstone (Parkman Sandstone Mbr, Judith River Fm) capped by a yellow channel sand (McClelland Ferry Mbr, Judith River Fm). These distinctive horizons can be followed south along near continuous outcrop exposed on the east bank river cliffs of the Judith River (the actual river) until we reach the mouth of Anderson Coulee, immediately west of the *Spiclypeus* locality (Section 1, Township 20N, Range 17E; Mallon et al., 2016). Here the succession in the cliffs again appears to represent the Claggett, Parkman, and McClelland Ferry Members as in the measured section 92-JRT-15 (Rogers et al, 2016). Reference to topographic maps shows that the *Spiclypeus* locality is not more than 200 feet (60 m) higher than the top of the cliffs at the mouth of Anderson Coulee. Thus it is possible that *Spiclypeus* occurs within the McClelland Ferry Mbr of the Judith River Fm.

Precise positioning of the quarry should be possible by measuring a section along Anderson Coulee from the basal contacts of the Judith River Fm up to the fossil locality. Here I have plotted the possible range of *Spiclypeus* as from the base of the upper Oldman Fm (possible correlation to the maximum regressive surface of the Judith River Fm) through to 76 Ma, the approximate age of the base of the Coal Ridge Mbr of the Judith River Fm based on a radiometric date from the top of the McLelland Ferry Mbr (Rogers et al, 2016; although these radiometric dates may have problems; see main entry). However it would not be surprising if the position of *Spiclypeus* might correlate with the lower part of the Oldman Fm, depending on correlation of the type Judith with sections in southern Alberta; as stated elsewhere, there are problems in correlating the newly described type section with subunits of the Judith River Formation in Northern Montana, and the Belly River Group in Alberta.

Stratigraphic position of *Spiclypeus* is very important in relation to other Campanian chasmosaurines to which it may be related. It is therefore critical to have a measured section for the specimen. Placement here is thus considered provisional.

---

### Aff. *Agujaceratops* *mariscalensis*

**Aff. *Agujaceratops* *mariscalensis* (Forster et al., 1993)**

**Ajuga Fm, TX, USA**

Forster et al. (1993) describe a near-complete chasmosaurine skull (TMM 43098-1) collected 50m above the base of the Upper Shale Mbr of the Aguja Fm, TX, which they attribute to "*Chasmosaurus*" (*Agujaceratops*) *mariscalensis*. However, the specimen lacks the diagnostic midline of the parietal, and cannot be reliably referred to *Agujaceratops* *mariscalensis* (Lehman, 1989; see Fowler, 2016), especially given that there is stratigraphic separation between the TMM 43098-1 and the holotypic material (ceratopsid taxa typically evolve rapidly, and as such the specimens might not be expected to be the same taxa). I am therefore being cautious and assigning TMM 43098-1 to aff. *Agujaceratops* *mariscalensis* (see Fowler, 2016. for more details).

**Stratigraphy**:

As has been shown elsewhere in this chart, the only diagnostic *Agujaceratops* material is derived from a single stratigraphic horizon ~20 m above the base of the Upper Shale Mbr (Lehman, 1989; Fowler, 2016), whereas the aff. *Agujaceratops* *mariscalensis* skull was collected ~50 m above the base of the Upper Shale Mbr. Hence we can say with confidence that aff. *A*. *mariscalensis* should occur above the type *A. mariscalensis* material, although age of the Upper Shale Mbr itself is poorly constrained (see individual entry). Here then I plot the positions of aff. *A. mariscalensis* and *A. mariscalensis* as adjacent cells in the position which I consider likely, but they may actually occur anywhere within the arrows, indicating the likely range of the Upper Shale Mbr of the Aguja Fm (see individual entry).

----

### *Agujaceratops mariscalensis*

***Agujaceratops* "*Chasmosaurus*" *mariscalensis*: (Lehman, 1989; Forster et al., 1993; Lucas & Sullivan, 2006)**

**Aguja Fm, TX, USA**

This taxon was originally described as *Chasmosaurus* *mariscalensis* (Lehman, 1989) but was transferred to the new genus, *Agujaceratops*, by Lucas & Sullivan (2006). There are a number of issues with *Agujaceratops* *mariscalensis*.

**Lack of a well preserved adult holotype**

The holotype material (Lehman, 1989) is almost all immature. The holotype specimen (UTEP P37.7.142) comprises a braincase, left supraorbital horncore, left maxilla, and right dentary. Also possibly attributable to the holotype are a right coracoid and right pubis (UTEP P37.7.7.142). The supraorbital horncore from the fragmentary holotype is approximately one third the length of the largest horncore also attributed to *A. mariscalensis* by Lehman (1989). Given that supraorbital horncores increase in size, both absolutely and relatively, into adulthood (e.g. Lehman, 1990a), it is probable that the holotype specimen is at most half adult size, and may not exhibit adult characters.

A nearly complete skull was described by Forster et al. (1993) and attributed to *Chasmosaurus* *mariscalensis*. However, here I remove it from this taxon and refer it instead to aff. *Agujaceratops* *mariscalensis* (see individual entry).

Stratigraphy, problems with attribution of undiagnostic material to *C. mariscalensis*

Most of the material in the type description by Lehman (1989) originates from the base of the Upper Mbr of the Aguja Fm, directly above the Terlingua Creek sst-McKinney Springs marine tongue (localities WPA1, WPA3, & TMM 42534). The time transgressive nature of the Upper Aguja / McKinney Springs contact means that the localities WPA1 & WPA3 are about 20m higher in the section than TMM 42534, and thus probably slightly younger.

However, some attributed material originates from two localities (TMM 41838 & 42303) that are stratigraphically distinct from WPA1, 3 & TMM 42534. Locality TMM 41838 is situated below the McKinney Springs marine tongue in the uppermost paralic beds of the Lower Aguja Mbr, (approximately 60m below WPA3). Locality TMM 42303 is positioned approximately 70m above the base of the Upper Aguja Mbr and 50m above WPA3. The 110m difference in height between these localities equates to as much as 50% of the total thickness of the Aguja Fm.

The locality from which material is derived is typically not explicitly mentioned by Lehman (1989), but specimen numbers seem to indicate provenance. Two small scapulae (TMM42534-19 & TMM42534-20) are the only specimens mentioned in the text that originate from locality TMM42534. However, ceratopsid postcrania are not considered diagnostic even to subfamilial level (Dodson & Currie, 1990). As such, no elements diagnosable as *C. mariscalensis* are known from TMM 42534. These scapulae should be referred to Ceratopsidae indet.

The lower locality (TMM 41838) yielded a skull fragment (TMM 41838-13), although it is not made clear exactly how much of the skull is represented, from the text it appears to comprise the postorbital, prefrontal, and perhaps a small part of the horncore. The morphology of TMM 41838-13 is stated to be slightly different from other prefrontals from the WPA1 &3 localities. If TMM 41838-13 contains a complete horncore then it may be considered diagnostic as *C. mariscalensis* (although this is debatable), but based on a lack of evidence provided, this specimen should be considered as ceratopsidae indet. The upper locality (TMM 42303) yielded a right scapulocoracoid (TMM 42303-1), which, as an undiagnostic element, should also be considered as Ceratopsidae indet.

Consequently, based on the apomorphies described by Lehman (1989), only the cranial material recovered from WPA1 & WPA3 can be reliably referred to *C. mariscalensis*. Thus *C. mariscalensis* can only be shown to have occurred within a very narrow stratigraphic horizon, since WPA1 & WPA3 are within 10m of each other stratigraphically. This is positioned very close to the base of the Upper Shale Mbr, and is very unlikely to be much younger than early Judithian, which would make *C. mariscalensis* the oldest named chasmosaurine by a margin of approximately 2-3my.

**Stratigraphy**

The only diagnostic *Agujaceratops* material is derived from a single stratigraphic horizon ~20 m above the base of the Upper Shale Mbr (Lehman, 1989; Fowler, in prep), whereas the aff. *Agujaceratops* *mariscalensis* skull was collected ~50 m above the base of the Upper Shale Mbr. Hence we can say with confidence that aff. *A. mariscalensis* should occur above the type *A. mariscalensis* material, although age of the Upper Shale Mbr itself is poorly constrained (see individual entry). Here then I plot the positions of aff. *A. mariscalensis* and *A. mariscalensis* as adjacent cells in the position which I consider likely, but they may actually occur anywhere within the arrows, indicating the likely range of the Upper Shale Mbr of the Aguja Fm (see individual entry).

----

### *Zuniceratops christopheri*

***Zuniceratops* *christopheri* (Wolfe & Kirkland, 1998)**

**Moreno Hill Fm, NM**

Holotype specimen MSM P2101 comprises the anterior half of a skull (Wolfe & Kirkland, 1998), although many additional specimens have been attributed to the taxon, including bonebed accumulations and immature material (Wolfe & Kirkland, 1998; Wolfe, 2000).

Although type and referred material are from slightly different stratigraphic positions, they are both considered by Wolfe & Kirkland (1998) to occur within the lower half of the Moreno Hill Fm, ~45 - 107 m above the formational base (total thickness 151 m). Hence here I show the occurrence of *Zuniceratops* as a single cell positioned in the middle of the Moreno Hill Fm (this is admittedly only approximate, but lack of taxa of similar age prevents any potential issues for the time being).

Note that this entry occurs within the chasmosaurinae column out of convenience: it is not implied that the basal ceratopsid *Zuniceratops* is a chasmosaurine.

----

## Centrosaurinae

### *Albertaceratops nesmoi*

***Albertaceratops* *nesmoi* (Ryan, 2007)**

**Oldman Fm, Alberta, CAN**

Holotype specimen is a nearly complete skull (TMP.2001.26.1) from the lower Oldman Fm, approximately 9m above the contact with the underlying Foremost Fm. (Ryan, 2007)

In the original description (Ryan, 2007), additional cranial material was referred to *Albertaceratops*, having been collected from the Mansfield bonebed (Judith River Fm, MT; stratigraphically equivalent to the type *Albertaceratops* quarry). This material was later moved into its own taxon, *Medusaceratops* *lokii* (Ryan et al., 2010a), on the basis of slight differences in the shape of the epiparietals. Moreover, despite their morphological similarity, *Medusaceratops* was proposed to represent a basal chasmosaurine, whereas *Albertaceratops* is a basal centrosaurine. Here I have plotted *Medusaceratops* is plotted as as a separate taxon from Albertaceratops, but this distinction and *Medusaceratops'* position within Chasmosaurinae is unconvincing. the phylogenetic position of *Medusaceratops* is possibly influenced by the differing system of epiparietal numbering used between the specimens.

**Stratigraphy**

The stratigraphic range of *Albertaceratops* nesmoi is constrained by radiometric dates from sections of the Judith River Fm in Montana which are stratigraphically equivalent to the Foremost and Oldman Fms of Alberta.

An underlying radiometric date of 79.52 Ma (Goodwin & Deino, 1989; recalibrated here) occurs 4.8m below the top of the marker A coal in the Judith River Fm of northern Montana, which is equivalent to the upper Taber Coal Zone of the Foremost Fm, Alberta. An overlying radiometric date of 79.22 Ma (Goodwin & Deino, 1989; recalibrated here) occurs 27m above the top of the Marker A coal bed, therefore ~18m above the *Albertaceratops* locality.

I have therefore shown *Albertaceratops* *nesmoi* here as occurring roughly in between these dates at 79.3 Ma.

----

### *Diabloceratops eatoni*

***Diabloceratops* *eatoni* (Kirkland and DeBlieux, 2010)**

**Wahweap Fm, UT, USA**

Holotype UMNH VP 16699, a skull preserving the entire left side, and some parts of the right side (Kirkland & DeBlieux, 2010). The specimen was known as the "last chance" skull in many publications prior to its full description.

Kirkland & DeBlieux (2010) refer a partial skull ("nipple butte ceratopsian") to *Diabloceratops* sp.. However, this specimen occurs in the uppermost part of the lower member ~50m stratigraphically lower than the holotype of *D. eatoni*, so it is shown as a separate unit here (see individual entry).

In what may be an additional specimen, Kirkland (2001) mentions a partial adult centrosaurine frill found with S. Sampson, M. Getty, D Powell, & C. Sorenson. The specimen was collected from "near the top of the Wahweap Fm". No morphological details are given.

Popular website discussion suggests the small-sized *Diabloceratops* *eatoni* may represent an immature individual (and therefore a junior synonym) of *Albertaceratops* nesmoi (Oldman Fm, CAN) with which it shares many anatomical features and may be contemporaneous. However, this hypothesis has not been officially published, and the taxa are considered separate here.

**Stratigraphy**

Kirkland & DeBleiux (2010) state that the holotype was recovered from a sandstone "near the middle of the middle mudstone Mbr, 51.72m above its base, and 105.30m above the contact ...with the underlying Drip Tank Mbr of the Straight Cliffs Fm".

A radiometric date of 79.9 Ma is recorded ~60 m above the base of the Wahweap Fm. As the *Diabloceratops* holotype quarry occurs at ~105m above the basal contact of the Wahweap Fm, then its age can be constrained as younger than 79.9 Ma. However an upper bound for the range of *Diabloceratops* is more difficult to ascertain as the only overlying chronostratigraphic indicators are the C33r-C33n bondary which occurs at 78.91 Ma 20 m below the top of the upper Wahweap Fm, and a radiometric date of 76.46 Ma from the uppermost part of the lower member of the overlying Kaiparowits Fm. It seems likely that the holotype of *Diabloceratops* is only a few hundred thousand years younger than the 79.9 Ma date, and that is what is shown here.

----

### "nipple butte" *Diabloceratops* sp.

**Ceratopsidae sp. "nipple butte ceratopsid" (Kirkland, 2005)**

**referred to *Diabloceratops* sp. (Kirkland & DeBlieux, 2010)**

**Wahweap Fm, UT, USA.**

UMNH VP 16704, the "nipple butte ceratopsid" is a fragmentary skull collected in 2000, comprising part of the right half of the frill, occipital condyle, braincase, jugal, orbital and antorbital region, palate, partial horncore, and fragmentary left and right pterygoids and maxillae (Kirkland & DeBlieux, 2010).

**Stratigraphy**

The nipple butte ceratopsid was collected from the uppermost sandstone bed of the lower sandstone member of the Wahweap Fm, ~50m above the contact with the underlying Drip Tank Mbr of the Straight Cliffs Fm (Kirkland & DeBlieux, 2010).

A radiometric date of 80.6 Ma occurs in the lower part of the middle member of the Wahweap Fm, although there is some inconsistency among authors regarding the precise position of this date (see individual entries; date recalibrated here). Regardless, the nipple butte ceratopsid occurs below the middle member, and so can be inferred to be older than 80.6 Ma, probably a few hundred thousand years older. However, a maximum age is only constrained by the age of the underlying unit, the Drip Tank, which is Upper Santonian, although it is expected that the age of the nipple butte ceratopsid is ~80.8 Ma (as shown here).

The specimen was described as a "long-horned centrosaurine" by Kirkland (2005), with the material described and figured by Kirkland & DeBlieux (2010), who refer it to *Diabloceratops* sp.. The type and only known specimen of *Diabloceratops* (see individual entry) was recovered from the middle of the middle member of the Wahweap Fm, ~50 m stratigraphically higher than the nipple butte skull. Given that ceratopsids are observed to evolve relatively rapidly, it might not be expected that the nipple butte specimen would be referable to *Diabloceratops*. As such, here I have plotted the nipple butte ceratopsian separate from *Diabloceratops* *eatoni*, which adds clarity.

Note that in their Fig. 21.14, Loewen et al. (2013) illustrate UMNH 16704 occurring in the lowermost part of the Middle Mbr, whereas in their text (p. 490) it is clearly described as occurring in the Lower Mbr, ~ 50m above the top of the Drip Tank Mbr of the Straight Cliffs Fm.

----

### Wahweap centrosaurine A

**Wahweap centrosaurine A (Loewen et al., 2013)**

**referred to Diabloceratops sp. (Kirkland & DeBlieux, 2010)**

**Wahweap Fm, UT, USA.**

UMNH VP 20600 comprises a partial braincase and a nearly complete parietosquamosal

frill (Loewen et al., 2013). Although unnamed, UMNH VP 20600 preserves enough diagnostic morphology to allow comparison to other centrosaurines. Loewen et al. (2013, p. 490) describe UMNH VP 20600 as:

"the parietals are relatively wide and fenestrate, with a large midline embayment, forming an M-shaped frill in dorsal view. The only epiossifications preserved on the frill are bilateral, laterally curving, dorsoventrally flattened hooks on either side of the embayment. These parietal ossifications differ in shape, length, and orientation from those of *Diabloceratops* *eatoni* (Kirkland and DeBlieux, 2010), a form recovered in the middle unit, and more closely resemble those of *Albertaceratops* *nesmoi* (Ryan and Russell, 2005)."

**Stratigraphy**

UMNH VP 20600 was recovered from the lower sandstone member of the Wahweap Fm, ~45m above the contact with the underlying Drip Tank Mbr of the Straight Cliffs Fm (Loewen et al., 2013).

A radiometric date of 80.6 Ma occurs in the lower part of the middle member of the Wahweap Fm, although there is some inconsistency among authors regarding the precise position of this date (see individual entries; date recalibrated here). Regardless, UMNH VP 20600 occurs below the middle member, and so can be inferred to be older than 80.6 Ma, probably a few hundred thousand years older given that it is from the upper part of the lower member. However, a maximum age is only constrained by the age of the underlying unit, the Drip Tank Mbr of the Straight Cliffs Fm, which is Upper Santonian.

UMNH VP 20600 occurs ~5m below the "Nipple Butte" ceratopsid (referred to *Diabloceratops* sp.; see individual entry), and is therefore plotted here in the cell immediately below.

----

### *Nasutocertops titusi*

***Nasutocertops* *titusi* (Sampson et al, 2013)**

**Kaiparowits Fm, UT, USA**

Holotype UMNH VP 16800 (nearly complete skull with partial postcrania), and referred specimens UMNH VP 19466 (disarticulated adult skull including a partial premaxilla, maxilla and nasal) and UMNH VP 19469, (squamosal) described by Sampson et al. (2013).

**Stratigraphy**

*Nasutoceratops* specimens have been recovered from the middle unit of the Kaiparowits Formation, between ~250–320 m above its base, and bound by ash dates of 75.51 Ma at 420 m, and 75.97 Ma at 190 m (Loewen et al., 2013b; Sampson et al., 2013; Roberts et al., 2013).

Hence here I show *Nasutoceratops* occurring between 75.8-75.6 Ma.

----

### *Avaceratops lammersi*

***Avaceratops* *lammersi* (Dodson, 1986; Penkalski & Dodson, 1999)**

**Judith River Fm, MT, USA**

The type (ANSP15800) is a juvenile partial skull & skeleton from Careless Creek Quarry, Judith River Fm, MT (Dodson, 1986). Redescibed by Penkalski & Dodson (1999), here they also attribute a much larger partial skull (MOR 692) to the genus.

**Stratigraphy, type specimen**

The stratigraphic horizon of the type locality, Careless Creek Quarry, MT is not asserted in either the original description or redescription. However, a stratigraphic section for the quarry is described in the taphonomic study by Fiorillo (1991). Here he places CCQ very low in the Judith River Fm. However, the base of the Judith River Fm is highly variable in age geographically (see individual entry).

CCQ is geographically located in central Montana, equivalent to ~20-30 km east of Havre (Fiorillo, 1991). This is significant as the age of the base of the Judith River Fm varies east-west depending on whether the equivalent of the Foremost, lower and upper Oldman Fms forms the base. This far east it is unlikely that the base of the Judith River is equivalent to either the Foremost or lower Oldman, and it is probably equivalent to the much younger upper Oldman (Unit 2 & 3).

Hence here I illustrate *Avaceratops* as occurring as an unknown horizon within the range of the upper Oldman Fm (Units 2 and 3). It is noted here that the age of the lower contact of the Oldman Fm is not well understood, such that *Avaceratops* might be older than shown here.

----

### *Machairoceratops cronusi*

***Machairoceratops cronusi* (Lund et al, 2016)**

**"Wahweap centrosaurine B" (Loewen et al., 2013)**

**Wahweap Fm, UT, USA**

Holotype UMNH VP 20550, a partial skull preserving orbital horns, left jugal, braincase, left squamosal, and the posterior part of the parietal includuing the midline with elongate spikes (Lund et al, 2016).

Popular website discussion suggests the small-sized *Diabloceratops* *eatoni* may represent an immature individual (and therefore a junior synonym) of *Albertaceratops* *nesmoi* (Oldman Fm, CAN) with which it shares many anatomical features and may be contemporaneous. However, this hypothesis has not been officially published, and the taxa are considered separate here.

**Stratigraphy**

Lund et al (2016) show that the holotype was recovered from a mudstone near the middle of the upper member of the Wahweap Fm, ~240 m above the base of the formation. This places it ~120 m higher in section than the holotype of *Diabloceratops* which is similar in appearance and closely related.

A radiometric date of 79.9 Ma (Jinnah, 2013) is recorded ~60 m above the base of the Wahweap Fm in the middle Mbr. As the *Machairoceratops* holotype quarry occurs at ~240m above the basal contact of the Wahweap Fm, then its age can be constrained as younger than 79.9 Ma. In a magnetostratigraphic analysis, Albright & Titus (2016) show that the uppermost ~20 m of the ~120 m thick upper mbr is of normal polarity, belonging to C33n, and place the C33r-C33n bondary at 78.91 Ma. Hence the *Machairoceratops* holotype quarry occurs within the reversed C33r zone in the middle of the upper mbr. The age of the *Machairoceratops* horizon is therfore constrained to between 79.9 Ma (radiometric date) and 78.91 Ma (C33r-C33n boundary).

*Machairoceratops* is shown here as a possible range from the lower contact of the upper Wahweap Fm (as plotted here), to the C33r-C33n boundary. However this is quite tenuous, and the relative stratigraphic positions of *Machairoceratops*, *Albertaceratops*, and *Diabloceratops* is fairly flexible, based on the lack of knowledge of the age of the upper half of the Wahweap Formation. It is firmly known that *Diabloceratops* is from lower in the Wahweap Fm than *Machairoceratops*, but it could be that either *Machairoceratops* or *Diabloceratops* is coeval with *Albertaceratops* (and therefore possibly an ontogimorph). Further fine-level stratigraphic knowledge of the upper Wahweap Fm is required to resolve this.

----

### *Xenoceratops foremostensis*

***Xenoceratops* foremostensis (Ryan et al., 2012a)**

**Foremost Fm, Alberta, CAN**

Holotype (CMN 53282; a partial parietal) paratypes (CMN 54950, 54951; parietals) and referred specimens (CMN 54952 through 54965; various cranial elements) were described by Ryan et al. (2012a).

**Stratigraphy**

The type and referred specimens were collected by Wann Langston Jr. in 1958 from a single low-diversity bonebed, illustrated by Ryan et al. (2012a) as occurring immediately beneath the Taber Coal Zone.

The age of the bonebed can be constrained by overlying and underlying radiometric dates. An overlying radiometric date of 79.52 Ma (Goodwin & Deino, 1989; recalibrated here) occurs 4.8m below the top of the marker A coal in the Judith River Fm of northern Montana, which is equivalent to the upper Taber Coal Zone. The underlying radiometric date of 80.17 Ma (Eberth, 2005; recalibrated here) occurs ~30m above the base of the Foremost Fm.

Thus *Xenoceratops* can be constrained as occurring in a single horizon somewhere between 80.17 to 79.52 Ma, and is stratigraphically closer to the 79.52 Ma horizon, hence here it is shown as occurring at 79.6 Ma.

----

### Menefee Fm ceratopsid

**Centrosaurinae sp. (Williamson, 1997)**

**Allison Mbr, Menefee Fm, NM, USA**

The specimen (NMMNH P-25052) comprises a complete left squamosal, partial right squamosal, partial jugal, partial frontal and postorbital, dentary, predentary, coalesced cervical vertebrae, various dorsal vertebrae, ribs, partial ilium, partial sacrum, and a femur (Williamson, 1997).

NMMNH P-25052 was recovered from locality L-3034 situated in the middle of the Allison Mbr of the Menefee Fm, NM, USA. Although fragmentary, this important specimen is potentially the oldest known ceratopsid from North America, although see basal Wahweap ceratopsids (Kirkland, 2001; 2005; Loewen et al., 2013).

The range shown here corresponds to the full estimated duration of the Allison Mbr of the Menefee Fm.

----

### *Pachyrhinosaurus perotorum*

***Pachyrhinosaurus* *perotorum* (Fiorillo & Tykoski, 2012)**

**Prince Creek Fm, AK**

*Pachyrhinosaurus* *perotorum* is described from the Kikak-Tegoseak Quarry bonebed, comprising holotype DMNH 21200 (posterior part of a parietal); paratypes DMNH 22558 (partial skull, missing frill), DMNH 21201 (partial parietal), and many other elements including postcrania (Fiorillo & Tykoski, 2012).

**Age**

Although the Kikak−Tegoseak Quarry is mentioned in a number of publications (Fiorillo et al., 2010; Fiorillo & Tykoski, 2012; 2014; and others), its stratigraphic position within the Prince Creek Fm is not explicitly noted by any authors. Furthermore, although a number of radiometric dates have been retrieved throughout the Prince Creek Fm (Conrad et al., 1992), these are not especially precise (yielding a range from 68.4 - 72.0 Ma; excluding a clearly incorrect single age of 64.19Ma), and are generally referred to by most authors (e.g. Fiorillo et al., 2010) as yielding an average date of 70.0 +/- 0.3 Ma (all dates recalibrated here).

In 1993, Obradovich reanalyzed a sample from one of the lower horizons sampled by Conrad et al. (1992; it is not specified which specific horizon this was). Obradovich's Ar / Ar date is recalibrated here as 73.4 Ma (see individual entry). As a more recent analysis (Ar / Ar, sanidine), this date is probably more reliable than the previous dates of Conrad et al., (1992). This presents some issues in that it is possible therefore that the various dinosaurs described from the Prince Creek Fm might be as old as 73.4 Ma, which is considerably older than the typically cited age of 70.0 Ma (see above).

For the time being, the range of Prince Creek Fm taxa should probably be considered as unresolved until the radiometric dates are fully reanalysed using modern methods and standards. I have therefore plotted the range of *P. perotorum* as unknown between 73.4 and 68.4 Ma.

Fiorillo & Tykoski (2012; 2014) state that *P. perotorum* is currently the youngest known centrosaurine. This is likely, although uncertainty over the age of the Prince Creek Fm means that it is still possible that *P. perotorum* might be of comparable age to pachyrhinosaur material from the Wapiti River Bonebed, Wapiti Fm, Alberta (Fanti et al., 2015).

----

### *Pachyrhinosaurus canadensis*

***Pachyrhinosaurus* *canadensis* (Sternberg, 1950)**

**Horseshoe Canyon Fm, CAN; St Mary River Fm, CAN**

Described by Sternberg (1950) from two specimens. The holotype (NMC8867)and paratype (NMC8866) consist of near complete facial bones and lower jaws, with only the base of the parietal and squamosals that constitute the frill.

The holotype & paratype specimens were recovered from the "Edmonton Fm", now called the Horseshoe Canyon Fm. Eberth et al. (2013) show that *Pachyrhinosaurus* *canadensis* material is only recovered from the Drumheller Mbr (0-1 coal), and the lower half (just below 8 coal) of the overlying Horsethief Mbr of the Horseshoe Canyon Fm.

Hence here I show the range of *P. canadensis* as the time between coals 0-8 of the Horseshoe Canyon Fm.

**St Mary River specimens**

Sternberg (1950) also ascribes a partial skull, NMC8860, to *P. canadensis*. This specimen was collected from Scabby Butte, now recognized as St Mary River Fm (although this is still far from certain, Horner & Weishampel, pers. comm 2007), and roughly equivalent to the type locality. More material is figured by Langston (1975).

----

### *Pachyrhinosaurus lakustai*

***Pachyrhinosaurus* *lakustai* (Currie et al., 2008)**

**Wapiti Fm, Alberta, Canada**

Holotype TMP 1986.55.258 comprises the anterior part of a skull, but there are many hundreds of referred elements from the Pipestone Creek bonebed from which the holotype was recovered, including many additional skulls and an ontogenetic series (Currie et al., 2008).

The Pipestone Creek locality occurs in the basal part of Unit 4, Wapiti Fm, Alberta, 27m below an ash, Ar / Ar dated as 73.73Ma (see Wapiti Fm entries).

Hence here I show *P. lakustai* occurring at 73.8 - 73.9 Ma.

----

### Wapiti River Pachyrhinosaur

**Pachyrhinosaur sp. (Tanke 2006)**

**Wapiti Gp, Alberta, CAN**

Various material has been recovered from the "Wapiti River bonebed"

Currie et al. 2009 note that *Pachyrhinosaurus* specimens from the Wapiti River Bonebed have more smooth supraorbital bosses than those found lower in section from the Pipestone Creek bonebed (*P. lakustai*). They comment that this may be a taxonomic difference, but this requires further study.

Tanke (2004) stated that this horizon (Wapiti River locality) was 114m higher in section than the Pipestone bonebed. However, Tanke (2006) states that the excavated portions of the Wapiti River locality have slumped somewhat, and that their original in situ position is not known. In the most detailed treatment of the locality, Fanti et al. (2015) state that the Wapiti River bonebed is 150 m higher in section than the Pipestone Creek bonebed.

An Ar / Ar date of 71.89 ± 0.14 Ma was recovered from ~180 cm below the bonebed layer (Fanti et al., 2015). Hence here I plot the Wapiti River pachyrhinosaur as 71.8 - 71.9 Ma. This causes overlap with material referred to *P. canadensis* from the upper part of the Horsethief Mbr of the Horseshoe Canyon Fm. This may have taxonomic implications, although this requires analysis of the Horsethief material, which has not been described (see Eberth et al., 2013, for a summary).

----

### *Achelousaurus horneri*

***Achelousaurus* *horneri* (Sampson, 1995)**

**Two Medicine Fm, MT**

*Achelousaurus* *horneri* was originally described as Centrosaurine C (Horner et al., 1992) later being assigned to *Achelousaurus* *horneri* by Sampson (1995). The holotype (MOR 485) is a partial skull, with two additional referred skulls, and postcrania (Sampson, 1995)

*Achelousaurus* occurs at three localities within the Two Medicine Fm. One locality is stratigraphically uninformative, but the other two are equivalent and occur approximately 20m below the contact with the overlying Bearpaw Shale (Sampson, 1995), ~10 m below an Ar / Ar date of 75.04 Ma (plagioclase), and ~50 m above an Ar / Ar date of 75.235 Ma (plagioclase; both dates from Rogers et al., 1993; recalibrated here).

Unless more specimens are discovered, the range of *A. horneri* is thus, limited to a single point in time which does not overlap with any other known centrosaurine taxa.

Therefore, here I show *Achelousaurus* occurring at 75.1- 75.0 Ma.

----

### *Einiosaurus procurvicornis*

***Einiosaurus* *procurvicornis* (Sampson, 1995)**

**Two Medicine Fm, MT**

*Einiosaurus* *procurvicornis* was originally referred to as Centrosaurine B, by Horner et al. (1992) but was later named *Einiosaurus* *procurvicornis* by Sampson (1995). *Einiosaurus* comprises holotype MOR 456-8-9-6-1 (complete skull) and a number of referred elements including additional skulls (Horner et al., 1992; Sampson, 1995).

*Einiosaurus* occurs at two localities within the Two Medicine Fm. These are equivalent (to within a meter) and occur approximately 45m below the contact with the overlying Bearpaw Shale (Sampson, 1995), ~35 m below an Ar / Ar date of 75.04 Ma (plagioclase), and ~25 m above an Ar / Ar date of 75.235 Ma (plagioclase; both dates from Rogers et al., 1993; recalibrated here).

Therefore, here I show *Einiosaurus* occurring at 75.2 - 75.1 Ma.

----

### *Centrosaurine A'*

**Centrosaurinae sp. (Centrosaurine A', Horner et al., 1992)**

**Two Medicine Fm, MT**

Centrosaurine A' is known from six specimens from the Two Medicine Fm, MT.

Centrosaurine A' was not described in detail by Horner et al. (1992), who briefly describe it as possessing a single pair of parietal horns, distinguishing it from *Styracosaurus* *albertensis*. It still possesses a large tall nasal horn, similar to *S. albertensis*.

One of the specimens assigned to Centrosaurine A' (MOR 492) was later referred to *Styracosaurus* *ovatus* by McDonald & Horner (2010), and used to justify erection of a new replacement genus, *Rubeosaurus* *ovatus*. However, current work suggests that MOR 492 might not be referable to *S. ovatus* after all, and hence this material should not be referred to as *Rubeosaurus* (J.P. Wilson, pers. comm.)

The other transitional taxa of Horner et al. (1992) were described by Sampson (1995) as new genera: *Einiosaurus procurvicornis* (B'), and *Achelousaurus horneri* (C').

Stratigraphically Centrosaurine A; material was recovered from approximately 5m below a horizon Ar / Ar dated as 75.235 Ma (plagioclase; Rogers et al., 1993; recalibrated here).

Here I show the specimen occurring at 75.3 - 75.4 Ma, based on the sanidine date.

----

### *Styracosaurus ovatus*

***Styracosaurus ovatus* (Gilmore, 1930)**

**Two Medicine Fm, MT**

*Styracosaurus ovatus* is named for a single parietal fragment from the Two Medicine Fm, MT (USNM 11869, Gilmore, 1930). In 2010, McDonald & Horner referred a partial skull (MOR 492) to the same taxon, renaming it *Rubeosaurus* *ovatus*. In 2011, McDonald also referred USNM 14765 (previously assigned to *Brachyceratops* *montanensis*) to *Rubeosaurus* *ovatus*. Here I restrict *Styracosaurus* *ovatus* to the original type material (USNM 11869; Gilmore, 1930) due to the probability that the material used to assert the new name *Rubeosaurus* may not be referable to *S. ovatus* after all (J.P. Wilson, pers. comm.).

Ryan (2003) states that the type locality is unknown, but it is thought to be near Landslide Butte, Northern Montana: the type locality for *Einiosaurus*. Given the intermediate character states illustrated in the parietal I suspect S. ovatus to occur stratigraphically below centrosaurine A' in the formation. The position as shown in this chart is hypothetical. The original quarry needs to be relocated and its exact stratigraphy calculated.

----

### "Pachyrhinosaur"

**"Pachyrhinosaur" sp. (Ryan et al., 2010b)**

**Dinosaur Park Fm, Alberta, CAN**

A centrosaurine specimen (TMP 2002.76.1) comprising the anterior half of a skull was collected in 2001 in the southeastern portion of Dinosaur Provincial Park (Ryan et al., 2010b). The specimen bears a nasal boss like Achelousaurus and the more derived Pachyrhinosaurus.

Reports of the stratigraphy of the specimen conflict between the original announcement (Ryan & Evans, 2005) and the full write-up (Ryan et al., 2010b). Ryan & Evans (2005), and Ryan (2003) report: "Stratigraphically the specimen occurs in the uppermost DPF, but as the quarry lies within an incised channel, its actual stratigraphic occurrence is at least coeval with the Dinosaur Park-Bearpaw boundary, if not younger (Brinkman, pers. comm.)". In contrast, Ryan et al. (2010b) state that the specimen was recovered 21 m below the Dinosaur Park - Bearpaw Fm contact, at the base of a 9 m thick incised channel, which had cut through the lowest coal of the 15 m thick Lethbridge coal zone. Ryan et al. (2010b) then suggest that the specimen should be considered as having derived from the Lethbridge coal zone.

As such the site is bound by two Ar / Ar dates. The first is dated at 75.46 Ma and occurs 8m above DPFm-Bearpaw contact; the second is dated at 76.10 Ma and occurs 3 m above the base of the Lethbridge coal zone. Here I show the range falling between 75.4 and 76.1 Ma.

This age is slightly unexpected as the specimen bears great similarity to *Achelousaurus* and later centrosaurines bearing nasal bosses (Ryan et al., 2010b), so might have been expected to have been younger. However, it plots in an older position than both *Achelousaurus* and *Einiosaurus*. This might reflect the problematic nature of the dates from the upper Two Medicine Fm (Rogers et al., 1993) as the analyses were conducted over two decades ago, and the source minerals (plagioclase) are not necessarily reliable. It is also possible that, if read literally, this shows that pachyrhinosaur centrosaurines diverged from other centrosaurines earlier than the appearance of *Achelousaurus* (which would suggest that the evolution of a nasal boss might be independent in both lineages). Or it is possible that the Dinosaur Park specimen represents a particularly aged individual. Whether one or many of these issues is the cause, this problem remains unresolved.

----

### *Styracosaurus albertensis*

***Styracosaurus* *albertensis* (Lambe, 1913)**

**Dinosaur Park Fm, Alberta, CAN**

Holotype CMN 344 (a nearly complete skull) was described by Lambe (1913). Many referred specimens are listed by Ryan et al., (2007) including complete skulls, early ontogenetic stages, and bonebed material.

**Lowermost occurrence**

The lowermost specimens of *Styracosaurus* are derived from the BB42 bonebed (Ryan et al., 2007). This is calculated to occur 29 m above the Oldman - Dinosaur Park Fm contact by Mallon et al. (2012), just below a 76.39 Ma bentonite at 36 m above the contact.

**Uppermost occurrence**

Ryan et al. (2007) illustrate TMP.89.97.1 as the highest known specimen of S. albertensis. The precise position of this specimen is not listed by Mallon et al. (2012), nor Currie & Russell (2005). Tanke (2010) notes that the specimen was found outside of Dinosaur Provincial Park, at Sage Creek near Onefour, Alberta, perhaps explaining the lack of precise stratigraphic data. The next highest specimen is listed as TMP 88.36.20 by Ryan et al. (2007), listed as occurring 45 m above the Oldman - DPFm contact by Mallon et al. (2012). However, Mallon et al. (2012) suggest that CMN 344 was recovered 46.9 m above the contact (i.e. there is slight disagreement between Ryan et al., 2007 over the stratigraphically highest specimen). Thus the highest specimens were recovered from above a 76.39 Ma bentonite at 36 m above the Oldman - Dinosaur Park Formation contact, and below a 76.10 Ma bentonite at 61.5 m.

Ryan & Russell (2005) in their description of *C. brinkmani*, state that there is no overlap of centrosaurine taxa (within the DPFm).

Here I have plotted *Styracosaurus* *albertensis* occurring between 76.4 - 76.2 Ma

**Stratigraphic overlap with *C. apertus*?**

**Currie (2005)**

Currie (2005) shows overlap in the ranges of *Styracosaurus* *albertensis* and *C. apertus*. This data is based on the absolute topographic height of the quarries from which the skulls were extracted. Currie's (2005) overlap is due to a single specimen held at the British Museum of Natural History (R8648), which was found significantly lower in the section than all other specimens. This specimen was collected in 1916 and sold to the BMNH by C.H. Sternberg, along with other fossils stated to have derived from the same bonebed, including a pachycephalosaur dome also designated as R8648 (although see *Pachycephalosaurus* entry): attributed to *Pachycephalosaurus* sp. (Wall & Galton, 1979). This is stratigraphically suspect as all other *Pachycephalosaurus* specimens are known from the significantly younger Hell Creek and Lance Fms (for full discussion see *Pachycephalosaurus* entry). Thus it is very likely that there were discrepancies (either intentional or accidental) in the associated R8648 specimens and accompanying data sold to the BMNH. This would not be unprecedented. The *Pentaceratops* skeleton sold to Uppsala by C.H. Sternberg was at least a partial composite containing 2 femora from the same side (Sullivan, pers. comm.). In view of this, I tentatively consider the stratigraphic data of the R8648 "*Styracosaurus*" as potentially erroneous, and will not use it here. This situation may change. The exclusion of BMNH R8648 from the dataset separates the ranges of S. albertensis and *C. apertus* as distinct.

**Ryan et al. 2007**

Note that a parietal fragment (TMP.98.68.33) is catalogued as a *Styracosaurus* albertensis, but that this specimen occurs within the C. apertus zone. Ryan et al. note that this specimen is probably a misidentified *C.* *apertus* P1 (Figure 4) or P3 (Figure 12) parietal spike. If their reassignment is correct, no *Styracosaurus* specimens are known to overlap with *C. apertus*.

----

### *Ce. apertus*

***Centrosaurus* *apertus* (Lambe, 1904)**

**Dinosaur Park Fm, Alberta, CAN**

Holotype NMC 971 is a complete parietal (Lambe, 1904). Since the original description, many specimens, including complete skulls, have been referred to *C. apertus*, including material from various bonebeds.

Ryan and Russell (2005) state that *Centrosaurus* *apertus* is known from the lower 30 m of the Dinosaur Park Formation, Alberta and Saskatchewan, Canada, and the time equivalent middle “muddy unit” of the Oldman Formation, Manyberries region, Alberta, Canada. This is similarly shown by Mallon et al. (2012) where *C. apertus* is limited to the lowermost ~25 m of the Dinosaur Park Fm.

**Stratigraphy**

A radiometric date of76.39 Ma (Eberth, 2011; public lecture) was retrieved from the "Plateau Tuff", which occurs 36m above the Oldman - Dinosaur Park Fm contact (i.e. 6-11 m above the highest known occurrence of *C. apertus*) . A radiometric date of 77.03 Ma (see individual entry) was retrieved from the "field station tuff", which occurs 5.5m below the Oldman - Dinosaur Park Fm contact (i.e. 5.5 m below the lowermost occurrence of *C. apertus*, although a short hiatus occurs across the Oldman - Dinosaur Park Fm contact) . As such, here I have plotted the range of *C. apertus* as 76.9 - 76.5 Ma.

----

### *Spinops sternbergorum*

***Spinops* *sternbergorum* (Farke et al., 2011)**

**Likely Oldman or Dinosaur Park Fm, Alberta, CAN**

Holotype specimen (NHMUK R16307) comprises a parietal; additional specimens recorded as having come from the same bonebed include a partial parietal, dentary, dorsal part of a skull, and a right squamosal (Farke et al., 2011).

**Stratigraphy**

Stratigraphic position of the type bonebed horizon is unknown, although the specimen is known to have been collected in Dinosaur Provincial Park. Personal letters from the discoverer, C. H. Sternberg suggest that the locality is ~90 m lower in section than the quarry from which the type specimen of *Styracosaurus* *albertensis* was recovered, however this is shown to be unlikely as this would place the specimen in the Foremost Fm, and well below any exposures in Dinosaur Provincial Park itself (Farke et al., 2011). Palynological analysis of sediment adhered to Spinops specimens suggest it derives from either the uppermost Oldman Fm, or anywhere within the Dinosaur Park Fm (Farke et al., 2011). Description of the quarry locality as "at floodplain level" by Sternberg suggests that it is no higher than the lower part of the Dinosaur Park Fm (Farke et al., 2011).

As such, here I have plotted the position of *Spinops* as possibly ranging from 77 Ma through 76.39 Ma.

----

### *Coronosaurus brinkmani*

***Coronosaurus* (*Centrosaurus*) *brinkmani* (Ryan & Russell, 2005; Ryan et al., 2012a)**

**Oldman Fm, Alberta, CAN**

Holotype specimen (TMP 2002.68.1) comprises a large adult-sized parietal from a bonebed (no. 138); various referred specimens include many elements derived from the same bonebed as the holotype, and another bonebed located ~180 km SW (Ryan & Russell, 2005).

The holotype bone bed (138) occurs near the top of the upper Oldman Formation, 14.6 m below the contact with the overlying Dinosaur Park Formation (Ryan & Russell, 2005). No precise data is offered for the MRR bonebed from which some referred material was collected.

**Stratigraphy**

The "field station tuff" yields a radiometric date of 77.03 Ma, and occurs 5.5m below the Oldman - Dinosaur Park Fm contact (see individual entry), i.e. 9.1 m above the stratigraphic position of the *Coronosaurus* holotype bonebed. It is likely therefore that *Coronosaurus* occurs at ~77.1 Ma, and this is what is plotted here.

----

### *Wendiceratops pinhornensis*

***Wendiceratops* *pinhornensis* (Ryan & Evans, 2015)**

**Oldman Fm, Alberta, CAN**

Holotype TMP 2011.051.0009 comprises an incomplete parietal lacking the midline bar and left ramus; referred specimens comprise an additional 11 different skull elements, and postcrania; multiple individuals are represented including several parietals and squamosals that share species-level diagnostic characters (Ryan & Evans, 2015).

**Stratigraphy**

The holotype bonebed horizon occurs 10 m above the top of the Taber Coal Zone, immediately above the Heronton sandstone within the lower mud-dominated unit of the Oldman Formation (Ryan & Evans, 2015). This horizon is stratigraphicaly controled by radiometrically dated horizons in the stratigraphically equivalent Judith River Formation near Rudyard, Montana (Goodwin & Deino, 1989; see individual entries).

## basal ceratopsians

### *Leptoceratops*

***Leptoceratops* *gracilis* (Brown, 1914c)**

**Scollard Fm, Alberta, CAN; Pinyin Conglomerate & Lance Fm, WY; Hell Creek Fm, MT**

*Leptoceratops* is known from 7 published specimens, including type and paratype, from the Scollard Fm, Canada (Brown, 1914c; Sternberg, 1951; Ott, 2006). Material is also recorded from Wyoming: the Lance Fm, (Ostrom, 1978) & Pinyin Conglomerate (McKenna & Love, 1970).

Two specimens referred to *L. gracilis* were recovered from the Hell Creek Fm, MT (UWGM-200: partial skull, & UWGM-201: maxillary tooth with root; Ott, 2006). Much new material (unpublished) has been recently recovered by field crews led by D.Fowler for both Museum of the Rockies and Dickinson Museum Center, ND., substantially increasing our knowledge of this taxon.

Published stratigraphic data is not available for many specimens. Specimens recovered from the Scollard Fm, Alberta are all recorded as either from the lower half, or lowermost Scollard (Eberth et al., 2013). Stratigraphic data is not explicitly recorded for US specimens (although Ostrom, 1978, noted that the Lance Fm specimens came from "quite close to the top of the Cretaceous", it is not clear what he meant by this and no useful details are offered), but I have personally observed many *Leptoceratops* sites in the Hell Creek Fm of Montana, all of which follow the observations of Eberth et al. (2013) in that they occur in the lower half of the unit.

I therefore plot *Leptoceratops* occurrence as limited to the lower half of the Scollard and Hell Creek Fms.

----

### *Montanoceratops cerorhynchus*

**Montanoceratops cerorhynchus (Brown & Schlaikjer, 1942)**

**St Mary River Fm, MT; Horseshoe Canyon Fm, Alberta, CAN.**

*Montanoceratops* is described from the the St Mary River Fm, MT (Brown & Schlaikjer, 1942; Chinnery & Weishampel, 1998), and the Tolman Mbr of the Horseshoe Canyon Fm, Canada (Eberth et al., 2013).

The holotype specimen (AMNH 5464; Brown & Schlaikjer, 1942) and referred specimen (MOR 542; Chinnery & Weishampel, 1998) are both thought to have been collected from the same horizon. This is described by Brown & Schlaikjer (1942) as being in the lower part of the St. Mary River Fm, near Buffalo Lake, Montana.

Thus here I illustrate the range of *Montanoceratops* as from the lower contact of the St Mary River Fm through to the top of the Tolman Mbr of the Horseshoe Canyon Fm.

----

### *Prenoceratops pieganensis*

***Prenoceratops* *pieganensis* (Chinnery, 2004)**

**Two Medicine Fm, MT, USA**

*Prenoceratops* is known from a single monospecific bonebed comprising multiple individuals (Chinnery, 2004).

The *Prenoceratops* bonebed occurs on private land in the Two Medicine Fm, Montana, 50m below the contact with the overlying Bearpaw Shale (Chinnery, 2004). The stratgraphic placement of this taxon is dependent on where in the Two Medicine Fm the bonebed occurs. The Bearpaw Shale overlies the Two Medicine Fm relatively earlier in more eastern sections, so this current placement could change by as much as 500 Ky older. Here I am simply assuming that the stratigraphy of the *Prenoceratops*-bearing section follows that which I illustrate here.

An ash ~10m below the contact with the Bearpaw Shale yields an Ar / Ar date of 75.038 Ma, and an ash ~70 m below the contact yields an Ar / Ar date of 75.235 Ma (both dates based on plagioclase; Rogers et al., 1993; recalibrated here). Therefore I have plotted *Prenoceratops* occurring between 75.1 and 75.2 Ma.

----

### *Unescoceratops koppelhusae*

***Unescoceratops* *koppelhusae* (Ryan et al., 2012b)**

**Oldman Fm, Alberta, CAN**

Holotype (TMP 95.12.6) is a partial left dentary, and referred specimen (TMP 74.10.31), a partial right dentary (Ryan et al., 2012b).

**Stratigraphy**

The holotype was collected from bonebed 55 in the Steveville region of Dinosaur Provincial Park, which occurs in the middle of the Dinosaur Park Fm, approximately 40 m above the contact with the Oldman Fm (Ryan et al., 2012b). This therefore places it ~4 m above the Plateau tuff, dated at 76.39 Ma, which occurs 36 m above the Oldman - Dinosaur Park Fm contact, and below the LCZ tuff, dated at 76.10 Ma, which occurs 61.5 m above the Oldman - Dinosaur Park Fm contact (see main entries).

The referred specimen was also collected from Dinosaur Provincial Park, and is cited as "probably from the Dinosaur Park Formation" by Ryan et al. (2012b).

----

### *Cerasinops*

***Cerasinops* *hodgkissi* (Chinnery & Horner, 2007)**

**Two Medicine Fm, MT**

Holotype MOR 300 is an associated skull and skeleton; referred specimens are USNM 13863 and 13864, both partial skeletons previously referred to Leptoceratops sp. (Gilmore, 1939; Chinnery & Horner, 2007).

The holotype specimen was collected from the Red Rocks Site, Teton County, Montana, whereas the referred specimens were excavated from Pondera County, Montana (Chinnery & Horner, 2007). All specimens are from lithofacies 3 in the lower Two Medicine Formation.

Here I show the possible position of *Cerasinops* as a variable arrow denoting the full duration of lithofacies 3.

----

### *Gryphoceratops morrisoni*

***Gryphoceratops* *morrisoni* (Ryan et al., 2012b)**

**Milk River Fm, Alberta, CAN**

The holotype (ROM 56635) is a partial right dentary.

**Stratigraphy**

The holotype and only known specimen was collected from Black Coulee (formerly Deadhorse Coulee), approximately 10 km east of Writing-on-Stone Provincial Park, Alberta, from the Deadhorse Coulee Mbr, Milk River Fm (Ryan et al., 2012b; see individual entry).

Hence here I show the range of *Gryphoceratops* as the range of the Deadhorse Coulee Mbr.

----

## Pachycephalosauridae

### *Sphaerolothus buchholtzae*

***Sphaerolothus* *buchholtzae* (Williamson & Carr, 2002)**

**=?*Prenocephale* (*Stegoceras*) *edmontonensis* (Sullivan, 2000)**

**Hell Creek Fm, MT**

The holotype TMP 87.113.3 was recovered from the Hell Creek Fm of Montana (Williamson & Carr, 2002). Greater stratigraphic detail is not given, although small-headed pachycephalosaur remains are known from the Jen Rex sst (middle Hell Creek Fm; pers. obs.), so the taxon ranges at least ~half way up the Hell Creek Fm. Here I have plotted the range of *S. buchholtzae* as unknown within the duration of the Hell Creek Fm.

Sullivan (2000; 2003; 2006) considers *S. buchholtzae* as a junior synonym of *Prenocephale edmontonensis*, which is defined based on specimens recovered from the Horseshoe Canyon Fm, ~3.5 m.y. older than the Hell Creek Fm.

Here I show *S. buchholtzae* and *P. edmontonensis* as separate simply because of the stratigraphic difference:

More discussion on the possible synonymy of *Sphaerotholus* *buchholtzae* and *P. edmontonensis* is given in the entry for *P. edmontonensis*.

----

### *Prenocephale edmontonensis*

***Prenocephale* (Troodon; *Stegoceras*) *edmontonensis* (Brown & Schlaikjer, 1943; Sullivan, 2000)**

**Tolman Mbr, Horseshoe Canyon Fm, Alberta, CAN**

*Prenocephale* *edmontonensis* has a contorted and confusing taxonomic history, and I am probably not going to make things much better here! The name *Troodon* *edmontonensis* was erected by Brown & Schlaikjer (1943), later amended to *Stegoceras* *edmontonensis*, *S. edmontonense*, then *Prenocephale* *edmontonensis* (see Sullivan, 2000, for details).

The holotype of *P. edmontonensis* (CMN 8830), a water abraded frontal, and two paratypes (CMN 8831 and 8832) were recovered from the same locality in the Horseshoe Canyon Fm (Brown and Schlaikjer, 1943), probably above the Drumheller Marine Tongue (Sullivan, 2000; 2006) in the lowermost part of the Tolman Mbr (Eberth et al., 2013). Additional specimens were referred to *P*. (*Stegoceras*) *edmontonensis* by Chapman et al. (1981; LACM 15345, 64000) and Giffin (1989; TMP 87.113.3). In renaming the taxon *P. edmontonensis*, Sullivan (2000) moved LACM 15345 into cf. *P*. edmontonensis, but otherwise agreed with the prior referrals. It should be noted here that the two LACM specimens and the TMP specimen were recovered from the Hell Creek Formation of Montana, not the Horseshoe Canyon Fm, and as such are ~3.5 m.y. younger.

The current status of *P. edmontonensis* is in flux. In a paper describing a new pachycephalosaur taxon, *Sphaerotholus*, Williamson & Carr (2002) suggested that the holotypic material of *P. edmontonensis* was undiagnostic, but tentatively referred most *P. edmontonensis* specimens to *Sphaerotholus* (no species is given), and made TMP 87.113.3 (previously referred to *P. edmontonensis* by Giffin, 1989, and Sullivan, 2000) the holotype of a new species, *Sphaerotholus* *buchholtzae* . Sullivan (2003) reaffirmed the validity of *P. edmontonensis* and further suggested that *Sphaerotholus* *buchholtzae* was therefore a junior synonym of *P. edmontonensis*. In the most recent treatment of pachycephalosaurs, Evans et al. (2013a) do not feature *P. edmontonensis*, but do include *Sphaerotholus* *buchholtzae*, which is shown with a range extending from the Horseshoe Canyon Fm to the Hell Creek Fm, thereby implying that *S. buchholtzae* includes all the holotypic and referred material of *P. edmontonensis* (ie. a stronger claim than Williamson & Carr, 2002, who did not assign a species designation to the *P. edmontonensis* material, merely referring it to either *Sphaerotholus*, or *Sphaerotholus* sp.)..

Here I take a slightly different approach. Given that the emphasis of this work is stratigraphy, then I have chosen to illustrate Horseshoe Canyon Fm *P. edmontonensis* material as separate from material from the Hell Creek Fm (which is shown separately here as *Sphaerotholus* *buchholtzae*). This is simply to show the stratigraphic separation between the specimens, which would typically result in slight difference in morphology and hence taxonomy*. P. edmontonensis* is therefore shown occurring in the lower half of the Tolman Mbr of the Horseshoe Canyon Fm (Eberth et al., 2013).

----

### *Prenocephale goodwini*

***Prenocephale* (*Sphaerotholus*) *goodwini* (Williamson & Carr, 2002; Sullivan, 2003)**

**De-na-zin Mbr, Kirtland Fm, NM**

Known from a single skull lacking the anterior 'facial' bones (NMMNH P-27403). This was described as a new genus, *Sphaerotholus* *goodwini*, by Williamson & Carr (2002). Sullivan (2003; 2006) states that characters used to assert generic distinction are not valid, and that instead, this is another North American occurrence of the 'typically' Asian genus *Prenocephale* (see entries for *P. brevis* & *P. edmontonensis*).

**Stratigraphy**

The specimen was collected from the De-na-zin Mbr of the Kirtland Fm, NM. The level within the member is not explicitly mentioned by Williamson & Carr (2002) so is shown here as an unknown arrow within the range of the De-na-zin Mbr.

----

### cf. *P. goodwini*

**cf. *Prenocephale* (*Sphaerotholus*) *goodwini***

**Farmington Mbr, Kirtland Fm, NM**

NMMNH P-30068 comprises a left dentary, squamosal fragment, and unidentified cranial fragment, tentatively ascribed to cf. *Sphaerotholus* *goodwini* by Williamson & Carr (2002). The elements comprising NMMNH P-30068 are assumed to represent a single inidivdual (Williamson & Carr, 2002) but may be from different individuals (Sullivan, 2003). It is not clear in the original description how reliable the assumption might be, especially since the specimens were recovered along with a *Daspletosaurus* skeleton, which had been partially removed illegally (not by the authors).

Sullivan (2003) reassigns all *Sphaerotholus* remains to the genus *Prenocephale*.

The holotype of *P*. (*Sphaerotholus*) *goodwini* was collected from the De-na-zin Mbr of the Kirtland Fm, whereas NMMNH P-30068 was collected from the underlying Farmington Mbr (Sullivan, 2003). There is a conspicuous change in both sedimentology and fauna between the two members, which would lead me to be more cautious in assigning such incomplete remains to *P. goodwini*, hence the separation here.

----

### *Foraminacephale brevis*

***Foraminacephale* *brevis* (*Stegoceras* *breve*; Lambe 1918), (*Prenocephale* *brevis*; Sullivan, 2000), (Schott & Evans, 2016)**

**Oldman & Dinosaur Park Fms, Alberta, CAN**

Holotype CMN 1423, a complete frontoparietal dome; referred specimens CMN 121, 193, 194, 8819, and TMP 85.36.292, 91.36.265, 99.55.122, and 2000.12.01, are also frontoprietal domes (Lambe, 1918; Sullivan, 2000). Placed into its own genus, *Foraminacephale*, by Schott & Evans (2016), who assign new specimens to the taxon, including elements not previously described; CMN 12351 and TMP 1985.043.0061, parietals; TMP P70.10.2, a partial frontoparietal with articulated partial squamosal, postorbitals, and posterior supraorbitals; ; TMP 1986.077.0085 and TMP 1992.036.1125, postorbitals; TMP 1987.050.0029, frontoparietal; and UALVP 49440, squamosal.

**Stratigraphy**:

Stratigraphy of *Foraminacephale* is uncertain (see review in Schott & Evans, 2016).

The holotype and most of the referred specimens are stated as having been collected from the Oldman Fm (Sullivan, 2000), with the exception of three specimens from the Dinosaur Park Fm (TMP 91.36.265; 99.55..122 and 2000.12.01). However, no detailed justification is given for the stratigraphic provenance of the specimens. Given that CMN specimens were mostly historically collected then it is possible that some might originate from the Dinosaur Park Fm. This is speculation on my behalf, but reference to Lambe (1918) shows that the localities are weakly described and the formation given as Belly River Fm. Moreover, the term "Oldman Fm" previously referred to the entirety of what are now called the Oldman and Dinosaur Park Fms (see Eberth, 2005). Regardless, it is likely that reference to the Oldman Fm probably does not include the lower Oldman Fm (Unit 1).

Schott & Evans (2016) note that the only specimen defintively collected from the Oldman Fm is TMP 2015.044.0041, which was recently colected from Unit 3 of the Oldman Fm exposed near Onefour in southern Alberta. In this area, Unit 3 of the Oldman Fm is partly correlative in age with the lower part of the Dinosaur Park Fm.

It is therefore possible that *F. brevis* is restricted to the time represented by the Dinosaur Park Fm, or the age equivalent, part of the Oldman Fm. There is no strong evidence that F*. brevis* is any older than this (see Schott & Evans, 2016).

Here, the range of *F. brevis* is shown as unknown from the base of the Comrey Sst (basal unit of the upper Oldman Fm) to the top of the fossiliferous zone within the Dinosaur Park Fm (ie. below the Lethbridge Coal Zone). This is more inclusive than what is likely based on Schott & Evans (2016).

----

### *Stygimoloch spinifer*

***Stygimoloch* *spinifer* (Galton & Sues, 1983):**

**=?*Stenotholus* *kohleri* (Giffin et al., 1987; Gabriel & Berghaus, 1988)**

**=?*Dracrorex* *hogwartsia* (Bakker et al., 2006)**

**Hell Creek Fm, MT, ND, SD**

*Stygimoloch spinifer* was described by Galton & Sues (1983) based on two isolated squamosals (holotype: UCMP 119433, and YPM 335), and defined as possessing a cluster of three to four large spikes, as opposed to the rounded knobs seen on *Pachycephalosaurus*. Upon discovery of more complete material (MPM 8111), their original diagnosis was emended by Goodwin et al. (1998), to include an elongate fronto-parietal dome. Sullivan (2006) notes that the parietal sometimes does not extend between the squamosals to help form the shelf at the posterior of the skull.

**Ontogeny**

Horner & Goodwin (2009) proposed that *Stygimoloch* *spinifer* and the similar *Dracorex* *hogwartsia* were simply younger ontogenetic stages of *Pachycephalosaurus*, demonstrating histologically that that the elongate squamosal spikes of the immature morph "*Stygimoloch*" were eroded and resorbed through ontogeny to produce the rounded knobs of the mature form *Pachycephalosaurus* (see individual entry). Despite this, there is evidence to suggest stratigraphic separation, with *Pachycephalosaurus* specimens collected from low in the Hell Creek Formation (or equivalent), and *Stygimoloch* specimens found relatively high (see below).

**Stratigraphy**

Most descriptions of *Stygimoloch* material are not accompanied by good stratigraphic data. However, those specimens which do have stratigraphic data occur mostly in the upper Hell Creek, with some in the middle of the unit. Goodwin et al. (1998) describe specimen MPM 8111 from MPM locality 3956: in the "upper third" of the 70-90m thick Hell Creek Fm near Martmarth, ND. Other referred specimens in Goodwin et al. (1998) have only locality numbers, not specific stratigraphic position. The holotype of *Dracorex* (TCMI 2004.17.1), probably a pre-domed juvenile of *Stygimoloch*, was collected from the "middle part" of the Hell Creek Fm, ND (Bakker et al., 2006).

Although this data is very limited, I am keeping *Pachycephalosaurus* and *Stygimoloch* as separate taxa, although this remains speculative until better data is published. It is worth noting that no large pachycephalosaur squamosals are currently recorded from the upper Hell Creek Formation (including unpublished specimens with which I am familiar), such that we almost certainly do not have mature specimens of *Stygimoloch* to compare with the large-sized specimens of *Pachycephalosaurus*.

The highest known pachycephalosaur material of which I am aware are unpublished MOR specimens from ~5 m below the top of the Hell Creek Formation, Montana.

***Stenotholus***

Giffin et al. (1987) described *Stenotholus* kohleri (MPM 7111) from a narrow fronto-parietal dome, found in the Hell Creek Fm, McCone county, MT, approximately 100ft above the contact with the underlying Fox Hills (placing it in the lower part of my middle Hell Creek). Associated squamosal spikes and the narrowness of the dome were used to synonymise it with *S. spinifer* (Gabriel & Berghaus, 1988; Sullivan, 2003). However, given that the narrowness of the dome may be an ontogenetic character, using it for synonymy is possibly erroneous. The squamosal spikes of MPM 7111 are not as elongate as those seen in other specimens of *Stygimoloch*, suggesting they may represent an intermediate form, corroborated by their stratigraphic position.

----

### *Pachycephalosaurus wyomingensis*

***Pachycephalosaurus* *wyomingensis* (Gilmore, 1931)**

**=*Tylosteus* *ornatus* (Leidy, 1872); =*P. grangeri* & *P. reinheimeri* (Brown & Schlaikjer, 1943)**

**=?*Stygimoloch* *spinifer* (Galton & Sues, 1983)**

**=?*Dracorex* *hogswartsia* (Bakker et al., 2006)**

**Lance Fm, WY; Hell Creek Fm, ND, SD, MT**

Holotype (USNM 12031) is a frontoparietal dome and squamosal shelf from the Lance Fm, WY, originally named *Troodon* *wyomingensis* (Gilmore, 1931), being later recombined as *Pachycephalosaurus* *wyomingensis* (Brown & Schlaikjer, 1943; Galton, 1971). Many additional specimens have been attributed to *P. wyomingensis* (see review by Sullivan, 2006), including numerous unpublished skulls, some of which are complete or nearly so.

*Stygimoloch* is *Pachycephalosaurus*?

Two related taxa have been described from the Hell Creek Formation: *Stygimoloch* *spinifer* is diagnosed by having more pointed squamosal nodes (Galton & Sues, 1983) and possibly a narrower frontoparietal dome (Goodwin, 1998); *Dracorex* *hogwartsia* (Bakker et al., 2006) also exhibits pointed squamosal spikes, but the frontal and parietal are not inflated into a dome. Known examples of these taxa are notably smaller than specimens attributed to *Pachycephalosaurus* which exhibit more rounded squamosal spikes.

Horner & Goodwin (2009) suggested that *Dracorex* *hogswartsia* and *Stygimoloch* *spinifer* represent immature forms of *Pachycephalosaurus* *wyominensis* rather than distinct taxa. This proposes that the frontoparietal dome inflates through ontogeny, and that the elongated squamosal spikes of the *Stygimoloch* ontogimorph eroded through ontogeny resulting in the short rounded nodes of *Pachycephalosaurus*.

A complication to the ontogimorph hypothesis arises due to a skull collected from the "Sue" *Tyrannosaurus* quarry (Galliano & Mehling, 2001; Sullivan, 2006) which is approximately the same size as specimens of *Dracorex* or *Stygimoloch*, with a dome intermediate in size between the two, suggesting that it represents an intermediate ontogimorph. However, the squamosals of this specimen do not exhibit elongate spikes; instead retaining short weakly spiked nodes, most similar to specimens attributed to *Pachycephalosaurus*. This suggests that either the elongate spikes are strongly variable within *Pachycephalosaurus*, or that *Stygimoloch* is possibly distinct from *Pachycephalosaurus*. The stratigraphy of known specimens suggests that there is stratigraphic separation between specimens referred to *Pachycephalosaurus* and *Stygimoloch*, although it should be noted that *Pachycephalosaurus* specimens are known to exhibit strong variation in the squamosal with some individuals exhibiting differing numbers of nodes on left and right sides (Horner, pers. comm. 2010).

**Stratigraphic range**

Precise stratigraphic data was rarely collected for historical specimens of *Pachycephalosaurus*, and locality quarries were typically insubstantial in size and thus difficult to relocate. However, all *Pachycephalosaurus* specimens for which we have reliable data were found low in their respective sections; the fine holotype of "*P. grangeri"* (AMNH 1696; Brown & Schlaikjer, 1943) was found low in the Hell Creek Fm near Ekalaka, MT (Horner, pers. comm. 2008); the undescibed juvenile (Galliano & Mehling, 2001) originates from the Sue *T. rex* quarry: 15 m above the Hell Creek-Fox Hills contact near Faith, SD (Larson, 2008; although from photos of the site in Galliano & Mehling, 2001, it appears that the specimen is not from the basal sand). Various undescribed specimens collected by Museum of the Rockies (pers. obs.) also occur low in section.

In contrast, the few *Stygimoloch* specimens which have stratigraphic data (see individual entry) were recovered from high in the Hell Creek Formation. For this reason, the taxa are plotted separately here, although clearly more specimens are needed, especially those with stratigraphic data.

**Taxonomic note**

*P. grangeri* & *P. reinheimeri* were synonymised with *P. wyomingensis* (Galton, 1971; Galton & Sues, 1983; Sues & Galton, 1987). The type specimen for *Tylosteus* (ANSP 8568; Leidy, 1872) is a left squamosal, exhibiting short knob-like nodes, and as such is synonymous with *P. wyomingensis* (Baird, 1979; although Sullivan, 2006 assigns it to *Dracorex* = *Stygimoloch*). Although *Tylosteus* ornatus is the senior synonym, the synonymy was not recognised for over a century, and as such Baird (1979) considered *Tylosteus* a nomen oblitum, retaining *Pachycephalosaurus* to preserve taxonomic stability, and because *Tylosteus* had only dubious geographic and stratigraphic data.

**Stratigraphic inconsistencies: London *Pachycephalosaurus***

R8648 (Natural History Museum, London) is a specimen attributed to *Pachycephalosaurus* sp. by Wall & Galton (1979), collected from a *Styracosaurus* bonebed by C. M. Sternberg in 1916. The specimen was found "low in the strata of the Oldman Formation [Canada] about 3 miles south of the Steveville Ferry, on the east side of the Red Deer River" (D. A. Russell and C. M. Sternberg, personal communication, 1976). This would make R8648 the oldest known occurrence of *Pachycephalosaurus* by a considerable margin (~12Ma) and the only known specimen from Canada (at least up until 1979). Although this provenance data was accepted by Wall & Galton (1979), it is inconsistent with the fact that all other *Pachycephalosaurus* specimens come from the Hell Creek and Lance Fms (late Maastrichtian), USA. Consequently, it is suspected to be incorrect by Sullivan (2006), and here I treat this specimen as of unknown stratigraphic position. Note that this specimen is referred to as R8646 by Sues & Galton (1987), Ryan & Evans (2005), and Williamson & Carr (2006). it is not clear which is the correct number.

----

### *Alaskacephale gangloffi*

***Alaskacephale* *gangloffi* (Sullivan, 2006):**

**Prince Creek Fm, AK**

Holotype is a near-complete left squamosal (UAM#AK-493-V-001) with a unique double row of nodes (Gangloff et al., 2005). This led Sullivan (2006) to erect a new taxon, Alaskacephale gangloffi, for the previously unnamed remains. Gangloff et al. (2005) note that closely spaced nodes could be considered as clusters and are as such, similar to what is seen in the younger taxon, *Pachycephalosaurus* *wyomingensis*.

**Stratigraphy**

The holotype was collected from talus accumulated on a beach below cliffs comprised of the Prince Creek Fm (Gangloff et al., 2005). As such stratigraphic position within the Prince Creek Fm is not precisely known, other than that it is presumably derived from the adjacent cliffs.

Gangloff et al., (2005) state that UAM#AK-493-V-001 was collected 4 km from hadrosaur bonebeds and 25 km from Ocean Point; the Prince Creek Fm strata both above and below these localities have yielded a number of radiometric dates (Conrad et al., 1992), although these are not especially precise (yielding a range from 68.4 - 72.0 Ma; excluding a clearly incorrect single age of 64.19Ma), and are generally referred to by most authors (e.g. Fiorillo et al., 2010) as yielding an average date of 70.0 +/- 0.3 Ma (all dates recalibrated here).

In 1993, Obradovich reanalyzed a sample from one of the lower horizons sampled by Conrad et al. (1992; it is not specified which specific horizon this was). Obradovich's Ar / Ar date is recalibrated here as 73.4 Ma (see individual entry). As a more recent analysis (Ar / Ar, sanidine), this date is probably more reliable than the previous dates of Conrad et al., (1992). This presents some issues in that it is possible therefore that the various dinosaurs described from the Prince Creek Fm might be as old as 73.4 Ma, which is considerably older than the typically cited age of 70.0 Ma (see above).

Based on the Obradovich reanalysis, Gangloff et al., (2005) suggest that *Alaskacephale* *gangloffi* is probably Late Campanian in age.

For the time being, the range of Prince Creek Fm taxa should probably be considered as unresolved until the radiometric dates are fully reanalysed using modern methodology and standards. I have therefore plotted the range of *A. gangloffi* as unknown between 73.4 and 68.4 Ma.

----

### Horsethief pachycephalosaur

**Horsethief pachycephalosaur (Eberth et al., 2013)**

**Horsethief Mbr, Horseshoe Canyon Fm, Alberta, CAN**

Eberth et al. (2013) mention three "skull caps" (TMP 1984.012.0004; CMN 11316; UALVP unknown number) collected from the Horsethief Mbr of the Horseshoe Canyon Fm. The specimens were collected from between coals 7-9 (TMP 1984.012.0004) or 8-9 (other specimens; Eberth et al., 2013).

Eberth et al. (2013) note tht the specimens bear similarity to *Colepiocephale*, which might be surprising since this taxon is much older, from the Foremost Fm. The specimens await formal description and are mentioned here as if they are related to the *Colepiocephale* clade, then they may represent the highest known occurence of this *Stegoceras*-allied clade.

The range is shown here as unknown within the Horsethief Mbr of the Horseshoe Canyon Fm.

----

### *Stegoceras novomexicanum*

***Stegoceras* *novomexicanum* (Jasinski & Sullivan, 2011)**

**Fruitland & Kirtland Fms, NM**

Jasinski & Sullivan (2011) describe *S. novomexicanum* from holotype NMMNH P-33898 (note it is incorrectly referred to as NMMNH P-33983 in their figure 3), a nearly complete frontoprietal dome, and paratypes SMP VP-2555, a posterior part of a left frontal and anterior part of left and right frontals, and SMP VP-2790, an incomplete parietal.

**Stratigraphy**

The holotype NMMNH P-33898 and paratype SMP VP-2555 were collected from the upper part of the Fruitland Fm (Sullivan & Lucas, 2006; Jasinski & Sullivan, 2011). Paratype SMP VP-2790 was collected from the lower part of the Hunter Wash Mbr of the Kirtland Fm (shown immediately above the Bisti Bed sst by Jasinski & Sullivan, 2011). Jasinski & Sullivan (2016) describe additional specimens from both the upper part of the Fruitland Fm and lower Hunter Wash Mbr of thr Kirtland Fm.

Hence here I show the range of *S. novomexicanum* as from the uppermost Fruitland Fm, to the lowermost Kirtland Fm.

----

### *Stegoceras validum*

***Stegoceras* *validum* (Lambe, 1918)**

**Dinosaur Park Fm, Alberta, CAN**

*Stegoceras validum* has a long and contorted taxonomic history, beginning with its description as *Stegoceras* *validus* by Lambe (1902), with many subsequent additions and revisions. *S. validum* is known from dozens of frontoparietal domes (Sullivan, 2006; Schott et al., 2011), including one complete skull (Gilmore, 1924).

Despite being relatively abundant, stratigraphic data for S. validum is scarce. Most publications merely refer to the specimens having derived from the Dinosaur Park Formation, making a true stratigraphic range difficult to ascertain.

Currie & Russell (2005) give the position of UA 02 as an elevation of 667.5, placing it very low in the Dinosaur Park Fm. Schott et al., (2011) note that newly referred specimens UCMZ(VP) 2008.001 was recovered 37m above the basal contact of the Dinosaur Park Fm, and UALVP 49531 40m above the contact, both therefore approximately halfway through the formation.

I have therefore chosen to show the range of *S. validum* as from the base of the Dinosaur Park Fm, to approximately halfway.

----

### *Hanssuesia sternbergi*

***Hanssuesia* (*Stegoceras*) *sternbergi* (Sullivan, 2003)**

**Dinosaur Park Fm, Alberta, CAN (although see below)**

Holotype CMN 8817, a nearly complete frontoparietal dome, was described as *Troodon* *sternbergi* by Brown and Schlaikjer (1943), then changed to *Stegoceras* *sternbergi* by Sternberg (1945). Sullivan (2003) reassigned the type specimen to a new genus, *Hanssuesia* *sternbergi* (new gen. et comb.), and referred an additional 11 specimens, some of which had been previously referred to other taxa (see Sullivan, 2003, for details).

**Stratigraphy**

Sullivan (2003) notes that the holotype CMN 8817 may derive from the Oldman Fm, citing Eberth pers. comm.. Otherwise, Sullivan (2003) states that all specimens are known from the Dinosaur Park Fm, but no further details are given. Sullivan (2006) states that the stratigraphic range of *Hanssuesia* *sternbergi* is from the Oldman to Dinosaur Park Fm, but gives no reference for the more definitive assertion of an Oldman age (nor which part of the Oldman Fm this might refer to).

I am unaware of another published source which cites the position within the Dinosaur Park Fm of *Hanssuesia* specimens.

Here I show the range of *Hanssuesia* as unknown within the full duration of the Dinosur Park Fm.

----

### *Texacephale*

***Texacephale* *langstoni* (Longrich et al., 2010)**

**Aguja Fm, TX**

Holotype LSUMNS 20010 is a weathered frontoparietal dome; referred specimen LSUMNS 20012 is an incomplete frontoparietal dome (Longrich, 2010). Jasinski & Sullivan (2011) considered the type material undiagnostic and therefore consider *Texacephale* *langstoni* as a nomen dubium.

Note that additional pachycephalosaur material from the Aguja (and equivalent San Carlos) Fm is mentioned by Lehman (1985), but not noted by Longrich et al. (2010). See individual entry for more information.

**Stratigraphy**

Longrich et al. (2010) state that holotype LSUMNS 20010 was recovered from the WPA-1 quarry (from which many chasmosaurine remains have also been recovered and referred to *Agujaceratops*; see individual entry), and that referred specimen LSUMNS 20012 was recovered from a different site "tens of metres above WPA-1".

Here I have limited the range of *Texacephale* to be equivalent to the WPA-1 bonebed (see *Agujaceratops* entry).

----

### Aguja & San Carlos Fm pachys

**Pachycephalosauria sp., (Lehman, 1985)**

**San Carlos & Aguja Fm, TX**

In his PhD dissertation, Lehman (1985; p. 262) states:

"A pachycephalosaur skull fragment (TMM 42010-1) also from the Aguja (probably the upper shale mbr, although the exact horizon is not known) resembles *Stegoceras*. Parts of three better preserved skull caps from the upper shale mbr of the San Carlos are similar in form, and more confidently referred to Stegoceras (TMM 42532-1, -2, and -3)."

At the time of publication (1985), all pachycephalosaurs from the Campanian of North America were designated as *Stegoceras*, so it is possible that the Aguja & San Carlos Fms specimens are additional specimens of taxa named or recognised since 1985.

These specimens remain undescribed and were not mentioned by Longrich et al. (2010) in their description of the only other pachycephalosaur material from the Aguja Fm, the holotype and referred specimen of *Texacephale* *langstoni*.

**Stratigraphy**

The San Carlos Fm is not shown in this current version of the chart, but is equivalent to the Aguja Fm (Lehman, 1985) and is treated here as such. Range of the undescribed material is shown here as spanning the "main unit" of the Upper Member of the Aguja Fm, although likely it pertains to a much more narrow duration.

----

### *Colepiocephale*

***Colepiocephale* (*Stegoceras*) *lambei* (Sternberg, 1945; Sullivan, 2003)**

**Foremost Fm, Alberta, CAN; Judith River Fm, MT**

Holotype CMN 8818 was originally described as *Stegoceras* *lambei* by Sternberg (1945). In his revision, Sullivan (2003) considered its morphology sufficiently unique to erect a new genus, *Colepiocephale*, and refers 13 additional specimens to the species. Schott et al. (2009) later refer an additonal nearly complete dome, UCMP 130048.

**Stratigraphy**

Baszio (1997) described six pachycephalosaur domes from Chin Coulee, near Foremost, Alberta. Two of these (TMP 86.146.1 and 86.146.2) were complete enough to be referred to C. lambei by Sullivan (2003). These are the oldest known examples of *Colepiocephale* as Chin Coulee exposures are shown by Baszio (1997) as being in the middle of the Foremost Fm, below the Taber Coal Zone. This would appear to agree with the range for *Colepiocephale* shown by Schott (2009), where the older end of the range is shown immediately above the McKay Coal Zone of the Foremeost Fm.

The uppermost occurrence of *Colepiocephale* is UCMP 130048, collected from a shelly horizon in the Judith River Fm, ~25 m above the Marker A coal in Kennedy Coulee, Montana (Schott, 2009). This should be from above the Herronton Sandstone equivalent (well exposed in Kennedy Coulee immediately above the Marker A coal) which is equivalent to Unit 1 of the lower Oldman Fm of Alberta.

----

### Middle Wahweap pachycephalosaur

**Pachycephalosauria indet. (Evans et al., 2013b)**

**Wahweap Fm, UT**

Evans et al. (2013b) describe UMNH VP 11939, a weathered anterior part of a frontoparietal dome missing most of the diagnostic posterior part of the parietal.

The specimen is significant as, despite its fragmentary nature, it is one of the oldest known pachycephalosaur remains from North America, and confirms their expected presence in the Wahweap Fm ecosystem.

**Stratigraphy**

Evans et al. (2013b) state that the specimen was recovered from the lower part of the Middle Member of the Wahweap Fm. This is corroborated in the first mention of the specimen (Kirkland & Deblieux, 2005; an SVP abstract specifically referring to the middle mudstone member, and suggestive that the specimen may represent a new species), however, in their stratigraphic column, Evans et al., (2013b) show UMNH VP 11939 occurring in the upper half of the Upper Member of the Wahweap Fm (presumably erroneously).

Here I show the occurrence of UMNH VP 11939 as occurring within the lower half of the Middle Member of the Wahwap Fm, consistent with the prevailing stratigraphic description.

----

### *Acrotholus audeti*

***Acrotholus* *audeti* (Evans et al., 2013a)**

**Milk River Fm, Alberta, CAN**

Holotype TMP 2008.045.0001 is a nearly complete frontoparietal dome; paratype ROM 2962 is an anterior half of a frontoparietal dome (Evans et al., 2013a). An additional specimen ROM 2964 (a damaged frontoparietal dome) was previously referred to by Sullivan (2003) as Pachycephalosaurinae indet., and may belong to *Acrotholus* *audeti* (Evans et al., 2013a).

**Stratigraphy**:

This is the oldest pachycephalosaur from North America.

The holotype, paratype, and possible referred specimen were all collected from the Deadhorse Coulee Mbr of the Milk River Fm (Evans et al., 2013a). It is not completely clear as to their position within the Deadhorse Coulee Mbr, based on Figure s3 of Evans et al. (2013a). however in the text they suggest the holotype was collected 17.1 m below the 34n-33r chron boundary (based on Leahy & Lerbekmo, 1995), and that ROM 2962 and 2964 were collected between 2 and 13 m below the chron boundary. Here I show the taxon occurring in the lowermost part of the Deadhorse Coulee Mbr.

----

## Hadrosauroidea

### *Huehuecanauhtlus tiquichensis*

***Huehuecanauhtlus* *tiquichensis* (Ramirez-Velasco et al., 2011)**

**Unnamed Fm, MEX**

**Material & diagnosis**

Holotype (IGM 6253) comprises a partial left maxilla, dentary fragment, and fragmentary postcrania. Paratype (IGM 6254) comprises a left dentary fragment, two teeth, and one cervical prezygapophysis (Ramirez-Velasco et al., 2011). In phylogenetic analysis, *Huehuecanauhtlus* is recovered as a basal hadrosauroid (Ramirez-Velasco et al., 2011).

**Stratigraphy**

The holotype and paratype were collected from an unnamed formation in Michoacan state, south-central Mexico (Ramirez-Velasco et al., 2011). The unnamed unit has been constrained to the upper part of magnetochron C34n, and has borne a radiometric date of 84 +/- 2.8 Ma (unknown method; Benammi et al., 2006). Thus here the taxon is shown occurring at some point within the Santonian.

----

### *Jeyawati rugoculus*

***Jeyawati* *rugoculus* (McDonald et al. 2010)**

**Moreno Hill Fm, NM**

Holotype specimen MSM P4166 comprises a disarticulated partial skull and postcranium (McDonald et al., 2010).

The type specimen was recovered ~10 m lower in section than the type locality of the ceratopsian *Zuniceratops* (see individual entry) which was recovered from within the lower half of the Moreno Hill Fm, ~45 - 107 m above the formational base (total thickness 151 m). Hence here I show the occurrence of Jeyawati as a single cell positioned in the middle of the Moreno Hill Fm, one cell below that of *Zuniceratops* (this is admittedly only approximate, but lack of taxa of similar age prevents any potential issues for the time being).

----

## Hadrosaurinae

### *Edmontosaurus annectens*

***Edmontosaurus* (*Claosaurus*) *annectens* (Marsh, 1892)**

**Hell Creek and regional equivalents, MT, WY, CO, ND, SD, Alberta, Saskatchewan**

The taxonomic history of *Edmontosaurus* is quite complex with six genera and many species named (*Anatosaurus*, *Anatotitan*, *Claosaurus*, *Edmontosaurus*, *Thespesius*, and *Trachodon*). Campione & Evans (2011) reduce this into just three stratigraphically separated taxa: *Edmontosaurus* *annectens* (Late Maastrichtian), *E. regalis* (Lower Maastrichtian), and *E*. sp (later renamed *Ugrunaaluk* *kuupikensis*; Lower to Upper Maastrichtian). See other entries for *E. regalis* and *U. kuupikensis*.

Here I follow Campione & Evans in showing *E. annectens* as present throughout the Hell Creek Fm, and regional equivalents.

----

### *"Ugrunaaluk" kuukpikensis*

**"*Ugrunaaluk*" *kuukpikensis* (Mori et al., 2016)**

**Prince Creek Fm, AK**

Mori et al. (2016) describe ontogenetically young material previously assigned to *Edmontosaurus* as the new taxon *Ugrunaaluk* *kuukpikensis* from the Prince Creek Fm, Alaska. The holotype (UAMES 12995) is the anterior part of a right premaxilla, with 38 paratypes comprising various cranial and postcranial material (Mori et al., 2016).

Here I show "*Ugrunaaluk*" in inverted commas as it has previously been considered as *Edmontosaurus* sp. by some authors (e.g. Campione & Evans, 2011; Xing et al., 2014), and its erection as a unique genus might cause paraphyly problems. However, following Campione & Evans (2011), I have chosen to plot it separately from *E. regalis* as it seems the relatively restricted stratigraphic range of diagnostic *E. regalis* material (see individual entry) means that "*Ugrunaaluk*" may in future be more strongly supported.

**Age**

Although a number of radiometric dates have been retrieved throughout the Prince Creek Fm (Conrad et al., 1992), these are not especially precise (yielding a range from 68.4 - 72.0 Ma; excluding a clearly incorrect single age of 64.19Ma), and are generally referred to by most authors (e.g. Fiorillo et al., 2010) as yielding an average date of 70.0 +/- 0.3 Ma (all dates recalibrated here).

In 1993, Obradovich reanalyzed a sample from one of the lower horizons sampled by Conrad et al. (1992; it is not specified which specific horizon this was). Obradovich's Ar / Ar date is recalibrated here as 73.4 Ma (see individual entry). As a more recent analysis (Ar / Ar, sanidine), this date is probably more reliable than the previous dates of Conrad et al., (1992). This presents some issues in that it is possible therefore that the various dinosaurs described from the Prince Creek Fm might be as old as 73.4 Ma, which is considerably older than the typically cited age of 70.0 Ma (see above).

For the time being, the range of Prince Creek Fm taxa should probably be considered as unresolved until the radiometric dates are fully reanalyzed using modern methods and standards. I have therefore plotted the range of "*U. kuukpikensis*" as unknown between 73.4 and 68.4 Ma.

----

### *Edmontosaurus regalis*

***Edmontosaurus regalis* (Lambe, 1917b)**

**Horseshoe Canyon Fm, Alberta, CAN.**

Type specimen (CMN 2288; complete skull and partial postcranium) and paratype (CMN 2289; near-complete skull and partial postcranium) originate from the Horseshoe Canyon Fm, Canada (Lambe, 1917). Since the original description, many specimens have been referred to *E. regalis*, including large samples from bonebeds. Although specimens from the Hell Creek and Lance Fms have been referred to *E. regalis*, Campione & Evans (2011) suggest that this material (where diagnostic) should be referred to E. annectens instead, and limit *E. regalis* material to that recovered from the Horseshoe Canyon Fm, Alberta.

**Age**

Eberth et al. (2013) state that *E. regalis* is only definitively identified from the Horsethief Mbr of the Horseshoe Canyon Fm where it is known from bonebed material. However, the presence of morphologically comparable material from slightly lower in section (upper half of the Drumheller Mbr) led Eberth et al. (2013) to tentatively extend the range of *Edmontosaurus*.

Following Eberth et al. (2013) here I show the range of *E. regalis* restricted to the Horsethief Mbr of the Horseshoe Canyon Fm.

----

### *Edmontosaurus* sp.

***Edmontosaurus* sp.**

**Horseshoe Canyon Fm, Alberta, CAN.**

Eberth et al. (2013) state that *Edmontosaurus regalis* is only definitively identified from the Horsethief Mbr of the Horseshoe Canyon Fm where it is known from bonebed material. However, the presence of morphologically comparable material from slightly lower in section (upper half of the Drumheller Mbr) led Eberth et al. (2013) to tentatively extend the range of *Edmontosaurus* down.

Here I limit this material to *Edmontosaurus* sp. and follow Eberth et al. (2013) in showing the range as the upper half of the Drumheller Mbr.

----

### *Maiasaura peeblesorum*

***Maiasaura* *peeblesorum* (Horner & Makela, 1979)**

**Two Medicine Fm, MT**

A bonebed of *Maiasaura* remains is referred to by three locality numbers: TM-003 ("Camposaur"; MOR 005, 238, & 593), TM-151 ("Brandvold site"; MOR 758), TM-158 ("kids dig" MOR 774). These are thought to all be the same bonebed horizon exposed over ~2 sq. km. The holotype (PU 22405) is from the edge of the *Maiasaura* bonebed (Horner, pers. comm. to E. Freedman, 2015).

TM-003 occurs as the uppermost part of lithofacies 3, just below the upper discontinuity (Horner et al., 2001; Varricchio et al., 2010). Varricchio et al. (2010) published an Ar / Ar date of 76.39 Ma (recalibrated) for a bentonite 4.3 m above bonebed TM-003. However, there are slight issues with this date as it is inconsistent with the position of the Upper Discontinuity (10.7 m above the TM-003 bentonite) being correlative with the top of the Comrey Sandstone (unit 2) of the Oldman Fm, Alberta. Since a radiometric date at the top of the conformably overlying unit 3 of the Oldman is dated at 77.03 Ma (recalibrated; see individual entry) then the 76.39 +/- 0.32 Ma date retrieved by Varricchio et al. (2010) is therefore too young. This may be a result of methodological differences, or that the Oldman Fm dates use the more reliable sanidine as the study mineral.

Here then, I have plotted *Maiasaura* as occurring within a possible range from just below the top of the Comrey Sandstone, through to 76.4 Ma.

----

### *Acristavus gagslarsoni*

***Acristavus* *gagslarsoni* (Gates et al., 2011)**

**Lower Two Medicine Fm, MT; Middle Mudstone Mbr, Wahweap Fm, UT**

*Acristavus* comprises Holotype MOR 1155, a complete skull and some postcrania, and referred specimen UMNH VP-16607, a partial skull roof (Gates et al., 2011).

The holotype was recovered from the base of a sandstone channel in the lower Two Medicine Fm, ~40 m above the crystal tuff Ar / Ar dated as 81.04 Ma (Rogers et al., 1993; recalibrated here; Gates et al., 2011).Two further Ar / Ar dates from the lowermost Two Medicine Fm occur ~10 or so above the 81.04 Ma date (see individual entries), the uppermost of which is dated at 80.64 Ma, thereby offering a maximum age for the specimen.

The referred specimen was collected from the upper part of the Middle Mudstone Mbr of the Wahweap Fm, ~ 170 m above the basal contact. The age of this horizon is constrained by a bentonite dated at 79.9 Ma which occurs in the Middle Mbr, ~60 m above the base of the Wahweap Fm, and which is itself underlain by another bentonite Ar / Ar dated at 80.6 Ma which occurs ~40-50 m above the base (Jinnah et al., 2013).

Therefore, here I have plotted the range of *Acristavus* as from 80.6 to 79.8 Ma, although this is only rough and may change with more precise stratigraphic work.

----

### Aff. *Brachylophosaurus* sp.

**Aff. *Brachylophosaurus* sp.**

**Kaiparowits Fm, UT**

Gates et al. (2013) figure a jugal from the lower unit, "near the base" of the Kaiparowits Fm, Utah (UMNH VP 16722) which they suggest is "brachylophosaur-type".

If so, then this is the stratigraphically highest known occurrence of brachylophosaur material.

Here I show an unknown range equivalent to the lower half of the lower unit of the Kaiparowits Fm.

----

### *Brachylophosaurus*

**Brachylophosaurus canadensis (Sternberg, 1953)**

**Oldman Fm, CAN; Judith River Fm, MT**

Freedman Fowler & Horner (2015) review the stratigraphic occurrence of *B. canadensis*. All specimens for which stratigraphic data are known occur within the Comrey Sandstone (unit 2) of the Oldman Fm, Alberta; or the equivalent sandstone unit within the Judith River Fm of Montana. Hence here I show the range of *B. canadensis* as the same as that of the Comrey Sandstone.

----

### *Probrachylophosaurus bergei*

***Probrachylophosaurus* *bergei* (Freedman Fowler & Horner, 2015)**

**Judith River Fm, MT; equivalent to Unit 1, Oldman Fm**

Holotype MOR 2919 comprises a complete skull and most of the postcrania; referred specimen MOR 1097 comprises a partial skull. Both specimens were collected from the Judith River Fm of Rudyard, MT, from the muddy zone equivalent to Unit 1 of the Oldman Fm, Alberta.

The holotype specimen is the only specimen for which precise stratigraphic data is available. It was recovered from 17.5 m above the top of the Taber Coal Zone (Marker A coal)., ~9m below an Ar / Ar date at 79.2Ma, and ~22 m above an Ar / Ar date of 79.5 Ma (Freedman Fowler & Horner, 2015; see individual entries for details of recalibrated bentonites). Hence here I plot *Probrachylophosaurus* occurring between 79.3 -79.2 Ma.

----

### Brachylophosaurini indet.

**Brachylophosaurini indet. (was c.f. *Gryposaurus* sp.; Lucas et al., 2006)**

**Mancos Shale, CO**

**Material & diagnosis**

Specimen MWC 129 consists of disarticulated skull elements and postcrania. Identified cranial elements are the right maxilla, the left and right quadratojugals, incomplete left and right dentaries, the left surangular with retroarticular process, both left and right jugals, fragmentary left and right nasals, left postorbital, part of the right ?frontal, left and right quadrates, a right ?squamosal, and a ?stapes. Postcrania consists of the left scapula, both humeri, a left ulna, both femora, both tibiae, a partial ilium, two dorsal vertebrae, two caudal vertebrae, numerous incomplete centra, and isolated metapodials (Lucas et al., 2006).

Many features of the skeleton mark it out as juvenile (unfused skull and vertebral elements). As such, Lucas et al were not confident ascribing it to any current genus, although they note that it was most similar to *Gryposaurus* / *Kritosaurus*.

**Stratigraphy**

MWC 129 was recovered from the marine Mancos Shale of Colorado, within the Baculites mclearni zone (80.67 - 80.21 Ma; Ogg & Hinnov, 2012). This places it as the oldest known hadrosaurine with affinities to the *Gryposaurus* clade.

**Subsequent analyses**

In their description of the hadrosaurine *Acristavus*, Gates et al. (2011) refer MWC 129 to Brachylophosaurini indet..

----

### *Saurolophus osborni*

***Saurolophus* *osborni* (Brown, 1913b)**

**Tolman Mbr, Horseshoe Canyon Fm, Alberta, CAN**

Holotype (AMNH 5220) comprises a complete skull and postcrania; paratype (AMNH 5221) is a disarticulated skull (Brown, 1913; redescribed by Bell, 2011).

Brown (1913b) states that the specimens were collected from the "Edmonton formation 500 feet below top of beds. Tolman Ferry, Red Deer River, Alberta, Canada.". Eberth et al. (2013) show *S. osborni* restricted to the Tolman Mbr of the Horseshoe Canyon Fm. It is worth noting that although Eberth et al. (2013) show *Saurolophus* ranging for the full duration of the Tolman Mbr, in their appendix they note that the only fully diagnostic material (holotype and paratype) both were recovered from the lowermost part of the Tolman Mbr at the contact zone with the underlying Morrin Mbr.

Hence here I show *S. osborni* as occurring as a definitive occurrence for the lowermost Tolman Mbr, and a possible range extension up to the top of the Tolman Mbr.

----

### *Naashoibitosaurus ostromi*

***Naashoibitosaurus* *ostromi* (Hunt & Lucas, 1993)**

**Denazin Mbr, Kirtland Fm, NM**

The holotype of *Naashoibitosaurus* is a partial skull (NMMNH P16106) described by Hunt & Lucas (1993). Although described as a unique taxon, the specimen has been referred to *Kritosaurus* *navajovius* (e.g. Horner, 1992; Williamson, 2000). However, recent work seems to suggest that *Naashoibitosaurus* may not be related to *Kritosaurus* (Prieto-Marquez, 2013; Freedman Fowler, pers. comm.). Hence here I retain it as a separate taxon.

Williamson (2000) noted that the type specimen was not recovered from the Naashoibito, but in fact derives from the lowermost in the De-Na-Zin Mbr of the Kirtland Fm, approximately the same level as an Ar / Ar date of 73.83 Ma (recalibrated, see individual entry). Hence here I show *Naashoibitosaurus* as occurring from 73.8 to 73.7 Ma.

----

### *Prosaurolophus blackfeetensis*

***Prosaurolophus* *blackfeetensis* (Horner, 1992)**

**Two Medicine Fm, MT**

In his description of *P. blackfeetensis*, Horner (1992) states that the holotype material (MOR 454, partial skull & skeleton) was collected from the "top of the Two Medicine Fm" of Landslide Butte, MT (localities TM-041 & TM-068). Further material assigned to this species (MOR 447 & part of MOR 553) consists of numerous partial skulls and skeletons, however the locality of this material is not explicitly mentioned.

Here I have shown the possible position of *P. blackfeetensis* as the duration of lithofacies 5 of the Two Medicine Fm. It is likely that *P. blackfeetensis* occurs in the upper part of the lithofacies 5 as other taxa collected from Landslide Butte (notably centrosaurines) are all high in section.

----

### *Prosaurolophus maximus*

***Prosaurolophus maximus* (Brown, 1916a)**

**Dinosaur Park Fm, Alberta, CAN**

Known only from the Dinosaur Park Fm, Canada.

Currie (2004) shows the lowest occurrence (CMN-2277) as 684m (just above the 1st appearance of *C. belli*). The final occurrence (ROM-1928) is at 714m: just above the final appearance of *C. irvinensis*.

Mallon et al. (2012) show the lowest occurrence as USNM 12712 at 32.0 m above the Oldman - Dinosaur Park Fm contact, and the uppermost occurrence as ROM 1928 at 52.6 m above the contact.

A 76.10 Ma bentonite occurs 61.5 m above the formational contact; 3.5 m into the Lethbridge Coal Zone, and a 76.39 Ma bentonite occurs at 36 m above the formational contact (see individual entries). Hence here I show *P. maximus* occurring from 76.4 - 76.2 Ma.

----

### *?Gryposaurus alsatei*

**?*Gryposaurus* *alsatei* (Lehman et al., 2016)**

**Javelina Fm, TX**

Lehman et al. (2016) describe a partial skull and postcranium (TMM 46033-1) as the holotype of ?*Gryposaurus alsatei*. The skull lacks the nasal and other elements which would allow confident referral to *Gryposaurus*, but has a frontal autapomorphies which distinguish it as a species unique from other . Lehman et al. (2016) therefore tentatively refer the new species to ?*Gryposaurus*. TMM 46033-1 was recovered from a bonebed which has also yielded remains of at least one other individual (Lehman et al., 2016).

If correctly identified then ?*G. alsatei* would be the latest surviving member of the *Gryposaurus*-*Kritosaurus* clade.

**Stratigraphy**

Lehman et al. (2016) report that TMM 46033-1 was recovered from relatively high in the Javelina Fm, illustrated as ~40 m below the contact with the overlying Black Peaks Fm. The base of the Javelina Fm is not exposed in this area. Elsewhere in the region an ash dated at 69.0 Ma occurs ~40 m below the contact with the Black Peaks Fm (Lehman et al., 2006). Hence here I show ?*G. alsatei* as unknown range, but close to 69 Ma in age.

----

### Cerro del Pueblo *"Kritosaurus"*

***Kritosaurus* sp. (Kirkland et al., 2000; 2006; Prieto-Marquez, 2013)**

**Cerro del Pueblo, Coahuila, Mexico**

Kirkland et al. (2000; 2006) describe a partial skull (IGM 6685) and referred it to *Kritosaurus* sp.. Prieto-Marquez (2013) suggests that (IGM 6685) cannot be distinguished from *K. navajovius*, and so assigns it to this taxon. However, this would be an unusually long range for a single species (although not impossible). Here I am conservative and follow Kirkland et al.'s original referral as *Kritosaurus* sp.

Here I plot the range of the Cerro del Pueblo *Kritosaurus* sp. as unknown within the range of the Cerro del Pueblo Fm itself.

----

### Bearpaw "*Kritosaurus*"

**"*Kritosaurus*" sp.? ("*Kritosaurus* *notablis*": Horner, 1979)**

**Bearpaw Shale, MT**

Horner (1979) describes 2 hadrosaur specimens; YPM-PU 16970: a fragmentary skull and postcrania ascribed to K. notablis; & YPM-PU 16969: a left ulna, partial pelvis, hindlimbs, and complete tail tentatively ascribed to *K. notablis*. Horner (1992) states that after acid preparation, the specimen bears closer resemblance to *K. navajovius*, although there is not enough material to make a positive referral.

Prieto-Marquez (2013) suggests that the cranial remains (YPM-PU 16970) specimens cannot be referred to *Kritosaurus*, and instead refers them to *Gryposaurus* sp.. If this is true then it would mean that *Gryposaurus* and *Kritosaurus* probably are contemporaneous at this point in time, and must therefore represent a speciation event. This issue remains unresolved.

**Age**

The presence of *B. compressus* with bones from this horizon suggests an age of approximately 74.21 - 73.91 Ma (Ogg & Hinnov, 2012). As such, here I plot the range of the Bearpaw *Kritosaurus* as unknown between 74.2 - 73.9 Ma.

----

### *Kritosaurus navajovius*

***Kritosaurus* *navajovius* (Brown, 1910)**

**=*Anasazisaurus* *horneri* (Hunt & Lucas, 1993),**

**Fruitland & Kirtland Fms, NM**

Much debate has surrounded the validity of *Kritosaurus* *navajovius*, collected in 1904 from the Kirtland Fm of New Mexico (Brown, 1910). The holotype skull (AMNH 5799) lacks diagnostic elements, most notably the nasals are extremely fragmentary (Williamson, 2000) making the taxon difficult to diagnose. Despite this, additional specimens from the Fruitland and Kirtland Fm have been assigned to the taxon (e.g. partial skull, USNM 8629; Prieto-Marquez, 2013), including holotypes of other taxa such as *Anasazisaurus horneri* and *Naashoibitosaurus ostromi* (Williamson, 2000).

Here I exclude *Naashoibitosaurus ostromi* from Kritosaurus (see individual entry), but I do include *Anasazisaurus* as a junior synonym of *Kritosaurus navajovius*. This is more out of convenience and familiarity than any solid anatomical grounding. I expect with further study, *Anasazisaurus* might be considered truly a separate taxon (see Prieto-Marquez, 2013).

Prieto-Marquez (2013) also suggests that *K. navajovius* is present in the Cerro del Pueblo Fm. This would extend the stratigraphic range by 2 million years, which would be surprisingly long for a single dinosaur species. Therefore, here I am being conservative and plotting the Cerro del Pueblo specimens separately.

The holotype of *Anasazisaurus* (BYU 12950) is from the Farmington Mbr of the Kirtland Fm (Hunt & Lucas, 1993; Williamson, 2000). The holotype of *Kritosaurus* *navajovius* (AMNH 5799) has imprecise locality data, other than the statement that it was collected from a horizon below the lower conglomerate which forms the basal bed of the Naashoibito Mbr of the Ojo Alamo Fm (Brown, 1910; Williamson, 2000). I expect that this could be further refined as bone from the Denazin Mbr tends to be very heavily mineralized and dark in color, such that with closer inspection it should be possible to tell if the holotype of *Kritosaurus* was from the Denazin Mbr.

Hence the range of *Kritosaurus* *navajovius* (including *Anasazisaurus*) is shown here as unknown within the full range of the Kirtland Fm. However, I expect it should be possible to refine this with further study.

----

### *Gryposaurus monumentensis*

***Gryposaurus* *monumentensis* (Gates & Sampson, 2007)**

**Middle Kaiparowits Fm, UT**

Holotype RAM 6797 is the only specimen currently described (Gates & Sampson, 2005) although additional specimens are mentioned in Gates et al. (2013).

*G. monumentensis* is known only from the middle unit of the Kaiparowits Fm. The holotype specimen was collected from the upper part of the middle Kaiparowits Fm, in between two ash horizons both of which have yielded an Ar / Ar age of 75.51 Ma (Roberts et al., 2013).

Thus *G. monumentensis* is plotted here as 75.5 - 75.6 Ma.

----

### *Rhinorex condrupus*

***Rhinorex* *condrupus* (Gates & Scheetz, 2012)**

**?*Gryposaurus***

**Neslen Fm, UT**

The holotype and only specimen BYU 13258 comprises a partial but mostly articulated skull and skeleton with skin impressions (Gates and Scheetz, 2012). The skull includes the diagnostic nasal-frontal area, and is almost complete although slightly cracked in this important area. Gates and Scheetz chose to erect a new genus, Rhinorex, for the specimen, although it is recovered within a *Gryposaurus* clade, and given its stratigraphic position, might be expected to an intermediate form of *Gryposaurus*. Revision of the taxonomy is beyond the scope of this note.

**Stratigraphy**

BYU 13258 was recovered 12 m above the base of the Neslen Fm.

----

### *Gryposaurus* sp.

***Gryposaurus* sp. (Currie & Russell, 2005; Gates et al., 2013)**

**Dinosaur Park Fm, Alberta, CAN; Kaiparowits Fm, UT**

In the Dinosaur Park Fm, Alberta, one Gryposaurus specimen possibly occurs much higher than the others. Currie & Russell (2005) place ACM 578 (quarry 076; assigned to *G. incurvimanus*) 53.3 m above the Oldman - DPFm contact (see entry on *G. notabilis* for details). This would be slightly below a radiometric date of 76.1 Ma at 61.5 m above the contact, and above a date of 76.3 Ma positioned 36 m above the contact. This occurrence remains tentative as the specimen is not mentioned by Mallon et al. (2012), and the topographic data upon which this is based is shown by Mallon et al. (2012).

However, the persistence of *Gryposaurus* through the Kaiparowits Fm of Utah shows that this genus did not go extinct when its record ends in the DPFm.

*Gryposaurus* sp. in the Lower Kaiparowits Fm, Utah; is this *G. notabilis*?

Gates et al. (2013) note *Gryposaurus* sp. skulls from the lower unit of the Kaiparowits Fm, Utah, and state that these skulls more closely resemble *G. notabilis* than *G. monumentensis*, found in the upper Kaiparowits Fm. Indeed, Gates et al. (2013) state that the only difference between the lower Kaiparowits skulls and *G. notabilis* is size. The specimen numbers for these specimens are slightly confusing; UMNH VP18568 (as given in Gates et al., 2013) is a complete skull mislabeled as UMNH VP1856 on exhibit in the museum itself (pers. obs.) and mislabeled as UMNH VP16667 in figure 19.9 of Gates et al. (2013; despite being referred to, presumably correctly in the text). A skull 20% larger than UMNH VP18568 is referred to as UMNH VP16668, 16669, or even possibly 16667 on the same page (p. 473). It is not discernible which number is correct for this second specimen.

In terms of age, these *Gryposaurus* sp. (cf. *G. notabilis*) skulls occur around a dated ash horizon of 76.46 +/-0.14 Ma from the upper part of the lower Kaiparowits Fm (Roberts et al., 2013), and no higher than an ash date of 75.97 +/- 0.18 Ma in the lower part of the overlying middle Kaiparowits Fm. This time frame is comparable to the middle to upper part of the Dinosaur Park Fm, Alberta (76.39 - 76.10 Ma respectively, see individual notes).

Here I have plotted *Gryposaurus* sp. as ranging from 76.5 Ma to 75.8 Ma to reflect the range as illustrated by Gates et al. (2013) and Roberts et al. (2013).

Note that in their discussion of *Gryposaurus* biostratigraphy, Gates et al. (2013; p. 478-479) suggest that the base of the lower Kaiparowits Fm is dated by a radiometric date of 76.1 Ma, but give no reference. This age is incorrect, and as far as I can tell, 76.1 Ma has never been an age assigned to either the middle or lower Kaiparowits Fm, either recalibrated or urecalibrated (e.g. Roberts et al., 2005; 2013).

----

### *G. notabilis*

***Gryposaurus* *notabilis* (Lambe, 1914a); syn. *G. incurvimanus* (Parks, 1919; 1920a; Prieto-Marquez, 2010)**

**Dinosaur Park Fm, Alberta, CAN**

Mallon et al. (2012) show *Gryposaurus notabilis* occurring in the lower part of the Dinosaur Park Fm (DPFm), Alberta. A similar dataset is presented by Currie & Russell (2005), who present the data as topographic height. Mallon et al. (2012) note that topographic data were uncorrected for dip (~1 degree), and reassessed the data provided in the accompanying CD using topographic maps and other methods, with a cited accuracy of +/- 7m. However, some specimens for which topographic data are provided by Currie & Russell (2005) are not listed in Mallon et al. (2012), and vice versa.

The holotype CMN 2278 occurs 9.7 m above the contact with the Oldman Fm (Mallon et al., 2012; supp. info.). The lowermost specimen (ROM 873) occurs at 0 m (ie. at the Oldman - DPFm contact; Mallon et al., 2012, supp. info.).

The highest cited specimen differs between sets of authors. Mallon et al. (2012; supp. info.) place their highest specimen, ROM 764, at 13.5 m. Currie & Russell (2005; p. 548) have a different highest specimen (ACM 578; quarry 076; assigned to *G. incurvimanus*), suggested to occur 43.6 m above the holotype, ie. 53.3 m above the Oldman contact, or 39.8 m above ROM 764 (which is not included in the dataset of Currie & Russell, 2005). ACM 578 is not included in the dataset of Mallon et al. (2012). If this data is correct, then *G. notabilis* would extend much higher into the Dinosaur Park Fm than suggested by Mallon et al. (2012) based on ROM 764.

Here I have plotted the taxon range as shown by Mallon et al. (2012), with the lowest specimen at 0 m, and the highest at 13.5 m.

The actual age of specimens from the DPFm are constrained by radiometric dates from ashes present 5.5 m below the Oldman-DPFm contact (77.03 Ma); 36 m above the contact (76.3 Ma), and 61.5 m above the contact (76.1 Ma; see individual entries for details on ages).

----

### Rudyard *Gryposaurus*

***Gryposaurus* sp. nov. (Freedman, in prep)**

**Lower Oldman Fm equivalent, Judith River Fm, MT**

A bonebed of multiple individuals of *Gryposaurus* sp. nov. from close to the Canadian border, nr. Rudyard, MT (Freedman, in prep). This part of the Judith River Fm is equivalent to the uppermost Foremost Fm through to the lower part of the Oldman Fm; most of the exposed section is equivalent to unit 1 of the Oldman Fm, sometimes referred to as 'lower muddy' (Eberth, 2005).

The "rocky" bonebed is 24.5m above marker A coal (taber coal equivalent), 29.3 m above an ash dated at 79.52 Ma (recalibrated, this article; originally 78.5 Ma; Goodwin & Deino, 1989; see individual entry), and ~4m below an ash horizon dated at 79.22 Ma (recalibrated, this article; originally 78.2Ma; Goodwin & Deino, 1989; see individual entry).

I have thus plotted this occurrence as 79.2 - 79.3 Ma.

----

### *Gryposaurus latidens*

***Gryposaurus* *latidens* (Horner, 1992)**

**Two Medicine Fm, MT**

Holotype AMNH FARB 5465 comprises a partial skull and postcranium; referred specimen MOR 478 comprises a partial skull and postcranium (Horner, 1992; Prieto-Marquez, 2012).

Horner (1984) states that the holotype *G. latidens* (AMNH FARB 5465) was collected from the Two Medicine River, near Shields Crossing, ~15 km SSW of Cut Bank, Glacier County, Montana. MOR 478 was collected from the Hillside Quarry bonebed at the Harwood Cliff site, Pomdera County, Montana (Horner, 1992).

The holotype locality was relocated and is ~100 m above the Virgelle Sandstone, in sediments "equivalent to the Lower Claggett Shale" (Horner, 1984; p.2). This places it among a series of three dated bentonites at the base of lithofacies 3 of the Two Medicine Fm. The three bentonites are dated at 81.0 Ma, 80.8 Ma, and 80.6 Ma (plagioclase; Rogers et al., 1993; see individual entries; recalibrated here). Thus here I show the possible position of *G. latidens* as between 81.0 - 80.6 Ma.

----

### *Gryposaurus* sp.

***Gryposaurus* sp. (Lucas et al., 2006)**

**Lower Shale, Aguja Fm, TX**

**Material & diagnosis**

Wagner (2001) describes TMM 42452-1, a partial skull of a hadrosaurine, comprising partial left and right nasals, a jugal, and some postcrania, from the Lower Shale of the Aguja Fm., suggesting that it is from a new species of *Kritosaurus* (once synonymized with *Gryposaurus*, but here considered separate). The material listed includes the diagnostic nasals such that a diagnosis should be possible upon official publication.

Wagner also mentions maxillae from the upper shale Mbr of the Aguja Fm that closely resemble *Gryposaurus* (Gates, 2007).

**Stratigraphy**

Here I show the possible range of *Gryposaurus* sp. as the range of the Lower Shale Mbr of the Aguja Fm.

**Subsequent analyses**

The specimen was attributed to hadrosauridae by Prieto-Marquez (2008).

----

## Lambeosaurinae

### Lambeosaurinae sp.

**Lambeosaurinae sp. (Sullivan et al., 2011)**

**Naashoibito Mbr, Ojo Alamo Fm, NM**

A right jugal (SMP VP-1534) and possibly a left humerus (SMP VP-2263) from a lambeosaurin (*Lambeosaurus* + *Corythosaurus* + *Hypacrosaurus*) are described from Naashoibito Mbr of the Ojo Alamo Fm, New Mexico (Sullivan et al., 2011).

Although the material is undoubtedly from a narrow stratigraphic interval, the taxon is here plotted as a large unknown range as the stratigraphic position of the Naashoibito Mbr is not well constrained. However, based on recent stratigraphic work (see individual entry) it is possible that this represents the youngest known lambeosaurine remains in North America, possibly globally.

----

### *Hypacrosaurus altispinus*

***Hypacrosaurus altispinus* (Brown, 1913a)**

**Horseshoe Canyon Fm, Alberta, CAN**

*H. altispinus* was originally defined on postcrania only (Brown, 1913a). However various lambeosaurine cranial remains have since been recovered from the same unit, and consequently attributed to *H. altispinus* (eg. Gilmore, 1924).

Eberth et al., (2013) show *H. altispinus* as occurring from the base of the Morrin Mbr to the top of the Tolman Mbr. This is what is shown here.

----

### *Velafrons coahuilensis*

***Velafrons* *coahuilensis* (Gates et al., 2007)**

**Cerro del Pueblo Fm, Coahuila, Mexico**

Holotype CPC-59 comprises a partial subadult skull (Gates et al., 2007).

The holotype was collected from a horizon ~ 335m from the base of the Cerro del Pueblo (up to 540 m thick). Hence, here *Velafrons* is shown as a single cell within the middle of the inferred range of the Cerro del Pueblo Fm, although age of this unit is possibly less precisely understood (see individual entry).

----

### *Hypacrosaurus stebingeri*

***Hypacrosaurus* *stebingeri* (Horner & Currie, 1994)**

**Two Medicine Fm, MT**

*Hypacrosaurus stebingeri* was described by Horner & Currie (1994) and is known from a complete ontogenetic series (Brink et al., 2014),

Holotype, MOR 549, and various referred specimens were collected from the upper Two Medicine Fm of Badger Creek, Glacier County, Montana, ~80m below the formational contact with the overlying Bearpaw Shale (Brink et al., 2014).

Horner & Currie (1994) reported an Ar / Ar date of 76.025 (recalibrated here) which was recovered from a bentonite "within a few meters" of the Devil's Coulee sites which yield *Hypacrosaurus stebingeri*.

Here I plot the occurrence of *Hypacrosaurus stebingeri* as occurring at 76.1 - 76.0 Ma.

----

### *Corythosaurus*

***Corythosaurus* *casuarius* (Brown, 1914b)**

**Dinosaur Park Fm, Alberta, CAN**

The taxonomy of *Corythosaurus* is complex. Here I follow Ryan and Evans (2005) in considering all specimens as belonging to a single species, *C. casuarius*. However, it is noted that some workers also recognize *C. intermedius* (e.g. Mallon et al., 2012).

*C. casuarius* is known from multiple skulls and skeletons, including a growth series (Ryan & Evans, 2005).

The lowermost specimen of *C. casuarius* (SDMNH 2989) occurs at 1.7 m above the Oldman - Dinosaur Park Fm contact (Mallon et al., 2012). The uppermost specimen (CMN 8676; "*C. intermedius*") occurs 26.5 m above the contact (Mallon et al., 2012).

The actual age of specimens from the DPFm are constrained by radiometric dates from ashes present 5.5 m below the Oldman-DPFm contact (77.03 Ma); 36 m above the contact (76.3 Ma), and 61.5 m above the contact (76.1 Ma; see individual entries for details on ages).

Hence, here I show *C. casuarius* occurring from 76.9 - 76.5 Ma.

----

### Lambeosaurinae sp.

**Lambeosaurinae sp.**

**Two Medicine Fm, MT**

Trexler (2001) notes the presence of a "*Hypacrosaurus* sp." tail from below the uppermost occurrence of *Maiasaura*. If correctly identified, this represents one of the earliest known occurrence of lambeosaurine material in the western interior.

----

### Wahweap lambeosaur

**Lambeosaurinae sp. (Gates et al., 2013)**

**Upper Sandstone Mbr, Wahweap Fm, Utah**

Gates et al. (2013) illustrate a single maxilla which they attribute to a lambeosaurine. Although fragmentary, this is possibly the oldest record of a lambeosaurine from North America (although see *Angulomasticator* entry).

The specimen was collected from the Upper Sandstone Mbr of the Wahweap Fm.

----

### *L. magnicristatus?*

**Latest occurrence of *Lambeosaurus* *magnicristatus*?**

**Bearpaw Shale, MT**

Horner (1979) describes a specimen designated as *L. magnicristatus* from the Bearpaw Shale of Western Montana. The specimen (PU21905) consists of fragmentary left and right maxillae, and part of a left dentary.

The specimen was designated *L. magnicristatus* by Lull & Wright (1942), however the fragmentary nature of the specimen brings this diagnosis into doubt (Horner, 1979).

Specimens of *Baculites* *compressus* found with bones of animals from the same deposit suggest an 'early Bearpaw age'. However, here I show a variable arrow depicting a conservative duration for the Bearpaw Shale, reflecting uncertainty in precise age of the specimen.

----

### *Lambeosaurus magnicristatus*

***Lambeosaurus* *magnicristatus* (Sternberg, 1935)**

**Dinosaur Park Fm, Alberta, CAN**

The taxonomy of *Lambeosaurus* is fairly complex. Here I follow Ryan and Evans (2005) in recognizing only two species: *L. lambei*, and *L. magnicristatus*. However, it is noted that some workers also recognize *L. clavintalis* (e.g. Mallon et al., 2012).

Ryan & Evans (2005) note that *L. magnicristatus* is known from two specimens, the holotype CMN 8705 was collected from Dinosaur Provincial Park, and the referred specimen TMP 66.04.01 was colected from the Manyberries region in southern Alberta.

Precise stratigraphic data is only published for the holotype specimen, although Ryan & Evans (2005) state that both specimens derive from the uppermost Dinosaur Park Fm. Mallon et al. (2012) state that the holotype (CMN 8705) was recovered 46.7 m above the Oldman - Dinosaur Park Fm contact, immediately beneath the Lethbridge Coal Zone.

The actual age of specimens from the DPFm are constrained by radiometric dates from ashes present 5.5 m below the Oldman-DPFm contact (77.03 Ma); 36 m above the contact (76.3 Ma), and 61.5 m above the contact (76.1 Ma; see individual entries for details on ages).

Hence, here I show *L. magnicristatus* occurring from 76.2 - 76.1 Ma.

----

### *Lambeosaurus lambei*

*Lambeosaurus lambei* (Parks, 1923)

Dinosaur Park Fm, CAN

The taxonomy of *Lambeosaurus* is fairly complex. Here I follow Ryan and Evans (2005) in recognizing only two species: *L. lambei*, and *L. magnicristatus*. However, it is noted that some workers also recognize *L. clavintalis* (e.g. Mallon et al., 2012).

*L. lambei* is known from at least 14 skulls (Ryan & Evans, 2005).

The lowermost specimen of *L. lambei* (CMN 8703' "L. clavintalis") occurs at 17.1 m above the Oldman - Dinosaur Park Fm contact (Mallon et al., 2012). The uppermost specimen (ROM 794) occurs 45.3 m above the contact (Mallon et al., 2012).

The actual age of specimens from the DPFm are constrained by radiometric dates from ashes present 5.5 m below the Oldman-DPFm contact (77.03 Ma); 36 m above the contact (76.3 Ma), and 61.5 m above the contact (76.1 Ma; see individual entries for details on ages).

Hence, here I show *L. lambei* occurring from 76.6 - 76.3 Ma.

----

### *Angulomasticator*

***Angulomasticator* *daviesi* (Wagner & Lehman, 2009)**

**Aguja Fm, TX**

The holotype, and only known specimen, TMM-43681-1, is an abraded maxilla which "differs from all other hadrosaurs in possession of [an] abrupt 45 [degree] ventral deflection of [the] anterior maxilla and occlusal plane such that occlusal surfaces of mesial teeth are well ventral to distal teeth in articulation with an unusual bend" (Wagner & Lehman, 2009).

**Stratigraphy**

Along with other abraded bone fragments, TMM-43681-1 was collected from a dark grey mudstone within a series of channel deposits, in the uppermost part of the Upper Shale Mbr of the Aguja Fm, ~10m below the contact with the overlying Javelina Fm (Wagner & Lehman, 2009).

It is possible that this horizon is the basalmost unit of the Javelina depositional sequence, and hence chronostratigraphically unrelated to the underlying remainder of the Ajuga Fm. In their sequence stratigraphic interpretation, Atchley et al. (2004) show the series of channel sands 10-15m below the Javelina-Aguja contact as being the lowermost part of their Fluvial Aggradational Cycle set 2, a package of sediments ~60m thick, which constitutes the lower 40-50m of the Javelina Fm . Atchley et al. propose that these beds likely are chronostratigraphically related to the Javelina depositional sequence, rather than the Aguja, with which they are classified (especially plausible in the light of reidentification of *Bravoceratops* material; see Fowler in prep). In which case, *Agulomasticator* might instead be Maastrichtian.

For the time being, I plot *Angulomasticator* as an unknown position within the hypothesized range of the Upper Shale Mbr of the Aguja Fm.

**Implications**

Davies (1983) describes isolated material from the lower parts of the Upper Aguja Fm (Quarry WPA1: immediately above the contact with the marine tongue) which he tentatively assigns to Lambeosaurinae. If correct, these represent the oldest lambeosaurine remains from North America.

----

### *P. tubicen*

***Parasaurolophus* *tubicen* (Wiman, 1931b)**

**Denazin Mbr, Kirtland Fm, NM**

*P. tubicen* is known from two complete skulls, both collected from the San Juan Basin, New Mexico. The holotype skull (PMU R1250) was described by Wiman (1931). A new complete skull of *Parasaurolophus* *tubicen* (NMMNH-P-25100) was recovered from the Denazin Mbr of the Kirtland Fm (Sullivan & Williamson, 1999).

**Stratigraphy**

The NMMNH specimen (Sullivan & Williamson, 1999) was recovered from 9m below the top of the Denazin Mbr. Stratigraphic data for the holotype is not known, however its preservation is distinctively black: which is the typical colouration of bone from the Denazin Mbr, and quite unlike the more chocolate to orange-brown bone colour of the underlying members. Hence both the holotype and the new skull are shown here to pertain from the Denazin mbr.

Age of *P. tubicen* is constrained by two Ar / Ar dates for the Denazin Mbr. The first dated at 73.83 Ma occurs <5m above the base of the Denazin Mbr, the second dated at 73.49 Ma occurs 4.9m below the upper contact (Fassett & Steiner, 1997; recalibrated here; see individual entries).

Hence here I plot *P. tubicen* occurring between 73.6 - 73.5 Ma.

----

### *P. cyrtocristatus* **(New Mexico)**

**Parasaurolophus cyrtocristatus (Ostrom, 1961)**

**Fruitland Fm, NM, USA.**

Holotype FMNH P27393 comprises a complete skull with postcranium (Ostrom, 1961). A partial juvenile skull is referred to *Parasaurolophus* sp. by Sullivan & Bennett (2000).

**Stratigraphy**

Both specimens are recorded from the Fruitland Fm (Ostrom, 1961; Sullivan & Bennett, 2000; Sullivan & Williamson, 1999). The exact horizon is not stated, however, given that the Neh-nah-ne-zad Mbr of the Fruitland is not fossiliferous, it is likely that this specimen was collected from the Fossil Forest Mbr, which is therefore what I show here.

**Status**

Even if the short crest of *P. cyrtocristatus* is eventually demonstrated to indicative of a juvenile status, it is possible that the species *P. cyrtocristatus* may be conserved for the New Mexico (type) material as this derives from the Fruitland Fm (Ostrom, 1961), and is hence stratigraphically distinct from both the type species *P. walkeri* (lowermost Dinosaur Park Fm; Mallon et al., 2012) and the later occurring *P. tubicen* (De-Na-Zin Mbr, Kirtland Fm, Wiman, 1931). However, a long-crested *Parasaurolophus* specimen has yet to be found in the Fruitland Fm, hence we can be uncertain of its morphology.

----

### *P. cyrtocristatus* **(Utah)**

*Parasaurolophus cyrtocristatus* (Ostrom, 1961)

Kaiparowits Fm, UT, USA

Weishampel and Jensen (1979) note that *P. cyrtocristatus* is recorded from the lower third of the Kaiparowits Fm, based on an eroded skull (BYU 2467). A more complete specimen UCMP 143270 was noted by Sullivan & Williamson (1999). More recent fieldwork has recovered at least eight partial skulls, including a juvenile (Gates et al., 2013; Farke et al., 2013) all of which derive from the sandstones in the middle unit of the Kaiparowits Fm.

More specific stratigraphic data are not offered, However, the stratigraphic column of Roberts et al. (2013) shows "ornithopods" occurring ~10-15 m below ash bed KBC-109, dated at 75.51 Ma. Few remains are shown occurring beneath a 76.46 Ma ash date in the lower Kaiparowits.

Hence here I show the range of *P. cyrtocristatus* within the Kaiparowits as between 76.4 - 75.5 Ma.

----

### *Parasaurolophus* sp*.*

***Parasaurolophus* sp. (Evans et al., 2007; 2009; Mallon et al., 2012)**

**Dinosaur Park Fm, Alberta, CAN**

Four specimens are referred to *Parasaurolophus* sp., a juvenile braincase (CMN 8502; Evans et al., 2007) an adult braincase (CMN 52845; Evans et al., 2009), and two specimens (TMP 1992.053.2001; UALVP 300) in the supporting information of Mallon et al. (2012).

**Stratigraphy**

All specimens derive from the Dinosaur Park Fm, Alberta. Mallon et al. (2012) shows the *Parasaurolophus* sp. specimens TMP 992.053.2001 and UALVP 300 shown occurring at 23.0 and 28.5 m (respectively) above the Oldman - Dinosaur Park Fm contact. Adult braincase CMN 52845 is thought to derive from the middle of the Dinosaur Park Fm, although this is not certain (Evans et al., 2009). No stratigraphic data is available for the juvenile braincase.

The actual age of specimens from the DPFm are constrained by radiometric dates from ashes present 5.5 m below the Oldman-DPFm contact (77.03 Ma); 36 m above the contact (76.3 Ma), and 61.5 m above the contact (76.1 Ma; see individual entries for details on ages).

Hence, here I show *Parasaurolophus* sp. occurring from 76.7 - 76.6 Ma.

----

### *Parasaurolophus walkeri*

***Parasaurolophus* *walkeri* (Parks, 1922)**

**Dinosaur Park Fm, Alberta, CAN**

ROM 768 is the only known skull of *P. walkeri* that preserves a complete elongated crest (Parks, 1922).

**Stratigraphy**

Mallon et al. (2012) show the holotype (ROM 768) occurring 1.9 m above the Oldman - Dinosaur Park Fm contact.

The actual age of specimens from the DPFm are constrained by radiometric dates from ashes present 5.5 m below the Oldman-DPFm contact (77.03 Ma); 36 m above the contact (76.3 Ma), and 61.5 m above the contact (76.1 Ma; see individual entries for details on ages).

Hence, here I show *P. walkeri* occurring from 76.9 - 76.8 Ma.

----

## Sauropoda

### *Alamosaurus*

***Alamosaurus* *sanjuanensis* (Gilmore, 1922)**

**Ojo Alamo Fm, NM; North Horn Fm, UT; Javelina Fm, TX**

The remains of titanosaurid sauropods have been recovered from a number of formations in North America, all of which outcrop in the lower US, and Mexico (Naashoibito Mbr, Ojo Alamo Fm, NM; Javelina Fm, TX; North Horn Fm, UT; Chihuahua, MEX). All of these remains are attributed to *Alamosaurus* *sanjuanensis*.

Here I show the possible occurrence of *Alamosaurus* as a fixed box around the 69 Ma datum (Javelina Fm, TX), with unknown range arrows illustrating the hypothesized timespans of the North Horn Fm and Naashoibito Mbr of the Ojo Alamo Fm.

**Taxonomic note**

Since the type material of *A. sanjuanensis* was described (left scapula & ischium, Gilmore, 1922) new discoveries of titanosaurid remains have shown the type *Alamosaurus* material to be undiagnostic at anything other than family titanosauridae level (making *Alamosaurus* *sanjuanensis* technically a nomen nudum, Lucas & Sullivan, 2000). Thus, *'Alamosaurus'* is recognized as a form taxon to which all sauropod remains from the Late Cretaceous of North America are assigned (Lucas & Sullivan, 2000). This has led to *'Alamosaurus'* being used to infer same-age relationships for N.Am Late Cretaceous units that yield sauropod bones: a convoluted problem caused by circular taxonomic issues.

Upchurch et al. (2004) rediagnose *A. sanjuanensis* as lacking caudal ribs from caudal vertebra 9 onwards, and having an acute rather than broad craniolateral process of the sternal plate. However, this is based on a partial titanosaurid skeleton from the North Horn Fm, Utah, attributed to *A. sanjuanensis* (Gilmore, 1946b). Since the holotype material is undiagnostic, this skeleton cannot be referred to *A. sanjuanensis*. Indeed, the North Horn material may be much younger than other *A. sanjuanensis* occurrences in New Mexico and Texas.
