# Supplementary figures and images for: Revised geochronology, correlation, and dinosaur stratigraphic ranges of the Santonian-Maastrichtian (Late Cretaceous) formations of the Western Interior of North America
Source: PLoS One. 2017 Nov 22;12(11):e0188426. doi: 10.1371/journal.pone.0188426 (PMC5699823; doi:10.1371/journal.pone.0188426)

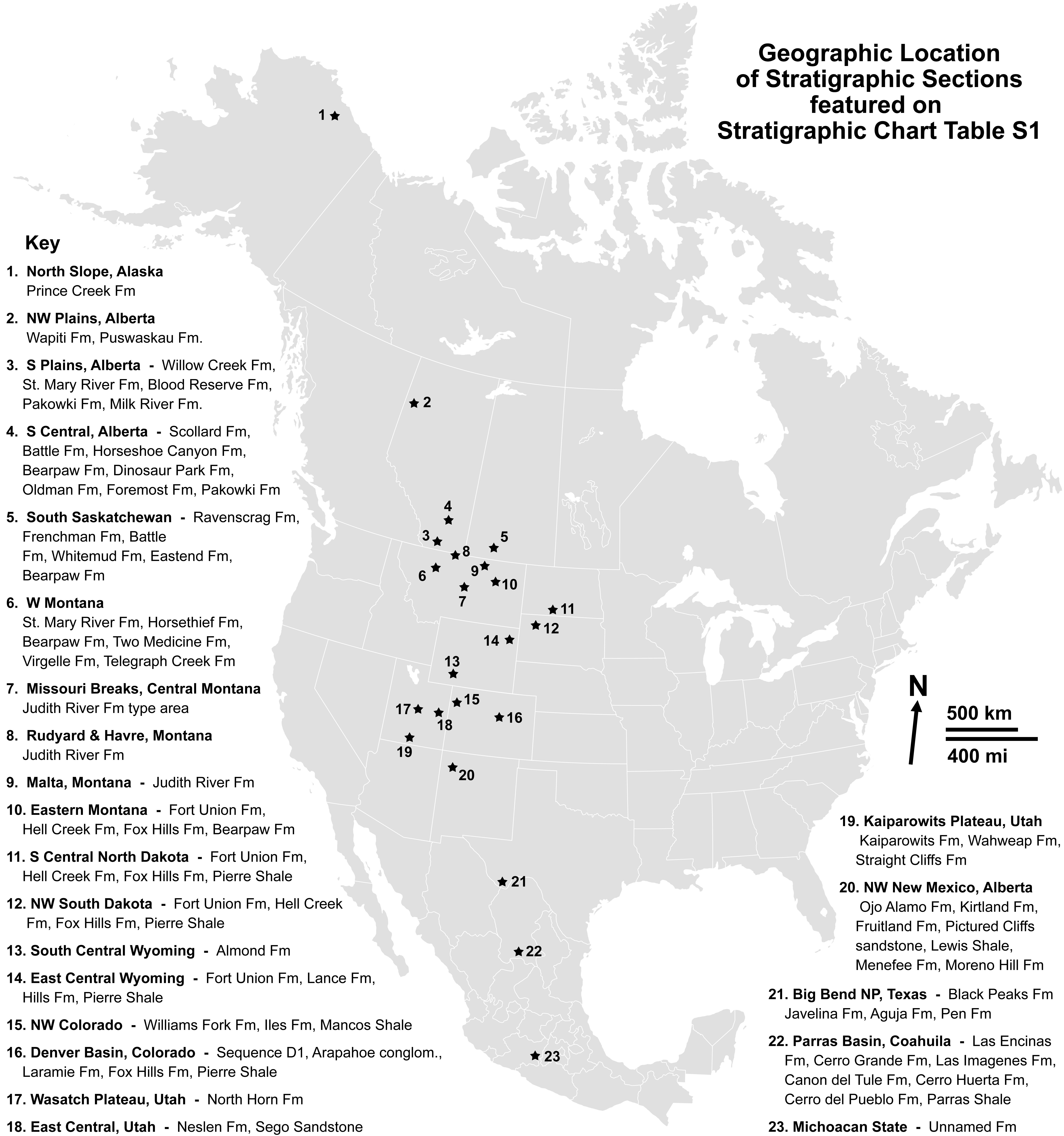

Supplement: S2 Fig — This map shows the geographic location of the different stratigraphic sections shown in stratigraphic chart S1 Table. (JPG) [file pone.0188426.s006.jpg]
